# SO_2_ Emissions in China – Their Network and Hierarchical Structures

**DOI:** 10.1038/srep46216

**Published:** 2017-04-07

**Authors:** Shaomin Yan, Guang Wu

**Affiliations:** 1 Bioscience and Biotechnology Research Center, Guangxi Academy of Sciences, 98 Daling Road, Nanning, Guangxi, 530007, China

## Abstract

SO_2_ emissions lead to various harmful effects on environment and human health. The SO_2_ emission in China has significant contribution to the global SO_2_ emission, so it is necessary to employ various methods to study SO_2_ emissions in China with great details in order to lay the foundation for policymaking to improve environmental conditions in China. Network analysis is used to analyze the SO_2_ emissions from power generation, industrial, residential and transportation sectors in China for 2008 and 2010, which are recently available from 1744 ground surface monitoring stations. The results show that the SO_2_ emissions from power generation sector were highly individualized as small-sized clusters, the SO_2_ emissions from industrial sector underwent an integration process with a large cluster contained 1674 places covering all industrial areas in China, the SO_2_ emissions from residential sector was not impacted by time, and the SO_2_ emissions from transportation sector underwent significant integration. Hierarchical structure is obtained by further combining SO_2_ emissions from all four sectors and is potentially useful to find out similar patterns of SO_2_ emissions, which can provide information on understanding the mechanisms of SO_2_ pollution and on designing different environmental measure to combat SO_2_ emissions.

Of various air pollutants, SO_2_ is extremely important because it can be adsorbed onto the surface of mineral dust and serves as adsorbed sulfite that is oxidized to form sulfate^1^. Therefore, the oxidation of SO_2_ to sulfate is significant although atmospheric sulfate can come from volcanic ash, sea spray and sulfur-containing species^2^. As the precursor for sulfate and sulfuric acid, which play crucial roles in the nucleation of fine particles^3^, the SO_2_ concentration is always high during new particle formation events^4^,^5^. Specifically, a long-term study from 1985 to 2000 showed that 1% increase in SO_2_ emission in East Asia resulted in 1.29% increase in surface aerosol sulfate concentration^6^. In general, the density of particle is about 1.7 g cm^−3 ^7, which was determined according to the density of sulfate and a major aerosol component in Beijing^8^. The formation of sulfate from SO_2_ can be facilitated by ozone^9^, surface defect sites of mineral dust^10^,^11^, photooxidation^12^,^13^, and NO_x_ because of their synergistic effect on the surface of mineral dust^14^. Furthermore, dimethylsulfide can also be converted into sulfate^15^,^16^.

Besides its adverse effect on human health, SO_2_ is harmful to environments^17^,^18^ because sulfate aerosols turn out the major source for the growth of fine particles, which subsequently lead to visibility impairment, acid rain, haze formation and photochemical smog. Indeed China and East Asia have been experiencing a continuous dimming in solar radiation since 2000^19^,^20^,^21^, acid rains^22^,^23^,^24^ and haze^25^,^26^,^27^. The primary anthropogenic source of SO_2_ emission is burning of coal and heavy oil whose sulfur content is usually higher than coal, and decomposition of Na_2_SO_4_^28^. The national average sulfur contents in coal in China were 1.08% and 1.02% in 2000 and 2005^29^,^30^,^31^,^32^, and 0.22% and 0.05% for diesel and gasoline^31^. The anthropogenic SO_2_ emission in China has accounted for one quarter of global emission since 1990s^30^,^33^,^34^. Although the concentration of SO_2_ was about 60–120 ppb during heavy haze episodes in cities in North China^15^, the coexistence of NO_x_ from vehicles speeds up the formation of fine particles.

SO_2_ emissions have been studied in different locations in China with ground surface observations, aircraft measurements and remote sensing in satellite^35^,^36^,^37^,^38^,^39^,^40^,^41^,^42^. Several models have been applied to simulating of SO_2_ chemistry and transport in East Asia^43^,^44^, whereas overestimation of SO_2_ concentrations is typical in modeling of sulfate aerosols on a global scale^45^. The consensus is that SO_2_ concentrations vary greatly in both spatial and temporal distributions^14^. So far, most studies have been concentrated on East, Middle and South China, where industrialized and heavily populated cities are located. For SO_2_ observations, the ground surface SO_2_ observations are less subject to the long-range transport, which usually takes place over 2000 meters in free troposphere^46^.

As a matter of fact, not many studies were oriented to the SO_2_ emissions beyond 2008 in China^30^,^47^. Surely 2008 is a turning point in this regard, and the coal consumption increased to 2740 Mt from 1271 Mt in 2000 at 10.1% annually before 2008, to which power generation contributed around 65%^47^. In 2008, the estimated total anthropogenic SO_2_ emissions were about 31.3 Tg in China^43^. On the other hand, Beijing Olympic Games in 2008 had a huge impact on the reduction of SO_2_ emission^48^,^49^.

Basically, Chinese government statistical reports and yearbooks carry SO_2_ emissions from both fuel combustion and non-combustion sources^47^. Recently, the very detailed SO_2_ concentrations on ground surface observations became available as MIX, the mosaic Asian anthropogenic emission inventory for 2008 and 2010^50^. The MIX documents the emissions from 40.125 E to 179.875 E and from 20.125 S to 89.875 N in 0.25 × 0.25 (∼25 km × 25 km) grid, including 2168 monitoring stations in China that collect monthly emission data from residential, industrial, power generation, transportation and agricultural sources^50^.

These two periods in MIX are important because (i) the Chinese government required all coal-fired power plants to install flue gas desulfurization (FGD) devices in 2005^51^, therefore the analysis on these two periods should reflect the situation after implementation of this regulation, for example, FGD reached an operation rate of 97% in July 2007 in Jiangsu province^32^; (ii) the proportion of FGD systems reached 81.7% in 2008^52^ despite of that the FGD penetration was planned to reach 71% in 2008 and 73% in 2010^53^; and (iii) it was estimated that a half of 3.1 billion-tons of coal consumed in 2010 was attributed to power generation in China^54^.

To date, a number of models have been applied to studying SO_2_ emissions, whereas network analysis has not yet been used as far as our knowledge is concerned. Nevertheless, this approach looks promising not only because each model has its own advantage and disadvantage and network can simultaneously analyze spatial and temporal relationship, but also because the SO_2_ concentration reflects the emission level in its surrounding area^47^. Additionally, the characteristic of network analysis decides its suitability to study SO_2_ emissions because network analysis studies various interrelationships in terms of graphic nodes and edges. For instances, nodes can be cities and edges can be roads between cities in transport network, nodes can be proteins and edges can be interactions between proteins in protein interaction network, nodes can be people and edges can be their friendships in social network, etc. In the context of SO_2_ emission, we define a node as an observation station, and an edge between two nodes as the correlation between two SO_2_ emission profiles. The use of correlation to define an edge between nodes can easily be found in other research fields such as gene co-expression network^55^. Important characters of SO_2_ include: (i) SO_2_ has a shorter lifetime than sulfate, (ii) the SO_2_ aloft in the free troposphere has a longer lifetime than the SO_2_ on ground surface^42^, and (iii) the SO_2_ emission from transportation sector is a mobile source emission^56^. These characters lay the foundation for network analysis of SO_2_ emissions. As the correlation in network analysis needs to capture a meaningful sense in two SO_2_ emission profiles, so a short lifetime pollutant will give more sensible correlation than stagnant pollutants. Hence, this study applies network analysis to exploring SO_2_ emission in China in 2008 and 2010. Based on the results of network analysis, we end up with building the hierarchical structure of SO_2_ emissions from power generation, industrial, residential and transportation sectors in order to get an integrated view.

## Results and Discussion

### SO_2_ emission from power generation sector

**Figure 1:**
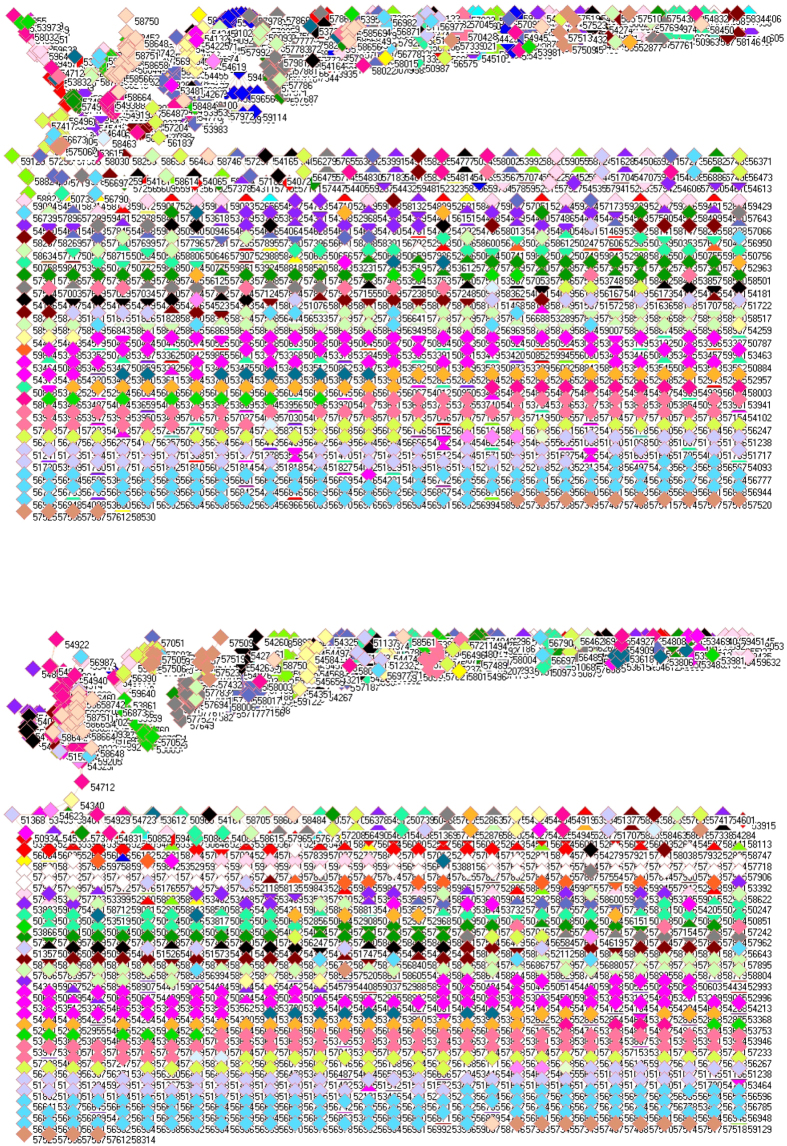
Network of SO_2_ emissions from power generation sector in China monitored by 1558 stations in 2008 (upper panel) and by 1636 stations in 2010 (lower panel). Yellow are 21 monitoring stations in Anhui, cyan 11 in Beijing, lime green 48 in Fujian, red 68 in Gansu, blue 64 in Guangdong, pink 60 in Guangxi, white 17 (upper panel) and 63 (lower panel) in Guizhou, orange 12 in Hainan, purple 96 in Hebei, cadet blue 80 in Henan, teal blue 79 in Heilongjiang, olive green 60 in Hubei, gray 69 in Hunan, black 48 in Jilin, maroon 55 in Jiangsu, light green 67 in Jiangxi, light yellow 42 in Liaoning, magenta 95 in Inner Mongolia, midnight blue 18 in Ningxia, dandelion 34 in Qinghai, wild straw berry 46 (upper panel) and 77 (lower panel) in Shandong, forest green 33 in Shanxi, salmon 67 in Shaanxi, light sky blue 4 in Shanghai, green yellow 114 in Sichuan, lavender 5 in Tianjin, light faded green 1 in Tibet, light purple 66 in Xinjiang, corn flower blue 97 (upper panel) and 98 (lower panel) in Yunnan, light orange 54 in Zhejiang, and tan 27 in Chongqing. (For details, see additional legends to Fig. 1 in Supplementary information files).

Figure 1 shows the network of SO_2_ emission from power generation sector in 2008 (upper panel) and 2010 (lower panel). In this type of figures, a symbol represents a monitoring station with its code, and 31 colors donate to 22 provinces, 4 municipalities and 5 autonomous regions in China. A line between two symbols interprets the SO_2_ emission profiles in the two monitoring stations having a good correlation. A cluster aggregates the symbols that more densely connect each other within the given cluster but sparsely connect with the symbols in other clusters. At first glance, network analysis discovers many isolated nodes, which occupy middle and lower parts of both panels in Fig. 1, namely, 782 and 816 SO_2_ emission profiles do not have any good correlations with any place in 2008 and 2010, respectively.

These isolated places include not only very geographically isolated and remote places such as Mohe (Heilongjiang, 50136), but also provincial capitals such as Guangzhou (59287), Haikou (59758), Hohhot (53463), Nanjing (58238), Nanning (59431), Urumqi (51469) and Xining (52866). In addition, several famous polluted places are included such as Baotou (Inner Mongolia, 53446), Datong (Shanxi, 53487) and Handan (Hebei, 53892), which are famous for their steel and coal industries. Also, several tourism destinations appear as isolated places such as Dali (Yunnan, 56751), Jiuzhaigou (Sichuan, 56097), Lijiang (Yunnan, 56651), Qinhuangdao (Hebei, 54449), Shangri-La (Yunnan, 56543), etc.

Because SO_2_ emission profiles in isolated places have no resemblance with any SO_2_ emission profiles, so the higher number of isolated places in 2010 would suggest the effects of implementation of FGD because it could theoretically eliminate common patterns in SO_2_ emission profiles and led to the local characteristic to dominate SO_2_ emission profiles^32^,^51^,^52^,^53^,^54^.

**Figure 2:**
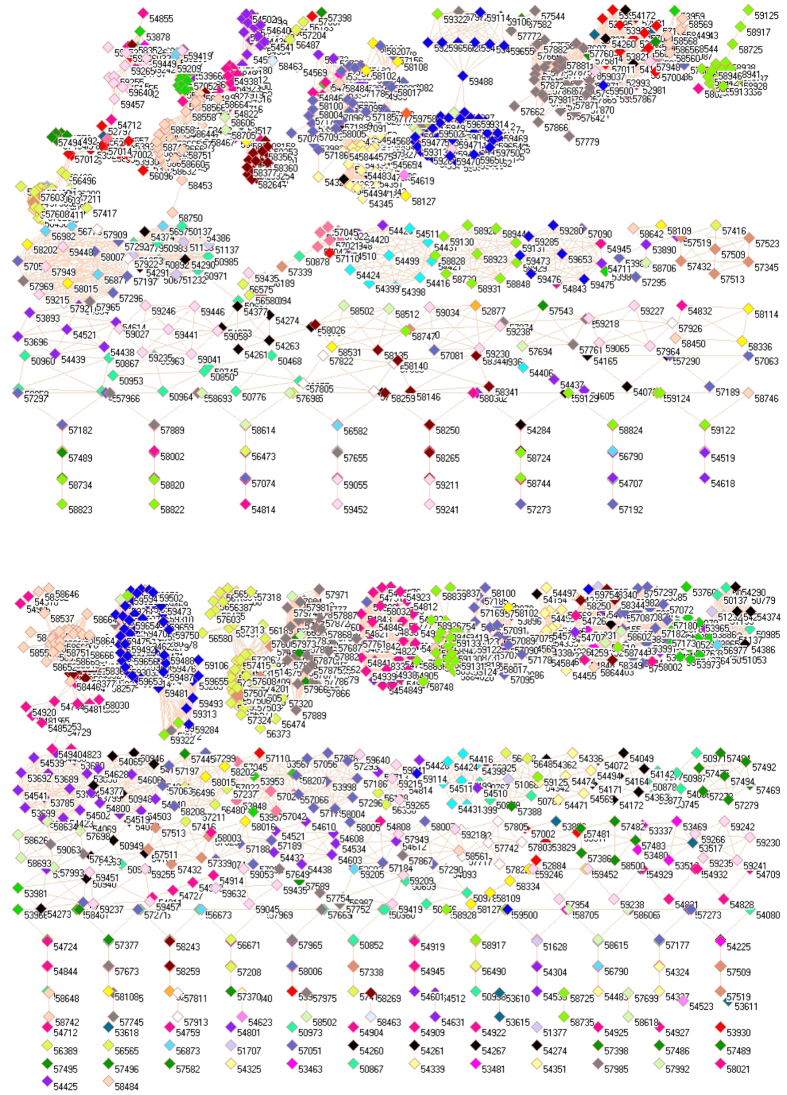
Network of SO_2_ emissions from power generation sector without isolated places in 2008 (upper panel) and 2010 (lower panel).

Figure 2 demonstrates the network of SO_2_ emissions from power generation sector without those isolated places in 2008 (upper panel) and 2010 (lower panel). Technically, Fig. 2 is a subset of Fig. 1 for the purpose of better visualization. An important feature in Fig. 2 is that the same colored symbols did not gather in a single cluster but spread in two or more clusters. For example, lime green symbols at right-upper corner in upper panel represent the places in Fujian province, however, a small cluster with lime green symbols can be found at middle of upper panel. As a result, the SO_2_ emissions from power generation sector in Fujian can be primarily divided into two clusters, indicating that each cluster has its own characteristic and requires different measures to control the emission even within the same province.

In social network analysis, the node with most edges is the central point, from where information propagates. If we apply this concept to Fig. 2, we found that the most connected nodes came from Sichuan province (the first left-middle cluster with green yellow symbols in upper panel and the third left-upper cluster with green yellow symbols in lower panel) and Guangdong province (the fourth right-upper cluster with blue symbols in upper panel and the second left-upper cluster with blue symbols in lower panel). Although these nodes do not represent major power generation places, their geographical locations could be the determinant factor for their similar SO_2_ emission profiles.

### SO_2_ emissions from industrial sector

**Figure 3:**
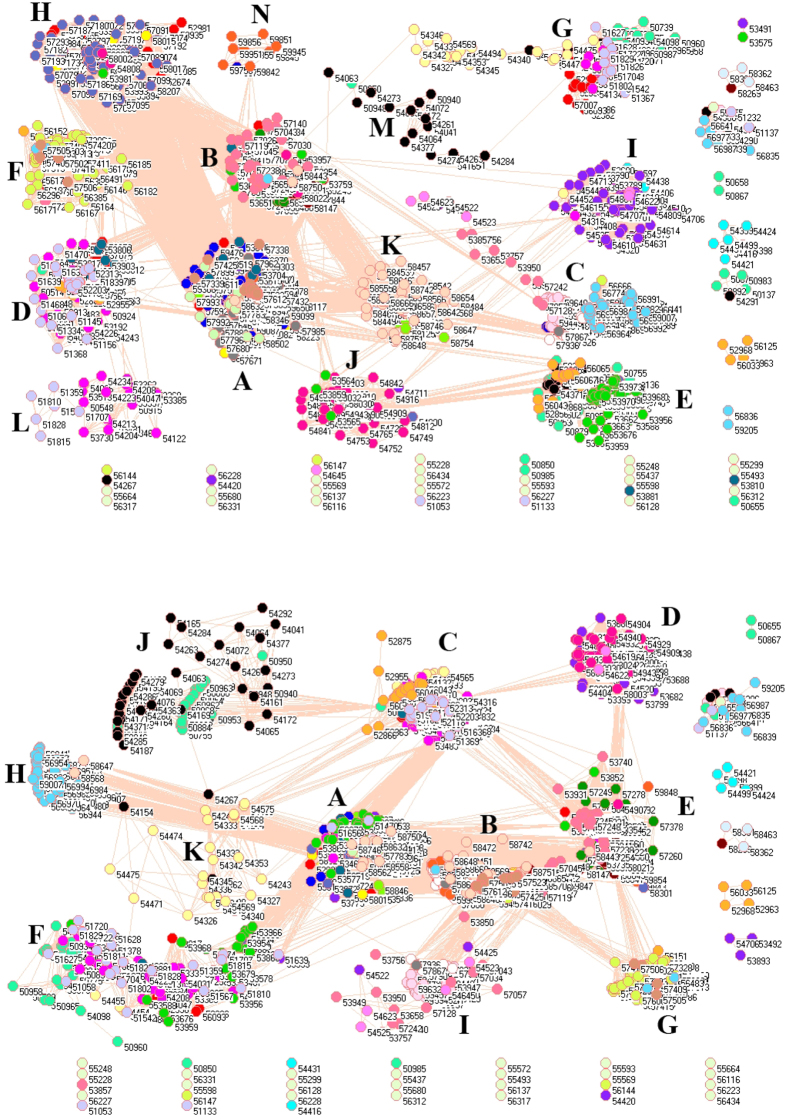
Network of SO_2_ emissions from industrial sector in 2008 (upper panel) and 2010 (lower panel). (For details, see additional legends to Fig. 3 in Supplementary information files).

Figure 3 illustrates the network of SO_2_ emissions from industrial sector in 2008 (upper panel) and 2010 (lower panel). As can be seen, there are less isolated places in Fig. 3 than in Fig. 1, so the SO_2_ emissions from industrial sector have more common features than that from power generation sector. This implies that it is somewhat easier to implement a common measure to reduce the SO_2_ emissions from industrial sector than that from power generation sector.

For 2008, network analysis discovers 23 clusters, among them 33 isolated places are classified as a single cluster and presented at the bottom of upper panel. The 8 clusters, which do not have any connection with outside clusters, are placed at right-hand periphery of upper panel. Then clusters A to N construct a large cluster, because it does not have any connection with any node in peripheral clusters. In this large cluster, the number of connections between the clusters from A to N varies greatly. For example, cluster M connects with cluster G through a single node (54284, Donggang) and with cluster B through a single node (54063, Fuyu), but there are many connections among clusters A, B, H and K. A cluster does not necessarily contain the places from the same province because network analyzes the correlation of two SO_2_ emission profiles for any two places.

Once again, we look at the node with most edges. Strikingly, the places, whose SO_2_ emission profiles correlate best with other places in both panels, are the places in the northern part of Anhui province although not many huge industrial enterprises are located there. Because the northern part of Anhui province is the terminal of Great North China Plain, therefore SO_2_ could be accumulated in this region due to strong winds from North China. This explanation is reasonable because the long-range transport of SO_2_ takes place over 2000 meters in free troposphere^46^.

For 2010, network analysis discovers 18 clusters, among them 31 isolated places are considered as a single cluster at the bottom of lower panel, and 6 clusters are presented at right-hand periphery of lower panel. Eventually, 11 clusters construct a large cluster, which does not have any connection with outside nodes, and this large cluster contains 1674 places across China, which is realistic because this large cluster includes almost all industrial areas across China. Naturally, each cluster does not exclusively include the places from a single province. For example, cluster J includes not only the places in Jilin province (black colored symbols) but also the places from Heilongjiang province (teal blue symbols), so these places have similar emission pattern, which is plausible because both provinces are located together.

Let us have a close look at two clusters. Cluster A is characterized as follows: (i) containing 100% monitoring stations in Fujian, 98.51% in Jiangxi, 88.41% in Hunan and 73.44% in Guangdong, which are four provinces geographically connected together; (ii) containing 88.89% monitoring stations in Henan and 64.94% in Shanxi, which are two provinces geographically connected together; (iii) containing 22.22% monitoring stations in Ningxia and 20.59% in Gansu, which are geographically connected together; and (iv) containing 25% monitoring stations in Shanghai, 15.79% in Anhui, 11.67% in Hubei, 5.1% in Hebei, 3.16% in Inner Mongolia, 2.6% in Shandong and 1.79% in Jiangsu, which are geographically corridors between most accounted provinces, for example, Anhui is located between Henan and Jiangsu, and between Henan and Zhejiang. Cluster B contains (i) 88.89% monitoring stations in Guizhou and 85.19% in Chongqing, and both are geographically connected together with 2.04% monitoring stations in Yunnan and 1.72% in Sichuan; (ii) 82.46% monitoring stations in Anhui and 77.78% in Zhejiang, and both are geographically connected together with 14.29% monitoring stations in Jiangsu, 1.49% in Jiangxi and 1.02% in Hubei; (iii) 66.67% monitoring stations in Hainan and 25% in Guangdong, and both are geographically connected together; and (iv) 20.9% monitoring stations in Shaanxi, 11.11% in Ningxia, 10.14% in Hunan, 2.94% in Gansu and 2.6% in Shanxi, and these five provinces form geographically a belt. These clusters perfectly classify similar pattern of SO_2_ emissions from different places, suggesting that environmental measures could be adopted in consideration of what these clusters are composed of.

### SO_2_ emissions from residential sector

**Figure 4:**
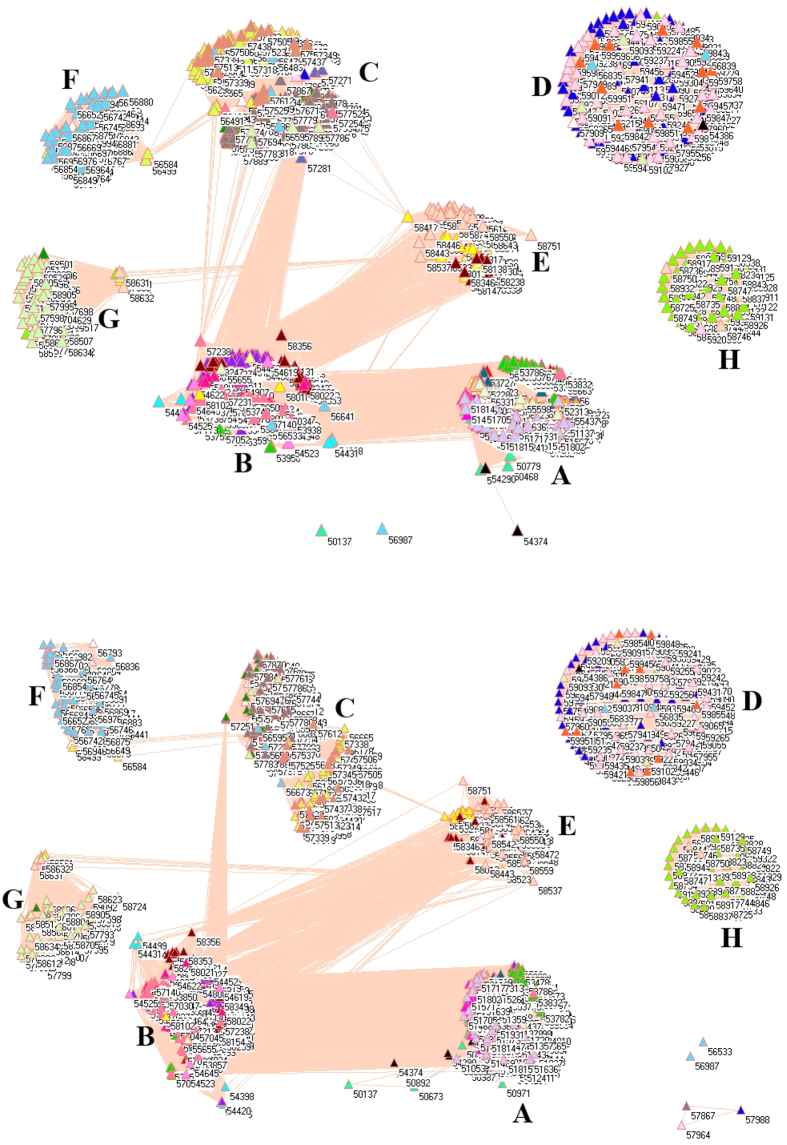
Network of SO_2_ emissions from residential sector in 2008 (upper panel) and 2010 (lower panel). (For details, see additional legends to Fig. 4 in Supplementary information files).

Figure 4 describes the network of SO_2_ emissions from residential sector in 2008 (upper panel) and 2010 (lower panel). At first glance, time did not have great impact on the SO_2_ emissions from residential sector, because network analysis did not find great difference between 2008 and 2010, namely, people’s living style did not change too much in terms of SO_2_ emissions between 2008 and 2010.

Some isolated places were very particular in their geographic locations: Beijicun (North Pole Village, 50137) appeared in 2008, Jinping (56987) just opposite to Vietnam appeared in both 2008 and 2010, and Gongshan (56533) near Myanmar and Tibet appeared in 2010.

In 2008 the isolated cluster D includes 100% monitoring stations in Hainan, 96.88% in Guangdong, 91.67% in Guangxi, 4.48% in Jiangxi, 4.17% in Fujian, 2.08% in Jilin, 2.04% in Yunnan and 1.59% in Guizhou. In 2010 it includes 100% in Hainan, 98.44% in Guangdong, 93.33% in Guangxi, 4.48% in Jiangxi, 4.17% in Fujian, 2.08% in Jilin, 2.04% in Yunnan and 1.59% in Guizhou. Another isolated cluster H includes 91.67% monitoring stations in Fujian, 3.7% in Zhejiang, 1.67% in Guangxi in 2008; in 2010 it includes 91.67% monitoring stations in Fujian and 3.7% in Zhejiang. These findings once more confirmed that the SO_2_ emissions from residential sector did not change significantly from 2008 to 2010. Particular attention should be given to cluster B, which in 2008 includes 100% monitoring stations in Beijing, 100% in Shandong, 100% in Tianjin, 95.92% in Hebei, 91.04% in Shaanxi, 90.12% in Henan, 82.14% in Jiangsu, 12.99% in Shanxi, 10.53% in Anhui, 4.55% in Tibet, 2.94% in Gansu, 2.38% in Liaoning, 2.04% in Yunnan, 1.67% in Hubei and 1.06% in Inner Mongolia. In 2010 it includes 100% monitoring stations in Beijing, 100% in Shandong, 100% in Tianjin, 95.92% in Hebei, 92.54% in Shaanxi, 90.12% in Henan, 80.36% in Jiangsu, 12.99% in Shanxi, 10.53% in Anhui, 4.76% in Liaoning, 4.55% in Tibet, 4.41% in Gansu and 1.05% in Inner Mongolia. Truly, cluster B is the rampant haze region, Beijing-Tianjin-Hebei, however network analysis suggests that this region should also include Shandong, Henan and Jiangsu because they have the same emission patterns.

### SO_2_ emissions from transportation sector

**Figure 5:**
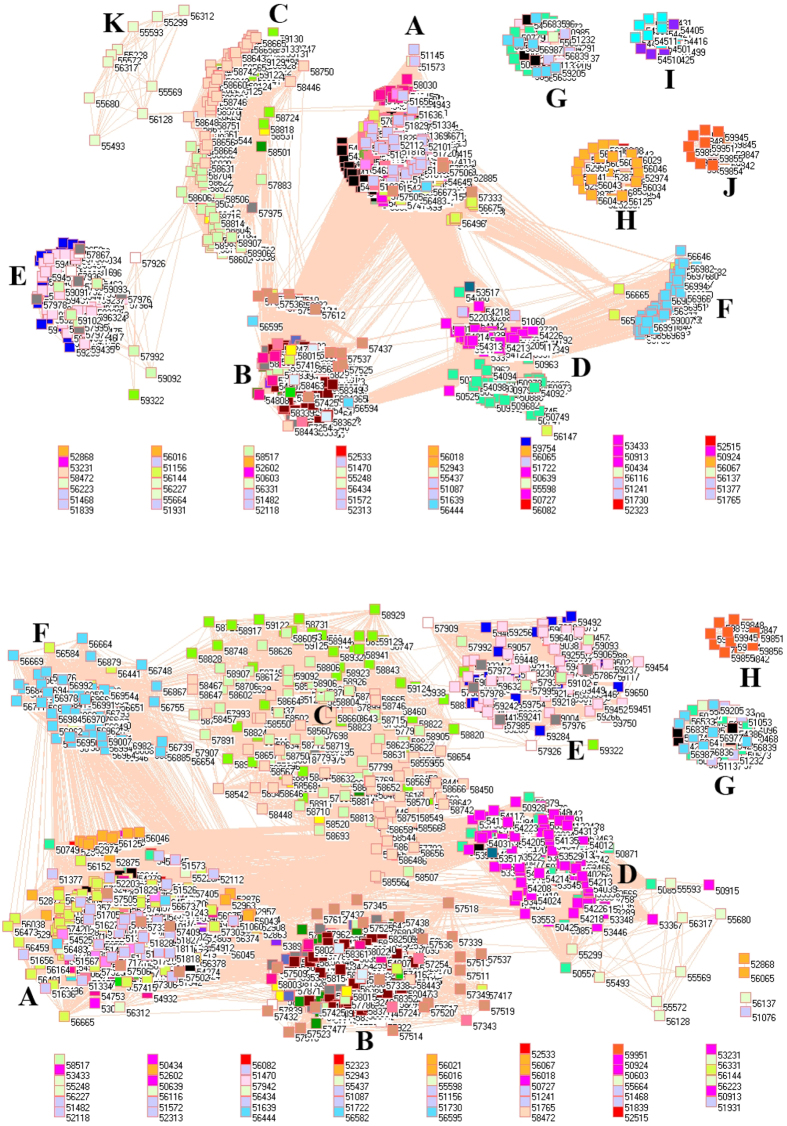
Network of SO_2_ emissions from transportation sector in 2008 (upper panel) and 2010 (lower panel). (For details, see additional legends to Fig. 5 in Supplementary information files).

Figure 5 pictures the network of SO_2_ emissions from transportation sector in 2008 (upper panel) and 2010 (lower panel). From these two panels, we can see the integration process of transportation sector because there were 11 clusters in 2008 but 8 clusters in 2010, i.e. clusters I, J and K were integrated into other clusters due to the development of highway systems in China. For instance, several places were integrated to cluster A in 2010 from cluster K in 2008 such as Shiquanhe (55228) and Linzhi (56312) in Tibet.

In 2010, clusters A, B, C, D and F interweave together with many connections between clusters, implying a high level transportation between them. Indeed, these five clusters include 100% monitoring stations in Anhui, 100% in Beijing, 100% in Chongqing, 93.75% in Fujian, 94.12% in Gansu, 1.67% in Guangxi, 93.65% in Guizhou, 100% in Hebei, 87.34% in Heilongjiang, 100% in Henan, 98.33% in Hubei, 92.75% in Hunan, 91.58% in Inner, 100% in Jiangsu, 91.04% in Jiangxi, 91.67% in Jilin, 100% in Liaoning, 100% in Ningxia, 76.47% in Qinghai, 100% in Shaanxi, 100% in Shandong, 100% in Shanghai, 100% in Shanxi, 99.14% in Sichuan, 100% in Tianjin, 50% in Tibet, 69.7% in Xinjiang, 88.78% in Yunnan and 98.15% in Zhejiang. Thus, only 2 provinces are excluded (Guangdong and Hainan). Such a compact network does represent the biggest transportation network in China, which should require a common measure to reduce SO_2_ emission.

### SO_2_ emissions characterized from four sectors

**Figure 6:**
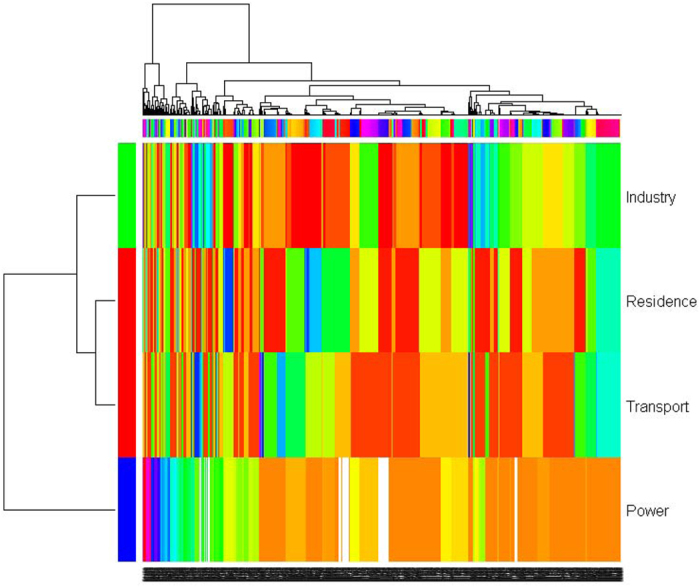
Heatmap and hierarchical cluster analysis on SO_2_ emissions from power generation, industrial, residential and transportation sectors in 2010. Different colors in heatmap indicate the membership of SO_2_ emissions with respect to clusters in power generation, industrial, residential and transportation sectors (Figs. 1, 2, 3, 4, 5). The dendrograms on the top and left-hand side indicate hierarchical relationship. The labels are superimposed each other because 1744 monitoring stations are included in analysis (the hierarchical relationships of 1744 monitoring stations can be found in Table A7 in Supplementary information files).

In order to get a balanced overview, Figure 6 puts all the SO_2_ emissions in terms of their cluster membership from all four sectors together with the use of heatmap and hierarchical cluster analysis in 2010. This hierarchical cluster analysis furthermore defines the patterns of SO_2_ emissions because network analysis can stratify SO_2_ emissions according to their similarity, but cannot define the hierarchical structure among clusters. On the right-hand side with respect to dendrogram structure on the left-hand side, we can see that the SO_2_ emissions from residential and transportation sectors are more similar, and then they merge with the SO_2_ emissions from industrial sector, and finally merge with the SO_2_ emissions from power generation sector. Clearly, the SO_2_ emission from power generation sector is different from others. Because 1744 monitoring stations are included in analysis, the labels are superimposed at the bottom of figure, but their hierarchical relationship is visible on the top of Fig. 6 (the hierarchical relationships of 1744 monitoring stations can be found in Table A7 in Supplementary information files). For example, an initial hierarchical relationship begins from merging of Dangshan (Anhui, 58015) and Funan (Anhui, 58202), and then Mianchi (Henan, 57063). For another example, Runan (Henan, 57197) merges with Xiaoxian (Anhui, 58016), and then merges with Bozhou (Anhui, 58102), which come from the merging of Guangshan (Henan, 57299) and Bozhou (Anhui, 58102) (Table A7 in Supplementary information files). Basically, this hierarchical structure is potentially useful to find out similar patterns of SO_2_ emissions, which can provide information on understanding the mechanisms of SO_2_ pollution and on designing different environmental measures to combat SO_2_ emissions.

In this study, we add the hierarchical structure analysis to study SO_2_ emission, which reveals interrelationship between sectors and emissions. To some extend, SO_2_ emission networks are somewhat similar to PM_2.5_ emission networks, which are reasonable because PM_2.5_ formation is closely connected to SO_2_ emission. Therefore both studies can give us more general patterns on hazardous emissions in China.

## Conclusions

To our best knowledge, this is the first study to analyze SO_2_ emissions in China using network analysis, and the results demonstrate the heterogeneity of SO_2_ emissions from different sectors and their dynamic changes. The obtained clusters and connectivity provide clear views of SO_2_ emission patterns from various places across China. Together with the hierarchical structure, we can trace similar emission patterns in detail, which shed new insights into the understanding of mechanisms of SO_2_ pollution. In particular, such analyses can help to make policy decision for different regions according to their pattern of SO_2_ emissions.

## Materials and Methods

### Data

The monthly SO_2_ emission data are available in the mosaic Asian anthropogenic emission inventory for 2008 and 2010 (MIX), which covers the emissions from 40.125 E to 179.875 E and from 20.125 S to 89.875 N in 0.25 × 0.25 (∼25 km × 25 km) grid^50^ including 2168 monitoring stations in China.

The grid in MIX is smaller than that used previously^52^, consequently several monitoring stations may be happened in the same grid. For the sake of single measurement per grid, only one monitoring station was selected for network analysis. Also, incomplete datasets were excluded from our analysis.

### Analysis

As abovementioned, an edge between two nodes dedicates a relationship. Thus, we define a Pearson’s correlation as a measure to determine whether two SO_2_ emission profiles obtained from two monitoring stations are relevant. In particular, we consider it significant when a Pearson’s correlation is larger than 0.95, whose root is approximate to 0.92 as a criterion to evaluate a method^57^. iGraph R package (http://igraph.org/) and Pajek^58^ were used in network analysis. Hierarchical structure was built using hierarchical cluster analysis in R package.

## Additional Information

**How to cite this article:** Yan, S. and Wu, G. SO_2_ Emissions in China – Their Network and Hierarchical Structures. *Sci. Rep.* **7**, 46216; doi: 10.1038/srep46216 (2017).

**Publisher's note:** Springer Nature remains neutral with regard to jurisdictional claims in published maps and institutional affiliations.

## Supplementary Material

Supplementary Information
