# Supplementary material for: SO2 Emissions in China – Their Network and Hierarchical Structures
Source: Sci Rep. 2017 Apr 7;7:46216. doi: 10.1038/srep46216 (PMC5384192; doi:10.1038/srep46216)
Supplement: Supplementary Information [file srep46216-s1.pdf]

Supplementary information files

SO_2 Emissions in China – Their Network and Hierarchical Structures

Shaomin Yan and Guang Wu

List of Supplementary information files:

Table A1. SO_2 emissions from industrial sector in 2008
Table A2. SO_2 emissions from industrial sector in 2010
Table A3. SO_2 emissions from residential sector in 2008
Table A4. SO_2 emissions from residential sector in 2010
Table A5. SO_2 emissions from transportation sector in 2008
Table A6. SO_2 emissions from transportation sector in 2010
Table A7. Merging process of SO_2 emissions from power generation, industrial, residential and transportation sectors in 2010 produced by R package
Additional Legends to Figures

Table A1. SO_2 emissions from industrial sector in 2008

Cluster A	1. Anhui Taihe, 58109; Anhui Tianzhushan, 58112; Anhui Suixi, 58113; Anhui Woyang, 58114; Anhui Leysin, 58117; Anhui Mengcheng, 58118; Anhui Suzhou, 58122; Anhui Lingbi, 58125; Anhui Sixian, 58126; Anhui Huaiyuan, 58127; Anhui Guzhen, 58128; Anhui Wuhe, 58129; Anhui Fuyang, 58203; Anhui Yingshang, 58210; Anhui Fengtai, 58212; Anhui Huoqiu, 58214; Anhui Changfeng, 58220; Anhui Fengyang, 58222; Anhui Mingguang, 58223; Anhui Dingyuan, 58225; Anhui Laian, 58234; Anhui Chuzhou, 58236; Anhui Jinzhai, 58306; Anhui Luan, 58311; Anhui Huoshan, 58314; Anhui Shucheng, 58316; Anhui Yuexi, 58317; Anhui Tongcheng, 58319; Anhui Feixi, 58320; Anhui Feidong, 58323; Anhui Chaohu, 58326; Anhui Lujiang, 58327; Anhui Wuwei, 58329; Anhui Hanshan, 58330; Anhui Wuhu, 58334; Anhui Maanshan, 58336; Anhui Wuhuxian, 58338; Anhui Susong, 58417; Anhui Tongling, 58429; Anhui Nanling, 58431; Anhui Jingxian, 58432; Anhui Xuancheng, 58433; Anhui Jingde, 58435; Anhui Ningguo, 58436; Anhui Huangshan, 58437; Anhui Guangde, 58441; Anhui Langxi, 58442; Anhui Qimen, 58520; Anhui Yixian, 58523; Anhui Shexian, 58530; Anhui Tunxi, 58531<br>2. Fujian Guangze, 58724; Fujian Ninghuai, 58818; Fujian Yongding, 59113; Fujian Zhaoan, 59320; Fujian Yunxiao, 59322<br>3. Gansu Kongtong, 53915; Gansu Qinan, 57002<br>4. Guangdong Lechang, 57988; Guangdong Renhua, 57989; Guangdong Nanxiong, 57996; Guangdong Yangshan, 59075; Guangdong Ruyuan, 59081; Guangdong Shaoguan, 59082; Guangdong Fogang, 59087; Guangdong Yingde, 59088; Guangdong Shixing, 59090; Guangdong Wengyuan, 59094; Guangdong Lianping, 59096; Guangdong Heping, 59099; Guangdong Pingyuan, 59106; Guangdong Longchuan, 59107; Guangdong Xingning, 59109; Guangdong Jiaoling, 59114; Guangdong Daipu, 59116; Guangdong Meixian, 59117; Guangdong Huaiji, 59270; Guangdong Guangning, 59271; Guangdong Sihui, 59276; Guangdong Sanshui, 59279; Guangdong Qingyuan, 59280; Guangdong Huadu, 59284; Guangdong Conghua, 59285; Guangdong Guangzhou, 59287; Guangdong Dongguan, 59289; Guangdong Longmen, 59290; Guangdong Heyuan, 59293; Guangdong Boluo, 59297; Guangdong Wuhua, 59303; Guangdong Zijin, 59304; Guangdong Jiexi, 59306; Guangdong Fengshun, 59310; Guangdong Chaozhou, 59312; Guangdong Raoping, 59313; Guangdong Puning, 59314; Guangdong Shantou, 59316; Guangdong Luoding, 59462; Guangdong Yangchun, 59469; Guangdong Xinxing, 59470; Guangdong Yunfu, 59471; Guangdong Heshan, 59473; Guangdong Kaiping, 59475; Guangdong Xinhui, 59476; Guangdong Enping, 59477; Guangdong Panyu, 59481; Guangdong Doumen, 59487; Guangdong Zhuhai, 59488; Guangdong Huidong, 59492; Guangdong Shenzhen, 59493; Guangdong Haifeng, 59500; Guangdong Lufeng, 59502; Guangdong Suixi, 59650; Guangdong Gaozhou, 59653; Guangdong Lianjiang, 59654; Guangdong Huazhou, 59655; Guangdong Wuchuan, 59656; Guangdong Maoming, 59659; Guangdong Leizhou, 59750

	5. Guizhou Hezhang, 56598; Guizhou Puan, 56792; Guizhou Tongzi, 57606; Guizhou Zhengan, 57625; Guizhou Wuchuan, 57634; Guizhou Dafang, 57708; Guizhou Xifeng, 57718; Guizhou Kaiyang, 57719; Guizhou Meitan, 57722; Guizhou Fenggang, 57723; Guizhou Shiqian, 57734; Guizhou Yuping, 57739; Guizhou Nayong, 57800; Guizhou Fuquan, 57821; Guizhou Guiding, 57824; Guizhou Taijiang, 57834; Guizhou Jianhe, 57835; Guizhou Leishan, 57837; Guizhou Ziyun, 57910; Guizhou Luodian, 57916; Guizhou Dushan, 57922; Guizhou Rongjiang, 57932<br>6. Hainan Chengmai, 59843; Hainan Changjiang, 59847; Hainan Baisha, 59848; Hainan Tunchang, 59854<br>7. Hubei Padang, 57355; Hubei Hefeng, 57543; Hubei Shishou, 57571; Hubei Jianli, 57573; Hubei Honghu, 57581; Hubei Chibi, 57582; Hubei Tongcheng, 57589; Hubei Wuxue, 58501<br>8. Hunan Longshan, 57544; Hunan Sangzhi, 57554; Hunan Zhangjiajie, 57558; Hunan Lixian, 57565; Hunan Nanxian, 57574; Hunan Huarong, 57575; Hunan Yueyang, 57584; Hunan Baojing, 57642; Hunan Yongshun, 57643; Hunan Guzhang, 57646; Hunan Jishou, 57649; Hunan Yuanling, 57655; Hunan Luxi, 57657; Hunan Taoyuan, 57661; Hunan Changde, 57662; Hunan Hanshou, 57663; Hunan Taojiang, 57666; Hunan Anhua, 57669; Hunan Yuanjiang, 57671; Hunan Xiangyin, 57673; Hunan Ningxiang, 57678; Hunan Milo, 57680; Hunan Pingjiang, 57682; Hunan Changsha, 57687; Hunan Liuyang, 57688; Hunan Xinhuang, 57744; Hunan Zhijiang, 57745; Hunan Xupu, 57752; Hunan Hongjiang, 57754; Hunan Lengshuijiang, 57760; Hunan Xinhua, 57761; Hunan Loudi, 57763; Hunan Shaoshan, 57771; Hunan Xiangxiang, 57772; Hunan Shuangfeng, 57774; Hunan Nanyue, 57776; Hunan Hengshan, 57777; Hunan Youxian, 57779; Hunan Zhuzhou, 57780; Hunan Liling, 57781; Hunan Tongtao, 57845; Hunan Lengshuitan, 57865; Hunan Yongzhou, 57866; Hunan Qiyang, 57868; Hunan Qidong, 57870; Hunan Hengyangxian, 57871; Hunan Hengyang, 57872; Hunan Changning, 57874; Hunan Hengnan, 57875; Hunan Anren, 57881; Hunan Chaling, 57882; Hunan Yongxing, 57887; Hunan Guangxi, 57889; Hunan Shuangpai, 57962; Hunan Daoxian, 57965; Hunan Ningyuan, 57966; Hunan Jiangyong, 57969; Hunan Xintian, 57971; Hunan Chenzhou, 57972; Hunan Guiyang, 57973; Hunan Jiahe, 57974; Hunan Lanshan, 57975; Hunan Yizhang, 57976; Hunan Linwu, 57978; Hunan Zixing, 57981; Hunan Rucheng, 57985; Hunan Jianghua, 59063<br>9. Jiangsu Fengxian, 58012; Jiangsu Peixian, 58013; Jiangsu Suining, 58130; Jiangsu Suyu, 58131; Jiangsu Xuyi, 58138; Jiangsu Pukou, 58237; Jiangsu Nanjing, 58238; Jiangsu Gaochun, 58339; Jiangsu Yixing, 58346; Jiangxi Xiushui, 57598; Jiangxi Tonggu, 57694; Jiangxi Wanzai, 57698; Jiangxi Shanggao, 57699; Jiangxi Pingxiang, 57786; Jiangxi Lianhua, 57789; Jiangxi Fenyi, 57792; Jiangxi Yichun, 57793; Jiangxi Xinyu, 57796; Jiangxi Anfu, 57798; Jiangxi Jianxian, 57799; Jiangxi Xiaping, 57883; Jiangxi Yongxin, 57891; Jiangxi Wanan, 57895; Jiangxi Suichuan, 57896; Jiangxi Taihe, 57899; Jiangxi Chongyi, 57990; Jiangxi Nankang, 57992; Jiangxi Ganxian, 57993; Jiangxi Xinfeng, 57995; Jiangxi

	Jiujiang, 58502; Jiangxi Ruichang, 58503; Jiangxi Lushan, 58506; Jiangxi Wuning, 58507; Jiangxi Dean, 58508; Jiangxi Hukou, 58510; Jiangxi Pengze, 58512; Jiangxi Duchang, 58517; Jiangxi Poyang, 58519; Jiangxi Jingdezhen, 58527; Jiangxi Wuyuan, 58529; Jiangxi Jingan, 58600; Jiangxi Fengxin, 58601; Jiangxi Anyi, 58602; Jiangxi Gaoan, 58605; Jiangxi Nanchang, 58606; Jiangxi Yugan, 58612; Jiangxi Jinxian, 58614; Jiangxi Wannian, 58615; Jiangxi Dongxiang, 58618; Jiangxi Linchuan, 58619; Jiangxi Dexing, 58622; Jiangxi Shangraoxian, 58623; Jiangxi Guixi, 58626; Jiangxi Qianshan, 58629; Jiangxi Yushan, 58634; Jiangxi Xinjian, 58693; Jiangxi Xiajiang, 58704; Jiangxi Yongfeng, 58705; Jiangxi Lean, 58706; Jiangxi Chongren, 58710; Jiangxi Jinxi, 58712; Jiangxi Nancheng, 58715; Jiangxi Nanfeng , 58718; Jiangxi Lichuan, 58719; Jiangxi Xingguo, 58804; Jiangxi Ningdu, 58806; Jiangxi Guangchang, 58813; Jiangxi Shicheng, 58814; Jiangxi Yudu, 58905; Jiangxi Huichang, 58906; Jiangxi Anyuan, 58907; Jiangxi Quannan, 59091; Jiangxi Longnan, 59092; Jiangxi Dingnan, 59093; Jiangxi Xunwu, 59102; Jiangxi Shangli, 57783<br>10. Ningxia Huinong, 53519; Ningxia Helan, 53610; Ningxia Pingluo, 53611; Ningxia Taole, 53615; Ningxia Zhongwei, 53704<br>11. Shaanxi Langao, 57247; Shaanxi Zhenping, 57343<br>12. Sichuan Wusheng, 57417<br>13. Yunnan Yiliang, 56594<br>14. Zhejiang Kaihua, 58537; Zhejiang Changshan, 58631; Zhejiang Jiangshan, 58632<br>15. Chongqing Chengkou, 57333; Chongqing Kaixian, 57338; Chongqing Yunyang, 57339; Chongqing Wuxi, 57345; Chongqing Wushan, 57349; Chongqing Dianjiang, 57425; Chongqing Wanzhou, 57432; Chongqing Zhongxian, 57437; Chongqing Shizhu, 57438; Chongqing Wansheng, 57509; Chongqing Tongliang, 57510; Chongqing Beibei, 57511; Chongqing Yubei, 57513; Chongqing Bishan, 57514; Chongqing Jiangjin, 57517; Chongqing Banan, 57518; Chongqing Nanchuan, 57519; Chongqing Changshou, 57520; Chongqing Fengdu, 57523; Chongqing Wulong, 57525; Chongqing Qianjiang, 57536; Chongqing Pengshui, 57537; Chongqing Qijiang, 57612
Cluster B	1. Anhui Tianchang, 58240<br>2. Fujian Wuyishan, 58730; Fujian Pucheng, 58731; Fujian Songxi, 58735; Fujian Zhenghe, 58736; Fujian Shouning, 58744; Fujian Zhouning, 58747; Fujian Fuan, 58748; Fujian Zherong, 58749; Fujian Jianning, 58822; Fujian Gutian, 58836; Fujian Youxi, 58837; Fujian Minhou, 58844; Fujian Lianjiang, 58848; Fujian Wuping, 58917; Fujian Datian, 58923; Fujian Zhangping, 58926; Fujian Huaan, 58928; Fujian Anxi, 58929; Fujian Jiuxianshan, 58931; Fujian Xiuyu, 58938; Fujian Changle, 58941; Fujian Fuqing, 58942; Fujian Pingtan, 58944; Fujian Putian, 58946; Fujian Changtai, 59122; Fujian Zhangpu, 59129; Fujian Tongan, 59130; Fujian Nanan, 59131; Fujian Chongwu, 59133<br>3. Gansu Wushan, 57004; Gansu Huixian, 57110; Gansu Liangdang, 57111<br>4. Guizhou Daozhen, 57623; Guizhou Yanhe, 57636; Guizhou Dejiang, 57637; Guizhou Songtao, 57647; Guizhou Bijie, 57707; Guizhou Jinsha, 57714; Guizhou Zunyi, 57717; Guizhou Wengan, 57728; Guizhou Yuqing, 57729; Guizhou Sinan,

	57731; Guizhou Cengong, 57735; Guizhou Jiangkou, 57736; Guizhou Shibing, 57737; Guizhou Wanshan, 57742; Guizhou Xianxi, 57803; Guizhou Zhijin, 57805; Guizhou Anshun, 57806; Guizhou Liuzhi, 57807; Guizhou Xiuwen, 57811; Guizhou Pingba, 57814; Guizhou Huangping, 57822; Guizhou Kaili, 57825; Guizhou Duyun, 57827; Guizhou Sansui, 57832; Guizhou Liping, 57839; Guizhou Tianzhu, 57840; Guizhou Jinping, 57844; Guizhou Qinglong, 57900; Guizhou Guanling, 57903; Guizhou Zhenfeng, 57905; Guizhou Wangmo, 57906; Guizhou Huishui, 57912; Guizhou Longli, 57913; Guizhou Sandu, 57923<br>5. Henan Xinye, 57271; Henan Biyang, 57281; Henan Tongbai, 57285; Henan Jigongshan, 57390; Henan Xixian, 57396; Henan Shangcheng, 58301<br>6. Hubei Zhuxi, 57249; Hubei Yunxi, 57251; Hubei Yunxian, 57253; Hubei Shiyan, 57256; Hubei Zhushan, 57257; Hubei Fangxian, 57259; Hubei Danjiangkou, 57260; Hubei Gucheng, 57268; Hubei Xiangyang, 57278; Hubei Zaoyang, 57279; Hubei Xingshan, 57359; Hubei Baokang, 57361; Hubei Shennongjia, 57362; Hubei Nanzhang, 57363; Hubei Yicheng, 57370; Hubei Jingmen, 57377; Hubei Zhongxiang, 57378; Hubei Suizhou, 57381; Hubei Xiaochang, 57386; Hubei Jingshan, 57387; Hubei Anlu, 57388; Hubei Hongan, 57398; Hubei Macheng, 57399; Hubei Lichuan, 57439; Hubei Jianshi, 57445; Hubei Enshi, 57447; Hubei Yiling , 57453; Hubei Wufeng, 57458; Hubei Songzi, 57469; Hubei Qianjiang, 57475; Hubei Gongan, 57477; Hubei Yingcheng, 57481; Hubei Xiaogan, 57482; Hubei Tianmen, 57483; Hubei Shayang, 57484; Hubei Xiantao, 57485; Hubei Hanchuan, 57486; Hubei Caidian, 57489; Hubei Xinzhou, 57492; Hubei Wuhan, 57494; Hubei Tuanfeng, 57495; Hubei Ezhou, 57496; Hubei Xianfeng, 57540; Hubei Xuanen, 57541; Hubei Jiayu, 57583; Hubei Chongyang, 57586; Hubei Xianning, 57590; Hubei Luotian, 58401; Hubei Yingshan, 58402; Hubei Qichun, 58408; Hubei Huangmei, 58409; Hubei Yangxin, 58500<br>7. Hunan Fenghuang, 57740<br>8. Jiangsu Pizhou, 58026; Jiangsu Xuzhou, 58027; Jiangsu Donghai, 58036; Jiangsu Shuyang, 58038; Jiangsu Ganyu, 58040; Jiangsu Xiliandao, 58041; Jiangsu Lianyungang, 58044; Jiangsu Xiangshui, 58045; Jiangsu Guanyun, 58047; Jiangsu Siyang, 58132; Jiangsu Sihong, 58135; Jiangsu Hongze, 58139; Jiangsu Lianshui, 58140; Jiangsu Huaian, 58141; Jiangsu Funing, 58143; Jiangsu Jianhu, 58146; Jiangsu Jinhu, 58147; Jiangsu Baoying, 58148; Jiangsu Yancheng, 58154; Jiangsu Dafeng, 58158; Jiangsu Liuhe, 58235; Jiangsu Gaoyou, 58241; Jiangsu Yizheng, 58242; Jiangsu Xinghua, 58243; Jiangsu Taizhou, 58246; Jiangsu Yangzhong, 58247; Jiangsu Taixing, 58249; Jiangsu Jiangyan, 58250; Jiangsu Dongtai, 58251; Jiangsu Haian, 58254; Jiangsu Rugao, 58255; Jiangsu Jingjiang, 58257; Jiangsu Nantong, 58259; Jiangsu Rudong, 58264; Jiangsu Lvsi, 58265; Jiangsu Lishui, 58340; Jiangsu Danyang, 58341; Jiangsu Jintan, 58342; Jiangsu Jurong, 58344; Jiangsu Suzhou, 58349; Jiangsu Changshu, 58352; Jiangsu Zhangjiagang, 58353; Jiangsu Wuxi, 58354; Jiangsu Kunshan, 58356; Jiangsu Haimen, 58360; Jiangsu Taicang, 58377<br>9. Inner Mongolia Henan, 53732<br>10. Ningxia Yongning, 53618

	11. Shandong Yutai, 54907; Shandong Xuechen, 58021; Shandong Yicheng, 58022<br>12. Shanxi Shilou, 53759; Shanxi Yonghe, 53852; Shanxi Hejin, 53957; Shanxi Yongji, 57052; Shanxi Ruicheng, 57053<br>13. Shaanxi Fugu, 53567; Shaanxi Yulin, 53646; Shaanxi Shenmu, 53651; Shaanxi Dingbian, 53725; Shaanxi Jingbian, 53735; Shaanxi Wuqi, 53738; Shaanxi Hengshan, 53740; Shaanxi Zichang, 53748; Shaanxi Suide, 53754; Shaanxi Zhidan, 53832; Shaanxi Ansai, 53841; Shaanxi Ganquan, 53848; Shaanxi Yanchuan, 53850; Shaanxi Yanchang, 53854; Shaanxi Fuxian, 53931; Shaanxi Xunyi, 53938; Shaanxi Baishui, 53941; Shaanxi Huangling, 53944; Shaanxi Huanglong, 53946; Shaanxi Tongchuan, 53947; Shaanxi Pucheng, 53948; Shaanxi Longxian, 57003; Shaanxi Baoji, 57016; Shaanxi Qianyang, 57021; Shaanxi Linyou, 57022; Shaanxi Fufeng, 57026; Shaanxi Meixian, 57027; Shaanxi Liquan, 57029; Shaanxi Yongshou, 57030; Shaanxi Wugong, 57034; Shaanxi Yaoxian, 57037; Shaanxi Xingping, 57038; Shaanxi Sanyuan, 57041; Shaanxi Fuping, 57042; Shaanxi Dali, 57043; Shaanxi Weinan, 57045; Shaanxi Huashan, 57046; Shaanxi Lueyang, 57106; Shaanxi Fengxian, 57113; Shaanxi Mianxian, 57119; Shaanxi Liuba, 57124; Shaanxi Foping, 57134; Shaanxi Ningshan, 57137; Shaanxi Zhashui, 57140; Shaanxi Shangxian, 57143; Shaanxi Danfeng, 57153; Shaanxi Shangnan, 57154; Shaanxi Shanyang, 57155; Shaanxi Nanzheng, 57213; Shaanxi Ziyang, 57231; Shaanxi Hanyin, 57233; Shaanxi Zhenba, 57238; Shaanxi Ankang, 57245; Shaanxi Pingli, 57248; Shaanxi Baihe, 57254<br>14. Yunnan Zhenxiong, 56595<br>15. Zhejiang Changxing, 58443; Zhejiang Wencheng, 58750
Cluster C	1. Guangdong Lianshan, 59074; Guangdong Yunan, 59268; Guangdong Xinyi, 59456<br>2. Guangxi Ziyuan, 57859; Guangxi Tiane, 57927; Guangxi Sanjiang, 57941; Guangxi Longsheng, 57942; Guangxi Rongshui, 57948; Guangxi Yongfu, 57949; Guangxi Lingui, 57954; Guangxi Xingan, 57955; Guangxi Quanzhou, 57960; Guangxi Guanyang, 57964; Guangxi Xilin, 59004; Guangxi Leye, 59012; Guangxi Lingyun, 59015; Guangxi Tianlin, 59017; Guangxi Fengshan, 59021; Guangxi Hechi, 59023; Guangxi Bama, 59027; Guangxi Yizhou, 59034; Guangxi Duan, 59037; Guangxi Xincheng, 59038; Guangxi Liucheng, 59041; Guangxi Luzhai, 59045; Guangxi Pingle, 59053; Guangxi Lipu, 59055; Guangxi Jinxiu, 59057; Guangxi Mengshan, 59058; Guangxi Hezhou, 59065; Guangxi Baise, 59211; Guangxi Debao, 59215; Guangxi Jingxi, 59218; Guangxi Tiandong, 59224; Guangxi Tiandeng, 59227; Guangxi Longan, 59229; Guangxi Mashan, 59230; Guangxi Shanglin, 59235; Guangxi Wuming , 59237; Guangxi Binyang, 59238; Guangxi Xiangzhou, 59241; Guangxi Laibin, 59242; Guangxi Wuxuan, 59246; Guangxi Pingnan, 59255; Guangxi Tengxian, 59256; Guangxi Wuzhou, 59265; Guangxi Cangwu, 59266; Guangxi Daxin, 59421; Guangxi Shangsi, 59429; Guangxi Nanning, 59431; Guangxi Yongning, 59435; Guangxi Hengxian, 59441; Guangxi Lingshan, 59446; Guangxi Pubei, 59448; Guangxi Bobai, 59449; Guangxi Beiliu, 59451; Guangxi Rongxian, 59452; Guangxi Cenxi, 59454;

	Guangxi Luchuan, 59457; Guangxi Qinzhou, 59632; Guangxi Hepu, 59640<br>3. Guizhou Weining, 56691; Guizhou Panxian, 56793; Guizhou Xingyi, 57907; Guizhou Ceheng, 57909; Guizhou Pingtang, 57921; Guizhou Libo, 57926; Guizhou Congjiang, 57936<br>4. Hebei Xinglong, 54425; Hebei Yutian, 54522<br>5. Hunan Dongan, 57867<br>6. Shaanxi Jiaxian, 53658; Shaanxi Wubao, 53756; Shaanxi Qingjian, 53757; Shaanxi Yichuan, 53857; Shaanxi Chengcheng, 53949; Shaanxi Heyang, 53950; Shaanxi Luonan, 57057; Shaanxi Chenggu, 57128; Shaanxi Xunyang, 57242<br>7. Sichuan Derong, 56441; Sichuan Pingshan, 56494; Sichuan Gongxian, 56499; Sichuan Panzhihua, 56666<br>8. Tianjin Wuqing , 54523; Tianjin Baodi, 54525; Tianjin Tanggu, 54623<br>9. Yunnan Deqin, 56444; Yunnan Yanjin, 56497; Yunnan Xianggelila, 56543; Yunnan Weixi, 56548; Yunnan Ninglang, 56567; Yunnan Daguan, 56582; Yunnan Ludian, 56585; Yunnan Weixin, 56596; Yunnan Liuku, 56643; Yunnan Lanping, 56645; Yunnan Jianchuan, 56646; Yunnan Eryuan, 56649; Yunnan Lijiang, 56651; Yunnan Yongsheng, 56652; Yunnan Heqing, 56654; Yunnan Huaping, 56664; Yunnan Yongren, 56669; Yunnan Dongchuan, 56688; Yunnan Xuanwei, 56697; Yunnan Tengchong, 56739; Yunnan Yunlong, 56742; Yunnan Yangbi, 56745; Yunnan Yongping, 56746; Yunnan Baoshan, 56748; Yunnan Dali, 56751; Yunnan Binchuan, 56752; Yunnan Midu, 56755; Yunnan Weishan, 56757; Yunnan Yaoan, 56764; Yunnan Mouding, 56766; Yunnan Nanhua, 56767; Yunnan Fumin, 56772; Yunnan Wuding, 56774; Yunnan Lufeng, 56777; Yunnan Kunming, 56778; Yunnan Malong, 56782; Yunnan Qujing, 56783; Yunnan Songming, 56785; Yunnan Fuyuan, 56790; Yunnan Lianghe, 56840; Yunnan Longling, 56841; Yunnan Shidian, 56842; Yunnan Changning, 56843; Yunnan Fengqing, 56846; Yunnan Yongde, 56849; Yunnan Yunxian, 56854; Yunnan Jingdong, 56856; Yunnan Shuangbai, 56862; Yunnan Anning, 56863; Yunnan Zhenyuan, 56867; Yunnan Xinping, 56869; Yunnan Yimen, 56870; Yunnan Jinning, 56871; Yunnan Chengjiang, 56873; Yunnan Yuxi, 56875; Yunnan Huaning, 56879; Yunnan Yiliang, 56880; Yunnan Shilin, 56881; Yunnan Shizong, 56883; Yunnan Mile, 56885; Yunnan Luxi, 56886; Yunnan Qiubei, 56889; Yunnan Luoping, 56891; Yunnan Eshan, 56898; Yunnan Cangyuan, 56944; Yunnan Gengma, 56946; Yunnan Ximeng, 56948; Yunnan Menglian, 56949; Yunnan Shuangjiang, 56950; Yunnan Lincang, 56951; Yunnan Jinggu, 56952; Yunnan Lancang, 56954; Yunnan Menghai, 56958; Yunnan Mojiang, 56962; Yunnan Simao, 56964; Yunnan Yuanjiang, 56966; Yunnan Mengla, 56969; Yunnan Shiping, 56970; Yunnan Yuanyang, 56976; Yunnan Luchun, 56978; Yunnan Kaiyuan, 56982; Yunnan Gejiu, 56984; Yunnan Yanshan, 56991; Yunnan Xichou, 56992; Yunnan Wenshan, 56994; Yunnan Guangnan, 59007
Cluster D	1. Gansu Mazongshan, 52323; Gansu Subei, 52515; Gansu Jiuquan, 52533; Gansu Minle, 52656; Gansu Wuwei, 52679; Gansu Minqin, 52681; Gansu Gulang, 52784; Gansu Wushaoling, 52787; Gansu Tianzhu, 52881; Gansu Yongdeng, 52885; Gansu Yuzhong, 52983; Gansu Hezheng, 52985; Gansu Kangle, 52988;

	Gansu Huining, 52993; Gansu Anding, 52995; Gansu Huajialing, 52996; Gansu Huanxian, 53821; Gansu Lingtai, 53924; Gansu Zhenyuan, 53925; Gansu Jingchuan, 53926; Gansu Huating, 53927; Gansu Huachi, 53930; Gansu Huishui, 53934; Gansu Luqu, 56071; Gansu Lintan, 56081; Gansu Zhuoni, 56082; Gansu Zhangxian, 56091; Gansu Dangchang, 56095; Gansu Wudu, 56096; Gansu Gangu, 57001; Gansu Qingshui, 57011; Gansu Maiji, 57014; Gansu Chengxian, 57102; Gansu Kangxian, 57105<br>2. Heilongjiang Jiagedaqi, 50442<br>3. Jilin Shuangliao, 54142<br>4. Liaoning Changtu, 54243; Liaoning Kangping, 54244; Liaoning Shenbei, 54248; Liaoning Tieling, 54249; Liaoning Xifeng, 54252; Liaoning Qingyuan, 54259; Liaoning Chaoyang, 54324; Liaoning Yangshan, 54325; Liaoning Fushun, 54351; Liaoning Caohekou, 54483; Liaoning Xiuyan, 54486; Liaoning Kuandian, 54493; Liaoning Dandong, 54497; Liaoning Wafangdian, 54563; Liaoning Jinzhou, 54568; Liaoning Pikou, 54575; Liaoning Changhai, 54579; Liaoning Changxingdao, 54565<br>5. Inner Mongolia Tulihe, 50434; Inner Mongolia Elunchunqi, 50445; Inner Mongolia Manzhouli, 50514; Inner Mongolia Evenkeqi, 50525; Inner Mongolia Xinbaerhuyouqi, 50603; Inner Mongolia Xinbaerhuzuoqi, 50618; Inner Mongolia Zhalantun, 50639; Inner Mongolia Aershan, 50727; Inner Mongolia Wulagai, 50913; Inner Mongolia Houlinguole, 50924; Inner Mongolia Bayaertuhushuo, 50928; Inner Mongolia Erlianhaote, 53068; Inner Mongolia Narenbaolige, 53083; Inner Mongolia Mandula, 53149; Inner Mongolia Abagaqi, 53192; Inner Mongolia Hailisu, 53231; Inner Mongolia Xianghuangqi, 53289; Inner Mongolia Wulatezhongqi, 53336; Inner Mongolia Dashetai, 53348; Inner Mongolia Damaoqi, 53352; Inner Mongolia Xilamuren, 53367; Inner Mongolia Chayouzhongqi, 53378; Inner Mongolia Huade, 53391; Inner Mongolia Dengkou, 53419; Inner Mongolia Hangjinhouqi , 53420; Inner Mongolia Wulateqianqi, 53433; Inner Mongolia Baotou, 53446; Inner Mongolia Tuyouqi, 53455; Inner Mongolia Dalateqi, 53457; Inner Mongolia Huhehaote, 53463; Inner Mongolia Huhehaote suburb, 53466; Inner Mongolia Tuoketuoxian, 53467; Inner Mongolia Helingeerxian, 53469; Inner Mongolia Zhuozi, 53472; Inner Mongolia Liangcheng, 53475; Inner Mongolia Xinghe, 53483; Inner Mongolia Wuhai, 53512; Inner Mongolia Yikewusu, 53522; Inner Mongolia Etukeqi, 53529; Inner Mongolia Hangjinqi, 53533; Inner Mongolia Dongsheng, 53543; Inner Mongolia Ejinhuoluoqi, 53545; Inner Mongolia Zhungeerqi, 53553; Inner Mongolia Qingshuihexian, 53562; Inner Mongolia Wushenqi, 53644; Inner Mongolia Xiwuzhumuqin, 54012; Inner Mongolia Fuhe, 54024; Inner Mongolia Balinzuoqi, 54027; Inner Mongolia Xilinhaote, 54102; Inner Mongolia Balinyouqi, 54113; Inner Mongolia Linxixian, 54115; Inner Mongolia Keshiketengqi, 54117; Inner Mongolia Qinglongshan, 54132; Inner Mongolia Tongliao, 54135; Inner Mongolia Zhenglanqi, 54205; Inner Mongolia Gangzi, 54214; Inner Mongolia Chifeng, 54218; Inner Mongolia Aohanqi, 54225; Inner Mongolia Baoguotu, 54226; Inner Mongolia Kezuohouqi, 54231;

	Inner Mongolia Taibushiqi, 54305; Inner Mongolia Kalaqinqi, 54313; Inner Mongolia Ningchengxian, 54320<br>6. Ningxia Shitanjing, 53517; Ningxia Wuzhong, 53612; Ningxia Zhongning, 53705; Ningxia Xingren, 53707; Ningxia Yanchi, 53723; Ningxia Maihuangshan, 53727; Ningxia Haiyuan, 53806; Ningxia Guyuan, 53817; Ningxia Xiji, 53903; Ningxia Liupanshan, 53910<br>7. Qinghai Huzhu, 52863; Qinghai Xining, 52866; Qinghai Pingan, 52875; Qinghai Hualong, 52877; Qinghai Guinan, 52955<br>8. Xinjiang Buerjin, 51060; Xinjiang Fuyun, 51087; Xinjiang Emin, 51145; Xinjiang Hebukesaier, 51156; Xinjiang Bole, 51238; Xinjiang Touli, 51241; Xinjiang Kelamayi, 51243; Xinjiang Jinghe, 51334; Xinjiang Shawan, 51357; Xinjiang Changji, 51368; Xinjiang Miquan, 51369; Xinjiang Tianshandaxigou, 51468; Xinjiang Urumqi Pastoral Test Station, 51469; Xinjiang Tianchi, 51470; Xinjiang Dabancheng, 51477; Xinjiang Mulei, 51482; Xinjiang Kumishi, 51526; Xinjiang Tuokexun, 51571; Xinjiang Tulufandongkan, 51572; Xinjiang Tulufan, 51573; Xinjiang Xinhe, 51636; Xinjiang Shaya, 51639; Xinjiang Kuerle, 51656; Xinjiang Wuqia, 51705; Xinjiang Kashi, 51709; Xinjiang Yuepuhu, 51717; Xinjiang Alaer, 51730; Xinjiang Tazhong, 51747; Xinjiang Tieqianlike, 51765; Xinjiang Yecheng, 51814; Xinjiang Pishan, 51818; Xinjiang Moyu, 51827; Xinjiang Minfeng, 51839; Xinjiang Qiemuo, 51855; Xinjiang Yutian, 51931; Xinjiang Balikun, 52101; Xinjiang Naomaohu, 52112; Xinjiang Yiwu, 52118; Xinjiang Hami, 52203; Xinjiang Hongliuhe, 52313
Cluster E	1. Gansu Sunan, 52643<br>2. Hebei Weixian, 53593; Hebei Laiyuan, 53599; Hebei Shexian, 53886<br>3. Henan Linzhou, 53889<br>4. Heilongjiang Mohe, 50136; Heilongjiang Tahe, 50246; Heilongjiang Huzhong, 50247; Heilongjiang Xinlin, 50349; Heilongjiang Huma, 50353; Heilongjiang Sunwu, 50564; Heilongjiang Xunke, 50566; Heilongjiang Wuyiling, 50674; Heilongjiang Gannan, 50741; Heilongjiang Fuyu, 50742; Heilongjiang Lindian, 50749; Heilongjiang Baiquan, 50755; Heilongjiang Yichun, 50774; Heilongjiang Suibin, 50787; Heilongjiang Fujin, 50788; Heilongjiang Tailai, 50844; Heilongjiang Qinggang, 50851; Heilongjiang Beilin, 50853; Heilongjiang Anda, 50854; Heilongjiang Tieli, 50862; Heilongjiang Huanan, 50879; Heilongjiang Shuangyashan, 50884; Heilongjiang Baoqing, 50888; Heilongjiang Haerbin, 50953; Heilongjiang Mulan, 50962; Heilongjiang Tonghe, 50963; Heilongjiang Shangzhi, 50968<br>5. Jilin Baicheng, 50936; Jilin Taonan, 50939; Jilin Daan, 50945; Jilin Songyuan, 50946; Jilin Qianguo, 50949; Jilin Changling, 54049; Jilin Jiutai, 54069; Jilin Shulan, 54076; Jilin Gujiazi, 54155; Jilin Yitong, 54164; Jilin Yongji, 54171; Jilin Jiaohe, 54181; Jilin Dunhua, 54186; Jilin Antu, 54187; Jilin Luozigou, 54192; Jilin Wangqing, 54195; Jilin Liaoyuan, 54260; Jilin Jiangyuan, 54279; Jilin Erdao, 54285; Jilin Helong, 54286; Jilin Tonghua, 54363; Jilin Baishan, 54371<br>6. Qinghai Lenghu, 52602; Qinghai Nuomuhong, 52825; Qinghai Doulan, 52836; Qinghai Qinghaihu 151, 52854; Qinghai Gonghe, 52856; Qinghai Guide, 52868;

	Qinghai Minhe, 52876; Qinghai Wudaoliang, 52908; Qinghai Shazhuyu, 52941; Qinghai Xinghai, 52943; Qinghai Tongde, 52957; Qinghai Xunhua, 52972; Qinghai Tongren, 52974; Qinghai Tuotuohe, 56004; Qinghai Zhiduo, 56016; Qinghai Zaduo, 56018; Qinghai Qumacai, 56021; Qinghai Yushu, 56029; Qinghai Qingshuihe, 56034; Qinghai Maqin, 56043; Qinghai Gander, 56045; Qinghai Dari, 56046; Qinghai Henan, 56065; Qinghai Jiuzhi, 56067<br>7. Shanxi Youyu, 53478; Shanxi Yanggao, 53486; Shanxi Datong, 53487; Shanxi Pinglu, 53574; Shanxi Shanyin, 53576; Shanxi Ningwu, 53577; Shanxi Shuozhou, 53578; Shanxi Daixian, 53579; Shanxi Hunyuan, 53582; Shanxi Yingxian, 53584; Shanxi Fanshi, 53585; Shanxi Wutaishan, 53588; Shanxi Guangling, 53590; Shanxi Linxian, 53659; Shanxi Kelan, 53662; Shanxi Wuzhai, 53663; Shanxi Xingxian, 53664; Shanxi Lanxian, 53665; Shanxi Jingle, 53666; Shanxi Yuanping, 53673; Shanxi Xinfu, 53674; Shanxi Dingxiang, 53676; Shanxi Jiancaoping, 53677; Shanxi Xiaodian, 53679; Shanxi Wutaixian, 53681; Shanxi Yuxian, 53685; Shanxi Pingding, 53687; Shanxi Liulin, 53753; Shanxi Fangshan, 53760; Shanxi Gujiao, 53763; Shanxi Lishi, 53764; Shanxi Zhongyang, 53767; Shanxi Xiaoyi, 53768; Shanxi Qingxu, 53774; Shanxi Taigu, 53775; Shanxi Pingyao, 53778; Shanxi Shouyang, 53780; Shanxi Yangquan, 53782; Shanxi Zuoquan, 53786; Shanxi Yushe, 53787; Shanxi Heshun, 53788; Shanxi Xixian, 53853; Shanxi Jiaokou, 53860; Shanxi Xiangfen, 53861; Shanxi Lingshi, 53862; Shanxi Jiexiu, 53863; Shanxi Puxian, 53864; Shanxi Fenxi, 53865; Shanxi Hongtong, 53866; Shanxi Wuxiang, 53871; Shanxi Qinxian, 53872; Shanxi Changzi, 53873; Shanxi Guxian, 53874; Shanxi Qinyuan, 53875; Shanxi Anze, 53877; Shanxi Licheng, 53878; Shanxi Lucheng, 53880; Shanxi Xiangning, 53953; Shanxi Jishan, 53954; Shanxi Wanrong, 53956; Shanxi Yanhu, 53959; Shanxi Xinjiang, 53964; Shanxi Jiangxian, 53965; Shanxi Fushan, 53966; Shanxi Yuanqu, 53968; Shanxi Qingshui, 53970; Shanxi Gaoping, 53973
Cluster F	1. Gansu Zhouqu, 56094; Gansu Wenxian, 56192<br>2. Qinghai Banma, 56151<br>3. Shaanxi Ningqiang, 57211<br>4. Sichuan Shiqu, 56038; Sichuan Ruoergai, 56079; Sichuan Jiuzhaigou, 56097; Sichuan Ganzi, 56146; Sichuan Seda, 56152; Sichuan Luhuo, 56158; Sichuan Rangtang, 56164; Sichuan Daofu, 56167; Sichuan Jinchuan, 56168; Sichuan Aba, 56171; Sichuan Maerkang, 56172; Sichuan Hongyuan, 56173; Sichuan Xiaojin, 56178; Sichuan Maoxian, 56180; Sichuan Chongzhou, 56181; Sichuan Songpan, 56182; Sichuan Wenchuan, 56183; Sichuan Lixian, 56184; Sichuan Heishui, 56185; Sichuan Mianzhu, 56186; Sichuan Wenjiang, 56187; Sichuan Dujiangyan, 56188; Sichuan Pengzhou, 56189; Sichuan Deyang, 56198; Sichuan Batang, 56247; Sichuan Xinlong, 56251; Sichuan Litang, 56257; Sichuan Danba, 56263; Sichuan Yajiang, 56267; Sichuan Baoxing, 56273; Sichuan Lushan, 56279; Sichuan Mingshan, 56280; Sichuan Pujiang, 56281; Sichuan Longquanyi, 56286; Sichuan Pengshan, 56289; Sichuan Jintang, 56296; Sichuan Renshou, 56297; Sichuan Ziyang, 56298; Sichuan Daocheng, 56357; Sichuan Luding, 56371; Sichuan Yingjing, 56373; Sichuan Kangding, 56374; Sichuan Hanyuan,

	56376; Sichuan Shimian, 56378; Sichuan Hongya, 56380; Sichuan Jiajiang, 56382; Sichuan Qingshen, 56383; Sichuan Emeishan, 56385; Sichuan Ebian, 56387; Sichuan Qianwei, 56389; Sichuan Jingyan, 56390; Sichuan Zizhong, 56393; Sichuan Weiyuan, 56395; Sichuan Zigong, 56396; Sichuan Fushun, 56399; Sichuan Xiangcheng, 56443; Sichuan Muli, 56459; Sichuan Jiulong, 56462; Sichuan Ganluo, 56473; Sichuan Mianning, 56474; Sichuan Yuexi, 56475; Sichuan Xide, 56478; Sichuan Chaojue, 56479; Sichuan Mabian, 56480; Sichuan Leibo, 56485; Sichuan Meigu, 56487; Sichuan Muchuan, 56490; Sichuan Yibinxian, 56491; Sichuan Nanxi, 56493; Sichuan Xingwen, 56496; Sichuan Yanyuan, 56565; Sichuan Dechang, 56569; Sichuan Xichang, 56571; Sichuan Puge, 56575; Sichuan Butuo, 56580; Sichuan Jinyang, 56584; Sichuan Changning, 56593; Sichuan Yanbian, 56665; Sichuan Miyi, 56670; Sichuan Huili, 56671; Sichuan Huidong, 56675; Sichuan Qingchuan, 57204; Sichuan Guangyuan, 57206; Sichuan Jiange, 57208; Sichuan Nanjiang, 57216; Sichuan Wangcang, 57217; Sichuan Wanyuan, 57237; Sichuan Langzhong, 57306; Sichuan Xichong, 57309; Sichuan Bazhong, 57313; Sichuan Nanbu, 57314; Sichuan Yilong, 57315; Sichuan Yingshan, 57318; Sichuan Tongjiang, 57320; Sichuan Pingchang, 57324; Sichuan Dachuan, 57328; Sichuan Kaijiang, 57329; Sichuan Shehong, 57401; Sichuan Suining, 57405; Sichuan Gaoping, 57411; Sichuan Quxian, 57413; Sichuan Guangan, 57415; Sichuan Linshui, 57416; Sichuan Dazhu, 57420; Sichuan Dongxing, 57503; Sichuan Longchang, 57507; Sichuan Jiangan, 57600; Sichuan Hejiang, 57603; Sichuan Xuyong, 57608<br>5. Yunnan Suijiang, 56483; Yunnan Qiaojia, 56673<br>6. Chongqing Tongnan, 57409; Chongqing Dazu, 57502; Chongqing Rongchang, 57505; Chongqing Yongchuan, 57506
Cluster G	1. Gansu Xiahe, 52978; Gansu Yongjing, 52980; Gansu Guanghe, 52982; Gansu Lintao, 52986; Gansu Weiyuan, 52998; Gansu Jingning, 53906; Gansu Zhuanglang, 53917; Gansu Hezuo, 56080; Gansu Dibu, 56084; Gansu Longxi, 56092; Gansu Minxian, 56093; Gansu Lixian, 57007<br>2. Heilongjiang Aihui, 50468; Heilongjiang Nenjiang, 50557; Heilongjiang Nehe, 50646; Heilongjiang Beian, 50656; Heilongjiang Kedong, 50659; Heilongjiang Longjiang, 50739; Heilongjiang Qiqihaer, 50745; Heilongjiang Yian, 50750; Heilongjiang Hailun, 50756; Heilongjiang Minshui, 50758; Heilongjiang Suileng, 50767; Heilongjiang Wuying, 50772; Heilongjiang Hegang, 50775; Heilongjiang Luobei, 50776; Heilongjiang Tongjiang, 50778; Heilongjiang Fuyuan, 50779; Heilongjiang Dumeng, 50842; Heilongjiang Wangkui, 50852; Heilongjiang Zhaodong, 50858; Heilongjiang Lansi, 50859; Heilongjiang Qingan, 50861; Heilongjiang Tangyuan, 50871; Heilongjiang Jiamusi, 50873; Heilongjiang Yilang, 50877; Heilongjiang Huachuan, 50878; Heilongjiang Hulan, 50956; Heilongjiang Acheng, 50958; Heilongjiang Binxian, 50960; Heilongjiang Zhengfang, 50964; Heilongjiang Yanshou, 50965; Heilongjiang Qitaihe, 50971; Heilongjiang Boli, 50973; Heilongjiang Jixi, 50978; Heilongjiang Linkou, 50979; Heilongjiang Jidong, 50987; Heilongjiang Wuchang, 54080; Heilongjiang Hailin, 54092; Heilongjiang Muling, 54093; Heilongjiang Mudanjiang, 54094;

	Heilongjiang Suifenhe, 54096; Heilongjiang Ningan, 54098<br>3. Jilin Lishu, 54154; Jilin Yanji, 54292; Jilin Tonghuaxian, 54362<br>4. Liaoning Zhangwu, 54236; Liaoning Jianpingxian, 54326; Liaoning Lingyuan, 54327; Liaoning Liaozhong, 54332; Liaoning Xinmin, 54333; Liaoning Taian, 54336; Liaoning Panshan, 54338; Liaoning Anshan, 54339; Liaoning Sujiatun, 54340; Liaoning Shenyang, 54342; Liaoning Liaoyangxian, 54345; Liaoning Benxi, 54346; Liaoning Xinbin, 54353; Liaoning Lianshan, 54453; Liaoning Suizhong, 54454; Liaoning Xingcheng, 54455; Liaoning Yingkou, 54471; Liaoning Gaizhou, 54474; Liaoning Dashiqiao, 54475; Liaoning Fengcheng, 54494; Liaoning Pulandian, 54569; Liaoning Zhuanghe, 54584<br>5. Inner Mongolia Eerguna, 50425; Inner Mongolia Moulidawawoer, 50645; Inner Mongolia Arunqi, 50647; Inner Mongolia Suolun, 50834; Inner Mongolia Tuquan, 50934; Inner Mongolia Jining, 53480; Inner Mongolia Shebotu, 54039; Inner Mongolia Kailu, 54134<br>6. Xinjiang Akedala, 51058; Xinjiang Fuhai, 51068; Xinjiang Aletai, 51076; Xinjiang Hutubi, 51367; Xinjiang Fukang, 51377; Xinjiang Jimusaer, 51378; Xinjiang Bayinbuluke, 51542; Xinjiang Wushi, 51627; Xinjiang Akesu, 51628; Xinjiang Atushi, 51704; Xinjiang Kepin, 51720; Xinjiang Awati, 51722; Xinjiang Yengjisha, 51802; Xinjiang Shashe, 51811; Xinjiang Cele, 51826; Xinjiang Luopu, 51829
Cluster H	1. Anhui Dangshan, 58015; Anhui Xiaoxian, 58016; Anhui Bozhou, 58102; Anhui Jieshou, 58108; Anhui Funan, 58202<br>2. Gansu Gaitai, 52546; Gansu Linze, 52557; Gansu Yongchang, 52674; Gansu Jingtai, 52797; Gansu Gaolan, 52884; Gansu Jingyuan, 52895; Gansu Baiyin, 52896; Gansu Dongxiang, 52981; Gansu Qingcheng, 53829; Gansu Xifeng, 53923; Gansu Zhengning, 53935; Gansu Maqu, 56074; Gansu Tianshui, 57006; Gansu Zhangjiachuan, 57012<br>3. Hebei Linzhang, 53773; Hebei Fengfeng, 53894; Hebei Weixian, 53896; Hebei Daming, 54804<br>4. Henan Qinyang, 53972; Henan Qixian, 53974; Henan Jiyuan, 53978; Henan Jiaozuo, 53982; Henan Fengqiu, 53983; Henan Xiuwu, 53984; Henan Huixian, 53985; Henan Xinxiang, 53986; Henan Tangyin, 53991; Henan Junxian, 53992; Henan Neihuang, 53993; Henan Changheng, 53998; Henan Qingfeng, 54902; Henan Fanxian, 54903; Henan Sanmenxia, 57051; Henan Lingbao, 57056; Henan Mianchi, 57063; Henan Luoning, 57066; Henan Xinan, 57070; Henan Mengjin, 57071; Henan Mengzhou, 57072; Henan Yichuan, 57074; Henan Ruzhou, 57075; Henan Ruyang, 57078; Henan Wenxian, 57079; Henan Gongyi, 57080; Henan Xingyang, 57081; Henan Dengfeng, 57082; Henan Changge, 57087; Henan Xuchang, 57089; Henan Zhongmou, 57090; Henan Kaifeng, 57091; Henan Lankao, 57093; Henan Yanling, 57095; Henan Qixian, 57096; Henan Taikang, 57099; Henan Xixia, 57156; Henan Neixiang, 57169; Henan Lushan, 57173; Henan Zhenping, 57175; Henan Nanzhao, 57176; Henan Wugang, 57177; Henan Nanyang, 57178; Henan Fangcheng, 57179; Henan Jiaxian, 57180; Henan Xiangcheng, 57182; Henan Linying, 57183; Henan Yexian, 57184; Henan Wuyang, 57185; Henan Luohe, 57186; Henan Sheqi, 57187;

	Henan Xiping, 57188; Henan Suiping, 57189; Henan Huaiyang, 57192; Henan Xihua, 57193; Henan Runan, 57197; Henan Tanghe, 57273; Henan Zhumadian, 57290; Henan Pingyu, 57292; Henan Xincai, 57293; Henan Zhengyang, 57295; Henan Xixian, 57296; Henan Xinyang, 57297; Henan Guangshan, 57299; Henan Minquan, 58004; Henan Shangqiu , 58005; Henan Yucheng, 58006; Henan Echeng, 58007; Henan Xiayi, 58017; Henan Dancheng, 58100; Henan Huangchuan, 58207; Henan Gushi, 58208<br>5. Shandong Xinxian, 54808; Shandong Caoxian, 58002<br>6. Shanxi Lingchuan, 53981
Cluster I	1. Beijing Yanqing, 54406; Beijing Zhaitang, 54501; Beijing, 54511; Beijing Xiayunling, 54597<br>2. Hebei Kangbao, 53392; Hebei Zhangbei, 53399; Hebei Yangyuan, 53492; Hebei Xuanhua, 53498; Hebei Wanquan, 53499; Hebei Shunping, 53596; Hebei Lingshou, 53680; Hebei Quyang, 53682; Hebei Xingtang, 53688; Hebei Jinzhou, 53689; Hebei Fuping, 53690; Hebei Tangxian, 53692; Hebei Dingzhou, 53696; Hebei Wuji, 53699; Hebei Shahe, 53781; Hebei Baixiang, 53785; Hebei Luancheng, 53789; Hebei Longyao, 53794; Hebei Zanhuang, 53795; Hebei Ningjin, 53796; Hebei Julu, 53799; Hebei Wuan, 53890; Hebei Handan, 53892; Hebei Quzhou, 53893; Hebei Guyuan, 54301; Hebei Chongli, 54304; Hebei Fengning, 54308; Hebei Weichang, 54311; Hebei Longhua, 54318; Hebei Pingquan, 54319; Hebei Zhangjiakou, 54401; Hebei Chicheng, 54404; Hebei Huailai, 54405; Hebei Zhulu, 54408; Hebei Chengde, 54423; Hebei Zunhua, 54429; Hebei Chengdexian, 54430; Hebei Kuancheng, 54432; Hebei Qianxi, 54434; Hebei Qinglong, 54436; Hebei Luannan, 54437; Hebei Lulong, 54438; Hebei Qianan, 54439; Hebei Qinhuangdao, 54449; Hebei Zhuozhou, 54502; Hebei Rongcheng, 54503; Hebei Gaobeidian, 54506; Hebei Dachang, 54510; Hebei Guan, 54512; Hebei Yongqing, 54519; Hebei Sanhe, 54520; Hebei Xianghe, 54521; Hebei Tangshan, 54534; Hebei Caofeidian, 54535; Hebei Leting, 54539; Hebei Changli, 54540; Hebei Funing, 54541; Hebei Xushui, 54601; Hebei Goyang, 54603; Hebei Anxin, 54605; Hebei Raoyang, 54606; Hebei Shenzhou, 54608; Hebei Renqiu, 54610; Hebei Wenan, 54612; Hebei Dacheng, 54613; Hebei Hejian, 54614; Hebei Qingxian, 54615; Hebei Cangzhou, 54616; Hebei Botou, 54618; Hebei Huanghua, 54624; Hebei Haixing, 54628; Hebei Guangzong, 54631; Hebei Xinhe, 54633; Hebei Jize, 54640; Hebei Wuqiang, 54700; Hebei Xinji, 54701; Hebei Hengshui, 54702; Hebei Wuyi, 54703; Hebei Qinghe, 54706; Hebei Gucheng, 54707; Hebei Fucheng, 54710; Hebei Dongguang, 54713; Hebei-Weixian, 54800; Hebei Linxi, 54801; Hebei Guantao, 54809<br>3. Liaoning Jianchang, 54452<br>4. Inner Mongolia Balihan, 54316<br>5. Tianjin Jinghai, 54619; Tianjin Jinnan, 54622
Cluster J	1. Hebei Jingxian, 54711<br>2. Henan Taiqian, 54817; Henan Puyang, 54900<br>3. Jilin Yantongshan, 54169

	4. Shandong Wucheng, 54709; Shandong Linyi, 54712; Shandong Ningjin, 54716; Shandong Yangxin, 54723; Shandong Shanghe, 54724; Shandong Leling, 54726; Shandong Zhangqiu, 54727; Shandong Gaoqing, 54729; Shandong Binzhou, 54734; Shandong Kenli, 54744; Shandong Laizhou, 54749; Shandong Longdao, 54751; Shandong Penglai, 54752; Shandong Longkou, 54753; Shandong Zhaoyuan, 54755; Shandong Qixia, 54759; Shandong Fushan, 54764; Shandong Yantai, 54765; Shandong Chengshantou, 54776; Shandong Wendeng, 54777; Shandong Linqing, 54802; Shandong Liaocheng, 54806; Shandong Qihe, 54812; Shandong Chiping, 54814; Shandong Donge, 54815; Shandong Feicheng, 54819; Shandong Jiyang, 54821; Shandong Zouping, 54822; Shandong Jinan, 54823; Shandong Taian, 54827; Shandong Laiwu, 54828; Shandong Zibo, 54830; Shandong Qingzhou, 54831; Shandong Shouguang, 54832; Shandong Huantai, 54833; Shandong Yiyuan, 54836; Shandong Changyi, 54841; Shandong Pingdu, 54842; Shandong Weifang, 54843; Shandong Anqiu, 54844; Shandong Gaomi, 54846; Shandong Zhucheng, 54848; Shandong Jiaozhou, 54849; Shandong Laiyang, 54852; Shandong Jimo, 54855; Shandong Rushan, 54861; Shandong Juancheng, 54904; Shandong Yuncheng, 54905; Shandong Heze, 54906; Shandong Dingtao, 54909; Shandong Liangshan, 54910; Shandong Dongping, 54911; Shandong Wenshang, 54912; Shandong Juye, 54914; Shandong Yanzhou, 54916; Shandong Zoucheng, 54919; Shandong Sishui, 54920; Shandong Xintai, 54922; Shandong Mengyin, 54923; Shandong Pingyi, 54925; Shandong Tengzhou, 54927; Shandong Feixian, 54929; Shandong Yishui, 54932; Shandong Linyi, 54938; Shandong Junan, 54939; Shandong Wulian, 54940; Shandong Huangdao, 54943; Shandong Rizhao, 54945; Shandong Chengwu, 58003; Shandong Zaozhuang, 58024; Shandong Cangshan, 58030; Shandong Linshu, 58032<br>5. Shanxi Hequ, 53564; Shanxi Pianguan, 53565; Shanxi Jixian, 53859
Cluster K	1. Fujian Shaowu, 58725; Fujian Jianyang, 58734; Fujian Fuding, 58754; Fujian Taining, 58820; Fujian Shunchang, 58823; Fujian Mingxi, 58824; Fujian Sanming, 58828; Fujian Shuqing, 58839; Fujian Xiapu, 58843; Fujian Ningde, 58846; Fujian Changting, 58911; Fujian Yongtai, 58932; Fujian Nanjing, 59124; Fujian Pinghe, 59125<br>2. Shanghai Jinshan, 58460<br>3. Shanghai Jinshan, 58460<br>4. Zhejiang Anji, 58446; Zhejiang Linan, 58448; Zhejiang Fuyang, 58449; Zhejiang Huzhou, 58450; Zhejiang Jiashan, 58451; Zhejiang Jiaxing, 58452; Zhejiang Shaoxing, 58453; Zhejiang Deqing, 58454; Zhejiang Hangzhou, 58457; Zhejiang Cixi, 58467; Zhejiang Shengsi, 58472; Zhejiang Daishan, 58484; Zhejiang Tonglu, 58542; Zhejiang Jiande, 58544; Zhejiang Pujiang, 58546; Zhejiang Longyou, 58547; Zhejiang Jinhua, 58549; Zhejiang Zhuji, 58550; Zhejiang Xinchang, 58555; Zhejiang Shengzhou, 58556; Zhejiang Dongyang, 58558; Zhejiang Tiantai, 58559; Zhejiang Panan, 58560; Zhejiang Zhenhai, 58561; Zhejiang Yinzhou, 58562; Zhejiang Fenghua, 58565; Zhejiang Xiangshan, 58566; Zhejiang Ninghai, 58567; Zhejiang Sanmen, 58568; Zhejiang Shipu, 58569; Zhejiang

	Putuo, 58570; Zhejiang Wuyi, 58642; Zhejiang Yongkang, 58643; Zhejiang Suichang, 58644; Zhejiang Lishui, 58646; Zhejiang Longquan, 58647; Zhejiang Xianju, 58652; Zhejiang Jinyun, 58654; Zhejiang Leqing, 58656; Zhejiang Qingtian, 58657; Zhejiang Yongjia, 58658; Zhejiang Linhai, 58660; Zhejiang Wenling, 58664; Zhejiang Hongjia, 58665; Zhejiang Dachen, 58666; Zhejiang Yunhe, 58742; Zhejiang Taishun, 58746; Zhejiang Pingyang, 58751; Zhejiang Jingning, 58648
Cluster L	1. Hebei Luanping, 54420<br>2. Heilongjiang Wudalianchi, 50655; Heilongjiang Daqing, 50850; Heilongjiang Mishan, 50985<br>3. Jilin Liuhe, 54267<br>4. Ningxia Tongxin, 53810; Ningxia Weizhou, 53881<br>5. Sichuan Dege, 56144; Sichuan Baiyu, 56147<br>6. Tianjin Dagang, 54645<br>7. Tibet Shiquanhe, 55228; Tibet Gaize, 55248; Tibet Naqu, 55299; Tibet Pulan, 55437; Tibet Dangxiong, 55493; Tibet Lazi, 55569; Tibet Nanmulin, 55572; Tibet Muozhugongka, 55593; Tibet Zedang, 55598; Tibet Dingri, 55664; Tibet Jiangzi, 55680; Tibet Dingqing, 56116; Tibet Leiwuqi, 56128; Tibet Changdu, 56137; Tibet Luolong, 56223; Tibet Bomi, 56227; Tibet Basu, 56228; Tibet Linzhi, 56312; Tibet Miling, 56317; Tibet Zuogong, 56331; Tibet Chayu, 56434<br>8. Xinjiang Habahe, 51053; Xinjiang Tacheng, 51133
Cluster M	1. Hebei Shangyi, 53397<br>2. Liaoning Jianpingzhen, 54321<br>3. Inner Mongolia Yakeshi, 50526; Inner Mongolia Xiaoergou, 50548; Inner Mongolia Wuzhumuqindong, 50915; Inner Mongolia Wuyuan, 53337; Inner Mongolia Guyangxian, 53357; Inner Mongolia Siziwang, 53362; Inner Mongolia Wuchuanxian, 53368; Inner Mongolia Chayouhouqi, 53384; Inner Mongolia Shangdu, 53385; Inner Mongolia Tumutezuoqi, 53464; Inner Mongolia Chayouqianqi, 53481; Inner Mongolia Linhe, 53513; Inner Mongolia Eduokeqianqi, 53730; Inner Mongolia Zhalute, 54026; Inner Mongolia Gaoliban, 54031; Inner Mongolia Kezuozhongqi, 54047; Inner Mongolia Alukeerqinqi, 54122; Inner Mongolia Zhengxiangbaiqi, 54204; Inner Mongolia Duolunxian, 54208; Inner Mongolia Wengniuteqi, 54213; Inner Mongolia Neiman, 54223; Inner Mongolia Kulun, 54234<br>4. Xinjiang Manasi, 51359; Xinjiang Yanqi, 51567; Xinjiang Jiashi, 51707; Xinjiang Maigaiti, 51810; Xinjiang Zepu, 51815; Xinjiang Hetan, 51828
Cluster N	1. Heilongjiang Zhaozhou, 50950<br>2. Jilin Zhenlai, 50940; Jilin Qianan, 50948; Jilin Tongyu, 54041; Jilin Fuyu, 54063; Jilin Nongan, 54064; Jilin Dehui, 54065; Jilin Yushu, 54072; Jilin Changchun, 54161; Jilin Shuangyang, 54165; Jilin Jilin Suburb, 54172; Jilin Tongfeng, 54261; Jilin Panshi, 54263; Jilin Huadian, 54273; Jilin Huinan, 54274; Jilin Donggang, 54284; Jilin Jian, 54377

Table A2. SO_2 emissions from industrial sector in 2010

Cluster A	1. Anhui Dangshan, 58015; Anhui Xiaoxian, 58016; Anhui Bozhou, 58102; Anhui Jieshou, 58108; Anhui Funan, 58202; Anhui Fuyang, 58203; Anhui Qimen, 58520; Anhui Yixian, 58523; Anhui Tunxi, 58531<br>2. Fujian Guangze, 58724; Fujian Shaowu, 58725; Fujian Wuyishan, 58730; Fujian Pucheng, 58731; Fujian Jianyang, 58734; Fujian Songxi, 58735; Fujian Zhenghe, 58736; Fujian Shouning, 58744; Fujian Zhouning, 58747; Fujian Fuan, 58748; Fujian Zherong, 58749; Fujian Fuding, 58754; Fujian Ninghuai, 58818; Fujian Taining, 58820; Fujian Jianning, 58822; Fujian Shunchang, 58823; Fujian Mingxi, 58824; Fujian Sanming, 58828; Fujian Gutian, 58836; Fujian Youxi, 58837; Fujian Shuqing, 58839; Fujian Xiapu, 58843; Fujian Minhou, 58844; Fujian Ningde, 58846; Fujian Lianjiang, 58848; Fujian Changting, 58911; Fujian Wuping, 58917; Fujian Datian, 58923; Fujian Zhangping, 58926; Fujian Huaan, 58928; Fujian Anxi, 58929; Fujian Jiuxianshan, 58931; Fujian Yongtai, 58932; Fujian Xiuyu, 58938; Fujian Changle, 58941; Fujian Fuqing, 58942; Fujian Pingtan, 58944; Fujian Putian, 58946; Fujian Yongding, 59113; Fujian Changtai, 59122; Fujian Nanjing, 59124; Fujian Pinghe, 59125; Fujian Zhangpu, 59129; Fujian Tongan, 59130; Fujian Nanan, 59131; Fujian Chongwu, 59133; Fujian Zhaoan, 59320; Fujian Yunxiao, 59322<br>3. Gansu Gaitai, 52546; Gansu Linze, 52557; Gansu Yongchang, 52674; Gansu Jingtai, 52797; Gansu Gaolan, 52884; Gansu Jingyuan, 52895; Gansu Baiyin, 52896; Gansu Qingcheng, 53829; Gansu Xifeng, 53923; Gansu Zhengning, 53935; Gansu Maqu, 56074; Gansu Qinan, 57002; Gansu Tianshui, 57006; Gansu Zhangjiachuan, 57012<br>4. Guangdong Lechang, 57988; Guangdong Renhua, 57989; Guangdong Nanxiong, 57996; Guangdong Ruyuan, 59081; Guangdong Shaoguan, 59082; Guangdong Fogang, 59087; Guangdong Wengyuan, 59094; Guangdong Lianping, 59096; Guangdong Heping, 59099; Guangdong Longchuan, 59107; Guangdong Xingning, 59109; Guangdong Jiaoling, 59114; Guangdong Meixian, 59117; Guangdong Huaiji, 59270; Guangdong Guangning, 59271; Guangdong Sihui, 59276; Guangdong Sanshui, 59279; Guangdong Qingyuan, 59280; Guangdong Huadu, 59284; Guangdong Conghua, 59285; Guangdong Guangzhou, 59287; Guangdong Dongguan, 59289; Guangdong Heyuan, 59293; Guangdong Boluo, 59297; Guangdong Jiexi, 59306; Guangdong Fengshun, 59310; Guangdong Chaozhou, 59312; Guangdong Raoping, 59313; Guangdong Puning, 59314; Guangdong Shantou, 59316; Guangdong Yunfu, 59471; Guangdong Heshan, 59473; Guangdong Kaiping, 59475; Guangdong Xinhui, 59476; Guangdong Panyu, 59481; Guangdong Doumen, 59487; Guangdong Zhuhai, 59488; Guangdong Huidong, 59492; Guangdong Shenzhen, 59493; Guangdong Lufeng, 59502; Guangdong Suixi, 59650; Guangdong Gaozhou, 59653; Guangdong Lianjiang, 59654; Guangdong Huazhou, 59655; Guangdong Wuchuan, 59656; Guangdong Maoming, 59659; Guangdong Leizhou, 59750<br>5. Hebei Linzhang, 53773; Hebei Shexian, 53886; Hebei Fengfeng, 53894; Hebei

	Weixian, 53896; Hebei Daming, 54804<br>6. Henan Qinyang, 53972; Henan Qixian, 53974; Henan Jiyuan, 53978; Henan Jiaozuo, 53982; Henan Fengqiu, 53983; Henan Xiuwu, 53984; Henan Huixian, 53985; Henan Xinxiang, 53986; Henan Tangyin, 53991; Henan Junxian, 53992; Henan Neihuang, 53993; Henan Changheng, 53998; Henan Qingfeng, 54902; Henan Fanxian, 54903; Henan Sanmenxia, 57051; Henan Lingbao, 57056; Henan Mianchi, 57063; Henan Luoning, 57066; Henan Xinan, 57070; Henan Mengjin, 57071; Henan Mengzhou, 57072; Henan Yichuan, 57074; Henan Ruzhou, 57075; Henan Ruyang, 57078; Henan Wenxian, 57079; Henan Gongyi, 57080; Henan Xingyang, 57081; Henan Dengfeng, 57082; Henan Changge, 57087; Henan Xuchang, 57089; Henan Zhongmou, 57090; Henan Kaifeng, 57091; Henan Lankao, 57093; Henan Yanling, 57095; Henan Qixian, 57096; Henan Taikang, 57099; Henan Xixia, 57156; Henan Neixiang, 57169; Henan Lushan, 57173; Henan Zhenping, 57175; Henan Nanzhao, 57176; Henan Wugang, 57177; Henan Nanyang, 57178; Henan Fangcheng, 57179; Henan Jiaxian, 57180; Henan Xiangcheng, 57182; Henan Linying, 57183; Henan Yexian, 57184; Henan Wuyang, 57185; Henan Luohe, 57186; Henan Sheqi, 57187; Henan Xiping, 57188; Henan Suiping, 57189; Henan Huaiyang, 57192; Henan Xihua, 57193; Henan Runan, 57197; Henan Tanghe, 57273; Henan Zhumadian, 57290; Henan Pingyu, 57292; Henan Xincai, 57293; Henan Zhengyang, 57295; Henan Xixian, 57296; Henan Xinyang, 57297; Henan Guangshan, 57299; Henan Minquan, 58004; Henan Shangqiu , 58005; Henan Yucheng, 58006; Henan Echeng, 58007; Henan Xiayi, 58017; Henan Dancheng, 58100; Henan Huangchuan, 58207; Henan Gushi, 58208<br>7. Hubei Hefeng, 57543; Hubei Shishou, 57571; Hubei Jianli, 57573; Hubei Honghu, 57581; Hubei Chibi, 57582; Hubei Tongcheng, 57589; Hubei Wuxue, 58501<br>8. Hunan Longshan, 57544; Hunan Sangzhi, 57554; Hunan Zhangjiajie, 57558; Hunan Lixian, 57565; Hunan Nanxian, 57574; Hunan Huarong, 57575; Hunan Yueyang, 57584; Hunan Yongshun, 57643; Hunan Guzhang, 57646; Hunan Jishou, 57649; Hunan Yuanling, 57655; Hunan Luxi, 57657; Hunan Taoyuan, 57661; Hunan Changde, 57662; Hunan Hanshou, 57663; Hunan Taojiang, 57666; Hunan Anhua, 57669; Hunan Yuanjiang, 57671; Hunan Xiangyin, 57673; Hunan Ningxiang, 57678; Hunan Milo, 57680; Hunan Pingjiang, 57682; Hunan Changsha, 57687; Hunan Liuyang, 57688; Hunan Xupu, 57752; Hunan Hongjiang, 57754; Hunan Lengshuijiang, 57760; Hunan Xinhua, 57761; Hunan Loudi, 57763; Hunan Shaoshan, 57771; Hunan Xiangxiang, 57772; Hunan Shuangfeng, 57774; Hunan Nanyue, 57776; Hunan Hengshan, 57777; Hunan Youxian, 57779; Hunan Zhuzhou, 57780; Hunan Liling, 57781; Hunan Lengshuitan, 57865; Hunan Yongzhou, 57866; Hunan Qiyang, 57868; Hunan Qidong, 57870; Hunan Hengyangxian, 57871; Hunan Hengyang, 57872; Hunan Changning, 57874; Hunan Hengnan, 57875; Hunan Anren, 57881; Hunan Chaling, 57882; Hunan Yongxing, 57887; Hunan Guangxi, 57889; Hunan Daoxian, 57965; Hunan Ningyuan, 57966; Hunan Jiangyong, 57969; Hunan

Xintian, 57971; Hunan Chenzhou, 57972; Hunan Guiyang, 57973; Hunan Jiahe, 57974; Hunan Lanshan, 57975; Hunan Yizhang, 57976; Hunan Linwu, 57978; Hunan Zixing, 57981; Hunan Jianghua, 59063

9. Jiangsu Suyu, 58131
10. Jiangxi Xiushui, 57598; Jiangxi Tonggu, 57694; Jiangxi Wanzai, 57698; Jiangxi Shanggao, 57699; Jiangxi Pingxiang, 57786; Jiangxi Lianhua, 57789; Jiangxi Fenyi, 57792; Jiangxi Yichun, 57793; Jiangxi Xinyu, 57796; Jiangxi Anfu, 57798; Jiangxi Jianxian, 57799; Jiangxi Xiaping, 57883; Jiangxi Yongxin, 57891; Jiangxi Wanan, 57895; Jiangxi Suichuan, 57896; Jiangxi Taihe, 57899; Jiangxi Chongyi, 57990; Jiangxi Nankang, 57992; Jiangxi Ganxian, 57993; Jiangxi Xinfeng, 57995; Jiangxi Jiujiang, 58502; Jiangxi Ruichang, 58503; Jiangxi Lushan, 58506; Jiangxi Wuning, 58507; Jiangxi Dean, 58508; Jiangxi Hukou, 58510; Jiangxi Pengze, 58512; Jiangxi Duchang, 58517; Jiangxi Poyang, 58519; Jiangxi Jingdezhen, 58527; Jiangxi Wuyuan, 58529; Jiangxi Jingan, 58600; Jiangxi Fengxin, 58601; Jiangxi Anyi, 58602; Jiangxi Gaoan, 58605; Jiangxi Nanchang, 58606; Jiangxi Yugan, 58612; Jiangxi Jinxian, 58614; Jiangxi Wannian, 58615; Jiangxi Dongxiang, 58618; Jiangxi Linchuan, 58619; Jiangxi Dexing, 58622; Jiangxi Shangraoxian, 58623; Jiangxi Guixi, 58626; Jiangxi Qianshan, 58629; Jiangxi Yushan, 58634; Jiangxi Xinjian, 58693; Jiangxi Xiajiang, 58704; Jiangxi Yongfeng, 58705; Jiangxi Lean, 58706; Jiangxi Chongren, 58710; Jiangxi Jinxi, 58712; Jiangxi Nancheng, 58715; Jiangxi Nanfeng , 58718; Jiangxi Lichuan, 58719; Jiangxi Xingguo, 58804; Jiangxi Ningdu, 58806; Jiangxi Guangchang, 58813; Jiangxi Shicheng, 58814; Jiangxi Yudu, 58905; Jiangxi Huichang, 58906; Jiangxi Anyuan, 58907; Jiangxi Quannan, 59091; Jiangxi Longnan, 59092; Jiangxi Dingnan, 59093; Jiangxi Shangli, 57783
11. Inner Mongolia Damaoqi, 53352; Inner Mongolia Huhehaote, 53463; Inner Mongolia Tumutezuoqi, 53464
12. Ningxia Huinong, 53519; Ningxia Pingluo, 53611; Ningxia Taole, 53615; Ningxia Yongning, 53618
13. Shandong Xinxian, 54808; Shandong Caoxian, 58002
14. Shanxi Hequ, 53564; Shanxi Pianguan, 53565; Shanxi Pinglu, 53574; Shanxi Shanyin, 53576; Shanxi Ningwu, 53577; Shanxi Daixian, 53579; Shanxi Yingxian, 53584; Shanxi Fanshi, 53585; Shanxi Linxian, 53659; Shanxi Kelan, 53662; Shanxi Wuzhai, 53663; Shanxi Xingxian, 53664; Shanxi Lanxian, 53665; Shanxi Jingle, 53666; Shanxi Yuanping, 53673; Shanxi Xinfu, 53674; Shanxi Jiancaoping, 53677; Shanxi Wutaixian, 53681; Shanxi Yuxian, 53685; Shanxi Pingding, 53687; Shanxi Liulin, 53753; Shanxi Gujiao, 53763; Shanxi Lishi, 53764; Shanxi Zhongyang, 53767; Shanxi Xiaoyi, 53768; Shanxi Taigu, 53775; Shanxi Pingyao, 53778; Shanxi Shouyang, 53780; Shanxi Yangquan, 53782; Shanxi Zuoquan, 53786; Shanxi Yushe, 53787; Shanxi Xixian, 53853; Shanxi Jiaokou, 53860; Shanxi Xiangfen, 53861; Shanxi Lingshi, 53862; Shanxi Jiexiu, 53863; Shanxi Puxian, 53864; Shanxi Fenxi, 53865; Shanxi Wuxiang, 53871; Shanxi Qinxian, 53872; Shanxi Changzi, 53873; Shanxi Qinyuan, 53875; Shanxi Anze, 53877; Shanxi Licheng, 53878; Shanxi Xiangning, 53953; Shanxi Xinjiang, 53964; Shanxi

	Jiangxian, 53965; Shanxi Qingshui, 53970; Shanxi Gaoping, 53973; Shanxi Lingchuan, 53981<br>15. Shanghai Jinshan, 58460<br>16. Xinjiang Tianchi, 51470; Xinjiang Kuerle, 51656<br>17. Zhejiang Fuyang, 58449; Zhejiang Cixi, 58467; Zhejiang Kaihua, 58537; Zhejiang Dongyang, 58558; Zhejiang Yinzhou, 58562; Zhejiang Changshan, 58631; Zhejiang Jiangshan, 58632; Zhejiang Taishun, 58746; Zhejiang Wencheng, 58750
Cluster B	1. Anhui Taihe, 58109; Anhui Tianzhushan, 58112; Anhui Suixi, 58113; Anhui Woyang, 58114; Anhui Leysin, 58117; Anhui Mengcheng, 58118; Anhui Suzhou, 58122; Anhui Lingbi, 58125; Anhui Sixian, 58126; Anhui Huaiyuan, 58127; Anhui Guzhen, 58128; Anhui Wuhe, 58129; Anhui Yingshang, 58210; Anhui Fengtai, 58212; Anhui Huoqiu, 58214; Anhui Changfeng, 58220; Anhui Fengyang, 58222; Anhui Mingguang, 58223; Anhui Dingyuan, 58225; Anhui Laian, 58234; Anhui Chuzhou, 58236; Anhui Jinzhai, 58306; Anhui Luan, 58311; Anhui Huoshan, 58314; Anhui Shucheng, 58316; Anhui Yuexi, 58317; Anhui Tongcheng, 58319; Anhui Feixi, 58320; Anhui Feidong, 58323; Anhui Chaohu, 58326; Anhui Lujiang, 58327; Anhui Wuwei, 58329; Anhui Hanshan, 58330; Anhui Wuhu, 58334; Anhui Maanshan, 58336; Anhui Wuhuxian, 58338; Anhui Susong, 58417; Anhui Tongling, 58429; Anhui Nanling, 58431; Anhui Jingxian, 58432; Anhui Xuancheng, 58433; Anhui Jingde, 58435; Anhui Ningguo, 58436; Anhui Huangshan, 58437; Anhui Guangde, 58441; Anhui Langxi, 58442; Anhui Shexian, 58530<br>2. Gansu Dongxiang, 52981; Gansu Kongtong, 53915<br>3. Guangdong Lianshan, 59074; Guangdong Yangshan, 59075; Guangdong Yingde, 59088; Guangdong Shixing, 59090; Guangdong Pingyuan, 59106; Guangdong Daipu, 59116; Guangdong Longmen, 59290; Guangdong Wuhua, 59303; Guangdong Zijin, 59304; Guangdong Xinyi, 59456; Guangdong Luoding, 59462; Guangdong Yangchun, 59469; Guangdong Xinxing, 59470; Guangdong Enping, 59477; Guangdong Haifeng, 59500; Guangdong Xuwen, 59754<br>4. Guizhou Hezhang, 56598; Guizhou Puan, 56792; Guizhou Tongzi, 57606; Guizhou Daozhen, 57623; Guizhou Zhengan, 57625; Guizhou Wuchuan, 57634; Guizhou Yanhe, 57636; Guizhou Dejiang, 57637; Guizhou Songtao, 57647; Guizhou Bijie, 57707; Guizhou Dafang, 57708; Guizhou Jinsha, 57714; Guizhou Zunyi, 57717; Guizhou Xifeng, 57718; Guizhou Kaiyang, 57719; Guizhou Meitan, 57722; Guizhou Fenggang, 57723; Guizhou Wengan, 57728; Guizhou Yuqing, 57729; Guizhou Sinan, 57731; Guizhou Shiqian, 57734; Guizhou Cengong, 57735; Guizhou Jiangkou, 57736; Guizhou Shibing, 57737; Guizhou Yuping, 57739; Guizhou Wanshan, 57742; Guizhou Nayong, 57800; Guizhou Xianxi, 57803; Guizhou Zhijin, 57805; Guizhou Anshun, 57806; Guizhou Liuzhi, 57807; Guizhou Xiuwen, 57811; Guizhou Pingba, 57814; Guizhou Fuquan, 57821; Guizhou Huangping, 57822; Guizhou Guiding, 57824; Guizhou Kaili, 57825; Guizhou Duyun, 57827; Guizhou Sansui, 57832; Guizhou Taijiang, 57834; Guizhou Jianhe, 57835; Guizhou Leishan, 57837; Guizhou Liping, 57839;

	Guizhou Tianzhu, 57840; Guizhou Jinping, 57844; Guizhou Qinglong, 57900; Guizhou Guanling, 57903; Guizhou Zhenfeng, 57905; Guizhou Wangmo, 57906; Guizhou Ziyun, 57910; Guizhou Huishui, 57912; Guizhou Longli, 57913; Guizhou Luodian, 57916; Guizhou Dushan, 57922; Guizhou Sandu, 57923; Guizhou Rongjiang, 57932<br>5. Hainan Haikou, 59758; Hainan Lingao, 59842; Hainan Danzhou, 59845; Hainan Dingan, 59851; Hainan Qonghai, 59855; Hainan Wenchang, 59856; Hainan Baoting, 59945; Hainan Wanning, 59951<br>6. Hubei Padang, 57355<br>7. Hunan Baojing, 57642; Hunan Fenghuang, 57740; Hunan Xinhuang, 57744; Hunan Zhijiang, 57745; Hunan Tongtao, 57845; Hunan Shuangpai, 57962; Hunan Rucheng, 57985<br>8. Jiangsu Fengxian, 58012; Jiangsu Peixian, 58013; Jiangsu Suining, 58130; Jiangsu Xuyi, 58138; Jiangsu Pukou, 58237; Jiangsu Nanjing, 58238; Jiangsu Gaochun, 58339; Jiangsu Yixing, 58346<br>9. Jiangxi Xunwu 59102<br>10. Ningxia Helan, 53610; Ningxia Zhongwei, 53704<br>11. Shanxi Hejin, 53957; Shanxi Yongji, 57052<br>12. Shaanxi Yanchuan, 53850; Shaanxi Pucheng, 53948; Shaanxi Qianyang, 57021; Shaanxi Linyou, 57022; Shaanxi Meixian, 57027; Shaanxi Liquan, 57029; Shaanxi Yongshou, 57030; Shaanxi Yaoxian, 57037; Shaanxi Sanyuan, 57041; Shaanxi Fuping, 57042; Shaanxi Weinan, 57045; Shaanxi Mianxian, 57119; Shaanxi Langao, 57247; Shaanxi Zhenping, 57343<br>13. Sichuan Linshui, 57416; Sichuan Wusheng, 57417<br>14. Yunnan Yiliang, 56594; Yunnan Zhenxiong, 56595<br>15. Zhejiang Anji, 58446; Zhejiang Linan, 58448; Zhejiang Huzhou, 58450; Zhejiang Jiashan, 58451; Zhejiang Jiaxing, 58452; Zhejiang Shaoxing, 58453; Zhejiang Deqing, 58454; Zhejiang Hangzhou, 58457; Zhejiang Shengsi, 58472; Zhejiang Daishan, 58484; Zhejiang Tonglu, 58542; Zhejiang Jiande, 58544; Zhejiang Pujiang, 58546; Zhejiang Longyou, 58547; Zhejiang Jinhua, 58549; Zhejiang Zhuji, 58550; Zhejiang Xinchang, 58555; Zhejiang Shengzhou, 58556; Zhejiang Tiantai, 58559; Zhejiang Panan, 58560; Zhejiang Zhenhai, 58561; Zhejiang Fenghua, 58565; Zhejiang Xiangshan, 58566; Zhejiang Ninghai, 58567; Zhejiang Shipu, 58569; Zhejiang Putuo, 58570; Zhejiang Wuyi, 58642; Zhejiang Yongkang, 58643; Zhejiang Suichang, 58644; Zhejiang Lishui, 58646; Zhejiang Xianju, 58652; Zhejiang Jinyun, 58654; Zhejiang Leqing, 58656; Zhejiang Qingtian, 58657; Zhejiang Yongjia, 58658; Zhejiang Linhai, 58660; Zhejiang Wenling, 58664; Zhejiang Hongjia, 58665; Zhejiang Dachen, 58666; Zhejiang Yunhe, 58742; Zhejiang Pingyang, 58751; Zhejiang Jingning, 58648<br>16. Chongqing Chengkou, 57333; Chongqing Kaixian, 57338; Chongqing Yunyang, 57339; Chongqing Wuxi, 57345; Chongqing Wushan, 57349; Chongqing Dianjiang, 57425; Chongqing Wanzhou, 57432; Chongqing Zhongxian, 57437; Chongqing Shizhu, 57438; Chongqing Wansheng, 57509; Chongqing Tongliang, 57510; Chongqing Beibei, 57511; Chongqing Yubei, 57513; Chongqing Bishan,

	57514; Chongqing Jiangjin, 57517; Chongqing Banan, 57518; Chongqing Nanchuan, 57519; Chongqing Changshou, 57520; Chongqing Fengdu, 57523; Chongqing Wulong, 57525; Chongqing Qianjiang, 57536; Chongqing Pengshui, 57537; Chongqing Qijiang, 57612
Cluster C	1. Gansu Mazongshan, 52323; Gansu Subei, 52515; Gansu Jiuquan, 52533; Gansu Sunan, 52643; Gansu Minle, 52656; Gansu Wuwei, 52679; Gansu Minqin, 52681; Gansu Gulang, 52784; Gansu Wushaoling, 52787; Gansu Tianzhu, 52881; Gansu Yongdeng, 52885; Gansu Yuzhong, 52983; Gansu Hezheng, 52985; Gansu Kangle, 52988; Gansu Huining, 52993; Gansu Anding, 52995; Gansu Huajialing, 52996; Gansu Huanxian, 53821; Gansu Lingtai, 53924; Gansu Zhenyuan, 53925; Gansu Jingchuan, 53926; Gansu Huating, 53927; Gansu Huachi, 53930; Gansu Huishui, 53934; Gansu Luqu, 56071; Gansu Lintan, 56081; Gansu Zhuoni, 56082; Gansu Zhangxian, 56091; Gansu Dangchang, 56095; Gansu Wudu, 56096; Gansu Gangu, 57001; Gansu Wushan, 57004; Gansu Qingshui, 57011; Gansu Maiji, 57014; Gansu Chengxian, 57102; Gansu Kangxian, 57105<br>2. Hebei Laiyuan, 53599<br>3. Heilongjiang Xinlin, 50349; Heilongjiang Jiagedaqi, 50442<br>4. Jilin Shuangliao, 54142<br>5. Liaoning Shenbei, 54248; Liaoning Xifeng, 54252; Liaoning Qingyuan, 54259; Liaoning Chaoyang, 54324; Liaoning Yangshan, 54325; Liaoning Fushun, 54351; Liaoning Kuandian, 54493; Liaoning Dandong, 54497; Liaoning Changhai, 54579; Liaoning Changxingdao, 54565<br>6. Inner Mongolia Tulihe, 50434; Inner Mongolia Elunchunqi, 50445; Inner Mongolia Manzhouli, 50514; Inner Mongolia Evenkeqi, 50525; Inner Mongolia Xinbaerhuyouqi, 50603; Inner Mongolia Xinbaerhuzuoqi, 50618; Inner Mongolia Zhalantun, 50639; Inner Mongolia Aershan, 50727; Inner Mongolia Wulagai, 50913; Inner Mongolia Houlinguole, 50924; Inner Mongolia Bayaertuhushuo, 50928; Inner Mongolia Erlianhaote, 53068; Inner Mongolia Narenbaolige, 53083; Inner Mongolia Mandula, 53149; Inner Mongolia Abagaqi, 53192; Inner Mongolia Hailisu, 53231; Inner Mongolia Xianghuangqi, 53289; Inner Mongolia Wulatezhongqi, 53336; Inner Mongolia Dashetai, 53348; Inner Mongolia Xilamuren, 53367; Inner Mongolia Chayouzhongqi, 53378; Inner Mongolia Huade, 53391; Inner Mongolia Dengkou, 53419; Inner Mongolia Hangjinhouqi , 53420; Inner Mongolia Wulateqianqi, 53433; Inner Mongolia Baotou, 53446; Inner Mongolia Tuyouqi, 53455; Inner Mongolia Dalateqi, 53457; Inner Mongolia Huhehaote suburb, 53466; Inner Mongolia Tuoketuoxian, 53467; Inner Mongolia Helingeerxian, 53469; Inner Mongolia Zhuozi, 53472; Inner Mongolia Liangcheng, 53475; Inner Mongolia Xinghe, 53483; Inner Mongolia Wuhai, 53512; Inner Mongolia Yikewusu, 53522; Inner Mongolia Etukeqi, 53529; Inner Mongolia Hangjinqi, 53533; Inner Mongolia Dongsheng, 53543; Inner Mongolia Ejinhuoluoqi, 53545; Inner Mongolia Zhungeerqi, 53553; Inner Mongolia Qingshuihexian, 53562; Inner Mongolia Wushenqi, 53644; Inner Mongolia Xiwuzhumuqin, 54012; Inner Mongolia Fuhe,

	54024; Inner Mongolia Balinzuoqi, 54027; Inner Mongolia Xilinhaote, 54102; Inner Mongolia Balinyouqi, 54113; Inner Mongolia Linxixian, 54115; Inner Mongolia Keshiketengqi, 54117; Inner Mongolia Qinglongshan, 54132; Inner Mongolia Tongliao, 54135; Inner Mongolia Zhenglanqi, 54205; Inner Mongolia Gangzi, 54214; Inner Mongolia Chifeng, 54218; Inner Mongolia Aohanqi, 54225; Inner Mongolia Baoguotu, 54226; Inner Mongolia Kezuohouqi, 54231; Inner Mongolia Taibushiqi, 54305; Inner Mongolia Kalaqinqi, 54313; Inner Mongolia Balihan, 54316; Inner Mongolia Ningchengxian, 54320<br>7. Ningxia Shitanjing, 53517; Ningxia Wuzhong, 53612; Ningxia Zhongning, 53705; Ningxia Xingren, 53707; Ningxia Yanchi, 53723; Ningxia Maihuangshan, 53727; Ningxia Haiyuan, 53806; Ningxia Tongxin, 53810; Ningxia Guyuan, 53817; Ningxia Xiji, 53903; Ningxia Liupanshan, 53910<br>8. Qinghai Lenghu, 52602; Qinghai Nuomuhong, 52825; Qinghai Doulan, 52836; Qinghai Qinghaihu 151, 52854; Qinghai Gonghe, 52856; Qinghai Huzhu, 52863; Qinghai Xining, 52866; Qinghai Guide, 52868; Qinghai Pingan, 52875; Qinghai Minhe, 52876; Qinghai Hualong, 52877; Qinghai Wudaoliang, 52908; Qinghai Shazhuyu, 52941; Qinghai Xinghai, 52943; Qinghai Guinan, 52955; Qinghai Tongde, 52957; Qinghai Xunhua, 52972; Qinghai Tongren, 52974; Qinghai Tuotuohe, 56004; Qinghai Zhiduo, 56016; Qinghai Zaduo, 56018; Qinghai Qumacai, 56021; Qinghai Yushu, 56029; Qinghai Qingshuihe, 56034; Qinghai Maqin, 56043; Qinghai Gander, 56045; Qinghai Dari, 56046; Qinghai Henan, 56065; Qinghai Jiuzhi, 56067<br>9. Shaanxi Zhidan, 53832<br>10. Xinjiang Buerjin, 51060; Xinjiang Fuyun, 51087; Xinjiang Emin, 51145; Xinjiang Hebukesaier, 51156; Xinjiang Bole, 51238; Xinjiang Touli, 51241; Xinjiang Kelamayi, 51243; Xinjiang Jinghe, 51334; Xinjiang Shawan, 51357; Xinjiang Changji, 51368; Xinjiang Miquan, 51369; Xinjiang Tianshandaxigou, 51468; Xinjiang Urumqi Pastoral Test Station, 51469; Xinjiang Dabancheng, 51477; Xinjiang Mulei, 51482; Xinjiang Kumishi, 51526; Xinjiang Tuokexun, 51571; Xinjiang Tulufandongkan, 51572; Xinjiang Tulufan, 51573; Xinjiang Xinhe, 51636; Xinjiang Wuqia, 51705; Xinjiang Kashi, 51709; Xinjiang Yuepuhu, 51717; Xinjiang Alaer, 51730; Xinjiang Tazhong, 51747; Xinjiang Tieqianlike, 51765; Xinjiang Yecheng, 51814; Xinjiang Pishan, 51818; Xinjiang Moyu, 51827; Xinjiang Minfeng, 51839; Xinjiang Qiemuo, 51855; Xinjiang Yutian, 51931; Xinjiang Balikun, 52101; Xinjiang Naomaohu, 52112; Xinjiang Yiwu, 52118; Xinjiang Hami, 52203; Xinjiang Hongliuhe, 52313
Cluster D	1. Beijing Yanqing, 54406; Beijing Zhaitang, 54501; Beijing, 54511; Beijing Xiayunling, 54597<br>2. Hebei Kangbao, 53392; Hebei Zhangbei, 53399; Hebei Xuanhua, 53498; Hebei Wanquan, 53499; Hebei Shunping, 53596; Hebei Lingshou, 53680; Hebei Quyang, 53682; Hebei Xingtang, 53688; Hebei Jinzhou, 53689; Hebei Fuping, 53690; Hebei Tangxian, 53692; Hebei Dingzhou, 53696; Hebei Wuji, 53699; Hebei Shahe, 53781; Hebei Baixiang, 53785; Hebei Luancheng, 53789; Hebei Longyao, 53794; Hebei Zanhuang, 53795; Hebei Ningjin, 53796; Hebei Julu,

	53799; Hebei Wuan, 53890; Hebei Handan, 53892; Hebei Guyuan, 54301; Hebei Chongli, 54304; Hebei Fengning, 54308; Hebei Weichang, 54311; Hebei Longhua, 54318; Hebei Pingquan, 54319; Hebei Zhangjiakou, 54401; Hebei Chicheng, 54404; Hebei Huailai, 54405; Hebei Zhulu, 54408; Hebei Chengde, 54423; Hebei Zunhua, 54429; Hebei Chengdexian, 54430; Hebei Kuancheng, 54432; Hebei Qianxi, 54434; Hebei Qinglong, 54436; Hebei Luannan, 54437; Hebei Lulong, 54438; Hebei Qianan, 54439; Hebei Qinhuangdao, 54449; Hebei Zhuozhou, 54502; Hebei Rongcheng, 54503; Hebei Gaobeidian, 54506; Hebei Dachang, 54510; Hebei Guan, 54512; Hebei Yongqing, 54519; Hebei Sanhe, 54520; Hebei Xianghe, 54521; Hebei Tangshan, 54534; Hebei Caofeidian, 54535; Hebei Leting, 54539; Hebei Changli, 54540; Hebei Funing, 54541; Hebei Xushui, 54601; Hebei Goyang, 54603; Hebei Anxin, 54605; Hebei Raoyang, 54606; Hebei Shenzhou, 54608; Hebei Renqiu, 54610; Hebei Wenan, 54612; Hebei Dacheng, 54613; Hebei Hejian, 54614; Hebei Qingxian, 54615; Hebei Cangzhou, 54616; Hebei Botou, 54618; Hebei Huanghua, 54624; Hebei Haixing, 54628; Hebei Guangzong, 54631; Hebei Xinhe, 54633; Hebei Jize, 54640; Hebei Wuqiang, 54700; Hebei Xinji, 54701; Hebei Hengshui, 54702; Hebei Wuyi, 54703; Hebei Gucheng, 54707; Hebei Fucheng, 54710; Hebei Jingxian, 54711; Hebei Dongguang, 54713; Hebei-Weixian, 54800; Hebei Linxi, 54801; Hebei Guantao, 54809<br>3. Henan Taiqian, 54817; Henan Puyang, 54900<br>4. Liaoning Jianchang, 54452<br>5. Shandong Wucheng, 54709; Shandong Linyi, 54712; Shandong Ningjin, 54716; Shandong Yangxin, 54723; Shandong Shanghe, 54724; Shandong Leling, 54726; Shandong Zhangqiu, 54727; Shandong Gaoqing, 54729; Shandong Binzhou, 54734; Shandong Kenli, 54744; Shandong Laizhou, 54749; Shandong Longdao, 54751; Shandong Penglai, 54752; Shandong Longkou, 54753; Shandong Zhaoyuan, 54755; Shandong Qixia, 54759; Shandong Fushan, 54764; Shandong Yantai, 54765; Shandong Chengshantou, 54776; Shandong Wendeng, 54777; Shandong Linqing, 54802; Shandong Liaocheng, 54806; Shandong Qihe, 54812; Shandong Chiping, 54814; Shandong Donge, 54815; Shandong Feicheng, 54819; Shandong Jiyang, 54821; Shandong Zouping, 54822; Shandong Jinan, 54823; Shandong Taian, 54827; Shandong Laiwu, 54828; Shandong Zibo, 54830; Shandong Qingzhou, 54831; Shandong Shouguang, 54832; Shandong Huantai, 54833; Shandong Yiyuan, 54836; Shandong Changyi, 54841; Shandong Pingdu, 54842; Shandong Weifang, 54843; Shandong Anqiu, 54844; Shandong Gaomi, 54846; Shandong Zhucheng, 54848; Shandong Jiaozhou, 54849; Shandong Laiyang, 54852; Shandong Jimo, 54855; Shandong Rushan, 54861; Shandong Juancheng, 54904; Shandong Yuncheng, 54905; Shandong Heze, 54906; Shandong Dingtao, 54909; Shandong Liangshan, 54910; Shandong Dongping, 54911; Shandong Wenshang, 54912; Shandong Juye, 54914; Shandong Yanzhou, 54916; Shandong Zoucheng, 54919; Shandong Sishui, 54920; Shandong Xintai, 54922; Shandong Mengyin, 54923; Shandong Pingyi, 54925; Shandong Tengzhou, 54927; Shandong Feixian, 54929; Shandong Yishui,

		54932; Shandong Linyi, 54938; Shandong Junan, 54939; Shandong Wulian, 54940; Shandong Huangdao, 54943; Shandong Rizhao, 54945; Shandong Chengwu, 58003; Shandong Zaozhuang, 58024; Shandong Cangshan, 58030; Shandong Linshu, 58032
	6.	Tianjin Jinghai, 54619; Tianjin Jinnan , 54622
Cluster E	1.	Anhui Tianchang, 58240
	2.	Gansu Liangdang, 57111
	3.	Hainan Chengmai, 59843; Hainan Changjiang, 59847; Hainan Baisha, 59848; Hainan Tunchang, 59854
	4.	Henan Xinye, 57271; Henan Biyang, 57281; Henan Tongbai, 57285; Henan Jigongshan, 57390; Henan Xixian, 57396; Henan Shangcheng, 58301
	5.	Hubei Zhuxi, 57249; Hubei Yunxi, 57251; Hubei Yunxian, 57253; Hubei Shiyan, 57256; Hubei Zhushan, 57257; Hubei Fangxian, 57259; Hubei Danjiangkou, 57260; Hubei Gucheng, 57268; Hubei Xiangyang, 57278; Hubei Zaoyang, 57279; Hubei Xingshan, 57359; Hubei Baokang, 57361; Hubei Shennongjia, 57362; Hubei Nanzhang, 57363; Hubei Yicheng, 57370; Hubei Jingmen, 57377; Hubei Zhongxiang, 57378; Hubei Suizhou, 57381; Hubei Xiaochang, 57386; Hubei Jingshan, 57387; Hubei Anlu, 57388; Hubei Hongan, 57398; Hubei Macheng, 57399; Hubei Lichuan, 57439; Hubei Jianshi, 57445; Hubei Enshi, 57447; Hubei Yiling , 57453; Hubei Wufeng, 57458; Hubei Songzi, 57469; Hubei Qianjiang, 57475; Hubei Gongan, 57477; Hubei Yingcheng, 57481; Hubei Xiaogan, 57482; Hubei Tianmen, 57483; Hubei Shayang, 57484; Hubei Xiantao, 57485; Hubei Hanchuan, 57486; Hubei Caidian, 57489; Hubei Xinzhou, 57492; Hubei Wuhan, 57494; Hubei Tuanfeng, 57495; Hubei Ezhou, 57496; Hubei Xianfeng, 57540; Hubei Xuanen, 57541; Hubei Jiayu, 57583; Hubei Chongyang, 57586; Hubei Xianning, 57590; Hubei Luotian, 58401; Hubei Yingshan, 58402; Hubei Qichun, 58408; Hubei Huangmei, 58409; Hubei Yangxin, 58500
	6.	Jiangsu Pizhou, 58026; Jiangsu Xuzhou, 58027; Jiangsu Donghai, 58036; Jiangsu Shuyang, 58038; Jiangsu Ganyu, 58040; Jiangsu Xiliandao, 58041; Jiangsu Lianyungang, 58044; Jiangsu Xiangshui, 58045; Jiangsu Guanyun, 58047; Jiangsu Siyang, 58132; Jiangsu Sihong, 58135; Jiangsu Hongze, 58139; Jiangsu Lianshui, 58140; Jiangsu Huaian, 58141; Jiangsu Funing, 58143; Jiangsu Jianhu, 58146; Jiangsu Jinhu, 58147; Jiangsu Baoying, 58148; Jiangsu Yancheng, 58154; Jiangsu Dafeng, 58158; Jiangsu Liuhe, 58235; Jiangsu Gaoyou, 58241; Jiangsu Yizheng, 58242; Jiangsu Xinghua, 58243; Jiangsu Taizhou, 58246; Jiangsu Yangzhong, 58247; Jiangsu Taixing, 58249; Jiangsu Jiangyan, 58250; Jiangsu Dongtai, 58251; Jiangsu Haian, 58254; Jiangsu Rugao, 58255; Jiangsu Jingjiang, 58257; Jiangsu Nantong, 58259; Jiangsu Rudong, 58264; Jiangsu Lvsi, 58265; Jiangsu Lishui, 58340; Jiangsu Danyang, 58341; Jiangsu Jintan, 58342; Jiangsu Jurong, 58344; Jiangsu Suzhou, 58349; Jiangsu Changshu, 58352; Jiangsu Zhangjiagang, 58353; Jiangsu Wuxi, 58354; Jiangsu Kunshan, 58356; Jiangsu Haimen, 58360; Jiangsu Taicang, 58377
	7.	Inner Mongolia Henan, 53732
	8.	Shandong Yutai, 54907; Shandong Xuechen, 58021; Shandong Yicheng, 58022

	9. Shanxi Shilou, 53759; Shanxi Yonghe, 53852; Shanxi Jixian, 53859; Shanxi Ruicheng, 57053<br>10. Shaanxi Fugu, 53567; Shaanxi Yulin, 53646; Shaanxi Shenmu, 53651; Shaanxi Dingbian, 53725; Shaanxi Jingbian, 53735; Shaanxi Wuqi, 53738; Shaanxi Hengshan, 53740; Shaanxi Zichang, 53748; Shaanxi Suide, 53754; Shaanxi Ansai, 53841; Shaanxi Ganquan, 53848; Shaanxi Yanchang, 53854; Shaanxi Fuxian, 53931; Shaanxi Xunyi, 53938; Shaanxi Baishui, 53941; Shaanxi Huangling, 53944; Shaanxi Huanglong, 53946; Shaanxi Longxian, 57003; Shaanxi Baoji, 57016; Shaanxi Fufeng, 57026; Shaanxi Xingping, 57038; Shaanxi Huashan, 57046; Shaanxi Lueyang, 57106; Shaanxi Fengxian, 57113; Shaanxi Liuba, 57124; Shaanxi Foping, 57134; Shaanxi Ningshan, 57137; Shaanxi Shangxian, 57143; Shaanxi Danfeng, 57153; Shaanxi Shangnan, 57154; Shaanxi Shanyang, 57155; Shaanxi Nanzheng, 57213; Shaanxi Ziyang, 57231; Shaanxi Hanyin, 57233; Shaanxi Zhenba, 57238; Shaanxi Ankang, 57245; Shaanxi Pingli, 57248; Shaanxi Baihe, 57254<br>11. Zhejiang Changxing, 58443
Cluster F	1. Gansu Xiahe, 52978; Gansu Yongjing, 52980; Gansu Guanghe, 52982; Gansu Lintao, 52986; Gansu Weiyuan, 52998; Gansu Jingning, 53906; Gansu Zhuanglang, 53917; Gansu Hezuo, 56080; Gansu Dibu, 56084; Gansu Longxi, 56092; Gansu Minxian, 56093; Gansu Lixian, 57007<br>2. Hebei Shangyi, 53397; Hebei Huaian, 53491; Hebei Weixian, 53593<br>3. Henan Linzhou, 53889<br>4. Heilongjiang Aihui, 50468; Heilongjiang Nenjiang, 50557; Heilongjiang Nehe, 50646; Heilongjiang Beian, 50656; Heilongjiang Keshan, 50658; Heilongjiang Kedong, 50659; Heilongjiang Longjiang, 50739; Heilongjiang Qiqihaer, 50745; Heilongjiang Yian, 50750; Heilongjiang Hailun, 50756; Heilongjiang Minshui, 50758; Heilongjiang Suileng, 50767; Heilongjiang Wuying, 50772; Heilongjiang Hegang, 50775; Heilongjiang Luobei, 50776; Heilongjiang Tongjiang, 50778; Heilongjiang Fuyuan, 50779; Heilongjiang Dumeng, 50842; Heilongjiang Wangkui, 50852; Heilongjiang Zhaodong, 50858; Heilongjiang Lansi, 50859; Heilongjiang Qingan, 50861; Heilongjiang Tangyuan, 50871; Heilongjiang Jiamusi, 50873; Heilongjiang Yilang, 50877; Heilongjiang Huachuan, 50878; Heilongjiang Hulan, 50956; Heilongjiang Acheng, 50958; Heilongjiang Binxian, 50960; Heilongjiang Zhengfang, 50964; Heilongjiang Yanshou, 50965; Heilongjiang Qitaihe, 50971; Heilongjiang Boli, 50973; Heilongjiang Jixi, 50978; Heilongjiang Linkou, 50979; Heilongjiang Jidong, 50987; Heilongjiang Wuchang, 54080; Heilongjiang Hailin, 54092; Heilongjiang Muling, 54093; Heilongjiang Mudanjiang, 54094; Heilongjiang Suifenhe, 54096; Heilongjiang Ningan, 54098<br>5. Liaoning Zhangwu, 54236; Liaoning Jianpingzhen, 54321; Liaoning Lianshan, 54453; Liaoning Suizhong, 54454; Liaoning Xingcheng, 54455; Liaoning Zhuanghe, 54584<br>6. Inner Mongolia Eerguna, 50425; Inner Mongolia Yakeshi, 50526; Inner Mongolia Xiaoergou, 50548; Inner Mongolia Moulidawawoer, 50645; Inner Mongolia Arunqi, 50647; Inner Mongolia Suolun, 50834; Inner Mongolia

		Wuzhumuqindong, 50915; Inner Mongolia Tuquan, 50934; Inner Mongolia Wuyuan, 53337; Inner Mongolia Guyangxian, 53357; Inner Mongolia Siziwang, 53362; Inner Mongolia Wuchuanxian, 53368; Inner Mongolia Chayouhouqi, 53384; Inner Mongolia Shangdu, 53385; Inner Mongolia Jining, 53480; Inner Mongolia Chayouqianqi, 53481; Inner Mongolia Linhe, 53513; Inner Mongolia Eduokeqianqi, 53730; Inner Mongolia Zhalute, 54026; Inner Mongolia Gaoliban, 54031; Inner Mongolia Shebotu, 54039; Inner Mongolia Kezuozhongqi, 54047; Inner Mongolia Alukeerqinqi, 54122; Inner Mongolia Kailu, 54134; Inner Mongolia Zhengxiangbaiqi, 54204; Inner Mongolia Duolunxian, 54208; Inner Mongolia Wengniuteqi, 54213; Inner Mongolia Neiman, 54223; Inner Mongolia Kulun, 54234
	7.	Ningxia Weizhou, 53881
	8.	Shanxi Youyu, 53478; Shanxi Yanggao, 53486; Shanxi Datong, 53487; Shanxi Shenchi, 53575; Shanxi Shuozhou, 53578; Shanxi Hunyuan, 53582; Shanxi Wutaishan, 53588; Shanxi Guangling, 53590; Shanxi Dingxiang, 53676; Shanxi Xiaodian, 53679; Shanxi Fangshan, 53760; Shanxi Qingxu, 53774; Shanxi Heshun, 53788; Shanxi Hongtong, 53866; Shanxi Guxian, 53874; Shanxi Lucheng, 53880; Shanxi Jishan, 53954; Shanxi Wanrong, 53956; Shanxi Yanhu, 53959; Shanxi Fushan, 53966; Shanxi Yuanqu, 53968
	9.	Xinjiang Akedala, 51058; Xinjiang Fuhai, 51068; Xinjiang Aletai, 51076; Xinjiang Manasi, 51359; Xinjiang Hutubi, 51367; Xinjiang Fukang, 51377; Xinjiang Jimusaer, 51378; Xinjiang Bayinbuluke, 51542; Xinjiang Yanqi, 51567; Xinjiang Wushi, 51627; Xinjiang Akesu, 51628; Xinjiang Shaya, 51639; Xinjiang Atushi, 51704; Xinjiang Jiashi, 51707; Xinjiang Kepin, 51720; Xinjiang Awati, 51722; Xinjiang Yengjisha, 51802; Xinjiang Maigaiti, 51810; Xinjiang Shashe, 51811; Xinjiang Zepu, 51815; Xinjiang Cele, 51826; Xinjiang Hetan, 51828; Xinjiang Luopu, 51829
Cluster G	1.	Gansu Zhouqu, 56094; Gansu Wenxian, 56192
	2.	Qinghai Banma, 56151
	3.	Shaanxi Ningqiang, 57211
	4.	Sichuan Shiqu, 56038; Sichuan Ruoergai, 56079; Sichuan Jiuzhaigou, 56097; Sichuan Ganzi, 56146; Sichuan Seda, 56152; Sichuan Luhuo, 56158; Sichuan Rangtang, 56164; Sichuan Daofu, 56167; Sichuan Jinchuan, 56168; Sichuan Aba, 56171; Sichuan Maerkang, 56172; Sichuan Hongyuan, 56173; Sichuan Xiaojin, 56178; Sichuan Maoxian, 56180; Sichuan Chongzhou, 56181; Sichuan Songpan, 56182; Sichuan Wenchuan, 56183; Sichuan Lixian, 56184; Sichuan Heishui, 56185; Sichuan Mianzhu, 56186; Sichuan Wenjiang, 56187; Sichuan Dujiangyan, 56188; Sichuan Pengzhou, 56189; Sichuan Deyang, 56198; Sichuan Batang, 56247; Sichuan Xinlong, 56251; Sichuan Litang, 56257; Sichuan Danba, 56263; Sichuan Yajiang, 56267; Sichuan Baoxing, 56273; Sichuan Lushan, 56279; Sichuan Mingshan, 56280; Sichuan Pujiang, 56281; Sichuan Longquanyi, 56286; Sichuan Pengshan, 56289; Sichuan Jintang, 56296; Sichuan Renshou, 56297; Sichuan Ziyang, 56298; Sichuan Daocheng, 56357; Sichuan Luding, 56371; Sichuan Yingjing, 56373; Sichuan Kangding, 56374; Sichuan Hanyuan,

	56376; Sichuan Shimian, 56378; Sichuan Hongya, 56380; Sichuan Jiajiang, 56382; Sichuan Qingshen, 56383; Sichuan Emeishan, 56385; Sichuan Ebian, 56387; Sichuan Qianwei, 56389; Sichuan Jingyan, 56390; Sichuan Zizhong, 56393; Sichuan Weiyuan, 56395; Sichuan Zigong, 56396; Sichuan Fushun, 56399; Sichuan Xiangcheng, 56443; Sichuan Muli, 56459; Sichuan Jiulong, 56462; Sichuan Ganluo, 56473; Sichuan Mianning, 56474; Sichuan Yuexi, 56475; Sichuan Xide, 56478; Sichuan Chaojue, 56479; Sichuan Mabian, 56480; Sichuan Leibo, 56485; Sichuan Meigu, 56487; Sichuan Muchuan, 56490; Sichuan Yibinxian, 56491; Sichuan Nanxi, 56493; Sichuan Xingwen, 56496; Sichuan Yanyuan, 56565; Sichuan Dechang, 56569; Sichuan Xichang, 56571; Sichuan Puge, 56575; Sichuan Butuo, 56580; Sichuan Jinyang, 56584; Sichuan Changning, 56593; Sichuan Yanbian, 56665; Sichuan Miyi, 56670; Sichuan Huili, 56671; Sichuan Huidong, 56675; Sichuan Qingchuan, 57204; Sichuan Guangyuan, 57206; Sichuan Jiange, 57208; Sichuan Nanjiang, 57216; Sichuan Wangcang, 57217; Sichuan Wanyuan, 57237; Sichuan Langzhong, 57306; Sichuan Xichong, 57309; Sichuan Bazhong, 57313; Sichuan Nanbu, 57314; Sichuan Yilong, 57315; Sichuan Yingshan, 57318; Sichuan Tongjiang, 57320; Sichuan Pingchang, 57324; Sichuan Dachuan, 57328; Sichuan Kaijiang, 57329; Sichuan Shehong, 57401; Sichuan Suining, 57405; Sichuan Gaoping, 57411; Sichuan Quxian, 57413; Sichuan Guangan, 57415; Sichuan Dazhu, 57420; Sichuan Dongxing, 57503; Sichuan Longchang, 57507; Sichuan Jiangan, 57600; Sichuan Hejiang, 57603; Sichuan Xuyong, 57608<br>5. Yunnan Suijiang, 56483; Yunnan Qiaojia, 56673<br>6. Chongqing Tongnan, 57409; Chongqing Dazu, 57502; Chongqing Rongchang, 57505; Chongqing Yongchuan, 57506
Cluster H	1. Guizhou Weining, 56691; Guizhou Panxian, 56793; Guizhou Xingyi, 57907<br>2. Sichuan Derong, 56441; Sichuan Pingshan, 56494; Sichuan Gongxian, 56499; Sichuan Panzhihua, 56666<br>3. Yunnan Deqin, 56444; Yunnan Yanjin, 56497; Yunnan Xianggelila, 56543; Yunnan Weixi, 56548; Yunnan Ninglang, 56567; Yunnan Daguan, 56582; Yunnan Ludian, 56585; Yunnan Weixin, 56596; Yunnan Liuku, 56643; Yunnan Lanping, 56645; Yunnan Jianchuan, 56646; Yunnan Eryuan, 56649; Yunnan Lijiang, 56651; Yunnan Yongsheng, 56652; Yunnan Heqing, 56654; Yunnan Huaping, 56664; Yunnan Yongren, 56669; Yunnan Dongchuan, 56688; Yunnan Xuanwei, 56697; Yunnan Tengchong, 56739; Yunnan Yunlong, 56742; Yunnan Yangbi, 56745; Yunnan Yongping, 56746; Yunnan Baoshan, 56748; Yunnan Dali, 56751; Yunnan Binchuan, 56752; Yunnan Midu, 56755; Yunnan Weishan, 56757; Yunnan Yaoan, 56764; Yunnan Mouding, 56766; Yunnan Nanhua, 56767; Yunnan Fumin, 56772; Yunnan Wuding, 56774; Yunnan Lufeng, 56777; Yunnan Kunming, 56778; Yunnan Malong, 56782; Yunnan Qujing, 56783; Yunnan Songming, 56785; Yunnan Fuyuan, 56790; Yunnan Lianghe, 56840; Yunnan Longling, 56841; Yunnan Shidian, 56842; Yunnan Changning, 56843; Yunnan Fengqing, 56846; Yunnan Yongde, 56849; Yunnan Yunxian, 56854; Yunnan Jingdong, 56856; Yunnan Shuangbai, 56862; Yunnan Anning, 56863; Yunnan

	Zhenyuan, 56867; Yunnan Xinping, 56869; Yunnan Yimen, 56870; Yunnan Jinning, 56871; Yunnan Chengjiang, 56873; Yunnan Yuxi, 56875; Yunnan Huaning, 56879; Yunnan Yiliang, 56880; Yunnan Shilin, 56881; Yunnan Shizong, 56883; Yunnan Mile, 56885; Yunnan Luxi, 56886; Yunnan Qiubei, 56889; Yunnan Luoping, 56891; Yunnan Eshan, 56898; Yunnan Cangyuan, 56944; Yunnan Gengma, 56946; Yunnan Ximeng, 56948; Yunnan Menglian, 56949; Yunnan Shuangjiang, 56950; Yunnan Lincang, 56951; Yunnan Jinggu, 56952; Yunnan Lancang, 56954; Yunnan Menghai, 56958; Yunnan Mojiang, 56962; Yunnan Simao, 56964; Yunnan Yuanjiang, 56966; Yunnan Mengla, 56969; Yunnan Shiping, 56970; Yunnan Yuanyang, 56976; Yunnan Luchun, 56978; Yunnan Kaiyuan, 56982; Yunnan Gejiu, 56984; Yunnan Yanshan, 56991; Yunnan Xichou, 56992; Yunnan Wenshan, 56994; Yunnan Guangnan, 59007<br>4. Zhejiang Sanmen, 58568; Zhejiang Longquan, 58647
Cluster I	1. Gansu Huixian, 57110<br>2. Guangdong Yunan, 59268<br>3. Guangxi Ziyuan, 57859; Guangxi Tiane, 57927; Guangxi Sanjiang, 57941; Guangxi Longsheng, 57942; Guangxi Rongshui, 57948; Guangxi Yongfu, 57949; Guangxi Lingui, 57954; Guangxi Xingan, 57955; Guangxi Quanzhou, 57960; Guangxi Guanyang, 57964; Guangxi Xilin, 59004; Guangxi Leye, 59012; Guangxi Lingyun, 59015; Guangxi Tianlin, 59017; Guangxi Fengshan, 59021; Guangxi Hechi, 59023; Guangxi Bama, 59027; Guangxi Yizhou, 59034; Guangxi Duan, 59037; Guangxi Xincheng, 59038; Guangxi Liucheng, 59041; Guangxi Luzhai, 59045; Guangxi Pingle, 59053; Guangxi Lipu, 59055; Guangxi Jinxiu, 59057; Guangxi Mengshan, 59058; Guangxi Hezhou, 59065; Guangxi Baise, 59211; Guangxi Debao, 59215; Guangxi Jingxi, 59218; Guangxi Tiandong, 59224; Guangxi Tiandeng, 59227; Guangxi Longan, 59229; Guangxi Mashan, 59230; Guangxi Shanglin, 59235; Guangxi Wuming , 59237; Guangxi Binyang, 59238; Guangxi Xiangzhou, 59241; Guangxi Laibin, 59242; Guangxi Wuxuan, 59246; Guangxi Pingnan, 59255; Guangxi Tengxian, 59256; Guangxi Wuzhou, 59265; Guangxi Cangwu, 59266; Guangxi Daxin, 59421; Guangxi Shangsi, 59429; Guangxi Nanning, 59431; Guangxi Yongning, 59435; Guangxi Hengxian, 59441; Guangxi Lingshan, 59446; Guangxi Pubei, 59448; Guangxi Bobai, 59449; Guangxi Beiliu, 59451; Guangxi Rongxian, 59452; Guangxi Cenxi, 59454; Guangxi Luchuan, 59457; Guangxi Qinzhou, 59632; Guangxi Hepu, 59640<br>4. Guizhou Ceheng, 57909; Guizhou Pingtang, 57921; Guizhou Libo, 57926; Guizhou Congjiang, 57936<br>5. Hebei Xinglong, 54425; Hebei Yutian, 54522<br>6. Hunan Dongan, 57867<br>7. Shaanxi Jiaxian, 53658; Shaanxi Wubao, 53756; Shaanxi Qingjian, 53757; Shaanxi Tongchuan, 53947; Shaanxi Chengcheng, 53949; Shaanxi Heyang, 53950; Shaanxi Wugong, 57034; Shaanxi Dali, 57043; Shaanxi Luonan, 57057; Shaanxi Chenggu, 57128; Shaanxi Zhashui, 57140; Shaanxi Xunyang, 57242<br>8. Tianjin Wuqing , 54523; Tianjin Baodi, 54525; Tianjin Tanggu, 54623; Tianjin Dagang, 54645

Cluster J	1. Heilongjiang Mohe, 50136; Heilongjiang Tahe, 50246; Heilongjiang Huzhong, 50247; Heilongjiang Huma, 50353; Heilongjiang Sunwu, 50564; Heilongjiang Xunke, 50566; Heilongjiang Wuyiling, 50674; Heilongjiang Gannan, 50741; Heilongjiang Fuyu, 50742; Heilongjiang Lindian, 50749; Heilongjiang Baiquan, 50755; Heilongjiang Yichun, 50774; Heilongjiang Suibin, 50787; Heilongjiang Fujin, 50788; Heilongjiang Tailai, 50844; Heilongjiang Qinggang, 50851; Heilongjiang Beilin, 50853; Heilongjiang Anda, 50854; Heilongjiang Tieli, 50862; Heilongjiang Huanan, 50879; Heilongjiang Shuangyashan, 50884; Heilongjiang Baoqing, 50888; Heilongjiang Zhaozhou, 50950; Heilongjiang Haerbin, 50953; Heilongjiang Mulan, 50962; Heilongjiang Tonghe, 50963; Heilongjiang Shangzhi, 50968<br>2. Jilin Baicheng, 50936; Jilin Taonan, 50939; Jilin Zhenlai, 50940; Jilin Daan, 50945; Jilin Songyuan, 50946; Jilin Qianan, 50948; Jilin Qianguo, 50949; Jilin Tongyu, 54041; Jilin Changling, 54049; Jilin Fuyu, 54063; Jilin Nongan, 54064; Jilin Dehui, 54065; Jilin Jiutai, 54069; Jilin Yushu, 54072; Jilin Shulan, 54076; Jilin Gujiazi, 54155; Jilin Changchun, 54161; Jilin Yitong, 54164; Jilin Shuangyang, 54165; Jilin Yantongshan, 54169; Jilin Yongji, 54171; Jilin Jilin Suburb, 54172; Jilin Jiaohe, 54181; Jilin Dunhua, 54186; Jilin Antu, 54187; Jilin Luozigou, 54192; Jilin Wangqing, 54195; Jilin Liaoyuan, 54260; Jilin Tongfeng, 54261; Jilin Panshi, 54263; Jilin Huadian, 54273; Jilin Huinan, 54274; Jilin Jiangyuan, 54279; Jilin Donggang, 54284; Jilin Erdao, 54285; Jilin Helong, 54286; Jilin Yanji, 54292; Jilin Tonghua, 54363; Jilin Baishan, 54371; Jilin Jian, 54377
Cluster K	1. Beijing Miyun, 54416; Beijing Tongzhou, 54431<br>2. Hebei Luanping, 54420<br>3. Heilongjiang Daqing, 50850; Heilongjiang Mishan, 50985<br>4. Shaanxi Yichuan, 53857<br>5. Sichuan Dege, 56144; Sichuan Baiyu, 56147<br>6. Tibet Shiquanhe, 55228; Tibet Gaize, 55248; Tibet Naqu, 55299; Tibet Pulan, 55437; Tibet Dangxiong, 55493; Tibet Lazi, 55569; Tibet Nanmulin, 55572; Tibet Muozhugongka, 55593; Tibet Zedang, 55598; Tibet Dingri, 55664; Tibet Jiangzi, 55680; Tibet Dingqing, 56116; Tibet Leiwuqi, 56128; Tibet Changdu, 56137; Tibet Luolong, 56223; Tibet Bomi, 56227; Tibet Basu, 56228; Tibet Linzhi, 56312; Tibet Miling, 56317; Tibet Zuogong, 56331; Tibet Chayu, 56434<br>7. Xinjiang Habahe, 51053; Xinjiang Tacheng, 51133

Table A3. SO_2 emissions from residential sector in 2008

Cluster A	1. Gansu Anding, 52995; Gansu Baiyin, 52896; Gansu Chengxian, 57102; Gansu Dangchang, 56095; Gansu Dibu, 56084; Gansu Dongxiang, 52981; Gansu Gangu, 57001; Gansu Gaolan, 52884; Gansu Gaitai, 52546; Gansu Gulang, 52784; Gansu Guanghe, 52982; Gansu Huishui, 53934; Gansu Hezuo, 56080; Gansu Hezheng, 52985; Gansu Huachi, 53930; Gansu Huajialing, 52996; Gansu Huating, 53927; Gansu Huanxian, 53821; Gansu Huining, 52993; Gansu Jingchuan, 53926; Gansu Jingtai, 52797; Gansu Jingyuan, 52895; Gansu Jingning, 53906; Gansu Jiuquan, 52533; Gansu Kangle, 52988; Gansu Kangxian, 57105; Gansu Kongtong, 53915; Gansu Lixian, 57007; Gansu Lintan, 56081; Gansu Lintao, 52986; Gansu Linze, 52557; Gansu Lingtai, 53924; Gansu Longxi, 56092; Gansu Luqu, 56071; Gansu Mazongshan, 52323; Gansu Maqu, 56074; Gansu Maiji, 57014; Gansu Minle, 52656; Gansu Minqin, 52681; Gansu Minxian, 56093; Gansu Qinan, 57002; Gansu Qingshui, 57011; Gansu Qingcheng, 53829; Gansu Subei, 52515; Gansu Sunan, 52643; Gansu Tianshui, 57006; Gansu Tianzhu, 52881; Gansu Weiyuan, 52998; Gansu Wushaoling, 52787; Gansu Wudu, 56096; Gansu Wushan, 57004; Gansu Wuwei, 52679; Gansu Xifeng, 53923; Gansu Xiahe, 52978; Gansu Yongchang, 52674; Gansu Yongdeng, 52885; Gansu Yongjing, 52980; Gansu Yuzhong, 52983; Gansu Zhangjiachuan, 57012; Gansu Zhangxian, 56091; Gansu Zhenyuan, 53925; Gansu Zhengning, 53935; Gansu Zhuanglang, 53917; Gansu Zhuoni, 56082<br>2. Hebei Huaian, 53491; Hebei Shangyi, 53397; Hebei Shexian, 53886; Hebei Weixian, 53593<br>3. Henan Linzhou, 53889<br>4. Heilongjiang Acheng, 50958; Heilongjiang Aihui, 50468; Heilongjiang Anda, 50854; Heilongjiang Bayan, 50867; Heilongjiang Baiquan, 50755; Heilongjiang Baoqing, 50888; Heilongjiang Beian, 50656; Heilongjiang Beilin, 50853; Heilongjiang Binxian, 50960; Heilongjiang Boli, 50973; Heilongjiang Daqing, 50850; Heilongjiang Dumeng, 50842; Heilongjiang Zhengfang, 50964; Heilongjiang Fuyuan, 50779; Heilongjiang Fujin, 50788; Heilongjiang Fuyu, 50742; Heilongjiang Gannan, 50741; Heilongjiang Haerbin, 50953; Heilongjiang Hailin, 54092; Heilongjiang Hailun, 50756; Heilongjiang Hegang, 50775; Heilongjiang Hulan, 50956; Heilongjiang Huma, 50353; Heilongjiang Huzhong, 50247; Heilongjiang Hulin, 50983; Heilongjiang Huachuan, 50878; Heilongjiang Huanan, 50879; Heilongjiang Jidong, 50987; Heilongjiang Jixi, 50978; Heilongjiang Jiagedaqi, 50442; Heilongjiang Jiamusi, 50873; Heilongjiang Jiayin, 50673; Heilongjiang Kedong, 50659; Heilongjiang Keshan, 50658; Heilongjiang Lansi, 50859; Heilongjiang Lindian, 50749; Heilongjiang Linkou, 50979; Heilongjiang Longjiang, 50739; Heilongjiang Luobei, 50776; Heilongjiang Mishan, 50985; Heilongjiang Minshui, 50758; Heilongjiang Mohe, 50136; Heilongjiang Mudanjiang, 54094; Heilongjiang Mulan, 50962; Heilongjiang Muling, 54093; Heilongjiang Nehe, 50646; Heilongjiang Nenjiang, 50557; Heilongjiang Ningan, 54098; Heilongjiang Qitaihe, 50971; Heilongjiang

	Qiqihaer, 50745; Heilongjiang Qinggang, 50851; Heilongjiang Qingan, 50861; Heilongjiang Raohe, 50892; Heilongjiang Shangzhi, 50968; Heilongjiang Shuangyashan, 50884; Heilongjiang Suibin, 50787; Heilongjiang Suifenhe, 54096; Heilongjiang Suileng, 50767; Heilongjiang Sunwu, 50564; Heilongjiang Tahe, 50246; Heilongjiang Tailai, 50844; Heilongjiang Tangyuan, 50871; Heilongjiang Tieli, 50862; Heilongjiang Tonghe, 50963; Heilongjiang Tongjiang, 50778; Heilongjiang Wangkui, 50852; Heilongjiang Wuyiling, 50674; Heilongjiang Wuchang, 54080; Heilongjiang Wudalianchi, 50655; Heilongjiang Wuying, 50772; Heilongjiang Xinlin, 50349; Heilongjiang Xunke, 50566; Heilongjiang Yanshou, 50965; Heilongjiang Yichun, 50774; Heilongjiang Yian, 50750; Heilongjiang Yilang, 50877; Heilongjiang Zhaodong, 50858; Heilongjiang Zhaozhou, 50950<br>5. Jilin Antu, 54187; Jilin Baicheng, 50936; Jilin Baishan, 54371; Jilin Changchun, 54161; Jilin Changling, 54049; Jilin Daan, 50945; Jilin Dehui, 54065; Jilin Tongfeng, 54261; Jilin Donggang, 54284; Jilin Dunhua, 54186; Jilin Erdao, 54285; Jilin Fuyu, 54063; Jilin Gujiazi, 54155; Jilin Helong, 54286; Jilin Huadian, 54273; Jilin Huichun, 54291; Jilin Huinan, 54274; Jilin Jilin Suburb, 54172; Jilin Jian, 54377; Jilin Jiangyuan, 54279; Jilin Jiaohe, 54181; Jilin Jiutai, 54069; Jilin Lishu, 54154; Jilin Liaoyuan, 54260; Jilin Linjiang, 54374; Jilin Liuhe, 54267; Jilin Longjing, 54290; Jilin Luozigou, 54192; Jilin Nongan, 54064; Jilin Panshi, 54263; Jilin Qianguo, 50949; Jilin Qianan, 50948; Jilin Shulan, 54076; Jilin Shuangliao, 54142; Jilin Shuangyang, 54165; Jilin Songyuan, 50946; Jilin Taonan, 50939; Jilin Tonghua, 54363; Jilin Tonghuaxian, 54362; Jilin Tongyu, 54041; Jilin Wangqing, 54195; Jilin Yantongshan, 54169; Jilin Yanji, 54292; Jilin Yitong, 54164; Jilin Yongji, 54171; Jilin Yushu, 54072; Jilin Zhenlai, 50940<br>6. Liaoning Anshan, 54339; Liaoning Benxi, 54346; Liaoning Caohekou, 54483; Liaoning Changtu, 54243; Liaoning Changhai, 54579; Liaoning Changxingdao, 54565; Liaoning Chaoyang, 54324; Liaoning Dashiqiao, 54475; Liaoning Dandong, 54497; Liaoning Fengcheng, 54494; Liaoning Fushun, 54351; Liaoning Gaizhou, 54474; Liaoning Jianpingxian, 54326; Liaoning Jianpingzhen, 54321; Liaoning Jinzhou, 54568; Liaoning Kangping, 54244; Liaoning Kuandian, 54493; Liaoning Lianshan, 54453; Liaoning Liaoyangxian, 54345; Liaoning Liaozhong, 54332; Liaoning Lingyuan, 54327; Liaoning Panshan, 54338; Liaoning Pikou, 54575; Liaoning Pulandian, 54569; Liaoning Qingyuan, 54259; Liaoning Shenbei, 54248; Liaoning Shenyang, 54342; Liaoning Sujiatun, 54340; Liaoning Suizhong, 54454; Liaoning Taian, 54336; Liaoning Tieling, 54249; Liaoning Wafangdian, 54563; Liaoning Xifeng, 54252; Liaoning Xinbin, 54353; Liaoning Xinmin, 54333; Liaoning Xingcheng, 54455; Liaoning Xiuyan, 54486; Liaoning Yangshan, 54325; Liaoning Yingkou, 54471; Liaoning Zhangwu, 54236; Liaoning Zhuanghe, 54584<br>7. Inner Mongolia Abagaqi, 53192; Inner Mongolia Alukeerqinqi, 54122; Inner Mongolia Arunqi, 50647; Inner Mongolia Aohanqi, 54225; Inner Mongolia Balihan, 54316; Inner Mongolia Balinyouqi, 54113; Inner Mongolia Balinzuoqi, 54027; Inner Mongolia Bayaertuhushuo, 50928; Inner Mongolia Baotou, 53446;

		Inner Mongolia Baoguotu, 54226; Inner Mongolia Chayouhouqi, 53384; Inner Mongolia Chayouqianqi, 53481; Inner Mongolia Chayouzhongqi, 53378; Inner Mongolia Chifeng, 54218; Inner Mongolia Dalateqi, 53457; Inner Mongolia Damaoqi, 53352; Inner Mongolia Dashetai, 53348; Inner Mongolia Dengkou, 53419; Inner Mongolia Dongsheng, 53543; Inner Mongolia Wuzhumuqindong, 50915; Inner Mongolia Duolunxian, 54208; Inner Mongolia Eerguna, 50425; Inner Mongolia Elunchunqi, 50445; Inner Mongolia Etukeqi, 53529; Inner Mongolia Eduokeqianqi, 53730; Inner Mongolia Evenkeqi, 50525; Inner Mongolia Erlianhaote, 53068; Inner Mongolia Fuhe, 54024; Inner Mongolia Gangzi, 54214; Inner Mongolia Gaoliban, 54031; Inner Mongolia Guyangxian, 53357; Inner Mongolia Hailisu, 53231; Inner Mongolia Hangjinhouqi , 53420; Inner Mongolia Hangjinqi, 53533; Inner Mongolia Helingeerxian, 53469; Inner Mongolia Huhehaote, 53463; Inner Mongolia Huhehaote suburb, 53466; Inner Mongolia Huade, 53391; Inner Mongolia Houlinguole, 50924; Inner Mongolia Jining, 53480; Inner Mongolia Kalaqinqi, 54313; Inner Mongolia Kailu, 54134; Inner Mongolia Kezuohouqi, 54231; Inner Mongolia Kezuozhongqi, 54047; Inner Mongolia Keshiketengqi, 54117; Inner Mongolia Kulun, 54234; Inner Mongolia Liangcheng, 53475; Inner Mongolia Linxixian, 54115; Inner Mongolia Linhe, 53513; Inner Mongolia Mandula, 53149; Inner Mongolia Manzhouli, 50514; Inner Mongolia Moulidawawoer, 50645; Inner Mongolia Narenbaolige, 53083; Inner Mongolia Neiman, 54223; Inner Mongolia Ningchengxian, 54320; Inner Mongolia Qinglongshan, 54132; Inner Mongolia Qingshuihexian, 53562; Inner Mongolia Shangdu, 53385; Inner Mongolia Shebotu, 54039; Inner Mongolia Siziwang, 53362; Inner Mongolia Suolun, 50834; Inner Mongolia Taibushiqi, 54305; Inner Mongolia Tongliao, 54135; Inner Mongolia Tuquan, 50934; Inner Mongolia Tulihe, 50434; Inner Mongolia Tumutezuoqi, 53464; Inner Mongolia Tuyouqi, 53455; Inner Mongolia Tuoketuoxian, 53467; Inner Mongolia Wengniuteqi, 54213; Inner Mongolia Wuhai, 53512; Inner Mongolia Wulagai, 50913; Inner Mongolia Wulateqianqi, 53433; Inner Mongolia Wulatezhongqi, 53336; Inner Mongolia Wushenqi, 53644; Inner Mongolia Wuyuan, 53337; Inner Mongolia Wuchuanxian, 53368; Inner Mongolia Xiwuzhumuqin, 54012; Inner Mongolia Xilamuren, 53367; Inner Mongolia Xilinhaote, 54102; Inner Mongolia Xianghuangqi, 53289; Inner Mongolia Xiaoergou, 50548; Inner Mongolia Xinbaerhuyouqi, 50603; Inner Mongolia Xinbaerhuzuoqi, 50618; Inner Mongolia Xinghe, 53483; Inner Mongolia Yakeshi, 50526; Inner Mongolia Ejinhuoluoqi, 53545; Inner Mongolia Yikewusu, 53522; Inner Mongolia Zhalantun, 50639; Inner Mongolia Zhalute, 54026; Inner Mongolia Zhenglanqi, 54205; Inner Mongolia Zhengxiangbaiqi, 54204; Inner Mongolia Zhungeerqi, 53553; Inner Mongolia Zhuozi, 53472
	8.	Ningxia Guyuan, 53817; Ningxia Haiyuan, 53806; Ningxia Helan, 53610; Ningxia Huinong, 53519; Ningxia Liupanshan, 53910; Ningxia Maihuangshan, 53727; Ningxia Pingluo, 53611; Ningxia Shitanjing, 53517; Ningxia Taole, 53615; Ningxia Tongxin, 53810; Ningxia Weizhou, 53881; Ningxia Wuzhong, 53612; Ningxia Xiji, 53903; Ningxia Xingren, 53707; Ningxia Yanchi, 53723; Ningxia

	Yongning, 53618; Ningxia Zhongning, 53705; Ningxia Zhongwei, 53704<br>9. Qinghai Dari, 56046; Qinghai Doulan, 52836; Qinghai Gander, 56045; Qinghai Gonghe, 52856; Qinghai Guide, 52868; Qinghai Guinan, 52955; Qinghai Henan, 56065; Qinghai Huzhu, 52863; Qinghai Hualong, 52877; Qinghai Jianzha, 52963; Qinghai Jiuzhi, 56067; Qinghai Maduo, 56033; Qinghai Maqin, 56043; Qinghai Minhe, 52876; Qinghai Nangqian, 56125; Qinghai Nuomuhong, 52825; Qinghai Pingan, 52875; Qinghai Qinghaihu 151, 52854; Qinghai Qingshuihe, 56034; Qinghai Qumacai, 56021; Qinghai Shazhuyu, 52941; Qinghai Tongde, 52957; Qinghai Tongren, 52974; Qinghai Tuotuohe, 56004; Qinghai Wudaoliang, 52908; Qinghai Xining, 52866; Qinghai Xinghai, 52943; Qinghai Xunhua, 52972; Qinghai Yushu, 56029; Qinghai Zaduo, 56018; Qinghai Zeku, 52968; Qinghai Zhiduo, 56016<br>10. Shanxi Anze, 53877; Shanxi Changzi, 53873; Shanxi Datong, 53487; Shanxi Daixian, 53579; Shanxi Dingxiang, 53676; Shanxi Fanshi, 53585; Shanxi Fangshan, 53760; Shanxi Fenxi, 53865; Shanxi Fushan, 53966; Shanxi Gaoping, 53973; Shanxi Gujiao, 53763; Shanxi Guxian, 53874; Shanxi Guangling, 53590; Shanxi Heshun, 53788; Shanxi Hongtong, 53866; Shanxi Hunyuan, 53582; Shanxi Jishan, 53954; Shanxi Jiancaoping, 53677; Shanxi Jiangxian, 53965; Shanxi Jiaokou, 53860; Shanxi Jiexiu, 53863; Shanxi Jingle, 53666; Shanxi Kelan, 53662; Shanxi Lanxian, 53665; Shanxi Lishi, 53764; Shanxi Licheng, 53878; Shanxi Linxian, 53659; Shanxi Lingshi, 53862; Shanxi Lucheng, 53880; Shanxi Ningwu, 53577; Shanxi Pianguan, 53565; Shanxi Pingding, 53687; Shanxi Pinglu, 53574; Shanxi Pingyao, 53778; Shanxi Puxian, 53864; Shanxi Qingshui, 53970; Shanxi Qinxian, 53872; Shanxi Qinyuan, 53875; Shanxi Qingxu, 53774; Shanxi Shanyin, 53576; Shanxi Shenchi, 53575; Shanxi Shouyang, 53780; Shanxi Shuozhou, 53578; Shanxi Taigu, 53775; Shanxi Wutaishan, 53588; Shanxi Wutaixian, 53681; Shanxi Wuzhai, 53663; Shanxi Wuxiang, 53871; Shanxi Xixian, 53853; Shanxi Xiangning, 53953; Shanxi Xiangfen, 53861; Shanxi Xiaodian, 53679; Shanxi Xiaoyi, 53768; Shanxi Xinfu, 53674; Shanxi Xinjiang, 53964; Shanxi Xingxian, 53664; Shanxi Yanhu, 53959; Shanxi Yanggao, 53486; Shanxi Yangquan, 53782; Shanxi Yingxian, 53584; Shanxi Youyu, 53478; Shanxi Yuxian, 53685; Shanxi Yushe, 53787; Shanxi Yuanqu, 53968; Shanxi Yuanping, 53673; Shanxi Zhongyang, 53767; Shanxi Zuoquan, 53786<br>11. Shaanxi Longxian, 57003; Shaanxi Zhidan, 53832<br>12. Sichuan Baiyu, 56147; Sichuan Dege, 56144<br>13. Tibet Basu, 56228; Tibet Bomi, 56227; Tibet Chayu, 56434; Tibet Changdu, 56137; Tibet Dangxiong, 55493; Tibet Dingqing, 56116; Tibet Dingri, 55664; Tibet Gaize, 55248; Tibet Jiangzi, 55680; Tibet Lazi, 55569; Tibet Leiwuqi, 56128; Tibet Linzhi, 56312; Tibet Luolong, 56223; Tibet Miling, 56317; Tibet Muozhugongka, 55593; Tibet Naqu, 55299; Tibet Nanmulin, 55572; Tibet Pulan, 55437; Tibet Shiquanhe, 55228; Tibet Zedang, 55598; Tibet Zuogong, 56331<br>14. Xinjiang Akedala, 51058; Xinjiang Akesu, 51628; Xinjiang Alaer, 51730; Xinjiang Alashankou, 51232; Xinjiang Aletai, 51076; Xinjiang Atushi, 51704; Xinjiang Awati, 51722; Xinjiang Balikun, 52101; Xinjiang Bayinbuluke, 51542; Xinjiang

	Bole, 51238; Xinjiang Buerjin, 51060; Xinjiang Cele, 51826; Xinjiang Changji, 51368; Xinjiang Dabancheng, 51477; Xinjiang Emin, 51145; Xinjiang Fuhai, 51068; Xinjiang Fukang, 51377; Xinjiang Fuyun, 51087; Xinjiang Jiashi, 51707; Xinjiang Habahe, 51053; Xinjiang Hami, 52203; Xinjiang Hebukesaier, 51156; Xinjiang Hetan, 51828; Xinjiang Hongliuhe, 52313; Xinjiang Hutubi, 51367; Xinjiang Jimusaer, 51378; Xinjiang Jinghe, 51334; Xinjiang Kashi, 51709; Xinjiang Kepin, 51720; Xinjiang Kelamayi, 51243; Xinjiang Kuerle, 51656; Xinjiang Kumishi, 51526; Xinjiang Luopu, 51829; Xinjiang Manasi, 51359; Xinjiang Maigaiti, 51810; Xinjiang Miquan, 51369; Xinjiang Minfeng, 51839; Xinjiang Moyu, 51827; Xinjiang Mulei, 51482; Xinjiang Naomaohu, 52112; Xinjiang Pishan, 51818; Xinjiang Qiemuo, 51855; Xinjiang Shawan, 51357; Xinjiang Shaya, 51639; Xinjiang Shashe, 51811; Xinjiang Tacheng, 51133; Xinjiang Tazhong, 51747; Xinjiang Tianchi, 51470; Xinjiang Tianshandaxigou, 51468; Xinjiang Tieqianlike, 51765; Xinjiang Tulufan, 51573; Xinjiang Tulufandongkan, 51572; Xinjiang Tuokexun, 51571; Xinjiang Touli, 51241; Xinjiang Urumqi Pastoral Test Station, 51469; Xinjiang Wuqia, 51705; Xinjiang Wushi, 51627; Xinjiang Xinhe, 51636; Xinjiang Yanqi, 51567; Xinjiang Yecheng, 51814; Xinjiang Yiwu, 52118; Xinjiang Yengjisha, 51802; Xinjiang Yutian, 51931; Xinjiang Yumin, 51137; Xinjiang Yuepuhu, 51717; Xinjiang Zepu, 51815
Cluster B	1. Anhui Bozhou, 58102; Anhui Dangshan, 58015; Anhui Funan, 58202; Anhui Jieshou, 58108; Anhui Tianchang, 58240; Anhui Xiaoxian, 58016<br>2. Beijing, 54511; Beijing Changping, 54499; Beijing Haidian, 54399; Beijing Miyun, 54416; Beijing Miyunshangdianzi, 54421; Beijing Pinggu, 54424; Beijing Shunyi, 54398; Beijing Tongzhou, 54431; Beijing Xiayunling, 54597; Beijing Yanqing, 54406; Beijing Zhaitang, 54501<br>3. Gansu Huixian, 57110; Gansu Liangdang, 57111<br>4. Hebei Anxin, 54605; Hebei Baixiang, 53785; Hebei Botou, 54618; Hebei Cangzhou, 54616; Hebei Caofeidian, 54535; Hebei Changli, 54540; Hebei Chengde, 54423; Hebei Chengdexian, 54430; Hebei Chicheng, 54404; Hebei Chongli, 54304; Hebei Dachang, 54510; Hebei Dacheng, 54613; Hebei Daming, 54804; Hebei Dingzhou, 53696; Hebei Dongguang, 54713; Hebei Fengning, 54308; Hebei Fengfeng, 53894; Hebei Funing, 54541; Hebei Fucheng, 54710; Hebei Fuping, 53690; Hebei Gaobeidian, 54506; Hebei Goyang, 54603; Hebei Guyuan, 54301; Hebei Guan, 54512; Hebei Gucheng, 54707; Hebei Guantao, 54809; Hebei Guangzong, 54631; Hebei Haixing, 54628; Hebei Handan, 53892; Hebei Hejian, 54614; Hebei Hengshui, 54702; Hebei Huailai, 54405; Hebei Huanghua, 54624; Hebei Jize, 54640; Hebei Jinzhou, 53689; Hebei Jingxian, 54711; Hebei Julu, 53799; Hebei Kangbao, 53392; Hebei Kuancheng, 54432; Hebei Laiyuan, 53599; Hebei Leting, 54539; Hebei Linxi, 54801; Hebei Linzhang, 53773; Hebei Lingshou, 53680; Hebei Longhua, 54318; Hebei Longyao, 53794; Hebei Lulong, 54438; Hebei Luancheng, 53789; Hebei Luannan, 54437; Hebei Luanping, 54420; Hebei Ningjin, 53796; Hebei Pingquan, 54319; Hebei Qianan, 54439; Hebei Qianxi, 54434; Hebei Qinhuangdao, 54449; Hebei Qinglong, 54436; Hebei Qingxian, 54615; Hebei Qinghe, 54706; Hebei Quyang, 53682;

Hebei Quzhou, 53893; Hebei Raoyang, 54606; Hebei Renqiu, 54610; Hebei Rongcheng, 54503; Hebei Sanhe, 54520; Hebei Shahe, 53781; Hebei Shenzhou, 54608; Hebei Shunping, 53596; Hebei Tangshan, 54534; Hebei Tangxian, 53692; Hebei Wanquan, 53499; Hebei-Weixian, 54800; Hebei Weichang, 54311; Hebei Weixian, 53896; Hebei Wenan, 54612; Hebei Wuji, 53699; Hebei Wuan, 53890; Hebei Wuqiang, 54700; Hebei Wuyi, 54703; Hebei Xianghe, 54521; Hebei Xinji, 54701; Hebei Xinhe, 54633; Hebei Xinglong, 54425; Hebei Xingtang, 53688; Hebei Xushui, 54601; Hebei Xuanhua, 53498; Hebei Yangyuan, 53492; Hebei Yongqing, 54519; Hebei Yutian, 54522; Hebei Zanhuang, 53795; Hebei Zhangbei, 53399; Hebei Zhangjiakou, 54401; Hebei Zhulu, 54408; Hebei Zhuozhou, 54502; Hebei Zunhua, 54429

5. Henan Changge, 57087; Henan Changheng, 53998; Henan Dancheng, 58100; Henan Dengfeng, 57082; Henan Fanxian, 54903; Henan Fangcheng, 57179; Henan Fengqiu, 53983; Henan Gongyi, 57080; Henan Gushi, 58208; Henan Guangshan, 57299; Henan Huaiyang, 57192; Henan Huangchuan, 58207; Henan Huixian, 53985; Henan Jiyuan, 53978; Henan Jiaxian, 57180; Henan Jiaozuo, 53982; Henan Junxian, 53992; Henan Kaifeng, 57091; Henan Lankao, 57093; Henan Linying, 57183; Henan Lingbao, 57056; Henan Lushan, 57173; Henan Luoning, 57066; Henan Luohe, 57186; Henan Mengjin, 57071; Henan Mengzhou, 57072; Henan Minquan, 58004; Henan Neihuang, 53993; Henan Neixiang, 57169; Henan Nanyang, 57178; Henan Nanzhao, 57176; Henan Pingyu, 57292; Henan Puyang, 54900; Henan Qixian, 53974; Henan Qixian, 57096; Henan Qinyang, 53972; Henan Qingfeng, 54902; Henan Runan, 57197; Henan Ruyang, 57078; Henan Ruzhou, 57075; Henan Sanmenxia, 57051; Henan Shangqiu , 58005; Henan Sheqi, 57187; Henan Mianchi, 57063; Henan Suiping, 57189; Henan Taiqian, 54817; Henan Taikang, 57099; Henan Tangyin, 53991; Henan Wenxian, 57079; Henan Wugang, 57177; Henan Wuyang, 57185; Henan Xihua, 57193; Henan Xiping, 57188; Henan Xixia, 57156; Henan Xixian, 57296; Henan Xiayi, 58017; Henan Xiangcheng, 57182; Henan Xinan, 57070; Henan Xincai, 57293; Henan Xinxiang, 53986; Henan Xinyang, 57297; Henan Xiuwu, 53984; Henan Xuchang, 57089; Henan Yanling, 57095; Henan Yexian, 57184; Henan Yichuan, 57074; Henan Xingyang, 57081; Henan Yucheng, 58006; Henan Echeng, 58007; Henan Zhenping, 57175; Henan Zhengyang, 57295; Henan Zhongmou, 57090; Henan Zhumadian, 57290
6. Hubei Zhuxi, 57249
7. Jiangsu Baoying, 58148; Jiangsu Changshu, 58352; Jiangsu Dafeng, 58158; Jiangsu Danyang, 58341; Jiangsu Donghai, 58036; Jiangsu Dongtai, 58251; Jiangsu Fengxian, 58012; Jiangsu Funing, 58143; Jiangsu Ganyu, 58040; Jiangsu Gaoyou, 58241; Jiangsu Guanyun, 58047; Jiangsu Haian, 58254; Jiangsu Haimen, 58360; Jiangsu Hongze, 58139; Jiangsu Huaian, 58141; Jiangsu Jianhu, 58146; Jiangsu Jiangyan, 58250; Jiangsu Jintan, 58342; Jiangsu Jingjiang, 58257; Jiangsu Jurong, 58344; Jiangsu Kunshan, 58356; Jiangsu Lishui, 58340; Jiangsu Lianyungang, 58044; Jiangsu Lianshui, 58140; Jiangsu Liuhe, 58235; Jiangsu Lvsi, 58265; Jiangsu Nantong, 58259; Jiangsu Pizhou, 58026; Jiangsu Rudong,

	58264; Jiangsu Rugao, 58255; Jiangsu Shuyang, 58038; Jiangsu Sihong, 58135; Jiangsu Siyang, 58132; Jiangsu Suzhou, 58349; Jiangsu Suyu, 58131; Jiangsu Taixing, 58249; Jiangsu Taizhou, 58246; Jiangsu Wuxi, 58354; Jiangsu Xiliandao, 58041; Jiangsu Xiangshui, 58045; Jiangsu Xinghua, 58243; Jiangsu Xuzhou, 58027; Jiangsu Yancheng, 58154; Jiangsu Yangzhong, 58247; Jiangsu Yizheng, 58242; Jiangsu Zhangjiagang, 58353<br>8. Liaoning Jianchang, 54452<br>9. Inner Mongolia Henan, 53732<br>10. Shandong Anqiu, 54844; Shandong Binzhou, 54734; Shandong Cangshan, 58030; Shandong Caoxian, 58002; Shandong Changyi, 54841; Shandong Longdao, 54751; Shandong Chengshantou, 54776; Shandong Chengwu, 58003; Shandong Chiping, 54814; Shandong Dingtao, 54909; Shandong Donge, 54815; Shandong Dongping, 54911; Shandong Feicheng, 54819; Shandong Feixian, 54929; Shandong Fushan, 54764; Shandong Gaomi, 54846; Shandong Gaoqing, 54729; Shandong Heze, 54906; Shandong Huantai, 54833; Shandong Huangdao, 54943; Shandong Jimo, 54855; Shandong Jinan, 54823; Shandong Jiyang, 54821; Shandong Jiaozhou, 54849; Shandong Junan, 54939; Shandong Juye, 54914; Shandong Juancheng, 54904; Shandong Kenli, 54744; Shandong Laiwu, 54828; Shandong Laiyang, 54852; Shandong Laizhou, 54749; Shandong Leling, 54726; Shandong Liangshan, 54910; Shandong Liaocheng, 54806; Shandong Linqing, 54802; Shandong Linshu, 58032; Shandong Linyi, 54938; Shandong Linyi, 54712; Shandong Longkou, 54753; Shandong Mengyin, 54923; Shandong Ningjin, 54716; Shandong Penglai, 54752; Shandong Pingdu, 54842; Shandong Pingyi, 54925; Shandong Qixia, 54759; Shandong Qihe, 54812; Shandong Qingzhou, 54831; Shandong Rizhao, 54945; Shandong Rushan, 54861; Shandong Shanghe, 54724; Shandong Shouguang, 54832; Shandong Sishui, 54920; Shandong Taian, 54827; Shandong Tengzhou, 54927; Shandong Weifang, 54843; Shandong Wendeng, 54777; Shandong Wenshang, 54912; Shandong Wulian, 54940; Shandong Wucheng, 54709; Shandong Xinxian, 54808; Shandong Xintai, 54922; Shandong Xuechen, 58021; Shandong Yantai, 54765; Shandong Yanzhou, 54916; Shandong Yangxin, 54723; Shandong Yishui, 54932; Shandong Yiyuan, 54836; Shandong Yicheng, 58022; Shandong Yutai, 54907; Shandong Yuncheng, 54905; Shandong Zaozhuang, 58024; Shandong Zhangqiu, 54727; Shandong Zhaoyuan, 54755; Shandong Zhucheng, 54848; Shandong Zibo, 54830; Shandong Zoucheng, 54919; Shandong Zouping, 54822<br>11. Shanxi Hejin, 53957; Shanxi Hequ, 53564; Shanxi Jixian, 53859; Shanxi Lingchuan, 53981; Shanxi Liulin, 53753; Shanxi Ruicheng, 57053; Shanxi Shilou, 53759; Shanxi Wanrong, 53956; Shanxi Yonghe, 53852; Shanxi Yongji, 57052<br>12. Shaanxi Ankang, 57245; Shaanxi Ansai, 53841; Shaanxi Baishui, 53941; Shaanxi Baoji, 57016; Shaanxi Chenggu, 57128; Shaanxi Chengcheng, 53949; Shaanxi Dali, 57043; Shaanxi Danfeng, 57153; Shaanxi Dingbian, 53725; Shaanxi Fengxian, 57113; Shaanxi Foping, 57134; Shaanxi Fufeng, 57026; Shaanxi Fugu, 53567; Shaanxi Fuping, 57042; Shaanxi Fuxian, 53931; Shaanxi Ganquan, 53848; Shaanxi Hanyin, 57233; Shaanxi Heyang, 53950; Shaanxi Hengshan,

	53740; Shaanxi Huashan, 57046; Shaanxi Huangling, 53944; Shaanxi Huanglong, 53946; Shaanxi Jiaxian, 53658; Shaanxi Jingbian, 53735; Shaanxi Liquan, 57029; Shaanxi Linyou, 57022; Shaanxi Liuba, 57124; Shaanxi Lueyang, 57106; Shaanxi Luonan, 57057; Shaanxi Meixian, 57027; Shaanxi Mianxian, 57119; Shaanxi Nanzheng, 57213; Shaanxi Ningshan, 57137; Shaanxi Pingli, 57248; Shaanxi Pucheng, 53948; Shaanxi Qianyang, 57021; Shaanxi Qingjian, 53757; Shaanxi Sanyuan, 57041; Shaanxi Shanyang, 57155; Shaanxi Shangnan, 57154; Shaanxi Shangxian, 57143; Shaanxi Shenmu, 53651; Shaanxi Suide, 53754; Shaanxi Tongchuan, 53947; Shaanxi Weinan, 57045; Shaanxi Wubao, 53756; Shaanxi Wuqi, 53738; Shaanxi Wugong, 57034; Shaanxi Xingping, 57038; Shaanxi Xunyang, 57242; Shaanxi Xunyi, 53938; Shaanxi Yanchang, 53854; Shaanxi Yanchuan, 53850; Shaanxi Yaoxian, 57037; Shaanxi Yichuan, 53857; Shaanxi Yongshou, 57030; Shaanxi Yulin, 53646; Shaanxi Zhenba, 57238; Shaanxi Zichang, 53748; Shaanxi Ziyang, 57231; Shaanxi Zhashui, 57140<br>13. Tianjin Baodi, 54525; Tianjin Dagang, 54645; Tianjin Jinnan , 54622; Tianjin Jinghai, 54619; Tianjin Tanggu, 54623; Tianjin Wuqing , 54523<br>14. Tibet Nielamu, 55655<br>15. Yunnan Fugong, 56641; Yunnan Gongshan, 56533
Cluster C	1. Gansu Wenxian, 56192; Gansu Zhouqu, 56094<br>2. Guangdong Lechang, 57988<br>3. Guangxi Guanyang, 57964; Guangxi Ziyuan, 57859<br>4. Guizhou Anshun, 57806; Guizhou Bijie, 57707; Guizhou Cengong, 57735; Guizhou Congjiang, 57936; Guizhou Dafang, 57708; Guizhou Daozhen, 57623; Guizhou Dejiang, 57637; Guizhou Duyun, 57827; Guizhou Dushan, 57922; Guizhou Fenggang, 57723; Guizhou Fuquan, 57821; Guizhou Guanling, 57903; Guizhou Guiding, 57824; Guizhou Hezhang, 56598; Guizhou Huangping, 57822; Guizhou Huishui, 57912; Guizhou Jianhe, 57835; Guizhou Jiangkou, 57736; Guizhou Jinsha, 57714; Guizhou Jinping, 57844; Guizhou Kaiyang, 57719; Guizhou Kaili, 57825; Guizhou Leishan, 57837; Guizhou Liping, 57839; Guizhou Libo, 57926; Guizhou Liuzhi, 57807; Guizhou Longli, 57913; Guizhou Luodian, 57916; Guizhou Meitan, 57722; Guizhou Nayong, 57800; Guizhou Pingba, 57814; Guizhou Pingtang, 57921; Guizhou Puan, 56792; Guizhou Xianxi, 57803; Guizhou Qinglong, 57900; Guizhou Rongjiang, 57932; Guizhou Sandu, 57923; Guizhou Sansui, 57832; Guizhou Shibing, 57737; Guizhou Shiqian, 57734; Guizhou Sinan, 57731; Guizhou Songtao, 57647; Guizhou Taijiang, 57834; Guizhou Tianzhu, 57840; Guizhou Tongzi, 57606; Guizhou Wanshan, 57742; Guizhou Wangmo, 57906; Guizhou Weining, 56691; Guizhou Wengan, 57728; Guizhou Wuchuan, 57634; Guizhou Xifeng, 57718; Guizhou Xiuwen, 57811; Guizhou Yanhe, 57636; Guizhou Yuqing, 57729; Guizhou Yuping, 57739; Guizhou Zhenfeng, 57905; Guizhou Zhengan, 57625; Guizhou Zhijin, 57805; Guizhou Ziyun, 57910; Guizhou Zunyi, 57717<br>5. Henan Jigongshan, 57390; Henan Biyang, 57281; Henan Shangcheng, 58301; Henan Tanghe, 57273; Henan Tongbai, 57285; Henan Xixian, 57396; Henan Xinye, 57271

	6. Hubei Anlu, 57388; Hubei Padang, 57355; Hubei Baokang, 57361; Hubei Caidian, 57489; Hubei Chibi, 57582; Hubei Chongyang, 57586; Hubei Danjiangkou, 57260; Hubei Ezhou, 57496; Hubei Enshi, 57447; Hubei Fangxian, 57259; Hubei Gongan, 57477; Hubei Gucheng, 57268; Hubei Hanchuan, 57486; Hubei Hefeng, 57543; Hubei Hongan, 57398; Hubei Honghu, 57581; Hubei Huangmei, 58409; Hubei Jiayu, 57583; Hubei Jianli, 57573; Hubei Jianshi, 57445; Hubei Jingshan, 57387; Hubei Jingmen, 57377; Hubei Lichuan, 57439; Hubei Luotian, 58401; Hubei Macheng, 57399; Hubei Nanzhang, 57363; Hubei Qichun, 58408; Hubei Qianjiang, 57475; Hubei Shayang, 57484; Hubei Shennongjia, 57362; Hubei Shiyan, 57256; Hubei Shishou, 57571; Hubei Songzi, 57469; Hubei Suizhou, 57381; Hubei Tianmen, 57483; Hubei Tongcheng, 57589; Hubei Tuanfeng, 57495; Hubei Wufeng, 57458; Hubei Wuhan, 57494; Hubei Xiantao, 57485; Hubei Xianfeng, 57540; Hubei Xianning, 57590; Hubei Xiangyang, 57278; Hubei Xiaochang, 57386; Hubei Xiaogan, 57482; Hubei Xinzhou, 57492; Hubei Xingshan, 57359; Hubei Xuanen, 57541; Hubei Yangxin, 58500; Hubei Yiling , 57453; Hubei Yicheng, 57370; Hubei Yingcheng, 57481; Hubei Yingshan, 58402; Hubei Yunxi, 57251; Hubei Yunxian, 57253; Hubei Zaoyang, 57279; Hubei Zhongxiang, 57378; Hubei Zhushan, 57257<br>7. Hunan Anhua, 57669; Hunan Anren, 57881; Hunan Baojing, 57642; Hunan Chaling, 57882; Hunan Changsha, 57687; Hunan Changde, 57662; Hunan Changning, 57874; Hunan Chenzhou, 57972; Hunan Daoxian, 57965; Hunan Dongan, 57867; Hunan Fenghuang, 57740; Hunan Guzhang, 57646; Hunan Guangxi, 57889; Hunan Guiyang, 57973; Hunan Hanshou, 57663; Hunan Hengnan, 57875; Hunan Hengshan, 57777; Hunan Hengyang, 57872; Hunan Hengyangxian, 57871; Hunan Hongjiang, 57754; Hunan Huarong, 57575; Hunan Jishou, 57649; Hunan Jiahe, 57974; Hunan Jianghua, 59063; Hunan Jiangyong, 57969; Hunan Lanshan, 57975; Hunan Lengshuijiang, 57760; Hunan Lengshuitan, 57865; Hunan Lixian, 57565; Hunan Liling, 57781; Hunan Linwu, 57978; Hunan Liuyang, 57688; Hunan Longshan, 57544; Hunan Loudi, 57763; Hunan Luxi, 57657; Hunan Milo, 57680; Hunan Nanxian, 57574; Hunan Nanyue, 57776; Hunan Ningxiang, 57678; Hunan Ningyuan, 57966; Hunan Pingjiang, 57682; Hunan Qidong, 57870; Hunan Qiyang, 57868; Hunan Rucheng, 57985; Hunan Sangzhi, 57554; Hunan Shaoshan, 57771; Hunan Shuangfeng, 57774; Hunan Shuangpai, 57962; Hunan Taojiang, 57666; Hunan Taoyuan, 57661; Hunan Tongtao, 57845; Hunan Xiangxiang, 57772; Hunan Xiangyin, 57673; Hunan Xinhua, 57761; Hunan Xinhuang, 57744; Hunan Xintian, 57971; Hunan Xupu, 57752; Hunan Yizhang, 57976; Hunan Yongshun, 57643; Hunan Yongxing, 57887; Hunan Yongzhou, 57866; Hunan Youxian, 57779; Hunan Yuanjiang, 57671; Hunan Yuanling, 57655; Hunan Yueyang, 57584; Hunan Zhangjiajie, 57558; Hunan Zhijiang, 57745; Hunan Zhuzhou, 57780; Hunan Zixing, 57981<br>8. Jiangxi Lianhua, 57789; Jiangxi Pingxiang, 57786; Jiangxi Shangli, 57783; Jiangxi Tonggu, 57694<br>9. Qinghai Banma, 56151<br>10. Shaanxi Baihe, 57254; Shaanxi Langao, 57247; Shaanxi Ningqiang, 57211;

	Shaanxi Zhenping, 57343<br>11. Sichuan Aba, 56171; Sichuan Batang, 56247; Sichuan Bazhong, 57313; Sichuan Baoxing, 56273; Sichuan Butuo, 56580; Sichuan Changning, 56593; Sichuan Chongzhou, 56181; Sichuan Dachuan, 57328; Sichuan Dazhu, 57420; Sichuan Danba, 56263; Sichuan Daofu, 56167; Sichuan Daocheng, 56357; Sichuan Dechang, 56569; Sichuan Deyang, 56198; Sichuan Dongxing, 57503; Sichuan Dujiangyan, 56188; Sichuan Ebian, 56387; Sichuan Emeishan, 56385; Sichuan Fushun, 56399; Sichuan Ganluo, 56473; Sichuan Ganzi, 56146; Sichuan Gaoping, 57411; Sichuan Guangan, 57415; Sichuan Guangyuan, 57206; Sichuan Hanyuan, 56376; Sichuan Hejiang, 57603; Sichuan Heishui, 56185; Sichuan Hongyuan, 56173; Sichuan Hongya, 56380; Sichuan Huidong, 56675; Sichuan Huili, 56671; Sichuan Jiajiang, 56382; Sichuan Qianwei, 56389; Sichuan Jiange, 57208; Sichuan Jiangan, 57600; Sichuan Jinchuan, 56168; Sichuan Jintang, 56296; Sichuan Jingyan, 56390; Sichuan Jiulong, 56462; Sichuan Jiuzhaigou, 56097; Sichuan Kaijiang, 57329; Sichuan Kangding, 56374; Sichuan Langzhong, 57306; Sichuan Leibo, 56485; Sichuan Litang, 56257; Sichuan Lixian, 56184; Sichuan Linshui, 57416; Sichuan Longquanyi, 56286; Sichuan Longchang, 57507; Sichuan Lushan, 56279; Sichuan Luding, 56371; Sichuan Luhuo, 56158; Sichuan Mabian, 56480; Sichuan Maerkang, 56172; Sichuan Maoxian, 56180; Sichuan Meigu, 56487; Sichuan Miyi, 56670; Sichuan Mianzhu, 56186; Sichuan Mianning, 56474; Sichuan Mingshan, 56280; Sichuan Muli, 56459; Sichuan Muchuan, 56490; Sichuan Nanbu, 57314; Sichuan Nanjiang, 57216; Sichuan Nanxi, 56493; Sichuan Pengshan, 56289; Sichuan Pengzhou, 56189; Sichuan Pingchang, 57324; Sichuan Pujiang, 56281; Sichuan Puge, 56575; Sichuan Qingchuan, 57204; Sichuan Qingshen, 56383; Sichuan Quxian, 57413; Sichuan Rangtang, 56164; Sichuan Renshou, 56297; Sichuan Ruoergai, 56079; Sichuan Seda, 56152; Sichuan Shehong, 57401; Sichuan Shimian, 56378; Sichuan Shiqu, 56038; Sichuan Songpan, 56182; Sichuan Suining, 57405; Sichuan Tongjiang, 57320; Sichuan Wanyuan, 57237; Sichuan Wangcang, 57217; Sichuan Weiyuan, 56395; Sichuan Wenjiang, 56187; Sichuan Wenchuan, 56183; Sichuan Wusheng, 57417; Sichuan Xichang, 56571; Sichuan Xichong, 57309; Sichuan Xide, 56478; Sichuan Xiangcheng, 56443; Sichuan Xiaojin, 56178; Sichuan Xinlong, 56251; Sichuan Xingwen, 56496; Sichuan Xuyong, 57608; Sichuan Yajiang, 56267; Sichuan Yanbian, 56665; Sichuan Yanyuan, 56565; Sichuan Yilong, 57315; Sichuan Yibinxian, 56491; Sichuan Yingjing, 56373; Sichuan Yingshan, 57318; Sichuan Yuexi, 56475; Sichuan Chaojue, 56479; Sichuan Ziyang, 56298; Sichuan Zizhong, 56393; Sichuan Zigong, 56396<br>12. Yunnan Qiaojia, 56673; Yunnan Suijiang, 56483; Yunnan Yiliang, 56594; Yunnan Zhenxiong, 56595<br>13. Chongqing Banan, 57518; Chongqing Beibei, 57511; Chongqing Bishan, 57514; Chongqing Changshou, 57520; Chongqing Chengkou, 57333; Chongqing Dazu, 57502; Chongqing Dianjiang, 57425; Chongqing Fengdu, 57523; Chongqing Jiangjin, 57517; Chongqing Kaixian, 57338; Chongqing Nanchuan, 57519; Chongqing Pengshui, 57537; Chongqing Qijiang, 57612; Chongqing Qianjiang,

	57536; Chongqing Rongchang, 57505; Chongqing Shizhu, 57438; Chongqing Tongliang, 57510; Chongqing Tongnan, 57409; Chongqing Wansheng, 57509; Chongqing Wanzhou, 57432; Chongqing Wushan, 57349; Chongqing Wuxi, 57345; Chongqing Wulong, 57525; Chongqing Yongchuan, 57506; Chongqing Yubei, 57513; Chongqing Yunyang, 57339; Chongqing Zhongxian, 57437
Cluster D	1. Fujian Yongding, 59113; Fujian Zhaoan, 59320<br>2. Guangdong Boluo, 59297; Guangdong Chaozhou, 59312; Guangdong Conghua, 59285; Guangdong Daipu, 59116; Guangdong Dongguan, 59289; Guangdong Doumen, 59487; Guangdong Enping, 59477; Guangdong Panyu, 59481; Guangdong Fengshun, 59310; Guangdong Fogang, 59087; Guangdong Gaozhou, 59653; Guangdong Guangning, 59271; Guangdong Guangzhou, 59287; Guangdong Haifeng, 59500; Guangdong Heping, 59099; Guangdong Heyuan, 59293; Guangdong Heshan, 59473; Guangdong Huadu, 59284; Guangdong Huazhou, 59655; Guangdong Huaiji, 59270; Guangdong Huidong, 59492; Guangdong Jiaoling, 59114; Guangdong Jiexi, 59306; Guangdong Kaiping, 59475; Guangdong Leizhou, 59750; Guangdong Lianping, 59096; Guangdong Lianshan, 59074; Guangdong Lianjiang, 59654; Guangdong Longchuan, 59107; Guangdong Longmen, 59290; Guangdong Lufeng, 59502; Guangdong Luoding, 59462; Guangdong Maoming, 59659; Guangdong Meixian, 59117; Guangdong Nanxiong, 57996; Guangdong Pingyuan, 59106; Guangdong Puning, 59314; Guangdong Qingyuan, 59280; Guangdong Raoping, 59313; Guangdong Renhua, 57989; Guangdong Ruyuan, 59081; Guangdong Sanshui, 59279; Guangdong Shantou, 59316; Guangdong Shaoguan, 59082; Guangdong Shenzhen, 59493; Guangdong Shixing, 59090; Guangdong Sihui, 59276; Guangdong Suixi, 59650; Guangdong Wengyuan, 59094; Guangdong Wuchuan, 59656; Guangdong Wuhua, 59303; Guangdong Xinhui, 59476; Guangdong Xinxing, 59470; Guangdong Xinyi, 59456; Guangdong Xingning, 59109; Guangdong Xuwen, 59754; Guangdong Yangchun, 59469; Guangdong Yangshan, 59075; Guangdong Yingde, 59088; Guangdong Yunan, 59268; Guangdong Yunfu, 59471; Guangdong Zhuhai, 59488<br>3. Guangxi Bama, 59027; Guangxi Baise, 59211; Guangxi Beiliu, 59451; Guangxi Binyang, 59238; Guangxi Bobai, 59449; Guangxi Cangwu, 59266; Guangxi Cenxi, 59454; Guangxi Daxin, 59421; Guangxi Debao, 59215; Guangxi Duan, 59037; Guangxi Fengshan, 59021; Guangxi Hepu, 59640; Guangxi Hechi, 59023; Guangxi Hezhou, 59065; Guangxi Hengxian, 59441; Guangxi Jinxiu, 59057; Guangxi Jingxi, 59218; Guangxi Laibin, 59242; Guangxi Leye, 59012; Guangxi Lipu, 59055; Guangxi Lingui, 57954; Guangxi Lingshan, 59446; Guangxi Lingyun, 59015; Guangxi Liucheng, 59041; Guangxi Longsheng, 57942; Guangxi Longan, 59229; Guangxi Luchuan, 59457; Guangxi Luzhai, 59045; Guangxi Mashan, 59230; Guangxi Mengshan, 59058; Guangxi Nanning, 59431; Guangxi Pingle, 59053; Guangxi Pingnan, 59255; Guangxi Pingxiang, 59419; Guangxi Pubei, 59448; Guangxi Qinzhou, 59632; Guangxi Quanzhou, 57960; Guangxi Rongxian, 59452; Guangxi Rongshui, 57948; Guangxi Sanjiang, 57941; Guangxi Shanglin, 59235; Guangxi Shangsi, 59429; Guangxi Tengxian, 59256; Guangxi Tiandeng,

	59227; Guangxi Tiane, 57927; Guangxi Tiandong, 59224; Guangxi Wuzhou, 59265; Guangxi Wuming , 59237; Guangxi Wuxuan, 59246; Guangxi Xiangzhou, 59241; Guangxi Xincheng, 59038; Guangxi Xingan, 57955; Guangxi Yizhou, 59034; Guangxi Yongning, 59435; Guangxi Yongfu, 57949<br>4. Guizhou Ceheng, 57909<br>5. Hainan Baisha, 59848; Hainan Baoting, 59945; Hainan Changjiang, 59847; Hainan Chengmai, 59843; Hainan Danzhou, 59845; Hainan Dingan, 59851; Hainan Haikou, 59758; Hainan Lingao, 59842; Hainan Qonghai, 59855; Hainan Tunchang, 59854; Hainan Wanning, 59951; Hainan Wenchang, 59856<br>6. Jilin Changbai, 54386<br>7. Jiangxi Dingnan, 59093; Jiangxi Quannan, 59091; Jiangxi Xunwu, 59102<br>8. Yunnan Longchuan, 56835; Yunnan Zhenkang, 56839
Cluster E	1. Anhui Changfeng, 58220; Anhui Chaohu, 58326; Anhui Chuzhou, 58236; Anhui Dingyuan, 58225; Anhui Feidong, 58323; Anhui Feixi, 58320; Anhui Fengtai, 58212; Anhui Fengyang, 58222; Anhui Fuyang, 58203; Anhui Guzhen, 58128; Anhui Guangde, 58441; Anhui Hanshan, 58330; Anhui Huaiyuan, 58127; Anhui Huangshan, 58437; Anhui Huoqiu, 58214; Anhui Huoshan, 58314; Anhui Jinzhai, 58306; Anhui Jingxian, 58432; Anhui Jingde, 58435; Anhui Laian, 58234; Anhui Langxi, 58442; Anhui Leysin, 58117; Anhui Lingbi, 58125; Anhui Luan, 58311; Anhui Lujiang, 58327; Anhui Maanshan, 58336; Anhui Mengcheng, 58118; Anhui Mingguang, 58223; Anhui Nanling, 58431; Anhui Ningguo, 58436; Anhui Shucheng, 58316; Anhui Sixian, 58126; Anhui Susong, 58417; Anhui Suzhou, 58122; Anhui Suixi, 58113; Anhui Taihe, 58109; Anhui Tianzhushan, 58112; Anhui Tongcheng, 58319; Anhui Tongling, 58429; Anhui Woyang, 58114; Anhui Wuwei, 58329; Anhui Wuhu, 58334; Anhui Wuhuxian, 58338; Anhui Wuhe, 58129; Anhui Shexian, 58530; Anhui Xuancheng, 58433; Anhui Yixian, 58523; Anhui Yingshang, 58210; Anhui Yuexi, 58317<br>2. Jiangsu Gaochun, 58339; Jiangsu Jinhu, 58147; Jiangsu Nanjing, 58238; Jiangsu Peixian, 58013; Jiangsu Pukou, 58237; Jiangsu Qidong, 58269; Jiangsu Suining, 58130; Jiangsu Taicang, 58377; Jiangsu Xuyi, 58138; Jiangsu Yixing, 58346<br>3. Shanghai Baoshan, 58362; Shanghai Fengxian, 58463; Shanghai Jinshan, 58460; Shanghai Minhang, 58361<br>4. Zhejiang Anji, 58446; Zhejiang Changxing, 58443; Zhejiang Cixi, 58467; Zhejiang Dachen, 58666; Zhejiang Daishan, 58484; Zhejiang Deqing, 58454; Zhejiang Dongyang, 58558; Zhejiang Fenghua, 58565; Zhejiang Fuyang, 58449; Zhejiang Hangzhou, 58457; Zhejiang Hongjia, 58665; Zhejiang Huzhou, 58450; Zhejiang Jiashan, 58451; Zhejiang Jiaxing, 58452; Zhejiang Jiande, 58544; Zhejiang Jinhua, 58549; Zhejiang Jinyun, 58654; Zhejiang Jingning, 58648; Zhejiang Kaihua, 58537; Zhejiang Leqing, 58656; Zhejiang Lishui, 58646; Zhejiang Linan, 58448; Zhejiang Linhai, 58660; Zhejiang Longquan, 58647; Zhejiang Longyou, 58547; Zhejiang Ninghai, 58567; Zhejiang Panan, 58560; Zhejiang Pingyang, 58751; Zhejiang Pujiang, 58546; Zhejiang Putuo, 58570; Zhejiang Qingtian, 58657; Zhejiang Sanmen, 58568; Zhejiang Shaoxing, 58453; Zhejiang Shengsi, 58472; Zhejiang Shengzhou, 58556; Zhejiang Shipu, 58569; Zhejiang Suichang,

	58644; Zhejiang Tiantai, 58559; Zhejiang Tonglu, 58542; Zhejiang Wenling, 58664; Zhejiang Wuyi, 58642; Zhejiang Xianju, 58652; Zhejiang Xiangshan, 58566; Zhejiang Xinchang, 58555; Zhejiang Yinzhou, 58562; Zhejiang Yongjia, 58658; Zhejiang Yongkang, 58643; Zhejiang Yunhe, 58742; Zhejiang Zhenhai, 58561; Zhejiang Zhuji, 58550
Cluster F	1. Guangxi Tianlin, 59017; Guangxi Xilin, 59004<br>2. Guizhou Panxian, 56793; Guizhou Xingyi, 57907<br>3. Sichuan Derong, 56441; Sichuan Gongxian, 56499; Sichuan Jinyang, 56584; Sichuan Panzhihua, 56666; Sichuan Pingshan, 56494<br>4. Yunnan Anning, 56863; Yunnan Baoshan, 56748; Yunnan Binchuan, 56752; Yunnan Cangyuan, 56944; Yunnan Changning, 56843; Yunnan Chengjiang, 56873; Yunnan Daguan, 56582; Yunnan Dali, 56751; Yunnan Deqin, 56444; Yunnan Dongchuan, 56688; Yunnan Eshan, 56898; Yunnan Eryuan, 56649; Yunnan Fengqing, 56846; Yunnan Fumin, 56772; Yunnan Funing, 59205; Yunnan Fuyuan, 56790; Yunnan Gejiu, 56984; Yunnan Gengma, 56946; Yunnan Guangnan, 59007; Yunnan Heqing, 56654; Yunnan Huaning, 56879; Yunnan Huaping, 56664; Yunnan Jianchuan, 56646; Yunnan Jiangcheng, 56977; Yunnan Jinning, 56871; Yunnan Jingdong, 56856; Yunnan Jinggu, 56952; Yunnan Kaiyuan, 56982; Yunnan Kunming, 56778; Yunnan Lanping, 56645; Yunnan Lancang, 56954; Yunnan Lijiang, 56651; Yunnan Lianghe, 56840; Yunnan Lincang, 56951; Yunnan Liuku, 56643; Yunnan Longling, 56841; Yunnan Luxi, 56886; Yunnan Ludian, 56585; Yunnan Lufeng, 56777; Yunnan Luchun, 56978; Yunnan Luoping, 56891; Yunnan Malong, 56782; Yunnan Menghai, 56958; Yunnan Mengla, 56969; Yunnan Menglian, 56949; Yunnan Midu, 56755; Yunnan Mile, 56885; Yunnan Mojiang, 56962; Yunnan Mouding, 56766; Yunnan Nanhua, 56767; Yunnan Ninglang, 56567; Yunnan Qiubei, 56889; Yunnan Qujing, 56783; Yunnan Shizong, 56883; Yunnan Shidian, 56842; Yunnan Shilin, 56881; Yunnan Shiping, 56970; Yunnan Shuangbai, 56862; Yunnan Shuangjiang, 56950; Yunnan Simao, 56964; Yunnan Songming, 56785; Yunnan Tengchong, 56739; Yunnan Weixin, 56596; Yunnan Weishan, 56757; Yunnan Weixi, 56548; Yunnan Wenshan, 56994; Yunnan Wuding, 56774; Yunnan Xichou, 56992; Yunnan Ximeng, 56948; Yunnan Xianggelila, 56543; Yunnan Xinping, 56869; Yunnan Xuanwei, 56697; Yunnan Yanjin, 56497; Yunnan Yanshan, 56991; Yunnan Yangbi, 56745; Yunnan Yaoan, 56764; Yunnan Yiliang, 56880; Yunnan Yimen, 56870; Yunnan Yingjiang, 56836; Yunnan Yongde, 56849; Yunnan Yongping, 56746; Yunnan Yongren, 56669; Yunnan Yongsheng, 56652; Yunnan Yuxi, 56875; Yunnan Yuanjiang, 56966; Yunnan Yuanyang, 56976; Yunnan Yunlong, 56742; Yunnan Yunxian, 56854; Yunnan Zhenyuan, 56867
Cluster G	1. Anhui Qimen, 58520; Anhui Tunxi, 58531<br>2. Fujian Guangze, 58724; Fujian Ninghuai, 58818<br>3. Hubei Wuxue, 58501<br>4. Jiangxi Anfu, 57798; Jiangxi Anyi, 58602; Jiangxi Anyuan, 58907; Jiangxi Poyang, 58519; Jiangxi Chongren, 58710; Jiangxi Chongyi, 57990; Jiangxi Dean, 58508; Jiangxi Dexing, 58622; Jiangxi Dongxiang, 58618; Jiangxi Duchang, 58517;

	Jiangxi Fenyi, 57792; Jiangxi Fengxin, 58601; Jiangxi Ganxian, 57993; Jiangxi Gaoan, 58605; Jiangxi Guangchang, 58813; Jiangxi Guixi, 58626; Jiangxi Hukou, 58510; Jiangxi Huichang, 58906; Jiangxi Jianxian, 57799; Jiangxi Jinxi, 58712; Jiangxi Jinxian, 58614; Jiangxi Jingdezhen, 58527; Jiangxi Jingan, 58600; Jiangxi Jiujiang, 58502; Jiangxi Lean, 58706; Jiangxi Lichuan, 58719; Jiangxi Linchuan, 58619; Jiangxi Longnan, 59092; Jiangxi Lushan, 58506; Jiangxi Nanchang, 58606; Jiangxi Nancheng, 58715; Jiangxi Nanfeng , 58718; Jiangxi Nankang, 57992; Jiangxi Ningdu, 58806; Jiangxi Pengze, 58512; Jiangxi Qianshan, 58629; Jiangxi Ruichang, 58503; Jiangxi Shanggao, 57699; Jiangxi Shangraoxian, 58623; Jiangxi Shicheng, 58814; Jiangxi Suichuan, 57896; Jiangxi Taihe, 57899; Jiangxi Wanan, 57895; Jiangxi Wannian, 58615; Jiangxi Wanzai, 57698; Jiangxi Wuning, 58507; Jiangxi Wuyuan, 58529; Jiangxi Xiajiang, 58704; Jiangxi Xiaping, 57883; Jiangxi Xinjian, 58693; Jiangxi Xinyu, 57796; Jiangxi Xinfeng, 57995; Jiangxi Xingguo, 58804; Jiangxi Xiushui, 57598; Jiangxi Yichun, 57793; Jiangxi Yongfeng, 58705; Jiangxi Yongxin, 57891; Jiangxi Yudu, 58905; Jiangxi Yugan, 58612; Jiangxi Yushan, 58634<br>5. Zhejiang Changshan, 58631; Zhejiang Jiangshan, 58632
Cluster H	1. Fujian Anxi, 58929; Fujian Changle, 58941; Fujian Changtai, 59122; Fujian Changting, 58911; Fujian Chongwu, 59133; Fujian Datian, 58923; Fujian Fuan, 58748; Fujian Fuding, 58754; Fujian Fuqing, 58942; Fujian Gutian, 58836; Fujian Huaan, 58928; Fujian Jianning, 58822; Fujian Jianyang, 58734; Fujian Jiuxianshan, 58931; Fujian Lianjiang, 58848; Fujian Minhou, 58844; Fujian Shuqing, 58839; Fujian Mingxi, 58824; Fujian Nanan, 59131; Fujian Nanjing, 59124; Fujian Ningde, 58846; Fujian Pinghe, 59125; Fujian Pingtan, 58944; Fujian Putian, 58946; Fujian Pucheng, 58731; Fujian Sanming, 58828; Fujian Shaowu, 58725; Fujian Shouning, 58744; Fujian Shunchang, 58823; Fujian Songxi, 58735; Fujian Taining, 58820; Fujian Tongan, 59130; Fujian Wuping, 58917; Fujian Wuyishan, 58730; Fujian Xiapu, 58843; Fujian Xiuyu, 58938; Fujian Yongtai, 58932; Fujian Youxi, 58837; Fujian Yunxiao, 59322; Fujian Zhangping, 58926; Fujian Zhangpu, 59129; Fujian Zherong, 58749; Fujian Zhenghe, 58736; Fujian Zhouning, 58747<br>2. Guangxi Napo, 59209<br>3. Zhejiang Taishun, 58746; Zhejiang Wencheng, 58750

Table A4. SO_2 emissions from residential sector in 2010

Cluster A	1. Gansu Anding, 52995; Gansu Baiyin, 52896; Gansu Chengxian, 57102; Gansu Dangchang, 56095; Gansu Dibu, 56084; Gansu Dongxiang, 52981; Gansu Gangu, 57001; Gansu Gaolan, 52884; Gansu Gaitai, 52546; Gansu Gulang, 52784; Gansu Guanghe, 52982; Gansu Huishui, 53934; Gansu Hezuo, 56080; Gansu Hezheng, 52985; Gansu Huachi, 53930; Gansu Huajialing, 52996; Gansu Huating, 53927; Gansu Huanxian, 53821; Gansu Huining, 52993; Gansu Jingchuan, 53926; Gansu Jingtai, 52797; Gansu Jingyuan, 52895; Gansu Jingning, 53906; Gansu Jiuquan, 52533; Gansu Kangle, 52988; Gansu Kangxian, 57105; Gansu Kongtong, 53915; Gansu Lixian, 57007; Gansu Lintan, 56081; Gansu Lintao, 52986; Gansu Linze, 52557; Gansu Lingtai, 53924; Gansu Longxi, 56092; Gansu Luqu, 56071; Gansu Mazongshan, 52323; Gansu Maqu, 56074; Gansu Maiji, 57014; Gansu Minle, 52656; Gansu Minqin, 52681; Gansu Minxian, 56093; Gansu Qinan, 57002; Gansu Qingshui, 57011; Gansu Qingcheng, 53829; Gansu Subei, 52515; Gansu Sunan, 52643; Gansu Tianshui, 57006; Gansu Tianzhu, 52881; Gansu Weiyuan, 52998; Gansu Wushaoling, 52787; Gansu Wudu, 56096; Gansu Wushan, 57004; Gansu Wuwei, 52679; Gansu Xifeng, 53923; Gansu Xiahe, 52978; Gansu Yongchang, 52674; Gansu Yongdeng, 52885; Gansu Yongjing, 52980; Gansu Yuzhong, 52983; Gansu Zhangjiachuan, 57012; Gansu Zhangxian, 56091; Gansu Zhenyuan, 53925; Gansu Zhuanglang, 53917; Gansu Zhuoni, 56082<br>2. Hebei Huaian, 53491; Hebei Shangyi, 53397; Hebei Shexian, 53886; Hebei Weixian, 53593<br>3. Henan Linzhou, 53889<br>4. Heilongjiang Acheng, 50958; Heilongjiang Aihui, 50468; Heilongjiang Anda, 50854; Heilongjiang Bayan, 50867; Heilongjiang Baiquan, 50755; Heilongjiang Baoqing, 50888; Heilongjiang Beian, 50656; Heilongjiang Beijicun, 50137; Heilongjiang Beilin, 50853; Heilongjiang Binxian, 50960; Heilongjiang Boli, 50973; Heilongjiang Daqing, 50850; Heilongjiang Dumeng, 50842; Heilongjiang Zhengfang, 50964; Heilongjiang Fuyuan, 50779; Heilongjiang Fujin, 50788; Heilongjiang Fuyu, 50742; Heilongjiang Gannan, 50741; Heilongjiang Haerbin, 50953; Heilongjiang Hailin, 54092; Heilongjiang Hailun, 50756; Heilongjiang Hegang, 50775; Heilongjiang Hulan, 50956; Heilongjiang Huma, 50353; Heilongjiang Huzhong, 50247; Heilongjiang Hulin, 50983; Heilongjiang Huachuan, 50878; Heilongjiang Huanan, 50879; Heilongjiang Jidong, 50987; Heilongjiang Jixi, 50978; Heilongjiang Jiagedaqi, 50442; Heilongjiang Jiamusi, 50873; Heilongjiang Jiayin, 50673; Heilongjiang Kedong, 50659; Heilongjiang Keshan, 50658; Heilongjiang Lansi, 50859; Heilongjiang Lindian, 50749; Heilongjiang Linkou, 50979; Heilongjiang Longjiang, 50739; Heilongjiang Luobei, 50776; Heilongjiang Mishan, 50985; Heilongjiang Minshui, 50758; Heilongjiang Mohe, 50136; Heilongjiang Mudanjiang, 54094; Heilongjiang Mulan, 50962; Heilongjiang Muling, 54093; Heilongjiang Nehe, 50646; Heilongjiang Nenjiang, 50557; Heilongjiang Ningan, 54098; Heilongjiang

	Qitaihe, 50971; Heilongjiang Qiqihaer, 50745; Heilongjiang Qinggang, 50851; Heilongjiang Qingan, 50861; Heilongjiang Raohe, 50892; Heilongjiang Shangzhi, 50968; Heilongjiang Shuangyashan, 50884; Heilongjiang Suibin, 50787; Heilongjiang Suifenhe, 54096; Heilongjiang Suileng, 50767; Heilongjiang Sunwu, 50564; Heilongjiang Tahe, 50246; Heilongjiang Tailai, 50844; Heilongjiang Tangyuan, 50871; Heilongjiang Tieli, 50862; Heilongjiang Tonghe, 50963; Heilongjiang Tongjiang, 50778; Heilongjiang Wangkui, 50852; Heilongjiang Wuyiling, 50674; Heilongjiang Wuchang, 54080; Heilongjiang Wudalianchi, 50655; Heilongjiang Wuying, 50772; Heilongjiang Xinlin, 50349; Heilongjiang Xunke, 50566; Heilongjiang Yanshou, 50965; Heilongjiang Yichun, 50774; Heilongjiang Yian, 50750; Heilongjiang Yilang, 50877; Heilongjiang Zhaodong, 50858; Heilongjiang Zhaozhou, 50950<br>5. Jilin Antu, 54187; Jilin Baicheng, 50936; Jilin Baishan, 54371; Jilin Changchun, 54161; Jilin Changling, 54049; Jilin Daan, 50945; Jilin Dehui, 54065; Jilin Tongfeng, 54261; Jilin Donggang, 54284; Jilin Dunhua, 54186; Jilin Erdao, 54285; Jilin Fuyu, 54063; Jilin Gujiazi, 54155; Jilin Helong, 54286; Jilin Huadian, 54273; Jilin Huichun, 54291; Jilin Huinan, 54274; Jilin Jilin Suburb, 54172; Jilin Jian, 54377; Jilin Jiangyuan, 54279; Jilin Jiaohe, 54181; Jilin Jiutai, 54069; Jilin Lishu, 54154; Jilin Liaoyuan, 54260; Jilin Linjiang, 54374; Jilin Liuhe, 54267; Jilin Longjing, 54290; Jilin Luozigou, 54192; Jilin Nongan, 54064; Jilin Panshi, 54263; Jilin Qianguo, 50949; Jilin Qianan, 50948; Jilin Shulan, 54076; Jilin Shuangliao, 54142; Jilin Shuangyang, 54165; Jilin Songyuan, 50946; Jilin Taonan, 50939; Jilin Tonghua, 54363; Jilin Tonghuaxian, 54362; Jilin Tongyu, 54041; Jilin Wangqing, 54195; Jilin Yantongshan, 54169; Jilin Yanji, 54292; Jilin Yitong, 54164; Jilin Yongji, 54171; Jilin Yushu, 54072; Jilin Zhenlai, 50940<br>6. Liaoning Anshan, 54339; Liaoning Benxi, 54346; Liaoning Caohekou, 54483; Liaoning Changtu, 54243; Liaoning Changhai, 54579; Liaoning Changxingdao, 54565; Liaoning Chaoyang, 54324; Liaoning Dashiqiao, 54475; Liaoning Dandong, 54497; Liaoning Fengcheng, 54494; Liaoning Fushun, 54351; Liaoning Gaizhou, 54474; Liaoning Jianpingxian, 54326; Liaoning Jianpingzhen, 54321; Liaoning Jinzhou, 54568; Liaoning Kangping, 54244; Liaoning Kuandian, 54493; Liaoning Lianshan, 54453; Liaoning Liaoyangxian, 54345; Liaoning Liaozhong, 54332; Liaoning Panshan, 54338; Liaoning Pikou, 54575; Liaoning Pulandian, 54569; Liaoning Qingyuan, 54259; Liaoning Shenbei, 54248; Liaoning Shenyang, 54342; Liaoning Sujiatun, 54340; Liaoning Suizhong, 54454; Liaoning Taian, 54336; Liaoning Tieling, 54249; Liaoning Wafangdian, 54563; Liaoning Xifeng, 54252; Liaoning Xinbin, 54353; Liaoning Xinmin, 54333; Liaoning Xingcheng, 54455; Liaoning Xiuyan, 54486; Liaoning Yangshan, 54325; Liaoning Yingkou, 54471; Liaoning Zhangwu, 54236; Liaoning Zhuanghe, 54584<br>7. Inner Mongolia Abagaqi, 53192; Inner Mongolia Aershan, 50727; Inner Mongolia Alukeerqinqi, 54122; Inner Mongolia Arunqi, 50647; Inner Mongolia Aohanqi, 54225; Inner Mongolia Balihan, 54316; Inner Mongolia Balinyouqi, 54113; Inner Mongolia Balinzuoqi, 54027; Inner Mongolia Bayaertuhushuo, 50928; Inner Mongolia Baotou, 53446; Inner Mongolia Baoguotu, 54226; Inner

	Mongolia Chayouhouqi, 53384; Inner Mongolia Chayouqianqi, 53481; Inner Mongolia Chayouzhongqi, 53378; Inner Mongolia Chifeng, 54218; Inner Mongolia Dalateqi, 53457; Inner Mongolia Damaoqi, 53352; Inner Mongolia Dashetai, 53348; Inner Mongolia Dengkou, 53419; Inner Mongolia Dongsheng, 53543; Inner Mongolia Wuzhumuqindong, 50915; Inner Mongolia Duolunxian, 54208; Inner Mongolia Eerguna, 50425; Inner Mongolia Elunchunqi, 50445; Inner Mongolia Etukeqi, 53529; Inner Mongolia Eduokeqianqi, 53730; Inner Mongolia Evenkeqi, 50525; Inner Mongolia Erlianhaote, 53068; Inner Mongolia Fuhe, 54024; Inner Mongolia Gangzi, 54214; Inner Mongolia Gaoliban, 54031; Inner Mongolia Guyangxian, 53357; Inner Mongolia Hailisu, 53231; Inner Mongolia Hangjinhouqi , 53420; Inner Mongolia Hangjinqi, 53533; Inner Mongolia Helingeerxian, 53469; Inner Mongolia Huhehaote, 53463; Inner Mongolia Huhehaote suburb, 53466; Inner Mongolia Huade, 53391; Inner Mongolia Houlinguole, 50924; Inner Mongolia Jining, 53480; Inner Mongolia Kalaqinqi, 54313; Inner Mongolia Kailu, 54134; Inner Mongolia Kezuohouqi, 54231; Inner Mongolia Kezuozhongqi, 54047; Inner Mongolia Keshiketengqi, 54117; Inner Mongolia Kulun, 54234; Inner Mongolia Liangcheng, 53475; Inner Mongolia Linxixian, 54115; Inner Mongolia Linhe, 53513; Inner Mongolia Mandula, 53149; Inner Mongolia Manzhouli, 50514; Inner Mongolia Moulidawawoer, 50645; Inner Mongolia Narenbaolige, 53083; Inner Mongolia Neiman, 54223; Inner Mongolia Ningchengxian, 54320; Inner Mongolia Qinglongshan, 54132; Inner Mongolia Qingshuihexian, 53562; Inner Mongolia Shangdu, 53385; Inner Mongolia Shebotu, 54039; Inner Mongolia Siziwang, 53362; Inner Mongolia Suolun, 50834; Inner Mongolia Taibushiqi, 54305; Inner Mongolia Tongliao, 54135; Inner Mongolia Tuquan, 50934; Inner Mongolia Tulihe, 50434; Inner Mongolia Tumutezuoqi, 53464; Inner Mongolia Tuyouqi, 53455; Inner Mongolia Tuoketuoxian, 53467; Inner Mongolia Wengniuteqi, 54213; Inner Mongolia Wuhai, 53512; Inner Mongolia Wulagai, 50913; Inner Mongolia Wulateqianqi, 53433; Inner Mongolia Wulatezhongqi, 53336; Inner Mongolia Wushenqi, 53644; Inner Mongolia Wuyuan, 53337; Inner Mongolia Wuchuanxian, 53368; Inner Mongolia Xiwuzhumuqin, 54012; Inner Mongolia Xilamuren, 53367; Inner Mongolia Xilinhaote, 54102; Inner Mongolia Xianghuangqi, 53289; Inner Mongolia Xiaoergou, 50548; Inner Mongolia Xinbaerhuyouqi, 50603; Inner Mongolia Xinbaerhuzuoqi, 50618; Inner Mongolia Xinghe, 53483; Inner Mongolia Yakeshi, 50526; Inner Mongolia Ejinhuoluoqi, 53545; Inner Mongolia Yikewusu, 53522; Inner Mongolia Zhalantun, 50639; Inner Mongolia Zhalute, 54026; Inner Mongolia Zhenglanqi, 54205; Inner Mongolia Zhengxiangbaiqi, 54204; Inner Mongolia Zhungeerqi, 53553; Inner Mongolia Zhuozi, 53472<br>8. Ningxia Guyuan, 53817; Ningxia Haiyuan, 53806; Ningxia Helan, 53610; Ningxia Huinong, 53519; Ningxia Liupanshan, 53910; Ningxia Maihuangshan, 53727; Ningxia Pingluo, 53611; Ningxia Shitanjing, 53517; Ningxia Taole, 53615; Ningxia Tongxin, 53810; Ningxia Weizhou, 53881; Ningxia Wuzhong, 53612; Ningxia Xiji, 53903; Ningxia Xingren, 53707; Ningxia Yanchi, 53723; Ningxia

	Yongning, 53618; Ningxia Zhongning, 53705; Ningxia Zhongwei, 53704<br>9. Qinghai Dari, 56046; Qinghai Doulan, 52836; Qinghai Gander, 56045; Qinghai Gonghe, 52856; Qinghai Guide, 52868; Qinghai Guinan, 52955; Qinghai Henan, 56065; Qinghai Huzhu, 52863; Qinghai Hualong, 52877; Qinghai Jianzha, 52963; Qinghai Jiuzhi, 56067; Qinghai Maduo, 56033; Qinghai Maqin, 56043; Qinghai Minhe, 52876; Qinghai Nangqian, 56125; Qinghai Nuomuhong, 52825; Qinghai Pingan, 52875; Qinghai Qinghaihu 151, 52854; Qinghai Qingshuihe, 56034; Qinghai Qumacai, 56021; Qinghai Shazhuyu, 52941; Qinghai Tongde, 52957; Qinghai Tongren, 52974; Qinghai Tuotuohe, 56004; Qinghai Wudaoliang, 52908; Qinghai Xining, 52866; Qinghai Xinghai, 52943; Qinghai Xunhua, 52972; Qinghai Yushu, 56029; Qinghai Zaduo, 56018; Qinghai Zeku, 52968; Qinghai Zhiduo, 56016<br>10. Shanxi Anze, 53877; Shanxi Changzi, 53873; Shanxi Datong, 53487; Shanxi Daixian, 53579; Shanxi Dingxiang, 53676; Shanxi Fanshi, 53585; Shanxi Fangshan, 53760; Shanxi Fenxi, 53865; Shanxi Fushan, 53966; Shanxi Gaoping, 53973; Shanxi Gujiao, 53763; Shanxi Guxian, 53874; Shanxi Guangling, 53590; Shanxi Heshun, 53788; Shanxi Hongtong, 53866; Shanxi Hunyuan, 53582; Shanxi Jishan, 53954; Shanxi Jiancaoping, 53677; Shanxi Jiangxian, 53965; Shanxi Jiaokou, 53860; Shanxi Jiexiu, 53863; Shanxi Jingle, 53666; Shanxi Kelan, 53662; Shanxi Lanxian, 53665; Shanxi Lishi, 53764; Shanxi Licheng, 53878; Shanxi Linxian, 53659; Shanxi Lingshi, 53862; Shanxi Lucheng, 53880; Shanxi Ningwu, 53577; Shanxi Pianguan, 53565; Shanxi Pingding, 53687; Shanxi Pinglu, 53574; Shanxi Pingyao, 53778; Shanxi Puxian, 53864; Shanxi Qingshui, 53970; Shanxi Qinxian, 53872; Shanxi Qinyuan, 53875; Shanxi Qingxu, 53774; Shanxi Shanyin, 53576; Shanxi Shenchi, 53575; Shanxi Shouyang, 53780; Shanxi Shuozhou, 53578; Shanxi Taigu, 53775; Shanxi Wutaishan, 53588; Shanxi Wutaixian, 53681; Shanxi Wuzhai, 53663; Shanxi Wuxiang, 53871; Shanxi Xixian, 53853; Shanxi Xiangning, 53953; Shanxi Xiangfen, 53861; Shanxi Xiaodian, 53679; Shanxi Xiaoyi, 53768; Shanxi Xinfu, 53674; Shanxi Xinjiang, 53964; Shanxi Xingxian, 53664; Shanxi Yanhu, 53959; Shanxi Yanggao, 53486; Shanxi Yangquan, 53782; Shanxi Yingxian, 53584; Shanxi Youyu, 53478; Shanxi Yuxian, 53685; Shanxi Yushe, 53787; Shanxi Yuanqu, 53968; Shanxi Yuanping, 53673; Shanxi Zhongyang, 53767; Shanxi Zuoquan, 53786<br>11. Shaanxi Zhidan, 53832<br>12. Sichuan Baiyu, 56147; Sichuan Dege, 56144<br>13. Tibet Basu, 56228; Tibet Bomi, 56227; Tibet Chayu, 56434; Tibet Changdu, 56137; Tibet Dangxiong, 55493; Tibet Dingqing, 56116; Tibet Dingri, 55664; Tibet Gaize, 55248; Tibet Jiangzi, 55680; Tibet Lazi, 55569; Tibet Leiwuqi, 56128; Tibet Linzhi, 56312; Tibet Luolong, 56223; Tibet Miling, 56317; Tibet Muozhugongka, 55593; Tibet Naqu, 55299; Tibet Nanmulin, 55572; Tibet Pulan, 55437; Tibet Shiquanhe, 55228; Tibet Zedang, 55598; Tibet Zuogong, 56331<br>14. Xinjiang Akedala, 51058; Xinjiang Akesu, 51628; Xinjiang Alaer, 51730; Xinjiang Alashankou, 51232; Xinjiang Aletai, 51076; Xinjiang Atushi, 51704; Xinjiang Awati, 51722; Xinjiang Balikun, 52101; Xinjiang Bayinbuluke, 51542; Xinjiang

		Bole, 51238; Xinjiang Buerjin, 51060; Xinjiang Cele, 51826; Xinjiang Changji, 51368; Xinjiang Dabancheng, 51477; Xinjiang Emin, 51145; Xinjiang Fuhai, 51068; Xinjiang Fukang, 51377; Xinjiang Fuyun, 51087; Xinjiang Jiashi, 51707; Xinjiang Habahe, 51053; Xinjiang Hami, 52203; Xinjiang Hebukesaier, 51156; Xinjiang Hetan, 51828; Xinjiang Hongliuhe, 52313; Xinjiang Hutubi, 51367; Xinjiang Jimusaer, 51378; Xinjiang Jinghe, 51334; Xinjiang Kashi, 51709; Xinjiang Kepin, 51720; Xinjiang Kelamayi, 51243; Xinjiang Kuerle, 51656; Xinjiang Kumishi, 51526; Xinjiang Luopu, 51829; Xinjiang Manasi, 51359; Xinjiang Maigaiti, 51810; Xinjiang Miquan, 51369; Xinjiang Minfeng, 51839; Xinjiang Moyu, 51827; Xinjiang Mulei, 51482; Xinjiang Naomaohu, 52112; Xinjiang Pishan, 51818; Xinjiang Qiemuo, 51855; Xinjiang Shawan, 51357; Xinjiang Shaya, 51639; Xinjiang Shashe, 51811; Xinjiang Tacheng, 51133; Xinjiang Tazhong, 51747; Xinjiang Tianchi, 51470; Xinjiang Tianshandaxigou, 51468; Xinjiang Tieqianlike, 51765; Xinjiang Tulufan, 51573; Xinjiang Tulufandongkan, 51572; Xinjiang Tuokexun, 51571; Xinjiang Touli, 51241; Xinjiang Urumqi Pastoral Test Station, 51469; Xinjiang Wuqia, 51705; Xinjiang Wushi, 51627; Xinjiang Xinhe, 51636; Xinjiang Yanqi, 51567; Xinjiang Yecheng, 51814; Xinjiang Yiwu, 52118; Xinjiang Yengjisha, 51802; Xinjiang Yutian, 51931; Xinjiang Yumin, 51137; Xinjiang Yuepuhu, 51717; Xinjiang Zepu, 51815
Cluster B	1.	Anhui Bozhou, 58102; Anhui Dangshan, 58015; Anhui Funan, 58202; Anhui Jieshou, 58108; Anhui Tianchang, 58240; Anhui Xiaoxian, 58016
	2.	Beijing, 54511; Beijing Changping, 54499; Beijing Haidian, 54399; Beijing Miyun, 54416; Beijing Miyunshangdianzi, 54421; Beijing Pinggu, 54424; Beijing Shunyi, 54398; Beijing Tongzhou, 54431; Beijing Xiayunling, 54597; Beijing Yanqing, 54406; Beijing Zhaitang, 54501
	3.	Gansu Huixian, 57110; Gansu Liangdang, 57111; Gansu Zhengning, 53935
	4.	Hebei Anxin, 54605; Hebei Baixiang, 53785; Hebei Botou, 54618; Hebei Cangzhou, 54616; Hebei Caofeidian, 54535; Hebei Changli, 54540; Hebei Chengde, 54423; Hebei Chengdexian, 54430; Hebei Chicheng, 54404; Hebei Chongli, 54304; Hebei Dachang, 54510; Hebei Dacheng, 54613; Hebei Daming, 54804; Hebei Dingzhou, 53696; Hebei Dongguang, 54713; Hebei Fengning, 54308; Hebei Fengfeng, 53894; Hebei Funing, 54541; Hebei Fucheng, 54710; Hebei Fuping, 53690; Hebei Gaobeidian, 54506; Hebei Goyang, 54603; Hebei Guyuan, 54301; Hebei Guan, 54512; Hebei Gucheng, 54707; Hebei Guantao, 54809; Hebei Guangzong, 54631; Hebei Haixing, 54628; Hebei Handan, 53892; Hebei Hejian, 54614; Hebei Hengshui, 54702; Hebei Huailai, 54405; Hebei Huanghua, 54624; Hebei Jize, 54640; Hebei Jinzhou, 53689; Hebei Jingxian, 54711; Hebei Julu, 53799; Hebei Kangbao, 53392; Hebei Kuancheng, 54432; Hebei Laiyuan, 53599; Hebei Leting, 54539; Hebei Linxi, 54801; Hebei Linzhang, 53773; Hebei Lingshou, 53680; Hebei Longhua, 54318; Hebei Longyao, 53794; Hebei Lulong, 54438; Hebei Luancheng, 53789; Hebei Luannan, 54437; Hebei Luanping, 54420; Hebei Ningjin, 53796; Hebei Pingquan, 54319; Hebei Qianan, 54439; Hebei Qianxi, 54434; Hebei Qinhuangdao, 54449; Hebei Qinglong, 54436; Hebei Qingxian, 54615; Hebei Qinghe, 54706; Hebei Quyang, 53682;

Hebei Quzhou, 53893; Hebei Raoyang, 54606; Hebei Renqiu, 54610; Hebei Rongcheng, 54503; Hebei Sanhe, 54520; Hebei Shahe, 53781; Hebei Shenzhou, 54608; Hebei Shunping, 53596; Hebei Tangshan, 54534; Hebei Tangxian, 53692; Hebei Wanquan, 53499; Hebei-Weixian, 54800; Hebei Weichang, 54311; Hebei Weixian, 53896; Hebei Wenan, 54612; Hebei Wuji, 53699; Hebei Wuan, 53890; Hebei Wuqiang, 54700; Hebei Wuyi, 54703; Hebei Xianghe, 54521; Hebei Xinji, 54701; Hebei Xinhe, 54633; Hebei Xinglong, 54425; Hebei Xingtang, 53688; Hebei Xushui, 54601; Hebei Xuanhua, 53498; Hebei Yangyuan, 53492; Hebei Yongqing, 54519; Hebei Yutian, 54522; Hebei Zanhuang, 53795; Hebei Zhangbei, 53399; Hebei Zhangjiakou, 54401; Hebei Zhulu, 54408; Hebei Zhuozhou, 54502; Hebei Zunhua, 54429

5. Henan Changge, 57087; Henan Changheng, 53998; Henan Dancheng, 58100; Henan Dengfeng, 57082; Henan Fanxian, 54903; Henan Fangcheng, 57179; Henan Fengqiu, 53983; Henan Gongyi, 57080; Henan Gushi, 58208; Henan Guangshan, 57299; Henan Huaiyang, 57192; Henan Huangchuan, 58207; Henan Huixian, 53985; Henan Jiyuan, 53978; Henan Jiaxian, 57180; Henan Jiaozuo, 53982; Henan Junxian, 53992; Henan Kaifeng, 57091; Henan Lankao, 57093; Henan Linying, 57183; Henan Lingbao, 57056; Henan Lushan, 57173; Henan Luoning, 57066; Henan Luohe, 57186; Henan Mengjin, 57071; Henan Mengzhou, 57072; Henan Minquan, 58004; Henan Neihuang, 53993; Henan Neixiang, 57169; Henan Nanyang, 57178; Henan Nanzhao, 57176; Henan Pingyu, 57292; Henan Puyang, 54900; Henan Qixian, 53974; Henan Qixian, 57096; Henan Qinyang, 53972; Henan Qingfeng, 54902; Henan Runan, 57197; Henan Ruyang, 57078; Henan Ruzhou, 57075; Henan Sanmenxia, 57051; Henan Shangqiu , 58005; Henan Sheqi, 57187; Henan Mianchi, 57063; Henan Suiping, 57189; Henan Taiqian, 54817; Henan Taikang, 57099; Henan Tangyin, 53991; Henan Wenxian, 57079; Henan Wugang, 57177; Henan Wuyang, 57185; Henan Xihua, 57193; Henan Xiping, 57188; Henan Xixia, 57156; Henan Xixian, 57296; Henan Xiayi, 58017; Henan Xiangcheng, 57182; Henan Xinan, 57070; Henan Xincai, 57293; Henan Xinxiang, 53986; Henan Xinyang, 57297; Henan Xiuwu, 53984; Henan Xuchang, 57089; Henan Yanling, 57095; Henan Yexian, 57184; Henan Yichuan, 57074; Henan Xingyang, 57081; Henan Yucheng, 58006; Henan Echeng, 58007; Henan Zhenping, 57175; Henan Zhengyang, 57295; Henan Zhongmou, 57090; Henan Zhumadian, 57290
6. Hubei Zhuxi, 57249
7. Jiangsu Baoying, 58148; Jiangsu Changshu, 58352; Jiangsu Dafeng, 58158; Jiangsu Danyang, 58341; Jiangsu Donghai, 58036; Jiangsu Dongtai, 58251; Jiangsu Funing, 58143; Jiangsu Ganyu, 58040; Jiangsu Gaoyou, 58241; Jiangsu Guanyun, 58047; Jiangsu Haian, 58254; Jiangsu Haimen, 58360; Jiangsu Hongze, 58139; Jiangsu Huaian, 58141; Jiangsu Jianhu, 58146; Jiangsu Jiangyan, 58250; Jiangsu Jintan, 58342; Jiangsu Jingjiang, 58257; Jiangsu Jurong, 58344; Jiangsu Kunshan, 58356; Jiangsu Lishui, 58340; Jiangsu Lianyungang, 58044; Jiangsu Lianshui, 58140; Jiangsu Liuhe, 58235; Jiangsu Lvsi, 58265; Jiangsu Nantong, 58259; Jiangsu Pizhou, 58026; Jiangsu Rudong, 58264; Jiangsu Rugao,

	58255; Jiangsu Shuyang, 58038; Jiangsu Sihong, 58135; Jiangsu Siyang, 58132; Jiangsu Suzhou, 58349; Jiangsu Suyu, 58131; Jiangsu Taixing, 58249; Jiangsu Taizhou, 58246; Jiangsu Wuxi, 58354; Jiangsu Xiliandao, 58041; Jiangsu Xiangshui, 58045; Jiangsu Xinghua, 58243; Jiangsu Xuzhou, 58027; Jiangsu Yancheng, 58154; Jiangsu Yangzhong, 58247; Jiangsu Yizheng, 58242; Jiangsu Zhangjiagang, 58353<br>8. Liaoning Jianchang, 54452; Liaoning Lingyuan, 54327<br>9. Inner Mongolia Henan, 53732<br>10. Shandong Anqiu, 54844; Shandong Binzhou, 54734; Shandong Cangshan, 58030; Shandong Caoxian, 58002; Shandong Changyi, 54841; Shandong Longdao, 54751; Shandong Chengshantou, 54776; Shandong Chengwu, 58003; Shandong Chiping, 54814; Shandong Dingtao, 54909; Shandong Donge, 54815; Shandong Dongping, 54911; Shandong Feicheng, 54819; Shandong Feixian, 54929; Shandong Fushan, 54764; Shandong Gaomi, 54846; Shandong Gaoqing, 54729; Shandong Heze, 54906; Shandong Huantai, 54833; Shandong Huangdao, 54943; Shandong Jimo, 54855; Shandong Jinan, 54823; Shandong Jiyang, 54821; Shandong Jiaozhou, 54849; Shandong Junan, 54939; Shandong Juye, 54914; Shandong Juancheng, 54904; Shandong Kenli, 54744; Shandong Laiwu, 54828; Shandong Laiyang, 54852; Shandong Laizhou, 54749; Shandong Leling, 54726; Shandong Liangshan, 54910; Shandong Liaocheng, 54806; Shandong Linqing, 54802; Shandong Linshu, 58032; Shandong Linyi, 54938; Shandong Linyi, 54712; Shandong Longkou, 54753; Shandong Mengyin, 54923; Shandong Ningjin, 54716; Shandong Penglai, 54752; Shandong Pingdu, 54842; Shandong Pingyi, 54925; Shandong Qixia, 54759; Shandong Qihe, 54812; Shandong Qingzhou, 54831; Shandong Rizhao, 54945; Shandong Rushan, 54861; Shandong Shanghe, 54724; Shandong Shouguang, 54832; Shandong Sishui, 54920; Shandong Taian, 54827; Shandong Tengzhou, 54927; Shandong Weifang, 54843; Shandong Wendeng, 54777; Shandong Wenshang, 54912; Shandong Wulian, 54940; Shandong Wucheng, 54709; Shandong Xinxian, 54808; Shandong Xintai, 54922; Shandong Xuechen, 58021; Shandong Yantai, 54765; Shandong Yanzhou, 54916; Shandong Yangxin, 54723; Shandong Yishui, 54932; Shandong Yiyuan, 54836; Shandong Yicheng, 58022; Shandong Yutai, 54907; Shandong Yuncheng, 54905; Shandong Zaozhuang, 58024; Shandong Zhangqiu, 54727; Shandong Zhaoyuan, 54755; Shandong Zhucheng, 54848; Shandong Zibo, 54830; Shandong Zoucheng, 54919; Shandong Zouping, 54822<br>11. Shanxi Hejin, 53957; Shanxi Hequ, 53564; Shanxi Jixian, 53859; Shanxi Lingchuan, 53981; Shanxi Liulin, 53753; Shanxi Ruicheng, 57053; Shanxi Shilou, 53759; Shanxi Wanrong, 53956; Shanxi Yonghe, 53852; Shanxi Yongji, 57052<br>12. Shaanxi Ankang, 57245; Shaanxi Ansai, 53841; Shaanxi Baishui, 53941; Shaanxi Baoji, 57016; Shaanxi Chenggu, 57128; Shaanxi Chengcheng, 53949; Shaanxi Dali, 57043; Shaanxi Danfeng, 57153; Shaanxi Dingbian, 53725; Shaanxi Fengxian, 57113; Shaanxi Foping, 57134; Shaanxi Fufeng, 57026; Shaanxi Fugu, 53567; Shaanxi Fuping, 57042; Shaanxi Fuxian, 53931; Shaanxi Ganquan, 53848; Shaanxi Hanyin, 57233; Shaanxi Heyang, 53950; Shaanxi Hengshan,

	53740; Shaanxi Huashan, 57046; Shaanxi Huangling, 53944; Shaanxi Huanglong, 53946; Shaanxi Jiaxian, 53658; Shaanxi Jingbian, 53735; Shaanxi Liquan, 57029; Shaanxi Linyou, 57022; Shaanxi Liuba, 57124; Shaanxi Longxian, 57003; Shaanxi Lueyang, 57106; Shaanxi Luonan, 57057; Shaanxi Meixian, 57027; Shaanxi Mianxian, 57119; Shaanxi Nanzheng, 57213; Shaanxi Ningshan, 57137; Shaanxi Pingli, 57248; Shaanxi Pucheng, 53948; Shaanxi Qianyang, 57021; Shaanxi Qingjian, 53757; Shaanxi Sanyuan, 57041; Shaanxi Shanyang, 57155; Shaanxi Shangnan, 57154; Shaanxi Shangxian, 57143; Shaanxi Shenmu, 53651; Shaanxi Suide, 53754; Shaanxi Tongchuan, 53947; Shaanxi Weinan, 57045; Shaanxi Wubao, 53756; Shaanxi Wuqi, 53738; Shaanxi Wugong, 57034; Shaanxi Xingping, 57038; Shaanxi Xunyang, 57242; Shaanxi Xunyi, 53938; Shaanxi Yanchang, 53854; Shaanxi Yanchuan, 53850; Shaanxi Yaoxian, 57037; Shaanxi Yichuan, 53857; Shaanxi Yongshou, 57030; Shaanxi Yulin, 53646; Shaanxi Zhenba, 57238; Shaanxi Zichang, 53748; Shaanxi Ziyang, 57231; Shaanxi Zhashui, 57140<br>13. Tianjin Baodi, 54525; Tianjin Dagang, 54645; Tianjin Jinnan , 54622; Tianjin Jinghai, 54619; Tianjin Tanggu, 54623; Tianjin Wuqing , 54523<br>14. Tibet Nielamu, 55655
Cluster C	1. Gansu Wenxian, 56192; Gansu Zhouqu, 56094<br>2. Guangxi Ziyuan, 57859<br>3. Guizhou Anshun, 57806; Guizhou Bijie, 57707; Guizhou Cengong, 57735; Guizhou Congjiang, 57936; Guizhou Dafang, 57708; Guizhou Daozhen, 57623; Guizhou Dejiang, 57637; Guizhou Duyun, 57827; Guizhou Dushan, 57922; Guizhou Fenggang, 57723; Guizhou Fuquan, 57821; Guizhou Guanling, 57903; Guizhou Guiding, 57824; Guizhou Hezhang, 56598; Guizhou Huangping, 57822; Guizhou Huishui, 57912; Guizhou Jianhe, 57835; Guizhou Jiangkou, 57736; Guizhou Jinsha, 57714; Guizhou Jinping, 57844; Guizhou Kaiyang, 57719; Guizhou Kaili, 57825; Guizhou Leishan, 57837; Guizhou Liping, 57839; Guizhou Libo, 57926; Guizhou Liuzhi, 57807; Guizhou Longli, 57913; Guizhou Luodian, 57916; Guizhou Meitan, 57722; Guizhou Nayong, 57800; Guizhou Pingba, 57814; Guizhou Pingtang, 57921; Guizhou Puan, 56792; Guizhou Xianxi, 57803; Guizhou Qinglong, 57900; Guizhou Rongjiang, 57932; Guizhou Sandu, 57923; Guizhou Sansui, 57832; Guizhou Shibing, 57737; Guizhou Shiqian, 57734; Guizhou Sinan, 57731; Guizhou Songtao, 57647; Guizhou Taijiang, 57834; Guizhou Tianzhu, 57840; Guizhou Tongzi, 57606; Guizhou Wanshan, 57742; Guizhou Wangmo, 57906; Guizhou Weining, 56691; Guizhou Wengan, 57728; Guizhou Wuchuan, 57634; Guizhou Xifeng, 57718; Guizhou Xiuwen, 57811; Guizhou Yanhe, 57636; Guizhou Yuqing, 57729; Guizhou Yuping, 57739; Guizhou Zhenfeng, 57905; Guizhou Zhengan, 57625; Guizhou Zhijin, 57805; Guizhou Ziyun, 57910; Guizhou Zunyi, 57717<br>4. Henan Jigongshan, 57390; Henan Biyang, 57281; Henan Shangcheng, 58301; Henan Tanghe, 57273; Henan Tongbai, 57285; Henan Xixian, 57396; Henan Xinye, 57271<br>5. Hubei Anlu, 57388; Hubei Padang, 57355; Hubei Baokang, 57361; Hubei

	Caidian, 57489; Hubei Chibi, 57582; Hubei Chongyang, 57586; Hubei Danjiangkou, 57260; Hubei Ezhou, 57496; Hubei Enshi, 57447; Hubei Fangxian, 57259; Hubei Gongan, 57477; Hubei Gucheng, 57268; Hubei Hanchuan, 57486; Hubei Hefeng, 57543; Hubei Hongan, 57398; Hubei Honghu, 57581; Hubei Huangmei, 58409; Hubei Jiayu, 57583; Hubei Jianli, 57573; Hubei Jianshi, 57445; Hubei Jingshan, 57387; Hubei Jingmen, 57377; Hubei Lichuan, 57439; Hubei Luotian, 58401; Hubei Macheng, 57399; Hubei Nanzhang, 57363; Hubei Qichun, 58408; Hubei Qianjiang, 57475; Hubei Shayang, 57484; Hubei Shennongjia, 57362; Hubei Shiyan, 57256; Hubei Shishou, 57571; Hubei Songzi, 57469; Hubei Suizhou, 57381; Hubei Tianmen, 57483; Hubei Tongcheng, 57589; Hubei Tuanfeng, 57495; Hubei Wufeng, 57458; Hubei Wuhan, 57494; Hubei Xiantao, 57485; Hubei Xianfeng, 57540; Hubei Xianning, 57590; Hubei Xiangyang, 57278; Hubei Xiaochang, 57386; Hubei Xiaogan, 57482; Hubei Xinzhou, 57492; Hubei Xingshan, 57359; Hubei Xuanen, 57541; Hubei Yangxin, 58500; Hubei Yiling , 57453; Hubei Yicheng, 57370; Hubei Yingcheng, 57481; Hubei Yingshan, 58402; Hubei Yunxi, 57251; Hubei Yunxian, 57253; Hubei Zaoyang, 57279; Hubei Zhongxiang, 57378; Hubei Zhushan, 57257<br>6. Hunan Anhua, 57669; Hunan Anren, 57881; Hunan Baojing, 57642; Hunan Chaling, 57882; Hunan Changsha, 57687; Hunan Changde, 57662; Hunan Changning, 57874; Hunan Chenzhou, 57972; Hunan Daoxian, 57965; Hunan Fenghuang, 57740; Hunan Guzhang, 57646; Hunan Guangxi, 57889; Hunan Guiyang, 57973; Hunan Hanshou, 57663; Hunan Hengnan, 57875; Hunan Hengshan, 57777; Hunan Hengyang, 57872; Hunan Hengyangxian, 57871; Hunan Hongjiang, 57754; Hunan Huarong, 57575; Hunan Jishou, 57649; Hunan Jiahe, 57974; Hunan Jianghua, 59063; Hunan Jiangyong, 57969; Hunan Lanshan, 57975; Hunan Lengshuijiang, 57760; Hunan Lengshuitan, 57865; Hunan Lixian, 57565; Hunan Liling, 57781; Hunan Linwu, 57978; Hunan Liuyang, 57688; Hunan Longshan, 57544; Hunan Loudi, 57763; Hunan Luxi, 57657; Hunan Milo, 57680; Hunan Nanxian, 57574; Hunan Nanyue, 57776; Hunan Ningxiang, 57678; Hunan Ningyuan, 57966; Hunan Pingjiang, 57682; Hunan Qidong, 57870; Hunan Qiyang, 57868; Hunan Rucheng, 57985; Hunan Sangzhi, 57554; Hunan Shaoshan, 57771; Hunan Shuangfeng, 57774; Hunan Shuangpai, 57962; Hunan Taojiang, 57666; Hunan Taoyuan, 57661; Hunan Tongtao, 57845; Hunan Xiangxiang, 57772; Hunan Xiangyin, 57673; Hunan Xinhua, 57761; Hunan Xinhuang, 57744; Hunan Xintian, 57971; Hunan Xupu, 57752; Hunan Yizhang, 57976; Hunan Yongshun, 57643; Hunan Yongxing, 57887; Hunan Yongzhou, 57866; Hunan Youxian, 57779; Hunan Yuanjiang, 57671; Hunan Yuanling, 57655; Hunan Yueyang, 57584; Hunan Zhangjiajie, 57558; Hunan Zhijiang, 57745; Hunan Zhuzhou, 57780; Hunan Zixing, 57981<br>7. Jiangxi Lianhua, 57789; Jiangxi Pingxiang, 57786; Jiangxi Shangli, 57783; Jiangxi Tonggu, 57694<br>8. Qinghai Banma, 56151<br>9. Shaanxi Baihe, 57254; Shaanxi Langao, 57247; Shaanxi Ningqiang, 57211; Shaanxi Zhenping, 57343

	10. Sichuan Aba, 56171; Sichuan Batang, 56247; Sichuan Bazhong, 57313; Sichuan Baoxing, 56273; Sichuan Butuo, 56580; Sichuan Changning, 56593; Sichuan Chongzhou, 56181; Sichuan Dachuan, 57328; Sichuan Dazhu, 57420; Sichuan Danba, 56263; Sichuan Daofu, 56167; Sichuan Daocheng, 56357; Sichuan Dechang, 56569; Sichuan Deyang, 56198; Sichuan Dongxing, 57503; Sichuan Dujiangyan, 56188; Sichuan Ebian, 56387; Sichuan Emeishan, 56385; Sichuan Fushun, 56399; Sichuan Ganluo, 56473; Sichuan Ganzi, 56146; Sichuan Gaoping, 57411; Sichuan Guangan, 57415; Sichuan Guangyuan, 57206; Sichuan Hanyuan, 56376; Sichuan Hejiang, 57603; Sichuan Heishui, 56185; Sichuan Hongyuan, 56173; Sichuan Hongya, 56380; Sichuan Huidong, 56675; Sichuan Huili, 56671; Sichuan Jiajiang, 56382; Sichuan Qianwei, 56389; Sichuan Jiange, 57208; Sichuan Jiangan, 57600; Sichuan Jinchuan, 56168; Sichuan Jintang, 56296; Sichuan Jingyan, 56390; Sichuan Jiulong, 56462; Sichuan Jiuzhaigou, 56097; Sichuan Kaijiang, 57329; Sichuan Kangding, 56374; Sichuan Langzhong, 57306; Sichuan Leibo, 56485; Sichuan Litang, 56257; Sichuan Lixian, 56184; Sichuan Linshui, 57416; Sichuan Longquanyi, 56286; Sichuan Longchang, 57507; Sichuan Lushan, 56279; Sichuan Luding, 56371; Sichuan Luhuo, 56158; Sichuan Mabian, 56480; Sichuan Maerkang, 56172; Sichuan Maoxian, 56180; Sichuan Meigu, 56487; Sichuan Miyi, 56670; Sichuan Mianzhu, 56186; Sichuan Mianning, 56474; Sichuan Mingshan, 56280; Sichuan Muli, 56459; Sichuan Muchuan, 56490; Sichuan Nanbu, 57314; Sichuan Nanjiang, 57216; Sichuan Nanxi, 56493; Sichuan Pengshan, 56289; Sichuan Pengzhou, 56189; Sichuan Pingchang, 57324; Sichuan Pujiang, 56281; Sichuan Puge, 56575; Sichuan Qingchuan, 57204; Sichuan Qingshen, 56383; Sichuan Quxian, 57413; Sichuan Rangtang, 56164; Sichuan Renshou, 56297; Sichuan Ruoergai, 56079; Sichuan Seda, 56152; Sichuan Shehong, 57401; Sichuan Shimian, 56378; Sichuan Shiqu, 56038; Sichuan Songpan, 56182; Sichuan Suining, 57405; Sichuan Tongjiang, 57320; Sichuan Wanyuan, 57237; Sichuan Wangcang, 57217; Sichuan Weiyuan, 56395; Sichuan Wenjiang, 56187; Sichuan Wenchuan, 56183; Sichuan Wusheng, 57417; Sichuan Xichang, 56571; Sichuan Xichong, 57309; Sichuan Xide, 56478; Sichuan Xiangcheng, 56443; Sichuan Xiaojin, 56178; Sichuan Xinlong, 56251; Sichuan Xingwen, 56496; Sichuan Xuyong, 57608; Sichuan Yajiang, 56267; Sichuan Yanbian, 56665; Sichuan Yanyuan, 56565; Sichuan Yilong, 57315; Sichuan Yibinxian, 56491; Sichuan Yingjing, 56373; Sichuan Yingshan, 57318; Sichuan Yuexi, 56475; Sichuan Chaojue, 56479; Sichuan Ziyang, 56298; Sichuan Zizhong, 56393; Sichuan Zigong, 56396<br>11. Yunnan Qiaojia, 56673; Yunnan Suijiang, 56483; Yunnan Yiliang, 56594; Yunnan Zhenxiong, 56595<br>12. Chongqing Banan, 57518; Chongqing Beibei, 57511; Chongqing Bishan, 57514; Chongqing Changshou, 57520; Chongqing Chengkou, 57333; Chongqing Dazu, 57502; Chongqing Dianjiang, 57425; Chongqing Fengdu, 57523; Chongqing Jiangjin, 57517; Chongqing Kaixian, 57338; Chongqing Nanchuan, 57519; Chongqing Pengshui, 57537; Chongqing Qijiang, 57612; Chongqing Qianjiang, 57536; Chongqing Rongchang, 57505; Chongqing Shizhu, 57438; Chongqing

	Tongliang, 57510; Chongqing Tongnan, 57409; Chongqing Wansheng, 57509; Chongqing Wanzhou, 57432; Chongqing Wushan, 57349; Chongqing Wuxi, 57345; Chongqing Wulong, 57525; Chongqing Yongchuan, 57506; Chongqing Yubei, 57513; Chongqing Yunyang, 57339; Chongqing Zhongxian, 57437
Cluster D	1. Fujian Yongding, 59113; Fujian Zhaoan, 59320<br>2. Guangdong Boluo, 59297; Guangdong Chaozhou, 59312; Guangdong Conghua, 59285; Guangdong Daipu, 59116; Guangdong Dongguan, 59289; Guangdong Doumen, 59487; Guangdong Enping, 59477; Guangdong Panyu, 59481; Guangdong Fengshun, 59310; Guangdong Fogang, 59087; Guangdong Gaozhou, 59653; Guangdong Guangning, 59271; Guangdong Guangzhou, 59287; Guangdong Haifeng, 59500; Guangdong Heping, 59099; Guangdong Heyuan, 59293; Guangdong Heshan, 59473; Guangdong Huadu, 59284; Guangdong Huazhou, 59655; Guangdong Huaiji, 59270; Guangdong Huidong, 59492; Guangdong Jiaoling, 59114; Guangdong Jiexi, 59306; Guangdong Kaiping, 59475; Guangdong Leizhou, 59750; Guangdong Lianping, 59096; Guangdong Lianshan, 59074; Guangdong Lianjiang, 59654; Guangdong Longchuan, 59107; Guangdong Longmen, 59290; Guangdong Lufeng, 59502; Guangdong Luoding, 59462; Guangdong Maoming, 59659; Guangdong Meixian, 59117; Guangdong Nanxiong, 57996; Guangdong Pingyuan, 59106; Guangdong Puning, 59314; Guangdong Qingyuan, 59280; Guangdong Raoping, 59313; Guangdong Renhua, 57989; Guangdong Ruyuan, 59081; Guangdong Sanshui, 59279; Guangdong Shantou, 59316; Guangdong Shaoguan, 59082; Guangdong Shenzhen, 59493; Guangdong Shixing, 59090; Guangdong Sihui, 59276; Guangdong Suixi, 59650; Guangdong Wengyuan, 59094; Guangdong Wuchuan, 59656; Guangdong Wuhua, 59303; Guangdong Xinhui, 59476; Guangdong Xinxing, 59470; Guangdong Xinyi, 59456; Guangdong Xingning, 59109; Guangdong Xuwen, 59754; Guangdong Yangchun, 59469; Guangdong Yangshan, 59075; Guangdong Yingde, 59088; Guangdong Yunan, 59268; Guangdong Yunfu, 59471; Guangdong Zhuhai, 59488; Guangdong Zijin, 59304<br>3. Guangxi Bama, 59027; Guangxi Baise, 59211; Guangxi Beiliu, 59451; Guangxi Binyang, 59238; Guangxi Bobai, 59449; Guangxi Cangwu, 59266; Guangxi Cenxi, 59454; Guangxi Daxin, 59421; Guangxi Debao, 59215; Guangxi Duan, 59037; Guangxi Fengshan, 59021; Guangxi Hepu, 59640; Guangxi Hechi, 59023; Guangxi Hezhou, 59065; Guangxi Hengxian, 59441; Guangxi Jinxiu, 59057; Guangxi Jingxi, 59218; Guangxi Laibin, 59242; Guangxi Leye, 59012; Guangxi Lipu, 59055; Guangxi Lingui, 57954; Guangxi Lingshan, 59446; Guangxi Lingyun, 59015; Guangxi Liucheng, 59041; Guangxi Longsheng, 57942; Guangxi Longan, 59229; Guangxi Luchuan, 59457; Guangxi Luzhai, 59045; Guangxi Mashan, 59230; Guangxi Mengshan, 59058; Guangxi Napo, 59209; Guangxi Nanning, 59431; Guangxi Pingle, 59053; Guangxi Pingnan, 59255; Guangxi Pingxiang, 59419; Guangxi Pubei, 59448; Guangxi Qinzhou, 59632; Guangxi Quanzhou, 57960; Guangxi Rongxian, 59452; Guangxi Rongshui, 57948; Guangxi Sanjiang, 57941; Guangxi Shanglin, 59235; Guangxi Shangsi, 59429; Guangxi Tengxian, 59256; Guangxi Tiandeng, 59227; Guangxi Tiane, 57927; Guangxi Tiandong,

	59224; Guangxi Wuzhou, 59265; Guangxi Wuming , 59237; Guangxi Wuxuan, 59246; Guangxi Xiangzhou, 59241; Guangxi Xincheng, 59038; Guangxi Xingan, 57955; Guangxi Yizhou, 59034; Guangxi Yongning, 59435; Guangxi Yongfu, 57949<br>4. Guizhou Ceheng, 57909<br>5. Hainan Baisha, 59848; Hainan Baoting, 59945; Hainan Changjiang, 59847; Hainan Chengmai, 59843; Hainan Danzhou, 59845; Hainan Dingan, 59851; Hainan Haikou, 59758; Hainan Lingao, 59842; Hainan Qonghai, 59855; Hainan Tunchang, 59854; Hainan Wanning, 59951; Hainan Wenchang, 59856<br>6. Jilin Changbai, 54386<br>7. Jiangxi Dingnan, 59093; Jiangxi Quannan, 59091; Jiangxi Xunwu, 59102<br>8. Yunnan Longchuan, 56835; Yunnan Zhenkang, 56839
Cluster E	1. Anhui Changfeng, 58220; Anhui Chaohu, 58326; Anhui Chuzhou, 58236; Anhui Dingyuan, 58225; Anhui Feidong, 58323; Anhui Feixi, 58320; Anhui Fengtai, 58212; Anhui Fengyang, 58222; Anhui Fuyang, 58203; Anhui Guzhen, 58128; Anhui Guangde, 58441; Anhui Hanshan, 58330; Anhui Huaiyuan, 58127; Anhui Huangshan, 58437; Anhui Huoqiu, 58214; Anhui Huoshan, 58314; Anhui Jinzhai, 58306; Anhui Jingxian, 58432; Anhui Jingde, 58435; Anhui Laian, 58234; Anhui Langxi, 58442; Anhui Leysin, 58117; Anhui Lingbi, 58125; Anhui Luan, 58311; Anhui Lujiang, 58327; Anhui Maanshan, 58336; Anhui Mengcheng, 58118; Anhui Mingguang, 58223; Anhui Nanling, 58431; Anhui Ningguo, 58436; Anhui Shucheng, 58316; Anhui Sixian, 58126; Anhui Susong, 58417; Anhui Suzhou, 58122; Anhui Suixi, 58113; Anhui Taihe, 58109; Anhui Tianzhushan, 58112; Anhui Tongcheng, 58319; Anhui Tongling, 58429; Anhui Woyang, 58114; Anhui Wuwei, 58329; Anhui Wuhu, 58334; Anhui Wuhuxian, 58338; Anhui Wuhe, 58129; Anhui Shexian, 58530; Anhui Xuancheng, 58433; Anhui Yixian, 58523; Anhui Yingshang, 58210; Anhui Yuexi, 58317<br>2. Jiangsu Fengxian, 58012; Jiangsu Gaochun, 58339; Jiangsu Jinhu, 58147; Jiangsu Nanjing, 58238; Jiangsu Peixian, 58013; Jiangsu Pukou, 58237; Jiangsu Qidong, 58269; Jiangsu Suining, 58130; Jiangsu Taicang, 58377; Jiangsu Xuyi, 58138; Jiangsu Yixing, 58346<br>3. Shanghai Baoshan, 58362; Shanghai Fengxian, 58463; Shanghai Jinshan, 58460; Shanghai Minhang, 58361<br>4. Zhejiang Anji, 58446; Zhejiang Changxing, 58443; Zhejiang Cixi, 58467; Zhejiang Dachen, 58666; Zhejiang Daishan, 58484; Zhejiang Deqing, 58454; Zhejiang Dongyang, 58558; Zhejiang Fenghua, 58565; Zhejiang Fuyang, 58449; Zhejiang Hangzhou, 58457; Zhejiang Hongjia, 58665; Zhejiang Huzhou, 58450; Zhejiang Jiashan, 58451; Zhejiang Jiaxing, 58452; Zhejiang Jiande, 58544; Zhejiang Jinhua, 58549; Zhejiang Jinyun, 58654; Zhejiang Jingning, 58648; Zhejiang Kaihua, 58537; Zhejiang Leqing, 58656; Zhejiang Lishui, 58646; Zhejiang Linan, 58448; Zhejiang Linhai, 58660; Zhejiang Longquan, 58647; Zhejiang Longyou, 58547; Zhejiang Ninghai, 58567; Zhejiang Panan, 58560; Zhejiang Pingyang, 58751; Zhejiang Pujiang, 58546; Zhejiang Putuo, 58570; Zhejiang Qingtian, 58657; Zhejiang Sanmen, 58568; Zhejiang Shaoxing, 58453; Zhejiang Shengsi,

	58472; Zhejiang Shengzhou, 58556; Zhejiang Shipu, 58569; Zhejiang Suichang, 58644; Zhejiang Tiantai, 58559; Zhejiang Tonglu, 58542; Zhejiang Wenling, 58664; Zhejiang Wuyi, 58642; Zhejiang Xianju, 58652; Zhejiang Xiangshan, 58566; Zhejiang Xinchang, 58555; Zhejiang Yinzhou, 58562; Zhejiang Yongjia, 58658; Zhejiang Yongkang, 58643; Zhejiang Yunhe, 58742; Zhejiang Zhenhai, 58561; Zhejiang Zhuji, 58550
Cluster F	1. Guangxi Tianlin, 59017; Guangxi Xilin, 59004<br>2. Guizhou Panxian, 56793; Guizhou Xingyi, 57907<br>3. Sichuan Derong, 56441; Sichuan Gongxian, 56499; Sichuan Jinyang, 56584; Sichuan Panzhihua, 56666; Sichuan Pingshan, 56494<br>4. Yunnan Anning, 56863; Yunnan Baoshan, 56748; Yunnan Binchuan, 56752; Yunnan Cangyuan, 56944; Yunnan Changning, 56843; Yunnan Chengjiang, 56873; Yunnan Daguan, 56582; Yunnan Dali, 56751; Yunnan Deqin, 56444; Yunnan Dongchuan, 56688; Yunnan Eshan, 56898; Yunnan Eryuan, 56649; Yunnan Fengqing, 56846; Yunnan Fugong, 56641; Yunnan Fumin, 56772; Yunnan Funing, 59205; Yunnan Fuyuan, 56790; Yunnan Gejiu, 56984; Yunnan Gengma, 56946; Yunnan Guangnan, 59007; Yunnan Heqing, 56654; Yunnan Huaning, 56879; Yunnan Huaping, 56664; Yunnan Jianchuan, 56646; Yunnan Jiangcheng, 56977; Yunnan Jinning, 56871; Yunnan Jingdong, 56856; Yunnan Jinggu, 56952; Yunnan Kaiyuan, 56982; Yunnan Kunming, 56778; Yunnan Lanping, 56645; Yunnan Lancang, 56954; Yunnan Lijiang, 56651; Yunnan Lianghe, 56840; Yunnan Lincang, 56951; Yunnan Liuku, 56643; Yunnan Longling, 56841; Yunnan Luxi, 56886; Yunnan Ludian, 56585; Yunnan Lufeng, 56777; Yunnan Luchun, 56978; Yunnan Luoping, 56891; Yunnan Malong, 56782; Yunnan Menghai, 56958; Yunnan Mengla, 56969; Yunnan Menglian, 56949; Yunnan Midu, 56755; Yunnan Mile, 56885; Yunnan Mojiang, 56962; Yunnan Mouding, 56766; Yunnan Nanhua, 56767; Yunnan Ninglang, 56567; Yunnan Qiubei, 56889; Yunnan Qujing, 56783; Yunnan Shizong, 56883; Yunnan Shidian, 56842; Yunnan Shilin, 56881; Yunnan Shiping, 56970; Yunnan Shuangbai, 56862; Yunnan Shuangjiang, 56950; Yunnan Simao, 56964; Yunnan Songming, 56785; Yunnan Tengchong, 56739; Yunnan Weixin, 56596; Yunnan Weishan, 56757; Yunnan Weixi, 56548; Yunnan Wenshan, 56994; Yunnan Wuding, 56774; Yunnan Xichou, 56992; Yunnan Ximeng, 56948; Yunnan Xianggelila, 56543; Yunnan Xinping, 56869; Yunnan Xuanwei, 56697; Yunnan Yanjin, 56497; Yunnan Yanshan, 56991; Yunnan Yangbi, 56745; Yunnan Yaoan, 56764; Yunnan Yiliang, 56880; Yunnan Yimen, 56870; Yunnan Yingjiang, 56836; Yunnan Yongde, 56849; Yunnan Yongping, 56746; Yunnan Yongren, 56669; Yunnan Yongsheng, 56652; Yunnan Yuxi, 56875; Yunnan Yuanjiang, 56966; Yunnan Yuanyang, 56976; Yunnan Yunlong, 56742; Yunnan Yunxian, 56854; Yunnan Zhenyuan, 56867
Cluster G	1. Anhui Qimen, 58520; Anhui Tunxi, 58531<br>2. Fujian Guangze, 58724; Fujian Ninghuai, 58818<br>3. Hubei Wuxue, 58501<br>4. Jiangxi Anfu, 57798; Jiangxi Anyi, 58602; Jiangxi Anyuan, 58907; Jiangxi Poyang,

	58519; Jiangxi Chongren, 58710; Jiangxi Chongyi, 57990; Jiangxi Dean, 58508; Jiangxi Dexing, 58622; Jiangxi Dongxiang, 58618; Jiangxi Duchang, 58517; Jiangxi Fenyi, 57792; Jiangxi Fengxin, 58601; Jiangxi Ganxian, 57993; Jiangxi Gaoan, 58605; Jiangxi Guangchang, 58813; Jiangxi Guixi, 58626; Jiangxi Hukou, 58510; Jiangxi Huichang, 58906; Jiangxi Jianxian, 57799; Jiangxi Jinxi, 58712; Jiangxi Jinxian, 58614; Jiangxi Jingdezhen, 58527; Jiangxi Jingan, 58600; Jiangxi Jiujiang, 58502; Jiangxi Lean, 58706; Jiangxi Lichuan, 58719; Jiangxi Linchuan, 58619; Jiangxi Longnan, 59092; Jiangxi Lushan, 58506; Jiangxi Nanchang, 58606; Jiangxi Nancheng, 58715; Jiangxi Nanfeng , 58718; Jiangxi Nankang, 57992; Jiangxi Ningdu, 58806; Jiangxi Pengze, 58512; Jiangxi Qianshan, 58629; Jiangxi Ruichang, 58503; Jiangxi Shanggao, 57699; Jiangxi Shangraoxian, 58623; Jiangxi Shicheng, 58814; Jiangxi Suichuan, 57896; Jiangxi Taihe, 57899; Jiangxi Wanan, 57895; Jiangxi Wannian, 58615; Jiangxi Wanzai, 57698; Jiangxi Wuning, 58507; Jiangxi Wuyuan, 58529; Jiangxi Xiajiang, 58704; Jiangxi Xiaping, 57883; Jiangxi Xinjian, 58693; Jiangxi Xinyu, 57796; Jiangxi Xinfeng, 57995; Jiangxi Xingguo, 58804; Jiangxi Xiushui, 57598; Jiangxi Yichun, 57793; Jiangxi Yongfeng, 58705; Jiangxi Yongxin, 57891; Jiangxi Yudu, 58905; Jiangxi Yugan, 58612; Jiangxi Yushan, 58634<br>5. Zhejiang Changshan, 58631; Zhejiang Jiangshan, 58632
Cluster H	1. Fujian Anxi, 58929; Fujian Changle, 58941; Fujian Changtai, 59122; Fujian Changting, 58911; Fujian Chongwu, 59133; Fujian Datian, 58923; Fujian Fuan, 58748; Fujian Fuding, 58754; Fujian Fuqing, 58942; Fujian Gutian, 58836; Fujian Huaan, 58928; Fujian Jianning, 58822; Fujian Jianyang, 58734; Fujian Jiuxianshan, 58931; Fujian Lianjiang, 58848; Fujian Minhou, 58844; Fujian Shuqing, 58839; Fujian Mingxi, 58824; Fujian Nanan, 59131; Fujian Nanjing, 59124; Fujian Ningde, 58846; Fujian Pinghe, 59125; Fujian Pingtan, 58944; Fujian Putian, 58946; Fujian Pucheng, 58731; Fujian Sanming, 58828; Fujian Shaowu, 58725; Fujian Shouning, 58744; Fujian Shunchang, 58823; Fujian Songxi, 58735; Fujian Taining, 58820; Fujian Tongan, 59130; Fujian Wuping, 58917; Fujian Wuyishan, 58730; Fujian Xiapu, 58843; Fujian Xiuyu, 58938; Fujian Yongtai, 58932; Fujian Youxi, 58837; Fujian Yunxiao, 59322; Fujian Zhangping, 58926; Fujian Zhangpu, 59129; Fujian Zherong, 58749; Fujian Zhenghe, 58736; Fujian Zhouning, 58747<br>2. Zhejiang Taishun, 58746; Zhejiang Wencheng, 58750

Table A5. SO_2 emissions from transportation sector in 2008

Cluster A	1.	Beijing Yanqing, 54406; Beijing Xiayunling, 54597
	2.	Gansu Gaitai, 52546; Gansu Linze, 52557; Gansu Minle, 52656; Gansu Yongchang, 52674; Gansu Wuwei, 52679; Gansu Gulang, 52784; Gansu Wushaoling, 52787; Gansu Jingtai, 52797; Gansu Tianzhu, 52881; Gansu Gaolan, 52884; Gansu Yongdeng, 52885; Gansu Jingyuan, 52895; Gansu Baiyin, 52896; Gansu Xiahe, 52978; Gansu Yongjing, 52980; Gansu Dongxiang, 52981; Gansu Guanghe, 52982; Gansu Yuzhong, 52983; Gansu Hezheng, 52985; Gansu Lintao, 52986; Gansu Kangle, 52988; Gansu Huining, 52993; Gansu Anding, 52995; Gansu Huajialing, 52996; Gansu Weiyuan, 52998; Gansu Huanxian, 53821; Gansu Qingcheng, 53829; Gansu Jingning, 53906; Gansu Kongtong, 53915; Gansu Zhuanglang, 53917; Gansu Xifeng, 53923; Gansu Lingtai, 53924; Gansu Zhenyuan, 53925; Gansu Jingchuan, 53926; Gansu Huating, 53927; Gansu Huachi, 53930; Gansu Huishui, 53934; Gansu Zhengning, 53935; Gansu Luqu, 56071; Gansu Maqu, 56074; Gansu Hezuo, 56080; Gansu Lintan, 56081; Gansu Dibu, 56084; Gansu Zhangxian, 56091; Gansu Longxi, 56092; Gansu Minxian, 56093; Gansu Zhouqu, 56094; Gansu Dangchang, 56095; Gansu Wudu, 56096; Gansu Wenxian, 56192; Gansu Gangu, 57001; Gansu Qinan, 57002; Gansu Wushan, 57004; Gansu Tianshui, 57006; Gansu Lixian, 57007; Gansu Qingshui, 57011; Gansu Zhangjiachuan, 57012; Gansu Maiji, 57014; Gansu Chengxian, 57102; Gansu Kangxian, 57105; Gansu Huixian, 57110; Gansu Liangdang, 57111
	3.	Hebei Kangbao, 53392; Hebei Zhangbei, 53399; Hebei Huaian, 53491; Hebei Yangyuan, 53492; Hebei Xuanhua, 53498; Hebei Wanquan, 53499; Hebei Weixian, 53593; Hebei Shunping, 53596; Hebei Laiyuan, 53599; Hebei Lingshou, 53680; Hebei Quyang, 53682; Hebei Xingtang, 53688; Hebei Jinzhou, 53689; Hebei Fuping, 53690; Hebei Tangxian, 53692; Hebei Dingzhou, 53696; Hebei Wuji, 53699; Hebei Shahe, 53781; Hebei Baixiang, 53785; Hebei Luancheng, 53789; Hebei Longyao, 53794; Hebei Zanhuang, 53795; Hebei Ningjin, 53796; Hebei Julu, 53799; Hebei Shexian, 53886; Hebei Wuan, 53890; Hebei Handan, 53892; Hebei Quzhou, 53893; Hebei Guyuan, 54301; Hebei Chongli, 54304; Hebei Fengning, 54308; Hebei Weichang, 54311; Hebei Longhua, 54318; Hebei Pingquan, 54319; Hebei Zhangjiakou, 54401; Hebei Chicheng, 54404; Hebei Zhulu, 54408; Hebei Chengde, 54423; Hebei Zunhua, 54429; Hebei Chengdexian, 54430; Hebei Kuancheng, 54432; Hebei Qianxi, 54434; Hebei Qinglong, 54436; Hebei Luannan, 54437; Hebei Lulong, 54438; Hebei Qianan, 54439; Hebei Qinhuangdao, 54449; Hebei Zhuozhou, 54502; Hebei Rongcheng, 54503; Hebei Gaobeidian, 54506; Hebei Guan, 54512; Hebei Yongqing, 54519; Hebei Sanhe, 54520; Hebei Xianghe, 54521; Hebei Yutian, 54522; Hebei Tangshan, 54534; Hebei Caofeidian, 54535; Hebei Leting, 54539; Hebei Changli, 54540; Hebei Funing, 54541; Hebei Xushui, 54601; Hebei Goyang, 54603; Hebei Anxin, 54605; Hebei Raoyang, 54606; Hebei Shenzhou, 54608; Hebei Renqiu, 54610; Hebei Wenan, 54612; Hebei Dacheng, 54613;

	Hebei Hejian, 54614; Hebei Qingxian, 54615; Hebei Cangzhou, 54616; Hebei Botou, 54618; Hebei Huanghua, 54624; Hebei Haixing, 54628; Hebei Guangzong, 54631; Hebei Xinhe, 54633; Hebei Jize, 54640; Hebei Wuqiang, 54700; Hebei Xinji, 54701; Hebei Hengshui, 54702; Hebei Wuyi, 54703; Hebei Qinghe, 54706; Hebei Gucheng, 54707; Hebei Fucheng, 54710; Hebei Jingxian, 54711; Hebei Dongguang, 54713; Hebei-Weixian, 54800; Hebei Linxi, 54801; Hebei Guantao, 54809<br>4. Henan Linzhou, 53889; Henan Taiqian, 54817; Henan Puyang, 54900<br>5. Heilongjiang Tailai, 50844; Heilongjiang Zhaozhou, 50950<br>6. Hubei Zhuxi, 57249<br>7. Jilin Baicheng, 50936; Jilin Taonan, 50939; Jilin Zhenlai, 50940; Jilin Daan, 50945; Jilin Songyuan, 50946; Jilin Qianan, 50948; Jilin Qianguo, 50949; Jilin Tongyu, 54041; Jilin Changling, 54049; Jilin Fuyu, 54063; Jilin Nongan, 54064; Jilin Dehui, 54065; Jilin Jiutai, 54069; Jilin Yushu, 54072; Jilin Shulan, 54076; Jilin Lishu, 54154; Jilin Gujiazi, 54155; Jilin Changchun, 54161; Jilin Yitong, 54164; Jilin Shuangyang, 54165; Jilin Yantongshan, 54169; Jilin Yongji, 54171; Jilin Jilin Suburb, 54172; Jilin Jiaohe, 54181; Jilin Dunhua, 54186; Jilin Antu, 54187; Jilin Luozigou, 54192; Jilin Wangqing, 54195; Jilin Liaoyuan, 54260; Jilin Tongfeng, 54261; Jilin Panshi, 54263; Jilin Liuhe, 54267; Jilin Huadian, 54273; Jilin Huinan, 54274; Jilin Jiangyuan, 54279; Jilin Donggang, 54284; Jilin Erdao, 54285; Jilin Helong, 54286; Jilin Yanji, 54292; Jilin Tonghuaxian, 54362; Jilin Tonghua, 54363; Jilin Baishan, 54371; Jilin Jian, 54377<br>8. Liaoning Zhangwu, 54236; Liaoning Changtu, 54243; Liaoning Kangping, 54244; Liaoning Shenbei, 54248; Liaoning Tieling, 54249; Liaoning Xifeng, 54252; Liaoning Qingyuan, 54259; Liaoning Jianpingzhen, 54321; Liaoning Chaoyang, 54324; Liaoning Yangshan, 54325; Liaoning Jianpingxian, 54326; Liaoning Lingyuan, 54327; Liaoning Liaozhong, 54332; Liaoning Xinmin, 54333; Liaoning Taian, 54336; Liaoning Panshan, 54338; Liaoning Anshan, 54339; Liaoning Sujiatun, 54340; Liaoning Shenyang, 54342; Liaoning Liaoyangxian, 54345; Liaoning Benxi, 54346; Liaoning Fushun, 54351; Liaoning Xinbin, 54353; Liaoning Jianchang, 54452; Liaoning Lianshan, 54453; Liaoning Suizhong, 54454; Liaoning Xingcheng, 54455; Liaoning Yingkou, 54471; Liaoning Gaizhou, 54474; Liaoning Dashiqiao, 54475; Liaoning Caohekou, 54483; Liaoning Xiuyan, 54486; Liaoning Kuandian, 54493; Liaoning Fengcheng, 54494; Liaoning Dandong, 54497; Liaoning Wafangdian, 54563; Liaoning Jinzhou, 54568; Liaoning Pulandian, 54569; Liaoning Pikou, 54575; Liaoning Changhai, 54579; Liaoning Zhuanghe, 54584; Liaoning Changxingdao, 54565<br>9. Inner Mongolia Chayouqianqi, 53481; Inner Mongolia Qingshuihexian, 53562; Inner Mongolia Henan, 53732; Inner Mongolia Qinglongshan, 54132; Inner Mongolia Zhengxiangbaiqi, 54204; Inner Mongolia Aohanqi, 54225; Inner Mongolia Kezuohouqi, 54231; Inner Mongolia Kulun, 54234; Inner Mongolia Taibushiqi, 54305; Inner Mongolia Balihan, 54316; Inner Mongolia Ningchengxian, 54320<br>10. Ningxia Huinong, 53519; Ningxia Helan, 53610; Ningxia Pingluo, 53611; Ningxia

	Wuzhong, 53612; Ningxia Taole, 53615; Ningxia Yongning, 53618; Ningxia Zhongwei, 53704; Ningxia Zhongning, 53705; Ningxia Xingren, 53707; Ningxia Yanchi, 53723; Ningxia Maihuangshan, 53727; Ningxia Haiyuan, 53806; Ningxia Tongxin, 53810; Ningxia Guyuan, 53817; Ningxia Weizhou, 53881; Ningxia Xiji, 53903; Ningxia Liupanshan, 53910<br>11. Qinghai Banma, 56151<br>12. Shandong Wucheng, 54709; Shandong Linyi, 54712; Shandong Ningjin, 54716; Shandong Yangxin, 54723; Shandong Shanghe, 54724; Shandong Leling, 54726; Shandong Zhangqiu, 54727; Shandong Gaoqing, 54729; Shandong Binzhou, 54734; Shandong Kenli, 54744; Shandong Laizhou, 54749; Shandong Longdao, 54751; Shandong Penglai, 54752; Shandong Longkou, 54753; Shandong Zhaoyuan, 54755; Shandong Qixia, 54759; Shandong Fushan, 54764; Shandong Yantai, 54765; Shandong Chengshantou, 54776; Shandong Wendeng, 54777; Shandong Linqing, 54802; Shandong Liaocheng, 54806; Shandong Qihe, 54812; Shandong Chiping, 54814; Shandong Donge, 54815; Shandong Feicheng, 54819; Shandong Jiyang, 54821; Shandong Zouping, 54822; Shandong Jinan, 54823; Shandong Taian, 54827; Shandong Laiwu, 54828; Shandong Zibo, 54830; Shandong Qingzhou, 54831; Shandong Shouguang, 54832; Shandong Huantai, 54833; Shandong Yiyuan, 54836; Shandong Changyi, 54841; Shandong Pingdu, 54842; Shandong Weifang, 54843; Shandong Anqiu, 54844; Shandong Gaomi, 54846; Shandong Zhucheng, 54848; Shandong Jiaozhou, 54849; Shandong Laiyang, 54852; Shandong Jimo, 54855; Shandong Rushan, 54861; Shandong Juancheng, 54904; Shandong Yuncheng, 54905; Shandong Heze, 54906; Shandong Dingtao, 54909; Shandong Liangshan, 54910; Shandong Dongping, 54911; Shandong Wenshang, 54912; Shandong Juye, 54914; Shandong Yanzhou, 54916; Shandong Zoucheng, 54919; Shandong Sishui, 54920; Shandong Xintai, 54922; Shandong Mengyin, 54923; Shandong Pingyi, 54925; Shandong Tengzhou, 54927; Shandong Feixian, 54929; Shandong Yishui, 54932; Shandong Linyi, 54938; Shandong Junan, 54939; Shandong Wulian, 54940; Shandong Huangdao, 54943; Shandong Rizhao, 54945; Shandong Zaozhuang, 58024; Shandong Cangshan, 58030; Shandong Linshu, 58032<br>13. Shanxi Youyu, 53478; Shanxi Yanggao, 53486; Shanxi Datong, 53487; Shanxi Hequ, 53564; Shanxi Pianguan, 53565; Shanxi Pinglu, 53574; Shanxi Shenchi, 53575; Shanxi Shanyin, 53576; Shanxi Ningwu, 53577; Shanxi Shuozhou, 53578; Shanxi Daixian, 53579; Shanxi Hunyuan, 53582; Shanxi Yingxian, 53584; Shanxi Fanshi, 53585; Shanxi Wutaishan, 53588; Shanxi Guangling, 53590; Shanxi Linxian, 53659; Shanxi Kelan, 53662; Shanxi Wuzhai, 53663; Shanxi Xingxian, 53664; Shanxi Lanxian, 53665; Shanxi Jingle, 53666; Shanxi Yuanping, 53673; Shanxi Xinfu, 53674; Shanxi Dingxiang, 53676; Shanxi Jiancaoping, 53677; Shanxi Xiaodian, 53679; Shanxi Wutaixian, 53681; Shanxi Yuxian, 53685; Shanxi Pingding, 53687; Shanxi Liulin, 53753; Shanxi Shilou, 53759; Shanxi Fangshan, 53760; Shanxi Gujiao, 53763; Shanxi Lishi, 53764; Shanxi Zhongyang, 53767; Shanxi Xiaoyi, 53768; Shanxi Qingxu, 53774; Shanxi Taigu, 53775; Shanxi Pingyao, 53778; Shanxi Shouyang, 53780; Shanxi Yangquan, 53782; Shanxi

	Zuoquan, 53786; Shanxi Yushe, 53787; Shanxi Heshun, 53788; Shanxi Yonghe, 53852; Shanxi Xixian, 53853; Shanxi Jixian, 53859; Shanxi Jiaokou, 53860; Shanxi Xiangfen, 53861; Shanxi Lingshi, 53862; Shanxi Jiexiu, 53863; Shanxi Puxian, 53864; Shanxi Fenxi, 53865; Shanxi Hongtong, 53866; Shanxi Wuxiang, 53871; Shanxi Qinxian, 53872; Shanxi Changzi, 53873; Shanxi Guxian, 53874; Shanxi Qinyuan, 53875; Shanxi Anze, 53877; Shanxi Licheng, 53878; Shanxi Lucheng, 53880; Shanxi Xiangning, 53953; Shanxi Jishan, 53954; Shanxi Wanrong, 53956; Shanxi Hejin, 53957; Shanxi Yanhu, 53959; Shanxi Xinjiang, 53964; Shanxi Jiangxian, 53965; Shanxi Fushan, 53966; Shanxi Yuanqu, 53968; Shanxi Qingshui, 53970; Shanxi Gaoping, 53973; Shanxi Yongji, 57052; Shanxi Ruicheng, 57053<br>14. Shaanxi Fugu, 53567; Shaanxi Yulin, 53646; Shaanxi Shenmu, 53651; Shaanxi Jiaxian, 53658; Shaanxi Dingbian, 53725; Shaanxi Jingbian, 53735; Shaanxi Wuqi, 53738; Shaanxi Hengshan, 53740; Shaanxi Zichang, 53748; Shaanxi Suide, 53754; Shaanxi Wubao, 53756; Shaanxi Qingjian, 53757; Shaanxi Zhidan, 53832; Shaanxi Ansai, 53841; Shaanxi Ganquan, 53848; Shaanxi Yanchuan, 53850; Shaanxi Yanchang, 53854; Shaanxi Yichuan, 53857; Shaanxi Fuxian, 53931; Shaanxi Xunyi, 53938; Shaanxi Baishui, 53941; Shaanxi Huangling, 53944; Shaanxi Huanglong, 53946; Shaanxi Tongchuan, 53947; Shaanxi Pucheng, 53948; Shaanxi Chengcheng, 53949; Shaanxi Heyang, 53950; Shaanxi Longxian, 57003; Shaanxi Baoji, 57016; Shaanxi Qianyang, 57021; Shaanxi Linyou, 57022; Shaanxi Fufeng, 57026; Shaanxi Meixian, 57027; Shaanxi Liquan, 57029; Shaanxi Yongshou, 57030; Shaanxi Wugong, 57034; Shaanxi Yaoxian, 57037; Shaanxi Xingping, 57038; Shaanxi Sanyuan, 57041; Shaanxi Fuping, 57042; Shaanxi Dali, 57043; Shaanxi Weinan, 57045; Shaanxi Huashan, 57046; Shaanxi Luonan, 57057; Shaanxi Lueyang, 57106; Shaanxi Fengxian, 57113; Shaanxi Mianxian, 57119; Shaanxi Liuba, 57124; Shaanxi Chenggu, 57128; Shaanxi Foping, 57134; Shaanxi Ningshan, 57137; Shaanxi Zhashui, 57140; Shaanxi Shangxian, 57143; Shaanxi Danfeng, 57153; Shaanxi Shangnan, 57154; Shaanxi Shanyang, 57155; Shaanxi Ningqiang, 57211; Shaanxi Nanzheng, 57213; Shaanxi Ziyang, 57231; Shaanxi Hanyin, 57233; Shaanxi Zhenba, 57238; Shaanxi Xunyang, 57242; Shaanxi Ankang, 57245; Shaanxi Pingli, 57248<br>15. Sichuan Shiqu, 56038; Sichuan Ruoergai, 56079; Sichuan Jiuzhaigou, 56097; Sichuan Ganzi, 56146; Sichuan Seda, 56152; Sichuan Luhuo, 56158; Sichuan Rangtang, 56164; Sichuan Daofu, 56167; Sichuan Jinchuan, 56168; Sichuan Aba, 56171; Sichuan Maerkang, 56172; Sichuan Hongyuan, 56173; Sichuan Xiaojin, 56178; Sichuan Maoxian, 56180; Sichuan Chongzhou, 56181; Sichuan Songpan, 56182; Sichuan Wenchuan, 56183; Sichuan Lixian, 56184; Sichuan Heishui, 56185; Sichuan Mianzhu, 56186; Sichuan Wenjiang, 56187; Sichuan Dujiangyan, 56188; Sichuan Pengzhou, 56189; Sichuan Deyang, 56198; Sichuan Batang, 56247; Sichuan Xinlong, 56251; Sichuan Litang, 56257; Sichuan Danba, 56263; Sichuan Yajiang, 56267; Sichuan Baoxing, 56273; Sichuan Lushan, 56279; Sichuan Mingshan, 56280; Sichuan Pujiang, 56281; Sichuan Longquanyi, 56286; Sichuan Pengshan, 56289; Sichuan Jintang, 56296; Sichuan Renshou,

	56297; Sichuan Ziyang, 56298; Sichuan Daocheng, 56357; Sichuan Luding, 56371; Sichuan Yingjing, 56373; Sichuan Kangding, 56374; Sichuan Hanyuan, 56376; Sichuan Shimian, 56378; Sichuan Hongya, 56380; Sichuan Jiajiang, 56382; Sichuan Qingshen, 56383; Sichuan Emeishan, 56385; Sichuan Ebian, 56387; Sichuan Qianwei, 56389; Sichuan Jingyan, 56390; Sichuan Zizhong, 56393; Sichuan Weiyuan, 56395; Sichuan Zigong, 56396; Sichuan Fushun, 56399; Sichuan Xiangcheng, 56443; Sichuan Muli, 56459; Sichuan Jiulong, 56462; Sichuan Ganluo, 56473; Sichuan Mianning, 56474; Sichuan Yuexi, 56475; Sichuan Xide, 56478; Sichuan Chaojue, 56479; Sichuan Mabian, 56480; Sichuan Leibo, 56485; Sichuan Meigu, 56487; Sichuan Muchuan, 56490; Sichuan Yibinxian, 56491; Sichuan Nanxi, 56493; Sichuan Xingwen, 56496; Sichuan Gongxian, 56499; Sichuan Yanyuan, 56565; Sichuan Dechang, 56569; Sichuan Xichang, 56571; Sichuan Puge, 56575; Sichuan Butuo, 56580; Sichuan Changning, 56593; Sichuan Miyi, 56670; Sichuan Huili, 56671; Sichuan Huidong, 56675; Sichuan Qingchuan, 57204; Sichuan Guangyuan, 57206; Sichuan Jiange, 57208; Sichuan Nanjiang, 57216; Sichuan Wangcang, 57217; Sichuan Wanyuan, 57237; Sichuan Langzhong, 57306; Sichuan Xichong, 57309; Sichuan Bazhong, 57313; Sichuan Nanbu, 57314; Sichuan Yilong, 57315; Sichuan Yingshan, 57318; Sichuan Tongjiang, 57320; Sichuan Pingchang, 57324; Sichuan Dachuan, 57328; Sichuan Kaijiang, 57329; Sichuan Shehong, 57401; Sichuan Suining, 57405; Sichuan Gaoping, 57411; Sichuan Quxian, 57413; Sichuan Guangan, 57415; Sichuan Dazhu, 57420; Sichuan Dongxing, 57503; Sichuan Longchang, 57507; Sichuan Jiangan, 57600; Sichuan Hejiang, 57603; Sichuan Xuyong, 57608<br>16. Tianjin Wuqing , 54523; Tianjin Baodi, 54525; Tianjin Jinghai, 54619; Tianjin Jinnan , 54622; Tianjin Tanggu, 54623; Tianjin Dagang, 54645<br>17. Tibet Basu, 56228<br>18. Xinjiang Akedala, 51058; Xinjiang Fuhai, 51068; Xinjiang Aletai, 51076; Xinjiang Emin, 51145; Xinjiang Bole, 51238; Xinjiang Kelamayi, 51243; Xinjiang Jinghe, 51334; Xinjiang Shawan, 51357; Xinjiang Manasi, 51359; Xinjiang Hutubi, 51367; Xinjiang Changji, 51368; Xinjiang Miquan, 51369; Xinjiang Jimusaer, 51378; Xinjiang Urumqi Pastoral Test Station, 51469; Xinjiang Dabancheng, 51477; Xinjiang Kumishi, 51526; Xinjiang Bayinbuluke, 51542; Xinjiang Yanqi, 51567; Xinjiang Tuokexun, 51571; Xinjiang Tulufan, 51573; Xinjiang Wushi, 51627; Xinjiang Akesu, 51628; Xinjiang Xinhe, 51636; Xinjiang Kuerle, 51656; Xinjiang Atushi, 51704; Xinjiang Wuqia, 51705; Xinjiang Jiashi, 51707; Xinjiang Kashi, 51709; Xinjiang Yuepuhu, 51717; Xinjiang Kepin, 51720; Xinjiang Tazhong, 51747; Xinjiang Yengjisha, 51802; Xinjiang Maigaiti, 51810; Xinjiang Shashe, 51811; Xinjiang Yecheng, 51814; Xinjiang Zepu, 51815; Xinjiang Pishan, 51818; Xinjiang Cele, 51826; Xinjiang Moyu, 51827; Xinjiang Hetan, 51828; Xinjiang Luopu, 51829; Xinjiang Qiemuo, 51855; Xinjiang Balikun, 52101; Xinjiang Naomaohu, 52112<br>19. Yunnan Suijiang, 56483; Yunnan Qiaojia, 56673<br>20. Chongqing Chengkou, 57333; Chongqing Tongnan, 57409; Chongqing Dazu, 57502; Chongqing Rongchang, 57505; Chongqing Yongchuan, 57506

Cluster B	1. Anhui Dangshan, 58015; Anhui Xiaoxian, 58016; Anhui Bozhou, 58102; Anhui Jieshou, 58108; Anhui Taihe, 58109; Anhui Tianzhushan, 58112; Anhui Suixi, 58113; Anhui Woyang, 58114; Anhui Leysin, 58117; Anhui Mengcheng, 58118; Anhui Suzhou, 58122; Anhui Lingbi, 58125; Anhui Sixian, 58126; Anhui Huaiyuan, 58127; Anhui Guzhen, 58128; Anhui Wuhe, 58129; Anhui Funan, 58202; Anhui Fuyang, 58203; Anhui Yingshang, 58210; Anhui Fengtai, 58212; Anhui Huoqiu, 58214; Anhui Changfeng, 58220; Anhui Fengyang, 58222; Anhui Mingguang, 58223; Anhui Dingyuan, 58225; Anhui Laian, 58234; Anhui Chuzhou, 58236; Anhui Tianchang, 58240; Anhui Jinzhai, 58306; Anhui Luan, 58311; Anhui Huoshan, 58314; Anhui Shucheng, 58316; Anhui Yuexi, 58317; Anhui Tongcheng, 58319; Anhui Feixi, 58320; Anhui Feidong, 58323; Anhui Chaohu, 58326; Anhui Lujiang, 58327; Anhui Wuwei, 58329; Anhui Hanshan, 58330; Anhui Wuhu, 58334; Anhui Maanshan, 58336; Anhui Wuhuxian, 58338; Anhui Susong, 58417; Anhui Tongling, 58429; Anhui Nanling, 58431; Anhui Jingxian, 58432; Anhui Xuancheng, 58433; Anhui Jingde, 58435; Anhui Ningguo, 58436; Anhui Huangshan, 58437; Anhui Guangde, 58441; Anhui Langxi, 58442; Anhui Yixian, 58523; Anhui Shexian, 58530<br>2. Guangxi Ziyuan, 57859<br>3. Guizhou Hezhang, 56598; Guizhou Weining, 56691; Guizhou Puan, 56792; Guizhou Tongzi, 57606; Guizhou Daozhen, 57623; Guizhou Zhengan, 57625; Guizhou Wuchuan, 57634; Guizhou Yanhe, 57636; Guizhou Dejiang, 57637; Guizhou Songtao, 57647; Guizhou Bijie, 57707; Guizhou Dafang, 57708; Guizhou Jinsha, 57714; Guizhou Zunyi, 57717; Guizhou Xifeng, 57718; Guizhou Kaiyang, 57719; Guizhou Meitan, 57722; Guizhou Fenggang, 57723; Guizhou Wengan, 57728; Guizhou Yuqing, 57729; Guizhou Sinan, 57731; Guizhou Shiqian, 57734; Guizhou Cengong, 57735; Guizhou Jiangkou, 57736; Guizhou Shibing, 57737; Guizhou Yuping, 57739; Guizhou Wanshan, 57742; Guizhou Nayong, 57800; Guizhou Xianxi, 57803; Guizhou Zhijin, 57805; Guizhou Anshun, 57806; Guizhou Liuzhi, 57807; Guizhou Xiuwen, 57811; Guizhou Pingba, 57814; Guizhou Fuquan, 57821; Guizhou Huangping, 57822; Guizhou Guiding, 57824; Guizhou Kaili, 57825; Guizhou Duyun, 57827; Guizhou Sansui, 57832; Guizhou Taijiang, 57834; Guizhou Jianhe, 57835; Guizhou Leishan, 57837; Guizhou Liping, 57839; Guizhou Tianzhu, 57840; Guizhou Jinping, 57844; Guizhou Qinglong, 57900; Guizhou Guanling, 57903; Guizhou Zhenfeng, 57905; Guizhou Wangmo, 57906; Guizhou Ziyun, 57910; Guizhou Huishui, 57912; Guizhou Longli, 57913; Guizhou Luodian, 57916; Guizhou Dushan, 57922; Guizhou Sandu, 57923; Guizhou Rongjiang, 57932<br>4. Hebei Linzhang, 53773; Hebei Fengfeng, 53894; Hebei Weixian, 53896; Hebei Daming, 54804<br>5. Henan Qinyang, 53972; Henan Qixian, 53974; Henan Jiyuan, 53978; Henan Jiaozuo, 53982; Henan Fengqiu, 53983; Henan Xiuwu, 53984; Henan Huixian, 53985; Henan Xinxiang, 53986; Henan Tangyin, 53991; Henan Junxian, 53992; Henan Neihuang, 53993; Henan Changheng, 53998; Henan Qingfeng, 54902; Henan Fanxian, 54903; Henan Sanmenxia, 57051; Henan Lingbao, 57056;

	Henan Mianchi, 57063; Henan Luoning, 57066; Henan Xinan, 57070; Henan Mengjin, 57071; Henan Mengzhou, 57072; Henan Yichuan, 57074; Henan Ruzhou, 57075; Henan Ruyang, 57078; Henan Wenxian, 57079; Henan Gongyi, 57080; Henan Xingyang, 57081; Henan Dengfeng, 57082; Henan Changge, 57087; Henan Xuchang, 57089; Henan Zhongmou, 57090; Henan Kaifeng, 57091; Henan Lankao, 57093; Henan Yanling, 57095; Henan Qixian, 57096; Henan Taikang, 57099; Henan Xixia, 57156; Henan Neixiang, 57169; Henan Lushan, 57173; Henan Zhenping, 57175; Henan Nanzhao, 57176; Henan Wugang, 57177; Henan Nanyang, 57178; Henan Fangcheng, 57179; Henan Jiaxian, 57180; Henan Xiangcheng, 57182; Henan Linying, 57183; Henan Yexian, 57184; Henan Wuyang, 57185; Henan Luohe, 57186; Henan Sheqi, 57187; Henan Xiping, 57188; Henan Suiping, 57189; Henan Huaiyang, 57192; Henan Xihua, 57193; Henan Runan, 57197; Henan Xinye, 57271; Henan Tanghe, 57273; Henan Biyang, 57281; Henan Tongbai, 57285; Henan Zhumadian, 57290; Henan Pingyu, 57292; Henan Xincai, 57293; Henan Zhengyang, 57295; Henan Xixian, 57296; Henan Xinyang, 57297; Henan Guangshan, 57299; Henan Jigongshan, 57390; Henan Xixian, 57396; Henan Minquan, 58004; Henan Shangqiu , 58005; Henan Yucheng, 58006; Henan Echeng, 58007; Henan Xiayi, 58017; Henan Dancheng, 58100; Henan Huangchuan, 58207; Henan Gushi, 58208; Henan Shangcheng, 58301<br>6. Hubei Yunxi, 57251; Hubei Yunxian, 57253; Hubei Shiyan, 57256; Hubei Zhushan, 57257; Hubei Fangxian, 57259; Hubei Danjiangkou, 57260; Hubei Gucheng, 57268; Hubei Xiangyang, 57278; Hubei Zaoyang, 57279; Hubei Padang, 57355; Hubei Xingshan, 57359; Hubei Baokang, 57361; Hubei Shennongjia, 57362; Hubei Nanzhang, 57363; Hubei Yicheng, 57370; Hubei Jingmen, 57377; Hubei Zhongxiang, 57378; Hubei Suizhou, 57381; Hubei Xiaochang, 57386; Hubei Jingshan, 57387; Hubei Anlu, 57388; Hubei Hongan, 57398; Hubei Macheng, 57399; Hubei Lichuan, 57439; Hubei Jianshi, 57445; Hubei Enshi, 57447; Hubei Yiling , 57453; Hubei Wufeng, 57458; Hubei Songzi, 57469; Hubei Qianjiang, 57475; Hubei Gongan, 57477; Hubei Yingcheng, 57481; Hubei Xiaogan, 57482; Hubei Tianmen, 57483; Hubei Shayang, 57484; Hubei Xiantao, 57485; Hubei Hanchuan, 57486; Hubei Caidian, 57489; Hubei Xinzhou, 57492; Hubei Wuhan, 57494; Hubei Tuanfeng, 57495; Hubei Ezhou, 57496; Hubei Xianfeng, 57540; Hubei Xuanen, 57541; Hubei Hefeng, 57543; Hubei Shishou, 57571; Hubei Jianli, 57573; Hubei Honghu, 57581; Hubei Chibi, 57582; Hubei Jiayu, 57583; Hubei Chongyang, 57586; Hubei Tongcheng, 57589; Hubei Xianning, 57590; Hubei Luotian, 58401; Hubei Yingshan, 58402; Hubei Qichun, 58408; Hubei Huangmei, 58409; Hubei Yangxin, 58500<br>7. Hunan Longshan, 57544; Hunan Sangzhi, 57554; Hunan Zhangjiajie, 57558; Hunan Lixian, 57565; Hunan Nanxian, 57574; Hunan Huarong, 57575; Hunan Yueyang, 57584; Hunan Baojing, 57642; Hunan Yongshun, 57643; Hunan Guzhang, 57646; Hunan Jishou, 57649; Hunan Yuanling, 57655; Hunan Luxi, 57657; Hunan Taoyuan, 57661; Hunan Changde, 57662; Hunan Hanshou, 57663; Hunan Taojiang, 57666; Hunan Anhua, 57669; Hunan Yuanjiang, 57671;

Hunan Xiangyin, 57673; Hunan Ningxiang, 57678; Hunan Milo, 57680; Hunan Pingjiang, 57682; Hunan Changsha, 57687; Hunan Liuyang, 57688; Hunan Fenghuang, 57740; Hunan Xinhuang, 57744; Hunan Zhijiang, 57745; Hunan Xupu, 57752; Hunan Hongjiang, 57754; Hunan Lengshuijiang, 57760; Hunan Xinhua, 57761; Hunan Loudi, 57763; Hunan Shaoshan, 57771; Hunan Xiangxiang, 57772; Hunan Shuangfeng, 57774; Hunan Nanyue, 57776; Hunan Hengshan, 57777; Hunan Youxian, 57779; Hunan Zhuzhou, 57780; Hunan Liling, 57781; Hunan Tongtao, 57845; Hunan Lengshuitan, 57865; Hunan Yongzhou, 57866; Hunan Qiyang, 57868; Hunan Qidong, 57870; Hunan Hengyangxian, 57871; Hunan Hengyang, 57872; Hunan Changning, 57874; Hunan Hengnan, 57875; Hunan Anren, 57881; Hunan Chaling, 57882; Hunan Yongxing, 57887; Hunan Guangxi, 57889; Hunan Shuangpai, 57962; Hunan Daoxian, 57965; Hunan Ningyuan, 57966; Hunan Jiangyong, 57969; Hunan Xintian, 57971; Hunan Guiyang, 57973; Hunan Jiahe, 57974; Hunan Zixing, 57981; Hunan Jianghua, 59063

8. Jiangsu Fengxian, 58012; Jiangsu Peixian, 58013; Jiangsu Pizhou, 58026; Jiangsu Xuzhou, 58027; Jiangsu Donghai, 58036; Jiangsu Shuyang, 58038; Jiangsu Ganyu, 58040; Jiangsu Xiliandao, 58041; Jiangsu Lianyungang, 58044; Jiangsu Xiangshui, 58045; Jiangsu Guanyun, 58047; Jiangsu Suining, 58130; Jiangsu Suyu, 58131; Jiangsu Siyang, 58132; Jiangsu Sihong, 58135; Jiangsu Xuyi, 58138; Jiangsu Hongze, 58139; Jiangsu Lianshui, 58140; Jiangsu Huaian, 58141; Jiangsu Funing, 58143; Jiangsu Jianhu, 58146; Jiangsu Jinhu, 58147; Jiangsu Baoying, 58148; Jiangsu Yancheng, 58154; Jiangsu Dafeng, 58158; Jiangsu Liuhe, 58235; Jiangsu Pukou, 58237; Jiangsu Nanjing, 58238; Jiangsu Gaoyou, 58241; Jiangsu Yizheng, 58242; Jiangsu Xinghua, 58243; Jiangsu Taizhou, 58246; Jiangsu Yangzhong, 58247; Jiangsu Taixing, 58249; Jiangsu Jiangyan, 58250; Jiangsu Dongtai, 58251; Jiangsu Haian, 58254; Jiangsu Rugao, 58255; Jiangsu Jingjiang, 58257; Jiangsu Nantong, 58259; Jiangsu Rudong, 58264; Jiangsu Lvsi, 58265; Jiangsu Qidong, 58269; Jiangsu Gaochun, 58339; Jiangsu Lishui, 58340; Jiangsu Danyang, 58341; Jiangsu Jintan, 58342; Jiangsu Jurong, 58344; Jiangsu Yixing, 58346; Jiangsu Suzhou, 58349; Jiangsu Changshu, 58352; Jiangsu Zhangjiagang, 58353; Jiangsu Wuxi, 58354; Jiangsu Kunshan, 58356; Jiangsu Haimen, 58360; Jiangsu Taicang, 58377
9. Jiangxi Tonggu, 57694; Jiangxi Pingxiang, 57786; Jiangxi Lianhua, 57789; Jiangxi Shangli, 57783
10. Shandong Xinxian, 54808; Shandong Yutai, 54907; Shandong Caoxian, 58002; Shandong Chengwu, 58003; Shandong Xuechen, 58021; Shandong Yicheng, 58022
11. Shanxi Lingchuan, 53981
12. Shaanxi Langao, 57247; Shaanxi Baihe, 57254; Shaanxi Zhenping, 57343
13. Shanghai Minhang, 58361; Shanghai Baoshan, 58362; Shanghai Fengxian, 58463
14. Sichuan Linshui, 57416; Sichuan Wusheng, 57417
15. Yunnan Yiliang, 56594; Yunnan Zhenxiong, 56595

	16. Zhejiang Changxing, 58443<br>17. Chongqing Kaixian, 57338; Chongqing Yunyang, 57339; Chongqing Wuxi, 57345; Chongqing Wushan, 57349; Chongqing Dianjiang, 57425; Chongqing Wanzhou, 57432; Chongqing Zhongxian, 57437; Chongqing Shizhu, 57438; Chongqing Wansheng, 57509; Chongqing Tongliang, 57510; Chongqing Beibei, 57511; Chongqing Yubei, 57513; Chongqing Bishan, 57514; Chongqing Jiangjin, 57517; Chongqing Banan, 57518; Chongqing Nanchuan, 57519; Chongqing Changshou, 57520; Chongqing Fengdu, 57523; Chongqing Wulong, 57525; Chongqing Qianjiang, 57536; Chongqing Pengshui, 57537; Chongqing Qijiang, 57612
Cluster C	1. Anhui Qimen, 58520; Anhui Tunxi, 58531<br>2. Fujian Guangze, 58724; Fujian Shaowu, 58725; Fujian Wuyishan, 58730; Fujian Pucheng, 58731; Fujian Jianyang, 58734; Fujian Songxi, 58735; Fujian Zhenghe, 58736; Fujian Shouning, 58744; Fujian Zhouning, 58747; Fujian Fuan, 58748; Fujian Zherong, 58749; Fujian Fuding, 58754; Fujian Ninghuai, 58818; Fujian Taining, 58820; Fujian Jianning, 58822; Fujian Shunchang, 58823; Fujian Mingxi, 58824; Fujian Sanming, 58828; Fujian Gutian, 58836; Fujian Youxi, 58837; Fujian Shuqing, 58839; Fujian Xiapu, 58843; Fujian Minhou, 58844; Fujian Ningde, 58846; Fujian Lianjiang, 58848; Fujian Changting, 58911; Fujian Wuping, 58917; Fujian Datian, 58923; Fujian Zhangping, 58926; Fujian Huaan, 58928; Fujian Anxi, 58929; Fujian Jiuxianshan, 58931; Fujian Yongtai, 58932; Fujian Xiuyu, 58938; Fujian Changle, 58941; Fujian Fuqing, 58942; Fujian Pingtan, 58944; Fujian Putian, 58946; Fujian Changtai, 59122; Fujian Nanjing, 59124; Fujian Pinghe, 59125; Fujian Zhangpu, 59129; Fujian Tongan, 59130; Fujian Nanan, 59131; Fujian Chongwu, 59133<br>3. Hubei Wuxue, 58501<br>4. Hunan Lanshan, 57975<br>5. Jiangxi Xiushui, 57598; Jiangxi Wanzai, 57698; Jiangxi Shanggao, 57699; Jiangxi Fenyi, 57792; Jiangxi Yichun, 57793; Jiangxi Xinyu, 57796; Jiangxi Anfu, 57798; Jiangxi Jianxian, 57799; Jiangxi Xiaping, 57883; Jiangxi Yongxin, 57891; Jiangxi Wanan, 57895; Jiangxi Suichuan, 57896; Jiangxi Taihe, 57899; Jiangxi Chongyi, 57990; Jiangxi Ganxian, 57993; Jiangxi Jiujiang, 58502; Jiangxi Ruichang, 58503; Jiangxi Lushan, 58506; Jiangxi Wuning, 58507; Jiangxi Dean, 58508; Jiangxi Hukou, 58510; Jiangxi Pengze, 58512; Jiangxi Poyang, 58519; Jiangxi Jingdezhen, 58527; Jiangxi Wuyuan, 58529; Jiangxi Jingan, 58600; Jiangxi Fengxin, 58601; Jiangxi Anyi, 58602; Jiangxi Gaoan, 58605; Jiangxi Nanchang, 58606; Jiangxi Yugan, 58612; Jiangxi Jinxian, 58614; Jiangxi Wannian, 58615; Jiangxi Dongxiang, 58618; Jiangxi Linchuan, 58619; Jiangxi Dexing, 58622; Jiangxi Shangraoxian, 58623; Jiangxi Guixi, 58626; Jiangxi Qianshan, 58629; Jiangxi Yushan, 58634; Jiangxi Xinjian, 58693; Jiangxi Xiajiang, 58704; Jiangxi Yongfeng, 58705; Jiangxi Lean, 58706; Jiangxi Chongren, 58710; Jiangxi Jinxi, 58712; Jiangxi Nancheng, 58715; Jiangxi Nanfeng , 58718; Jiangxi Lichuan, 58719; Jiangxi Xingguo, 58804; Jiangxi Ningdu, 58806; Jiangxi Guangchang, 58813; Jiangxi Shicheng, 58814; Jiangxi Yudu, 58905; Jiangxi Huichang, 58906; Jiangxi Anyuan, 58907

	6. Shanghai Jinshan, 58460<br>7. Zhejiang Anji, 58446; Zhejiang Linan, 58448; Zhejiang Fuyang, 58449; Zhejiang Huzhou, 58450; Zhejiang Jiashan, 58451; Zhejiang Jiaxing, 58452; Zhejiang Shaoxing, 58453; Zhejiang Deqing, 58454; Zhejiang Hangzhou, 58457; Zhejiang Cixi, 58467; Zhejiang Daishan, 58484; Zhejiang Kaihua, 58537; Zhejiang Tonglu, 58542; Zhejiang Jiande, 58544; Zhejiang Pujiang, 58546; Zhejiang Longyou, 58547; Zhejiang Jinhua, 58549; Zhejiang Zhuji, 58550; Zhejiang Xinchang, 58555; Zhejiang Shengzhou, 58556; Zhejiang Dongyang, 58558; Zhejiang Tiantai, 58559; Zhejiang Panan, 58560; Zhejiang Zhenhai, 58561; Zhejiang Yinzhou, 58562; Zhejiang Fenghua, 58565; Zhejiang Xiangshan, 58566; Zhejiang Ninghai, 58567; Zhejiang Sanmen, 58568; Zhejiang Shipu, 58569; Zhejiang Putuo, 58570; Zhejiang Changshan, 58631; Zhejiang Jiangshan, 58632; Zhejiang Wuyi, 58642; Zhejiang Yongkang, 58643; Zhejiang Suichang, 58644; Zhejiang Lishui, 58646; Zhejiang Longquan, 58647; Zhejiang Xianju, 58652; Zhejiang Jinyun, 58654; Zhejiang Leqing, 58656; Zhejiang Qingtian, 58657; Zhejiang Yongjia, 58658; Zhejiang Linhai, 58660; Zhejiang Wenling, 58664; Zhejiang Hongjia, 58665; Zhejiang Dachen, 58666; Zhejiang Yunhe, 58742; Zhejiang Taishun, 58746; Zhejiang Wencheng, 58750; Zhejiang Pingyang, 58751; Zhejiang Jingning, 58648
Cluster D	1. Gansu Minqin, 52681<br>2. Hebei Shangyi, 53397<br>3. Heilongjiang Mohe, 50136; Heilongjiang Tahe, 50246; Heilongjiang Huzhong, 50247; Heilongjiang Xinlin, 50349; Heilongjiang Huma, 50353; Heilongjiang Jiagedaqi, 50442; Heilongjiang Nenjiang, 50557; Heilongjiang Sunwu, 50564; Heilongjiang Xunke, 50566; Heilongjiang Nehe, 50646; Heilongjiang Wudalianchi, 50655; Heilongjiang Beian, 50656; Heilongjiang Keshan, 50658; Heilongjiang Kedong, 50659; Heilongjiang Wuyiling, 50674; Heilongjiang Longjiang, 50739; Heilongjiang Gannan, 50741; Heilongjiang Fuyu, 50742; Heilongjiang Qiqihaer, 50745; Heilongjiang Lindian, 50749; Heilongjiang Yian, 50750; Heilongjiang Baiquan, 50755; Heilongjiang Hailun, 50756; Heilongjiang Minshui, 50758; Heilongjiang Suileng, 50767; Heilongjiang Wuying, 50772; Heilongjiang Yichun, 50774; Heilongjiang Hegang, 50775; Heilongjiang Luobei, 50776; Heilongjiang Tongjiang, 50778; Heilongjiang Suibin, 50787; Heilongjiang Fujin, 50788; Heilongjiang Dumeng, 50842; Heilongjiang Daqing, 50850; Heilongjiang Qinggang, 50851; Heilongjiang Wangkui, 50852; Heilongjiang Beilin, 50853; Heilongjiang Anda, 50854; Heilongjiang Zhaodong, 50858; Heilongjiang Lansi, 50859; Heilongjiang Qingan, 50861; Heilongjiang Tieli, 50862; Heilongjiang Bayan, 50867; Heilongjiang Tangyuan, 50871; Heilongjiang Jiamusi, 50873; Heilongjiang Yilang, 50877; Heilongjiang Huachuan, 50878; Heilongjiang Huanan, 50879; Heilongjiang Shuangyashan, 50884; Heilongjiang Baoqing, 50888; Heilongjiang Haerbin, 50953; Heilongjiang Hulan, 50956; Heilongjiang Acheng, 50958; Heilongjiang Binxian, 50960; Heilongjiang Mulan, 50962; Heilongjiang Tonghe, 50963; Heilongjiang Zhengfang, 50964; Heilongjiang Yanshou, 50965; Heilongjiang Shangzhi, 50968; Heilongjiang Boli,

Cluster E	50973; Heilongjiang Jixi, 50978; Heilongjiang Linkou, 50979; Heilongjiang Wuchang, 54080; Heilongjiang Hailin, 54092; Heilongjiang Muling, 54093; Heilongjiang Mudanjiang, 54094; Heilongjiang Ningan, 54098<br>4. Jilin Shuangliao, 54142<br>5. Inner Mongolia Eerguna, 50425; Inner Mongolia Elunchunqi, 50445; Inner Mongolia Manzhouli, 50514; Inner Mongolia Evenkeqi, 50525; Inner Mongolia Yakeshi, 50526; Inner Mongolia Xiaoergou, 50548; Inner Mongolia Xinbaerhuzuoqi, 50618; Inner Mongolia Moulidawawoer, 50645; Inner Mongolia Arunqi, 50647; Inner Mongolia Suolun, 50834; Inner Mongolia Wuzhumuqindong, 50915; Inner Mongolia Bayaertuhushuo, 50928; Inner Mongolia Tuquan, 50934; Inner Mongolia Erlianhaote, 53068; Inner Mongolia Narenbaolige, 53083; Inner Mongolia Mandula, 53149; Inner Mongolia Abagaqi, 53192; Inner Mongolia Xianghuangqi, 53289; Inner Mongolia Wulatezhongqi, 53336; Inner Mongolia Wuyuan, 53337; Inner Mongolia Dashetai, 53348; Inner Mongolia Damaoqi, 53352; Inner Mongolia Guyangxian, 53357; Inner Mongolia Siziwang, 53362; Inner Mongolia Xilamuren, 53367; Inner Mongolia Wuchuanxian, 53368; Inner Mongolia Chayouzhongqi, 53378; Inner Mongolia Chayouhouqi, 53384; Inner Mongolia Shangdu, 53385; Inner Mongolia Huade, 53391; Inner Mongolia Dengkou, 53419; Inner Mongolia Hangjinhouqi , 53420; Inner Mongolia Baotou, 53446; Inner Mongolia Tuyouqi, 53455; Inner Mongolia Dalateqi, 53457; Inner Mongolia Huhehaote, 53463; Inner Mongolia Tumutezuoqi, 53464; Inner Mongolia Huhehaote suburb, 53466; Inner Mongolia Tuoketuoxian, 53467; Inner Mongolia Helingeerxian, 53469; Inner Mongolia Zhuozi, 53472; Inner Mongolia Liangcheng, 53475; Inner Mongolia Jining, 53480; Inner Mongolia Xinghe, 53483; Inner Mongolia Wuhai, 53512; Inner Mongolia Linhe, 53513; Inner Mongolia Yikewusu, 53522; Inner Mongolia Etukeqi, 53529; Inner Mongolia Hangjinqi, 53533; Inner Mongolia Dongsheng, 53543; Inner Mongolia Ejinhuoluoqi, 53545; Inner Mongolia Zhungeerqi, 53553; Inner Mongolia Wushenqi, 53644; Inner Mongolia Eduokeqianqi, 53730; Inner Mongolia Xiwuzhumuqin, 54012; Inner Mongolia Fuhe, 54024; Inner Mongolia Zhalute, 54026; Inner Mongolia Balinzuoqi, 54027; Inner Mongolia Gaoliban, 54031; Inner Mongolia Shebotu, 54039; Inner Mongolia Kezuozhongqi, 54047; Inner Mongolia Xilinhaote, 54102; Inner Mongolia Balinyouqi, 54113; Inner Mongolia Linxixian, 54115; Inner Mongolia Keshiketengqi, 54117; Inner Mongolia Alukeerqinqi, 54122; Inner Mongolia Kailu, 54134; Inner Mongolia Tongliao, 54135; Inner Mongolia Zhenglanqi, 54205; Inner Mongolia Duolunxian, 54208; Inner Mongolia Wengniuteqi, 54213; Inner Mongolia Gangzi, 54214; Inner Mongolia Chifeng, 54218; Inner Mongolia Neiman, 54223; Inner Mongolia Baoguotu, 54226; Inner Mongolia Kalaqinqi, 54313<br>6. Ningxia Shitanjing, 53517<br>7. Sichuan Baiyu, 56147<br>8. Xinjiang Buerjin, 51060; Xinjiang Hami, 52203
Cluster E	1. Fujian Yongding, 59113; Fujian Zhaoan, 59320; Fujian Yunxiao, 59322

	2. Guangdong Lechang, 57988; Guangdong Renhua, 57989; Guangdong Nanxiong, 57996; Guangdong Lianshan, 59074; Guangdong Yangshan, 59075; Guangdong Ruyuan, 59081; Guangdong Shaoguan, 59082; Guangdong Fogang, 59087; Guangdong Yingde, 59088; Guangdong Shixing, 59090; Guangdong Wengyuan, 59094; Guangdong Lianping, 59096; Guangdong Heping, 59099; Guangdong Pingyuan, 59106; Guangdong Longchuan, 59107; Guangdong Xingning, 59109; Guangdong Jiaoling, 59114; Guangdong Daipu, 59116; Guangdong Meixian, 59117; Guangdong Yunan, 59268; Guangdong Huaiji, 59270; Guangdong Guangning, 59271; Guangdong Sihui, 59276; Guangdong Sanshui, 59279; Guangdong Qingyuan, 59280; Guangdong Huadu, 59284; Guangdong Conghua, 59285; Guangdong Guangzhou, 59287; Guangdong Dongguan, 59289; Guangdong Longmen, 59290; Guangdong Heyuan, 59293; Guangdong Boluo, 59297; Guangdong Wuhua, 59303; Guangdong Zijin, 59304; Guangdong Jiexi, 59306; Guangdong Fengshun, 59310; Guangdong Chaozhou, 59312; Guangdong Raoping, 59313; Guangdong Puning, 59314; Guangdong Shantou, 59316; Guangdong Xinyi, 59456; Guangdong Luoding, 59462; Guangdong Yangchun, 59469; Guangdong Xinxing, 59470; Guangdong Yunfu, 59471; Guangdong Heshan, 59473; Guangdong Kaiping, 59475; Guangdong Xinhui, 59476; Guangdong Enping, 59477; Guangdong Panyu, 59481; Guangdong Doumen, 59487; Guangdong Zhuhai, 59488; Guangdong Huidong, 59492; Guangdong Shenzhen, 59493; Guangdong Haifeng, 59500; Guangdong Lufeng, 59502; Guangdong Suixi, 59650; Guangdong Gaozhou, 59653; Guangdong Lianjiang, 59654; Guangdong Huazhou, 59655; Guangdong Wuchuan, 59656; Guangdong Maoming, 59659; Guangdong Leizhou, 59750<br>3. Guangxi Tiane, 57927; Guangxi Sanjiang, 57941; Guangxi Longsheng, 57942; Guangxi Rongshui, 57948; Guangxi Yongfu, 57949; Guangxi Lingui, 57954; Guangxi Xingan, 57955; Guangxi Quanzhou, 57960; Guangxi Guanyang, 57964; Guangxi Xilin, 59004; Guangxi Leye, 59012; Guangxi Lingyun, 59015; Guangxi Tianlin, 59017; Guangxi Fengshan, 59021; Guangxi Hechi, 59023; Guangxi Bama, 59027; Guangxi Yizhou, 59034; Guangxi Duan, 59037; Guangxi Xincheng, 59038; Guangxi Liucheng, 59041; Guangxi Luzhai, 59045; Guangxi Pingle, 59053; Guangxi Lipu, 59055; Guangxi Jinxiu, 59057; Guangxi Mengshan, 59058; Guangxi Hezhou, 59065; Guangxi Baise, 59211; Guangxi Debao, 59215; Guangxi Jingxi, 59218; Guangxi Tiandong, 59224; Guangxi Tiandeng, 59227; Guangxi Longan, 59229; Guangxi Mashan, 59230; Guangxi Shanglin, 59235; Guangxi Wuming , 59237; Guangxi Binyang, 59238; Guangxi Xiangzhou, 59241; Guangxi Laibin, 59242; Guangxi Wuxuan, 59246; Guangxi Pingnan, 59255; Guangxi Tengxian, 59256; Guangxi Wuzhou, 59265; Guangxi Cangwu, 59266; Guangxi Daxin, 59421; Guangxi Shangsi, 59429; Guangxi Nanning, 59431; Guangxi Yongning, 59435; Guangxi Hengxian, 59441; Guangxi Lingshan, 59446; Guangxi Pubei, 59448; Guangxi Bobai, 59449; Guangxi Beiliu, 59451; Guangxi Rongxian, 59452; Guangxi Cenxi, 59454; Guangxi Luchuan, 59457; Guangxi Qinzhou, 59632; Guangxi Hepu, 59640<br>4. Guizhou Ceheng, 57909; Guizhou Pingtang, 57921; Guizhou Libo, 57926;

	Guizhou Congjiang, 57936<br>5. Hunan Dongan, 57867; Hunan Chenzhou, 57972; Hunan Yizhang, 57976; Hunan Linwu, 57978; Hunan Rucheng, 57985<br>6. Jiangxi Nankang, 57992; Jiangxi Xinfeng, 57995; Jiangxi Quannan, 59091; Jiangxi Longnan, 59092; Jiangxi Dingnan, 59093; Jiangxi Xunwu, 59102
Cluster F	1. Guizhou Panxian, 56793; Guizhou Xingyi, 57907<br>2. Sichuan Derong, 56441; Sichuan Pingshan, 56494; Sichuan Jinyang, 56584; Sichuan Yanbian, 56665; Sichuan Panzhihua, 56666<br>3. Yunnan Yanjin, 56497; Yunnan Xianggelila, 56543; Yunnan Weixi, 56548; Yunnan Ninglang, 56567; Yunnan Daguan, 56582; Yunnan Ludian, 56585; Yunnan Weixin, 56596; Yunnan Liuku, 56643; Yunnan Lanping, 56645; Yunnan Jianchuan, 56646; Yunnan Eryuan, 56649; Yunnan Lijiang, 56651; Yunnan Yongsheng, 56652; Yunnan Heqing, 56654; Yunnan Huaping, 56664; Yunnan Yongren, 56669; Yunnan Dongchuan, 56688; Yunnan Xuanwei, 56697; Yunnan Tengchong, 56739; Yunnan Yunlong, 56742; Yunnan Yangbi, 56745; Yunnan Yongping, 56746; Yunnan Baoshan, 56748; Yunnan Dali, 56751; Yunnan Binchuan, 56752; Yunnan Midu, 56755; Yunnan Weishan, 56757; Yunnan Yaoan, 56764; Yunnan Mouding, 56766; Yunnan Nanhua, 56767; Yunnan Fumin, 56772; Yunnan Wuding, 56774; Yunnan Lufeng, 56777; Yunnan Kunming, 56778; Yunnan Malong, 56782; Yunnan Qujing, 56783; Yunnan Songming, 56785; Yunnan Fuyuan, 56790; Yunnan Lianghe, 56840; Yunnan Longling, 56841; Yunnan Shidian, 56842; Yunnan Changning, 56843; Yunnan Fengqing, 56846; Yunnan Yongde, 56849; Yunnan Yunxian, 56854; Yunnan Jingdong, 56856; Yunnan Shuangbai, 56862; Yunnan Anning, 56863; Yunnan Zhenyuan, 56867; Yunnan Xinping, 56869; Yunnan Yimen, 56870; Yunnan Jinning, 56871; Yunnan Chengjiang, 56873; Yunnan Yuxi, 56875; Yunnan Huaning, 56879; Yunnan Yiliang, 56880; Yunnan Shilin, 56881; Yunnan Shizong, 56883; Yunnan Mile, 56885; Yunnan Luxi, 56886; Yunnan Qiubei, 56889; Yunnan Luoping, 56891; Yunnan Eshan, 56898; Yunnan Cangyuan, 56944; Yunnan Gengma, 56946; Yunnan Ximeng, 56948; Yunnan Menglian, 56949; Yunnan Shuangjiang, 56950; Yunnan Lincang, 56951; Yunnan Jinggu, 56952; Yunnan Lancang, 56954; Yunnan Menghai, 56958; Yunnan Mojiang, 56962; Yunnan Simao, 56964; Yunnan Yuanjiang, 56966; Yunnan Mengla, 56969; Yunnan Shiping, 56970; Yunnan Yuanyang, 56976; Yunnan Luchun, 56978; Yunnan Kaiyuan, 56982; Yunnan Gejiu, 56984; Yunnan Yanshan, 56991; Yunnan Xichou, 56992; Yunnan Wenshan, 56994; Yunnan Guangnan, 59007
Cluster G	Guangxi Napo, 59209; Guangxi Pingxiang, 59419<br>Heilongjiang Beijicun, 50137; Heilongjiang Aihui, 50468; Heilongjiang Jiayin, 50673; Heilongjiang Fuyuan, 50779; Heilongjiang Raohe, 50892; Heilongjiang Qitaihe, 50971; Heilongjiang Hulin, 50983; Heilongjiang Mishan, 50985; Heilongjiang Jidong, 50987; Heilongjiang Suifenhe, 54096<br>Jilin Longjing, 54290; Jilin Huichun, 54291; Jilin Linjiang, 54374; Jilin Changbai, 54386<br>Tibet Nielamu, 55655<br>Xinjiang Habahe, 51053; Xinjiang Tacheng, 51133; Xinjiang Yumin, 51137; Xinjiang

	Alashankou, 51232<br>Yunnan Gongshan, 56533; Yunnan Fugong, 56641; Yunnan Longchuan, 56835; Yunnan Yingjiang, 56836; Yunnan Zhenkang, 56839; Yunnan Jiangcheng, 56977; Yunnan Jinping, 56987; Yunnan Funing, 59205
Cluster H	1. Gansu Sunan, 52643<br>2. Qinghai Nuomuhong, 52825; Qinghai Doulan, 52836; Qinghai Qinghaihu 151, 52854; Qinghai Gonghe, 52856; Qinghai Huzhu, 52863; Qinghai Xining, 52866; Qinghai Pingan, 52875; Qinghai Minhe, 52876; Qinghai Hualong, 52877; Qinghai Wudaoliang, 52908; Qinghai Shazhuyu, 52941; Qinghai Guinan, 52955; Qinghai Tongde, 52957; Qinghai Jianzha, 52963; Qinghai Zeku, 52968; Qinghai Xunhua, 52972; Qinghai Tongren, 52974; Qinghai Tuotuohe, 56004; Qinghai Qumacai, 56021; Qinghai Yushu, 56029; Qinghai Maduo, 56033; Qinghai Qingshuihe, 56034; Qinghai Maqin, 56043; Qinghai Gander, 56045; Qinghai Dari, 56046; Qinghai Nangqian, 56125
Cluster I	1. Beijing Shunyi, 54398; Beijing Haidian, 54399; Beijing Miyun, 54416; Beijing Miyunshangdianzi, 54421; Beijing Pinggu, 54424; Beijing Tongzhou, 54431; Beijing Changping, 54499; Beijing Zhaitang, 54501; Beijing, 54511<br>2. Hebei Huailai, 54405; Hebei Luanping, 54420; Hebei Xinglong, 54425; Hebei Dachang, 54510
Cluster J	1. Hainan Haikou, 59758; Hainan Lingao, 59842; Hainan Chengmai, 59843; Hainan Danzhou, 59845; Hainan Changjiang, 59847; Hainan Baisha, 59848; Hainan Dingan, 59851; Hainan Tunchang, 59854; Hainan Qonghai, 59855; Hainan Wenchang, 59856; Hainan Baoting, 59945; Hainan Wanning, 59951
Cluster K	1. Tibet Shiquanhe, 55228; Tibet Naqu, 55299; Tibet Dangxiong, 55493; Tibet Lazi, 55569; Tibet Nanmulin, 55572; Tibet Muozhugongka, 55593; Tibet Jiangzi, 55680; Tibet Leiwuqi, 56128; Tibet Linzhi, 56312; Tibet Miling, 56317

Table A6. SO_2 emissions from transportation sector in 2010

Cluster A	1. Beijing Shunyi, 54398; Beijing Haidian, 54399; Beijing Yanqing, 54406; Beijing Miyun, 54416; Beijing Miyunshangdianzi, 54421; Beijing Pinggu, 54424; Beijing Tongzhou, 54431; Beijing Changping, 54499; Beijing Zhaitang, 54501; Beijing, 54511; Beijing Xiayunling, 54597<br>2. Gansu Gaitai, 52546; Gansu Linze, 52557; Gansu Sunan, 52643; Gansu Minle, 52656; Gansu Yongchang, 52674; Gansu Wuwei, 52679; Gansu Minqin, 52681; Gansu Gulang, 52784; Gansu Wushaoling, 52787; Gansu Jingtai, 52797; Gansu Tianzhu, 52881; Gansu Gaolan, 52884; Gansu Yongdeng, 52885; Gansu Jingyuan, 52895; Gansu Baiyin, 52896; Gansu Xiahe, 52978; Gansu Yongjing, 52980; Gansu Dongxiang, 52981; Gansu Guanghe, 52982; Gansu Yuzhong, 52983; Gansu Hezheng, 52985; Gansu Lintao, 52986; Gansu Kangle, 52988; Gansu Huining, 52993; Gansu Anding, 52995; Gansu Huajialing, 52996; Gansu Weiyuan, 52998; Gansu Huanxian, 53821; Gansu Qingcheng, 53829; Gansu Jingning, 53906; Gansu Kongtong, 53915; Gansu Zhuanglang, 53917; Gansu Xifeng, 53923; Gansu Lingtai, 53924; Gansu Zhenyuan, 53925; Gansu Jingchuan, 53926; Gansu Huating, 53927; Gansu Huachi, 53930; Gansu Huishui, 53934; Gansu Zhengning, 53935; Gansu Luqu, 56071; Gansu Maqu, 56074; Gansu Hezuo, 56080; Gansu Lintan, 56081; Gansu Dibu, 56084; Gansu Zhangxian, 56091; Gansu Longxi, 56092; Gansu Minxian, 56093; Gansu Zhouqu, 56094; Gansu Dangchang, 56095; Gansu Wudu, 56096; Gansu Wenxian, 56192; Gansu Gangu, 57001; Gansu Qinan, 57002; Gansu Wushan, 57004; Gansu Tianshui, 57006; Gansu Lixian, 57007; Gansu Qingshui, 57011; Gansu Zhangjiachuan, 57012; Gansu Maiji, 57014; Gansu Chengxian, 57102; Gansu Kangxian, 57105; Gansu Huixian, 57110; Gansu Liangdang, 57111<br>3. Hebei Kangbao, 53392; Hebei Zhangbei, 53399; Hebei Huaian, 53491; Hebei Yangyuan, 53492; Hebei Xuanhua, 53498; Hebei Wanquan, 53499; Hebei Weixian, 53593; Hebei Shunping, 53596; Hebei Laiyuan, 53599; Hebei Lingshou, 53680; Hebei Quyang, 53682; Hebei Xingtang, 53688; Hebei Jinzhou, 53689; Hebei Fuping, 53690; Hebei Tangxian, 53692; Hebei Dingzhou, 53696; Hebei Wuji, 53699; Hebei Shahe, 53781; Hebei Baixiang, 53785; Hebei Luancheng, 53789; Hebei Longyao, 53794; Hebei Zanhuang, 53795; Hebei Ningjin, 53796; Hebei Julu, 53799; Hebei Shexian, 53886; Hebei Wuan, 53890; Hebei Handan, 53892; Hebei Quzhou, 53893; Hebei Guyuan, 54301; Hebei Chongli, 54304; Hebei Fengning, 54308; Hebei Weichang, 54311; Hebei Longhua, 54318; Hebei Pingquan, 54319; Hebei Zhangjiakou, 54401; Hebei Chicheng, 54404; Hebei Huailai, 54405; Hebei Zhulu, 54408; Hebei Luanping, 54420; Hebei Chengde, 54423; Hebei Xinglong, 54425; Hebei Zunhua, 54429; Hebei Chengdexian, 54430; Hebei Kuancheng, 54432; Hebei Qianxi, 54434; Hebei Qinglong, 54436; Hebei Luannan, 54437; Hebei Lulong, 54438; Hebei Qianan, 54439; Hebei Qinhuangdao, 54449; Hebei Zhuozhou, 54502; Hebei Rongcheng, 54503; Hebei Gaobeidian, 54506; Hebei Dachang, 54510; Hebei Guan, 54512; Hebei Yongqing, 54519; Hebei Sanhe, 54520; Hebei Xianghe,

54521; Hebei Yutian, 54522; Hebei Tangshan, 54534; Hebei Caofeidian, 54535; Hebei Leting, 54539; Hebei Changli, 54540; Hebei Funing, 54541; Hebei Xushui, 54601; Hebei Goyang, 54603; Hebei Anxin, 54605; Hebei Raoyang, 54606; Hebei Shenzhou, 54608; Hebei Renqiu, 54610; Hebei Wenan, 54612; Hebei Dacheng, 54613; Hebei Hejian, 54614; Hebei Qingxian, 54615; Hebei Cangzhou, 54616; Hebei Botou, 54618; Hebei Huanghua, 54624; Hebei Haixing, 54628; Hebei Guangzong, 54631; Hebei Xinhe, 54633; Hebei Jize, 54640; Hebei Wuqiang, 54700; Hebei Xinji, 54701; Hebei Hengshui, 54702; Hebei Wuyi, 54703; Hebei Qinghe, 54706; Hebei Gucheng, 54707; Hebei Fucheng, 54710; Hebei Jingxian, 54711; Hebei Dongguang, 54713; Hebei-Weixian, 54800; Hebei Linxi, 54801; Hebei Guantao, 54809

4. Henan Linzhou, 53889; Henan Taiqian, 54817; Henan Puyang, 54900
5. Heilongjiang Lindian, 50749; Heilongjiang Tailai, 50844; Heilongjiang Wuchang, 54080
6. Hubei Zhuxi, 57249
7. Jilin Baicheng, 50936; Jilin Taonan, 50939; Jilin Zhenlai, 50940; Jilin Daan, 50945; Jilin Songyuan, 50946; Jilin Qianan, 50948; Jilin Qianguo, 50949; Jilin Tongyu, 54041; Jilin Changling, 54049; Jilin Fuyu, 54063; Jilin Nongan, 54064; Jilin Dehui, 54065; Jilin Jiutai, 54069; Jilin Yushu, 54072; Jilin Shulan, 54076; Jilin Lishu, 54154; Jilin Gujiazi, 54155; Jilin Changchun, 54161; Jilin Yitong, 54164; Jilin Shuangyang, 54165; Jilin Yantongshan, 54169; Jilin Yongji, 54171; Jilin Jilin Suburb, 54172; Jilin Jiaohe, 54181; Jilin Dunhua, 54186; Jilin Antu, 54187; Jilin Luozigou, 54192; Jilin Wangqing, 54195; Jilin Liaoyuan, 54260; Jilin Tongfeng, 54261; Jilin Panshi, 54263; Jilin Liuhe, 54267; Jilin Huadian, 54273; Jilin Huinan, 54274; Jilin Jiangyuan, 54279; Jilin Donggang, 54284; Jilin Erdao, 54285; Jilin Helong, 54286; Jilin Yanji, 54292; Jilin Tonghuaxian, 54362; Jilin Tonghua, 54363; Jilin Baishan, 54371; Jilin Jian, 54377
8. Liaoning Zhangwu, 54236; Liaoning Changtu, 54243; Liaoning Kangping, 54244; Liaoning Shenbei, 54248; Liaoning Tieling, 54249; Liaoning Xifeng, 54252; Liaoning Qingyuan, 54259; Liaoning Jianpingzhen, 54321; Liaoning Chaoyang, 54324; Liaoning Yangshan, 54325; Liaoning Jianpingxian, 54326; Liaoning Lingyuan, 54327; Liaoning Liaozhong, 54332; Liaoning Xinmin, 54333; Liaoning Taian, 54336; Liaoning Panshan, 54338; Liaoning Anshan, 54339; Liaoning Sujiatun, 54340; Liaoning Shenyang, 54342; Liaoning Liaoyangxian, 54345; Liaoning Benxi, 54346; Liaoning Fushun, 54351; Liaoning Xinbin, 54353; Liaoning Jianchang, 54452; Liaoning Lianshan, 54453; Liaoning Suizhong, 54454; Liaoning Xingcheng, 54455; Liaoning Yingkou, 54471; Liaoning Gaizhou, 54474; Liaoning Dashiqiao, 54475; Liaoning Caohekou, 54483; Liaoning Xiuyan, 54486; Liaoning Kuandian, 54493; Liaoning Fengcheng, 54494; Liaoning Dandong, 54497; Liaoning Wafangdian, 54563; Liaoning Jinzhou, 54568; Liaoning Pulandian, 54569; Liaoning Pikou, 54575; Liaoning Changhai, 54579; Liaoning Zhuanghe, 54584; Liaoning Changxingdao, 54565
9. Inner Mongolia Evenkeqi, 50525; Inner Mongolia Narenbaolige, 53083; Inner Mongolia Chayouqianqi, 53481; Inner Mongolia Henan, 53732; Inner Mongolia

	Qinglongshan, 54132; Inner Mongolia Zhengxiangbaiqi, 54204; Inner Mongolia Aohanqi, 54225; Inner Mongolia Kezuohouqi, 54231; Inner Mongolia Kulun, 54234; Inner Mongolia Taibushiqi, 54305; Inner Mongolia Balihan, 54316; Inner Mongolia Ningchengxian, 54320<br>10. Ningxia Huinong, 53519; Ningxia Helan, 53610; Ningxia Pingluo, 53611; Ningxia Wuzhong, 53612; Ningxia Taole, 53615; Ningxia Yongning, 53618; Ningxia Zhongwei, 53704; Ningxia Zhongning, 53705; Ningxia Xingren, 53707; Ningxia Yanchi, 53723; Ningxia Maihuangshan, 53727; Ningxia Haiyuan, 53806; Ningxia Tongxin, 53810; Ningxia Guyuan, 53817; Ningxia Weizhou, 53881; Ningxia Xiji, 53903; Ningxia Liupanshan, 53910<br>11. Qinghai Nuomuhong, 52825; Qinghai Doulan, 52836; Qinghai Qinghaihu 151, 52854; Qinghai Gonghe, 52856; Qinghai Huzhu, 52863; Qinghai Xining, 52866; Qinghai Pingan, 52875; Qinghai Minhe, 52876; Qinghai Hualong, 52877; Qinghai Wudaoliang, 52908; Qinghai Shazhuyu, 52941; Qinghai Guinan, 52955; Qinghai Tongde, 52957; Qinghai Jianzha, 52963; Qinghai Zeku, 52968; Qinghai Xunhua, 52972; Qinghai Tongren, 52974; Qinghai Tuotuohe, 56004; Qinghai Yushu, 56029; Qinghai Maduo, 56033; Qinghai Qingshuihe, 56034; Qinghai Maqin, 56043; Qinghai Gander, 56045; Qinghai Dari, 56046; Qinghai Nangqian, 56125; Qinghai Banma, 56151<br>12. Shandong Wucheng, 54709; Shandong Linyi, 54712; Shandong Ningjin, 54716; Shandong Yangxin, 54723; Shandong Shanghe, 54724; Shandong Leling, 54726; Shandong Zhangqiu, 54727; Shandong Gaoqing, 54729; Shandong Binzhou, 54734; Shandong Kenli, 54744; Shandong Laizhou, 54749; Shandong Longdao, 54751; Shandong Penglai, 54752; Shandong Longkou, 54753; Shandong Zhaoyuan, 54755; Shandong Qixia, 54759; Shandong Fushan, 54764; Shandong Yantai, 54765; Shandong Chengshantou, 54776; Shandong Wendeng, 54777; Shandong Linqing, 54802; Shandong Liaocheng, 54806; Shandong Qihe, 54812; Shandong Chiping, 54814; Shandong Donge, 54815; Shandong Feicheng, 54819; Shandong Jiyang, 54821; Shandong Zouping, 54822; Shandong Jinan, 54823; Shandong Taian, 54827; Shandong Laiwu, 54828; Shandong Zibo, 54830; Shandong Qingzhou, 54831; Shandong Shouguang, 54832; Shandong Huantai, 54833; Shandong Yiyuan, 54836; Shandong Changyi, 54841; Shandong Pingdu, 54842; Shandong Weifang, 54843; Shandong Anqiu, 54844; Shandong Gaomi, 54846; Shandong Zhucheng, 54848; Shandong Jiaozhou, 54849; Shandong Laiyang, 54852; Shandong Jimo, 54855; Shandong Rushan, 54861; Shandong Juancheng, 54904; Shandong Yuncheng, 54905; Shandong Heze, 54906; Shandong Dingtao, 54909; Shandong Liangshan, 54910; Shandong Dongping, 54911; Shandong Wenshang, 54912; Shandong Juye, 54914; Shandong Yanzhou, 54916; Shandong Zoucheng, 54919; Shandong Sishui, 54920; Shandong Xintai, 54922; Shandong Mengyin, 54923; Shandong Pingyi, 54925; Shandong Tengzhou, 54927; Shandong Feixian, 54929; Shandong Yishui, 54932; Shandong Linyi, 54938; Shandong Junan, 54939; Shandong Wulian, 54940; Shandong Huangdao, 54943; Shandong Rizhao, 54945; Shandong Zaozhuang, 58024; Shandong Cangshan, 58030; Shandong Linshu, 58032

	13. Shanxi Youyu, 53478; Shanxi Yanggao, 53486; Shanxi Datong, 53487; Shanxi Hequ, 53564; Shanxi Pianguan, 53565; Shanxi Pinglu, 53574; Shanxi Shenchi, 53575; Shanxi Shanyin, 53576; Shanxi Ningwu, 53577; Shanxi Shuozhou, 53578; Shanxi Daixian, 53579; Shanxi Hunyuan, 53582; Shanxi Yingxian, 53584; Shanxi Fanshi, 53585; Shanxi Wutaishan, 53588; Shanxi Guangling, 53590; Shanxi Linxian, 53659; Shanxi Kelan, 53662; Shanxi Wuzhai, 53663; Shanxi Xingxian, 53664; Shanxi Lanxian, 53665; Shanxi Jingle, 53666; Shanxi Yuanping, 53673; Shanxi Xinfu, 53674; Shanxi Dingxiang, 53676; Shanxi Jiancaoping, 53677; Shanxi Xiaodian, 53679; Shanxi Wutaixian, 53681; Shanxi Yuxian, 53685; Shanxi Pingding, 53687; Shanxi Liulin, 53753; Shanxi Shilou, 53759; Shanxi Fangshan, 53760; Shanxi Gujiao, 53763; Shanxi Lishi, 53764; Shanxi Zhongyang, 53767; Shanxi Xiaoyi, 53768; Shanxi Qingxu, 53774; Shanxi Taigu, 53775; Shanxi Pingyao, 53778; Shanxi Shouyang, 53780; Shanxi Yangquan, 53782; Shanxi Zuoquan, 53786; Shanxi Yushe, 53787; Shanxi Heshun, 53788; Shanxi Yonghe, 53852; Shanxi Xixian, 53853; Shanxi Jixian, 53859; Shanxi Jiaokou, 53860; Shanxi Xiangfen, 53861; Shanxi Lingshi, 53862; Shanxi Jiexiu, 53863; Shanxi Puxian, 53864; Shanxi Fenxi, 53865; Shanxi Hongtong, 53866; Shanxi Wuxiang, 53871; Shanxi Qinxian, 53872; Shanxi Changzi, 53873; Shanxi Guxian, 53874; Shanxi Qinyuan, 53875; Shanxi Anze, 53877; Shanxi Licheng, 53878; Shanxi Lucheng, 53880; Shanxi Xiangning, 53953; Shanxi Jishan, 53954; Shanxi Wanrong, 53956; Shanxi Hejin, 53957; Shanxi Yanhu, 53959; Shanxi Xinjiang, 53964; Shanxi Jiangxian, 53965; Shanxi Fushan, 53966; Shanxi Yuanqu, 53968; Shanxi Qingshui, 53970; Shanxi Gaoping, 53973; Shanxi Yongji, 57052; Shanxi Ruicheng, 57053<br>14. Shaanxi Fugu, 53567; Shaanxi Yulin, 53646; Shaanxi Shenmu, 53651; Shaanxi Jiaxian, 53658; Shaanxi Dingbian, 53725; Shaanxi Jingbian, 53735; Shaanxi Wuqi, 53738; Shaanxi Hengshan, 53740; Shaanxi Zichang, 53748; Shaanxi Suide, 53754; Shaanxi Wubao, 53756; Shaanxi Qingjian, 53757; Shaanxi Zhidan, 53832; Shaanxi Ansai, 53841; Shaanxi Ganquan, 53848; Shaanxi Yanchuan, 53850; Shaanxi Yanchang, 53854; Shaanxi Yichuan, 53857; Shaanxi Fuxian, 53931; Shaanxi Xunyi, 53938; Shaanxi Baishui, 53941; Shaanxi Huangling, 53944; Shaanxi Huanglong, 53946; Shaanxi Tongchuan, 53947; Shaanxi Pucheng, 53948; Shaanxi Chengcheng, 53949; Shaanxi Heyang, 53950; Shaanxi Longxian, 57003; Shaanxi Baoji, 57016; Shaanxi Qianyang, 57021; Shaanxi Linyou, 57022; Shaanxi Fufeng, 57026; Shaanxi Meixian, 57027; Shaanxi Liquan, 57029; Shaanxi Yongshou, 57030; Shaanxi Wugong, 57034; Shaanxi Yaoxian, 57037; Shaanxi Xingping, 57038; Shaanxi Sanyuan, 57041; Shaanxi Fuping, 57042; Shaanxi Dali, 57043; Shaanxi Weinan, 57045; Shaanxi Huashan, 57046; Shaanxi Luonan, 57057; Shaanxi Lueyang, 57106; Shaanxi Fengxian, 57113; Shaanxi Mianxian, 57119; Shaanxi Liuba, 57124; Shaanxi Chenggu, 57128; Shaanxi Foping, 57134; Shaanxi Ningshan, 57137; Shaanxi Zhashui, 57140; Shaanxi Shangxian, 57143; Shaanxi Danfeng, 57153; Shaanxi Shangnan, 57154; Shaanxi Shanyang, 57155; Shaanxi Ningqiang, 57211; Shaanxi Nanzheng, 57213; Shaanxi Ziyang, 57231; Shaanxi Hanyin, 57233; Shaanxi Zhenba, 57238;

	Shaanxi Xunyang, 57242; Shaanxi Ankang, 57245; Shaanxi Pingli, 57248<br>15. Sichuan Shiqu, 56038; Sichuan Ruoergai, 56079; Sichuan Jiuzhaigou, 56097; Sichuan Ganzi, 56146; Sichuan Baiyu, 56147; Sichuan Seda, 56152; Sichuan Luhuo, 56158; Sichuan Rangtang, 56164; Sichuan Daofu, 56167; Sichuan Jinchuan, 56168; Sichuan Aba, 56171; Sichuan Maerkang, 56172; Sichuan Hongyuan, 56173; Sichuan Xiaojin, 56178; Sichuan Maoxian, 56180; Sichuan Chongzhou, 56181; Sichuan Songpan, 56182; Sichuan Wenchuan, 56183; Sichuan Lixian, 56184; Sichuan Heishui, 56185; Sichuan Mianzhu, 56186; Sichuan Wenjiang, 56187; Sichuan Dujiangyan, 56188; Sichuan Pengzhou, 56189; Sichuan Deyang, 56198; Sichuan Batang, 56247; Sichuan Xinlong, 56251; Sichuan Litang, 56257; Sichuan Danba, 56263; Sichuan Yajiang, 56267; Sichuan Baoxing, 56273; Sichuan Lushan, 56279; Sichuan Mingshan, 56280; Sichuan Pujiang, 56281; Sichuan Longquanyi, 56286; Sichuan Pengshan, 56289; Sichuan Jintang, 56296; Sichuan Renshou, 56297; Sichuan Ziyang, 56298; Sichuan Daocheng, 56357; Sichuan Luding, 56371; Sichuan Yingjing, 56373; Sichuan Kangding, 56374; Sichuan Hanyuan, 56376; Sichuan Shimian, 56378; Sichuan Hongya, 56380; Sichuan Jiajiang, 56382; Sichuan Qingshen, 56383; Sichuan Emeishan, 56385; Sichuan Ebian, 56387; Sichuan Qianwei, 56389; Sichuan Jingyan, 56390; Sichuan Zizhong, 56393; Sichuan Weiyuan, 56395; Sichuan Zigong, 56396; Sichuan Fushun, 56399; Sichuan Xiangcheng, 56443; Sichuan Muli, 56459; Sichuan Jiulong, 56462; Sichuan Ganluo, 56473; Sichuan Mianning, 56474; Sichuan Yuexi, 56475; Sichuan Xide, 56478; Sichuan Chaojue, 56479; Sichuan Mabian, 56480; Sichuan Leibo, 56485; Sichuan Meigu, 56487; Sichuan Muchuan, 56490; Sichuan Yibinxian, 56491; Sichuan Nanxi, 56493; Sichuan Xingwen, 56496; Sichuan Gongxian, 56499; Sichuan Yanyuan, 56565; Sichuan Dechang, 56569; Sichuan Xichang, 56571; Sichuan Puge, 56575; Sichuan Butuo, 56580; Sichuan Changning, 56593; Sichuan Yanbian, 56665; Sichuan Miyi, 56670; Sichuan Huili, 56671; Sichuan Huidong, 56675; Sichuan Qingchuan, 57204; Sichuan Guangyuan, 57206; Sichuan Jiange, 57208; Sichuan Nanjiang, 57216; Sichuan Wangcang, 57217; Sichuan Wanyuan, 57237; Sichuan Langzhong, 57306; Sichuan Xichong, 57309; Sichuan Bazhong, 57313; Sichuan Nanbu, 57314; Sichuan Yilong, 57315; Sichuan Yingshan, 57318; Sichuan Tongjiang, 57320; Sichuan Pingchang, 57324; Sichuan Dachuan, 57328; Sichuan Kaijiang, 57329; Sichuan Shehong, 57401; Sichuan Suining, 57405; Sichuan Gaoping, 57411; Sichuan Quxian, 57413; Sichuan Guangan, 57415; Sichuan Dazhu, 57420; Sichuan Dongxing, 57503; Sichuan Longchang, 57507; Sichuan Jiangan, 57600; Sichuan Hejiang, 57603; Sichuan Xuyong, 57608<br>16. Tianjin Wuqing , 54523; Tianjin Baodi, 54525; Tianjin Jinghai, 54619; Tianjin Jinnan , 54622; Tianjin Tanggu, 54623; Tianjin Dagang, 54645<br>17. Tibet Shiquanhe, 55228; Tibet Basu, 56228; Tibet Linzhi, 56312<br>18. Xinjiang Akedala, 51058; Xinjiang Buerjin, 51060; Xinjiang Fuhai, 51068; Xinjiang Emin, 51145; Xinjiang Bole, 51238; Xinjiang Kelamayi, 51243; Xinjiang Jinghe, 51334; Xinjiang Shawan, 51357; Xinjiang Manasi, 51359; Xinjiang Hutubi, 51367; Xinjiang Changji, 51368; Xinjiang Miquan, 51369; Xinjiang

	Fukang, 51377; Xinjiang Jimusaer, 51378; Xinjiang Urumqi Pastoral Test Station, 51469; Xinjiang Dabancheng, 51477; Xinjiang Kumishi, 51526; Xinjiang Bayinbuluke, 51542; Xinjiang Yanqi, 51567; Xinjiang Tuokexun, 51571; Xinjiang Tulufan, 51573; Xinjiang Wushi, 51627; Xinjiang Akesu, 51628; Xinjiang Xinhe, 51636; Xinjiang Kuerle, 51656; Xinjiang Atushi, 51704; Xinjiang Wuqia, 51705; Xinjiang Jiashi, 51707; Xinjiang Kashi, 51709; Xinjiang Yuepuhu, 51717; Xinjiang Kepin, 51720; Xinjiang Tazhong, 51747; Xinjiang Yengjisha, 51802; Xinjiang Maigaiti, 51810; Xinjiang Shashe, 51811; Xinjiang Yecheng, 51814; Xinjiang Zepu, 51815; Xinjiang Pishan, 51818; Xinjiang Cele, 51826; Xinjiang Moyu, 51827; Xinjiang Hetan, 51828; Xinjiang Luopu, 51829; Xinjiang Qiemuo, 51855; Xinjiang Balikun, 52101; Xinjiang Naomaohu, 52112; Xinjiang Hami, 52203<br>19. Yunnan Suijiang, 56483; Yunnan Qiaojia, 56673<br>20. Chongqing Chengkou, 57333; Chongqing Tongnan, 57409; Chongqing Dazu, 57502; Chongqing Rongchang, 57505; Chongqing Yongchuan, 57506
Cluster B	1. Anhui Dangshan, 58015; Anhui Xiaoxian, 58016; Anhui Bozhou, 58102; Anhui Jieshou, 58108; Anhui Taihe, 58109; Anhui Tianzhushan, 58112; Anhui Suixi, 58113; Anhui Woyang, 58114; Anhui Leysin, 58117; Anhui Mengcheng, 58118; Anhui Suzhou, 58122; Anhui Lingbi, 58125; Anhui Sixian, 58126; Anhui Huaiyuan, 58127; Anhui Guzhen, 58128; Anhui Wuhe, 58129; Anhui Funan, 58202; Anhui Fuyang, 58203; Anhui Yingshang, 58210; Anhui Fengtai, 58212; Anhui Huoqiu, 58214; Anhui Changfeng, 58220; Anhui Fengyang, 58222; Anhui Mingguang, 58223; Anhui Dingyuan, 58225; Anhui Laian, 58234; Anhui Chuzhou, 58236; Anhui Tianchang, 58240; Anhui Jinzhai, 58306; Anhui Luan, 58311; Anhui Huoshan, 58314; Anhui Shucheng, 58316; Anhui Yuexi, 58317; Anhui Tongcheng, 58319; Anhui Feixi, 58320; Anhui Feidong, 58323; Anhui Chaohu, 58326; Anhui Lujiang, 58327; Anhui Wuwei, 58329; Anhui Hanshan, 58330; Anhui Wuhu, 58334; Anhui Maanshan, 58336; Anhui Wuhuxian, 58338; Anhui Susong, 58417; Anhui Tongling, 58429; Anhui Nanling, 58431; Anhui Jingxian, 58432; Anhui Xuancheng, 58433; Anhui Jingde, 58435; Anhui Ningguo, 58436; Anhui Huangshan, 58437; Anhui Guangde, 58441; Anhui Langxi, 58442; Anhui Yixian, 58523; Anhui Shexian, 58530<br>2. Guangxi Ziyuan, 57859<br>3. Guizhou Hezhang, 56598; Guizhou Weining, 56691; Guizhou Puan, 56792; Guizhou Tongzi, 57606; Guizhou Daozhen, 57623; Guizhou Zhengan, 57625; Guizhou Wuchuan, 57634; Guizhou Yanhe, 57636; Guizhou Dejiang, 57637; Guizhou Songtao, 57647; Guizhou Bijie, 57707; Guizhou Dafang, 57708; Guizhou Jinsha, 57714; Guizhou Zunyi, 57717; Guizhou Xifeng, 57718; Guizhou Kaiyang, 57719; Guizhou Meitan, 57722; Guizhou Fenggang, 57723; Guizhou Wengan, 57728; Guizhou Yuqing, 57729; Guizhou Sinan, 57731; Guizhou Shiqian, 57734; Guizhou Cengong, 57735; Guizhou Jiangkou, 57736; Guizhou Shibing, 57737; Guizhou Yuping, 57739; Guizhou Wanshan, 57742; Guizhou Nayong, 57800; Guizhou Xianxi, 57803; Guizhou Zhijin, 57805; Guizhou Anshun, 57806; Guizhou Liuzhi, 57807; Guizhou Xiuwen, 57811; Guizhou Pingba, 57814; Guizhou Fuquan, 57821; Guizhou Huangping, 57822; Guizhou Guiding, 57824;

Guizhou Kaili, 57825; Guizhou Duyun, 57827; Guizhou Sansui, 57832; Guizhou Taijiang, 57834; Guizhou Jianhe, 57835; Guizhou Leishan, 57837; Guizhou Liping, 57839; Guizhou Tianzhu, 57840; Guizhou Jinping, 57844; Guizhou Qinglong, 57900; Guizhou Guanling, 57903; Guizhou Zhenfeng, 57905; Guizhou Wangmo, 57906; Guizhou Ziyun, 57910; Guizhou Huishui, 57912; Guizhou Longli, 57913; Guizhou Luodian, 57916; Guizhou Dushan, 57922; Guizhou Sandu, 57923; Guizhou Rongjiang, 57932

4. Hebei Linzhang, 53773; Hebei Fengfeng, 53894; Hebei Weixian, 53896; Hebei Daming, 54804
5. Henan Qinyang, 53972; Henan Qixian, 53974; Henan Jiyuan, 53978; Henan Jiaozuo, 53982; Henan Fengqiu, 53983; Henan Xiuwu, 53984; Henan Huixian, 53985; Henan Xinxiang, 53986; Henan Tangyin, 53991; Henan Junxian, 53992; Henan Neihuang, 53993; Henan Changheng, 53998; Henan Qingfeng, 54902; Henan Fanxian, 54903; Henan Sanmenxia, 57051; Henan Lingbao, 57056; Henan Mianchi, 57063; Henan Luoning, 57066; Henan Xinan, 57070; Henan Mengjin, 57071; Henan Mengzhou, 57072; Henan Yichuan, 57074; Henan Ruzhou, 57075; Henan Ruyang, 57078; Henan Wenxian, 57079; Henan Gongyi, 57080; Henan Xingyang, 57081; Henan Dengfeng, 57082; Henan Changge, 57087; Henan Xuchang, 57089; Henan Zhongmou, 57090; Henan Kaifeng, 57091; Henan Lankao, 57093; Henan Yanling, 57095; Henan Qixian, 57096; Henan Taikang, 57099; Henan Xixia, 57156; Henan Neixiang, 57169; Henan Lushan, 57173; Henan Zhenping, 57175; Henan Nanzhao, 57176; Henan Wugang, 57177; Henan Nanyang, 57178; Henan Fangcheng, 57179; Henan Jiaxian, 57180; Henan Xiangcheng, 57182; Henan Linying, 57183; Henan Yexian, 57184; Henan Wuyang, 57185; Henan Luohe, 57186; Henan Sheqi, 57187; Henan Xiping, 57188; Henan Suiping, 57189; Henan Huaiyang, 57192; Henan Xihua, 57193; Henan Runan, 57197; Henan Xinye, 57271; Henan Tanghe, 57273; Henan Biyang, 57281; Henan Tongbai, 57285; Henan Zhumadian, 57290; Henan Pingyu, 57292; Henan Xincai, 57293; Henan Zhengyang, 57295; Henan Xixian, 57296; Henan Xinyang, 57297; Henan Guangshan, 57299; Henan Jigongshan, 57390; Henan Xixian, 57396; Henan Minquan, 58004; Henan Shangqiu , 58005; Henan Yucheng, 58006; Henan Echeng, 58007; Henan Xiayi, 58017; Henan Dancheng, 58100; Henan Huangchuan, 58207; Henan Gushi, 58208; Henan Shangcheng, 58301
6. Hubei Yunxi, 57251; Hubei Yunxian, 57253; Hubei Shiyan, 57256; Hubei Zhushan, 57257; Hubei Fangxian, 57259; Hubei Danjiangkou, 57260; Hubei Gucheng, 57268; Hubei Xiangyang, 57278; Hubei Zaoyang, 57279; Hubei Padang, 57355; Hubei Xingshan, 57359; Hubei Baokang, 57361; Hubei Shennongjia, 57362; Hubei Nanzhang, 57363; Hubei Yicheng, 57370; Hubei Jingmen, 57377; Hubei Zhongxiang, 57378; Hubei Suizhou, 57381; Hubei Xiaochang, 57386; Hubei Jingshan, 57387; Hubei Anlu, 57388; Hubei Hongan, 57398; Hubei Macheng, 57399; Hubei Lichuan, 57439; Hubei Jianshi, 57445; Hubei Enshi, 57447; Hubei Yiling , 57453; Hubei Wufeng, 57458; Hubei Songzi, 57469; Hubei Qianjiang, 57475; Hubei Gongan, 57477; Hubei Yingcheng,

	57481; Hubei Xiaogan, 57482; Hubei Tianmen, 57483; Hubei Shayang, 57484; Hubei Xiantao, 57485; Hubei Hanchuan, 57486; Hubei Caidian, 57489; Hubei Xinzhou, 57492; Hubei Wuhan, 57494; Hubei Tuanfeng, 57495; Hubei Ezhou, 57496; Hubei Xianfeng, 57540; Hubei Xuanen, 57541; Hubei Hefeng, 57543; Hubei Shishou, 57571; Hubei Jianli, 57573; Hubei Honghu, 57581; Hubei Chibi, 57582; Hubei Jiayu, 57583; Hubei Chongyang, 57586; Hubei Tongcheng, 57589; Hubei Xianning, 57590; Hubei Luotian, 58401; Hubei Yingshan, 58402; Hubei Qichun, 58408; Hubei Huangmei, 58409; Hubei Yangxin, 58500<br>7. Hunan Longshan, 57544; Hunan Sangzhi, 57554; Hunan Zhangjiajie, 57558; Hunan Lixian, 57565; Hunan Nanxian, 57574; Hunan Huarong, 57575; Hunan Yueyang, 57584; Hunan Baojing, 57642; Hunan Yongshun, 57643; Hunan Guzhang, 57646; Hunan Jishou, 57649; Hunan Yuanling, 57655; Hunan Luxi, 57657; Hunan Taoyuan, 57661; Hunan Changde, 57662; Hunan Hanshou, 57663; Hunan Taojiang, 57666; Hunan Anhua, 57669; Hunan Yuanjiang, 57671; Hunan Xiangyin, 57673; Hunan Ningxiang, 57678; Hunan Milo, 57680; Hunan Pingjiang, 57682; Hunan Changsha, 57687; Hunan Liuyang, 57688; Hunan Fenghuang, 57740; Hunan Xinhuang, 57744; Hunan Zhijiang, 57745; Hunan Xupu, 57752; Hunan Hongjiang, 57754; Hunan Lengshuijiang, 57760; Hunan Xinhua, 57761; Hunan Loudi, 57763; Hunan Shaoshan, 57771; Hunan Xiangxiang, 57772; Hunan Shuangfeng, 57774; Hunan Nanyue, 57776; Hunan Hengshan, 57777; Hunan Youxian, 57779; Hunan Zhuzhou, 57780; Hunan Liling, 57781; Hunan Tongtao, 57845; Hunan Lengshuitan, 57865; Hunan Yongzhou, 57866; Hunan Qiyang, 57868; Hunan Qidong, 57870; Hunan Hengyangxian, 57871; Hunan Hengyang, 57872; Hunan Changning, 57874; Hunan Hengnan, 57875; Hunan Anren, 57881; Hunan Chaling, 57882; Hunan Yongxing, 57887; Hunan Guangxi, 57889; Hunan Shuangpai, 57962; Hunan Daoxian, 57965; Hunan Ningyuan, 57966; Hunan Jiangyong, 57969; Hunan Xintian, 57971; Hunan Guiyang, 57973; Hunan Jiahe, 57974; Hunan Zixing, 57981; Hunan Jianghua, 59063<br>8. Jiangsu Fengxian, 58012; Jiangsu Peixian, 58013; Jiangsu Pizhou, 58026; Jiangsu Xuzhou, 58027; Jiangsu Donghai, 58036; Jiangsu Shuyang, 58038; Jiangsu Ganyu, 58040; Jiangsu Xiliandao, 58041; Jiangsu Lianyungang, 58044; Jiangsu Xiangshui, 58045; Jiangsu Guanyun, 58047; Jiangsu Suining, 58130; Jiangsu Suyu, 58131; Jiangsu Siyang, 58132; Jiangsu Sihong, 58135; Jiangsu Xuyi, 58138; Jiangsu Hongze, 58139; Jiangsu Lianshui, 58140; Jiangsu Huaian, 58141; Jiangsu Funing, 58143; Jiangsu Jianhu, 58146; Jiangsu Jinhu, 58147; Jiangsu Baoying, 58148; Jiangsu Yancheng, 58154; Jiangsu Dafeng, 58158; Jiangsu Liuhe, 58235; Jiangsu Pukou, 58237; Jiangsu Nanjing, 58238; Jiangsu Gaoyou, 58241; Jiangsu Yizheng, 58242; Jiangsu Xinghua, 58243; Jiangsu Taizhou, 58246; Jiangsu Yangzhong, 58247; Jiangsu Taixing, 58249; Jiangsu Jiangyan, 58250; Jiangsu Dongtai, 58251; Jiangsu Haian, 58254; Jiangsu Rugao, 58255; Jiangsu Jingjiang, 58257; Jiangsu Nantong, 58259; Jiangsu Rudong, 58264; Jiangsu Lvsi, 58265; Jiangsu Qidong, 58269; Jiangsu Gaochun, 58339; Jiangsu Lishui, 58340; Jiangsu Danyang, 58341; Jiangsu Jintan, 58342; Jiangsu Jurong, 58344; Jiangsu

	Yixing, 58346; Jiangsu Suzhou, 58349; Jiangsu Changshu, 58352; Jiangsu Zhangjiagang, 58353; Jiangsu Wuxi, 58354; Jiangsu Kunshan, 58356; Jiangsu Haimen, 58360; Jiangsu Taicang, 58377<br>9. Jiangxi Tonggu, 57694; Jiangxi Pingxiang, 57786; Jiangxi Lianhua, 57789; Jiangxi Shangli, 57783<br>10. Shandong Xinxian, 54808; Shandong Yutai, 54907; Shandong Caoxian, 58002; Shandong Chengwu, 58003; Shandong Xuechen, 58021; Shandong Yicheng, 58022<br>11. Shanxi Lingchuan, 53981<br>12. Shaanxi Langao, 57247; Shaanxi Baihe, 57254; Shaanxi Zhenping, 57343<br>13. Shanghai Minhang, 58361; Shanghai Baoshan, 58362; Shanghai Fengxian, 58463<br>14. Sichuan Linshui, 57416; Sichuan Wusheng, 57417<br>15. Yunnan Yiliang, 56594<br>16. Zhejiang Changxing, 58443<br>17. Chongqing Kaixian, 57338; Chongqing Yunyang, 57339; Chongqing Wuxi, 57345; Chongqing Wushan, 57349; Chongqing Dianjiang, 57425; Chongqing Wanzhou, 57432; Chongqing Zhongxian, 57437; Chongqing Shizhu, 57438; Chongqing Wansheng, 57509; Chongqing Tongliang, 57510; Chongqing Beibei, 57511; Chongqing Yubei, 57513; Chongqing Bishan, 57514; Chongqing Jiangjin, 57517; Chongqing Banan, 57518; Chongqing Nanchuan, 57519; Chongqing Changshou, 57520; Chongqing Fengdu, 57523; Chongqing Wulong, 57525; Chongqing Qianjiang, 57536; Chongqing Pengshui, 57537; Chongqing Qijiang, 57612
Cluster C	1. Anhui Qimen, 58520; Anhui Tunxi, 58531<br>2. Fujian Guangze, 58724; Fujian Shaowu, 58725; Fujian Wuyishan, 58730; Fujian Pucheng, 58731; Fujian Jianyang, 58734; Fujian Songxi, 58735; Fujian Zhenghe, 58736; Fujian Shouning, 58744; Fujian Zhouning, 58747; Fujian Fuan, 58748; Fujian Zherong, 58749; Fujian Fuding, 58754; Fujian Ninghuai, 58818; Fujian Taining, 58820; Fujian Jianning, 58822; Fujian Shunchang, 58823; Fujian Mingxi, 58824; Fujian Sanming, 58828; Fujian Gutian, 58836; Fujian Youxi, 58837; Fujian Shuqing, 58839; Fujian Xiapu, 58843; Fujian Minhou, 58844; Fujian Ningde, 58846; Fujian Lianjiang, 58848; Fujian Changting, 58911; Fujian Wuping, 58917; Fujian Datian, 58923; Fujian Zhangping, 58926; Fujian Huaan, 58928; Fujian Anxi, 58929; Fujian Jiuxianshan, 58931; Fujian Yongtai, 58932; Fujian Xiuyu, 58938; Fujian Changle, 58941; Fujian Fuqing, 58942; Fujian Pingtan, 58944; Fujian Putian, 58946; Fujian Changtai, 59122; Fujian Nanjing, 59124; Fujian Pinghe, 59125; Fujian Zhangpu, 59129; Fujian Tongan, 59130; Fujian Nanan, 59131; Fujian Chongwu, 59133<br>3. Hubei Wuxue, 58501<br>4. Hunan Lanshan, 57975<br>5. Jiangxi Xiushui, 57598; Jiangxi Wanzai, 57698; Jiangxi Shanggao, 57699; Jiangxi Fenyi, 57792; Jiangxi Yichun, 57793; Jiangxi Xinyu, 57796; Jiangxi Anfu, 57798; Jiangxi Jianxian, 57799; Jiangxi Xiaping, 57883; Jiangxi Yongxin, 57891; Jiangxi Wanan, 57895; Jiangxi Suichuan, 57896; Jiangxi Taihe, 57899; Jiangxi Chongyi,

	57990; Jiangxi Ganxian, 57993; Jiangxi Jiujiang, 58502; Jiangxi Ruichang, 58503; Jiangxi Lushan, 58506; Jiangxi Wuning, 58507; Jiangxi Dean, 58508; Jiangxi Hukou, 58510; Jiangxi Pengze, 58512; Jiangxi Poyang, 58519; Jiangxi Jingdezhen, 58527; Jiangxi Wuyuan, 58529; Jiangxi Jingan, 58600; Jiangxi Fengxin, 58601; Jiangxi Anyi, 58602; Jiangxi Gaoan, 58605; Jiangxi Nanchang, 58606; Jiangxi Yugan, 58612; Jiangxi Jinxian, 58614; Jiangxi Wannian, 58615; Jiangxi Dongxiang, 58618; Jiangxi Linchuan, 58619; Jiangxi Dexing, 58622; Jiangxi Shangraoxian, 58623; Jiangxi Guixi, 58626; Jiangxi Qianshan, 58629; Jiangxi Yushan, 58634; Jiangxi Xinjian, 58693; Jiangxi Xiajiang, 58704; Jiangxi Yongfeng, 58705; Jiangxi Lean, 58706; Jiangxi Chongren, 58710; Jiangxi Jinxi, 58712; Jiangxi Nancheng, 58715; Jiangxi Nanfeng , 58718; Jiangxi Lichuan, 58719; Jiangxi Xingguo, 58804; Jiangxi Ningdu, 58806; Jiangxi Guangchang, 58813; Jiangxi Shicheng, 58814; Jiangxi Yudu, 58905; Jiangxi Huichang, 58906; Jiangxi Anyuan, 58907; Jiangxi Longnan, 59092<br>6. Shanghai Jinshan, 58460<br>7. Zhejiang Anji, 58446; Zhejiang Linan, 58448; Zhejiang Fuyang, 58449; Zhejiang Huzhou, 58450; Zhejiang Jiashan, 58451; Zhejiang Jiaxing, 58452; Zhejiang Shaoxing, 58453; Zhejiang Deqing, 58454; Zhejiang Hangzhou, 58457; Zhejiang Cixi, 58467; Zhejiang Daishan, 58484; Zhejiang Kaihua, 58537; Zhejiang Tonglu, 58542; Zhejiang Jiande, 58544; Zhejiang Pujiang, 58546; Zhejiang Longyou, 58547; Zhejiang Jinhua, 58549; Zhejiang Zhuji, 58550; Zhejiang Xinchang, 58555; Zhejiang Shengzhou, 58556; Zhejiang Dongyang, 58558; Zhejiang Tiantai, 58559; Zhejiang Panan, 58560; Zhejiang Zhenhai, 58561; Zhejiang Yinzhou, 58562; Zhejiang Fenghua, 58565; Zhejiang Xiangshan, 58566; Zhejiang Ninghai, 58567; Zhejiang Sanmen, 58568; Zhejiang Shipu, 58569; Zhejiang Putuo, 58570; Zhejiang Changshan, 58631; Zhejiang Jiangshan, 58632; Zhejiang Wuyi, 58642; Zhejiang Yongkang, 58643; Zhejiang Suichang, 58644; Zhejiang Lishui, 58646; Zhejiang Longquan, 58647; Zhejiang Xianju, 58652; Zhejiang Jinyun, 58654; Zhejiang Leqing, 58656; Zhejiang Qingtian, 58657; Zhejiang Yongjia, 58658; Zhejiang Linhai, 58660; Zhejiang Wenling, 58664; Zhejiang Hongjia, 58665; Zhejiang Dachen, 58666; Zhejiang Yunhe, 58742; Zhejiang Taishun, 58746; Zhejiang Wencheng, 58750; Zhejiang Pingyang, 58751; Zhejiang Jingning, 58648
Cluster D	1. Hebei Shangyi, 53397<br>2. Heilongjiang Mohe, 50136; Heilongjiang Tahe, 50246; Heilongjiang Huzhong, 50247; Heilongjiang Xinlin, 50349; Heilongjiang Huma, 50353; Heilongjiang Jiagedaqi, 50442; Heilongjiang Nenjiang, 50557; Heilongjiang Sunwu, 50564; Heilongjiang Xunke, 50566; Heilongjiang Nehe, 50646; Heilongjiang Wudalianchi, 50655; Heilongjiang Beian, 50656; Heilongjiang Keshan, 50658; Heilongjiang Kedong, 50659; Heilongjiang Wuyiling, 50674; Heilongjiang Longjiang, 50739; Heilongjiang Gannan, 50741; Heilongjiang Fuyu, 50742; Heilongjiang Qiqihaer, 50745; Heilongjiang Yian, 50750; Heilongjiang Baiquan, 50755; Heilongjiang Hailun, 50756; Heilongjiang Minshui, 50758; Heilongjiang Suileng, 50767; Heilongjiang Wuying, 50772; Heilongjiang Yichun, 50774;

	Heilongjiang Hegang, 50775; Heilongjiang Luobei, 50776; Heilongjiang Tongjiang, 50778; Heilongjiang Suibin, 50787; Heilongjiang Fujin, 50788; Heilongjiang Dumeng, 50842; Heilongjiang Daqing, 50850; Heilongjiang Qinggang, 50851; Heilongjiang Wangkui, 50852; Heilongjiang Beilin, 50853; Heilongjiang Anda, 50854; Heilongjiang Zhaodong, 50858; Heilongjiang Lansi, 50859; Heilongjiang Qingan, 50861; Heilongjiang Tieli, 50862; Heilongjiang Bayan, 50867; Heilongjiang Tangyuan, 50871; Heilongjiang Jiamusi, 50873; Heilongjiang Yilang, 50877; Heilongjiang Huachuan, 50878; Heilongjiang Huanan, 50879; Heilongjiang Shuangyashan, 50884; Heilongjiang Baoqing, 50888; Heilongjiang Zhaozhou, 50950; Heilongjiang Haerbin, 50953; Heilongjiang Hulan, 50956; Heilongjiang Acheng, 50958; Heilongjiang Binxian, 50960; Heilongjiang Mulan, 50962; Heilongjiang Tonghe, 50963; Heilongjiang Zhengfang, 50964; Heilongjiang Yanshou, 50965; Heilongjiang Shangzhi, 50968; Heilongjiang Boli, 50973; Heilongjiang Jixi, 50978; Heilongjiang Linkou, 50979; Heilongjiang Hailin, 54092; Heilongjiang Muling, 54093; Heilongjiang Mudanjiang, 54094; Heilongjiang Ningan, 54098<br>3. Jilin Shuangliao, 54142<br>4. Inner Mongolia Eerguna, 50425; Inner Mongolia Elunchunqi, 50445; Inner Mongolia Manzhouli, 50514; Inner Mongolia Yakeshi, 50526; Inner Mongolia Xiaoergou, 50548; Inner Mongolia Xinbaerhuzuoqi, 50618; Inner Mongolia Moulidawawoer, 50645; Inner Mongolia Arunqi, 50647; Inner Mongolia Suolun, 50834; Inner Mongolia Wuzhumuqindong, 50915; Inner Mongolia Bayaertuhushuo, 50928; Inner Mongolia Tuquan, 50934; Inner Mongolia Erlianhaote, 53068; Inner Mongolia Mandula, 53149; Inner Mongolia Abagaqi, 53192; Inner Mongolia Xianghuangqi, 53289; Inner Mongolia Wulatezhongqi, 53336; Inner Mongolia Wuyuan, 53337; Inner Mongolia Dashetai, 53348; Inner Mongolia Damaoqi, 53352; Inner Mongolia Guyangxian, 53357; Inner Mongolia Siziwang, 53362; Inner Mongolia Xilamuren, 53367; Inner Mongolia Wuchuanxian, 53368; Inner Mongolia Chayouzhongqi, 53378; Inner Mongolia Chayouhouqi, 53384; Inner Mongolia Shangdu, 53385; Inner Mongolia Huade, 53391; Inner Mongolia Dengkou, 53419; Inner Mongolia Hangjinhouqi , 53420; Inner Mongolia Baotou, 53446; Inner Mongolia Tuyouqi, 53455; Inner Mongolia Dalateqi, 53457; Inner Mongolia Huhehaote, 53463; Inner Mongolia Tumutezuoqi, 53464; Inner Mongolia Huhehaote suburb, 53466; Inner Mongolia Tuoketuoxian, 53467; Inner Mongolia Helingeerxian, 53469; Inner Mongolia Zhuozi, 53472; Inner Mongolia Liangcheng, 53475; Inner Mongolia Jining, 53480; Inner Mongolia Xinghe, 53483; Inner Mongolia Wuhai, 53512; Inner Mongolia Linhe, 53513; Inner Mongolia Yikewusu, 53522; Inner Mongolia Etukeqi, 53529; Inner Mongolia Hangjinqi, 53533; Inner Mongolia Dongsheng, 53543; Inner Mongolia Ejinhuoluoqi, 53545; Inner Mongolia Zhungeerqi, 53553; Inner Mongolia Qingshuihexian, 53562; Inner Mongolia Wushenqi, 53644; Inner Mongolia Eduokeqianqi, 53730; Inner Mongolia Xiwuzhumuqin, 54012; Inner Mongolia Fuhe, 54024; Inner Mongolia Zhalute, 54026; Inner Mongolia Balinzuoqi, 54027; Inner Mongolia Gaoliban, 54031; Inner Mongolia

	Shebotu, 54039; Inner Mongolia Kezuozhongqi, 54047; Inner Mongolia Xilinhaote, 54102; Inner Mongolia Balinyouqi, 54113; Inner Mongolia Linxixian, 54115; Inner Mongolia Keshiketengqi, 54117; Inner Mongolia Alukeerqinqi, 54122; Inner Mongolia Kailu, 54134; Inner Mongolia Tongliao, 54135; Inner Mongolia Zhenglanqi, 54205; Inner Mongolia Duolunxian, 54208; Inner Mongolia Wengniuteqi, 54213; Inner Mongolia Gangzi, 54214; Inner Mongolia Chifeng, 54218; Inner Mongolia Neiman, 54223; Inner Mongolia Baoguotu, 54226; Inner Mongolia Kalaqinqi, 54313<br>5. Ningxia Shitanjing, 53517<br>6. Tibet Naqu, 55299; Tibet Dangxiong, 55493; Tibet Lazi, 55569; Tibet Nanmulin, 55572; Tibet Muozhugongka, 55593; Tibet Jiangzi, 55680; Tibet Leiwuqi, 56128; Tibet Miling, 56317
Cluster E	1. Fujian Yongding, 59113; Fujian Zhaoan, 59320; Fujian Yunxiao, 59322<br>2. Guangdong Lechang, 57988; Guangdong Renhua, 57989; Guangdong Nanxiong, 57996; Guangdong Lianshan, 59074; Guangdong Yangshan, 59075; Guangdong Ruyuan, 59081; Guangdong Shaoguan, 59082; Guangdong Fogang, 59087; Guangdong Yingde, 59088; Guangdong Shixing, 59090; Guangdong Wengyuan, 59094; Guangdong Lianping, 59096; Guangdong Heping, 59099; Guangdong Pingyuan, 59106; Guangdong Longchuan, 59107; Guangdong Xingning, 59109; Guangdong Jiaoling, 59114; Guangdong Daipu, 59116; Guangdong Meixian, 59117; Guangdong Yunan, 59268; Guangdong Huaiji, 59270; Guangdong Guangning, 59271; Guangdong Sihui, 59276; Guangdong Sanshui, 59279; Guangdong Qingyuan, 59280; Guangdong Huadu, 59284; Guangdong Conghua, 59285; Guangdong Guangzhou, 59287; Guangdong Dongguan, 59289; Guangdong Longmen, 59290; Guangdong Heyuan, 59293; Guangdong Boluo, 59297; Guangdong Wuhua, 59303; Guangdong Zijin, 59304; Guangdong Jiexi, 59306; Guangdong Fengshun, 59310; Guangdong Chaozhou, 59312; Guangdong Raoping, 59313; Guangdong Puning, 59314; Guangdong Shantou, 59316; Guangdong Xinyi, 59456; Guangdong Luoding, 59462; Guangdong Yangchun, 59469; Guangdong Xinxing, 59470; Guangdong Yunfu, 59471; Guangdong Heshan, 59473; Guangdong Kaiping, 59475; Guangdong Xinhui, 59476; Guangdong Enping, 59477; Guangdong Panyu, 59481; Guangdong Doumen, 59487; Guangdong Zhuhai, 59488; Guangdong Huidong, 59492; Guangdong Shenzhen, 59493; Guangdong Haifeng, 59500; Guangdong Lufeng, 59502; Guangdong Suixi, 59650; Guangdong Gaozhou, 59653; Guangdong Lianjiang, 59654; Guangdong Huazhou, 59655; Guangdong Wuchuan, 59656; Guangdong Maoming, 59659; Guangdong Leizhou, 59750; Guangdong Xuwen, 59754<br>3. Guangxi Tiane, 57927; Guangxi Sanjiang, 57941; Guangxi Rongshui, 57948; Guangxi Yongfu, 57949; Guangxi Lingui, 57954; Guangxi Xingan, 57955; Guangxi Quanzhou, 57960; Guangxi Guanyang, 57964; Guangxi Xilin, 59004; Guangxi Leye, 59012; Guangxi Lingyun, 59015; Guangxi Tianlin, 59017; Guangxi Fengshan, 59021; Guangxi Hechi, 59023; Guangxi Bama, 59027; Guangxi Yizhou, 59034; Guangxi Duan, 59037; Guangxi Xincheng, 59038; Guangxi

	Liucheng, 59041; Guangxi Luzhai, 59045; Guangxi Pingle, 59053; Guangxi Lipu, 59055; Guangxi Jinxiu, 59057; Guangxi Mengshan, 59058; Guangxi Hezhou, 59065; Guangxi Baise, 59211; Guangxi Debao, 59215; Guangxi Jingxi, 59218; Guangxi Tiandong, 59224; Guangxi Tiandeng, 59227; Guangxi Longan, 59229; Guangxi Mashan, 59230; Guangxi Shanglin, 59235; Guangxi Wuming , 59237; Guangxi Binyang, 59238; Guangxi Xiangzhou, 59241; Guangxi Laibin, 59242; Guangxi Wuxuan, 59246; Guangxi Pingnan, 59255; Guangxi Tengxian, 59256; Guangxi Wuzhou, 59265; Guangxi Cangwu, 59266; Guangxi Daxin, 59421; Guangxi Shangsi, 59429; Guangxi Nanning, 59431; Guangxi Yongning, 59435; Guangxi Hengxian, 59441; Guangxi Lingshan, 59446; Guangxi Pubei, 59448; Guangxi Bobai, 59449; Guangxi Beiliu, 59451; Guangxi Rongxian, 59452; Guangxi Cenxi, 59454; Guangxi Luchuan, 59457; Guangxi Qinzhou, 59632; Guangxi Hepu, 59640<br>4. Guizhou Ceheng, 57909; Guizhou Pingtang, 57921; Guizhou Libo, 57926; Guizhou Congjiang, 57936<br>5. Hunan Dongan, 57867; Hunan Chenzhou, 57972; Hunan Yizhang, 57976; Hunan Linwu, 57978; Hunan Rucheng, 57985<br>6. Jiangxi Nankang, 57992; Jiangxi Xinfeng, 57995; Jiangxi Quannan, 59091; Jiangxi Dingnan, 59093; Jiangxi Xunwu, 59102
Cluster F	1. Guizhou Panxian, 56793; Guizhou Xingyi, 57907<br>2. Sichuan Derong, 56441; Sichuan Pingshan, 56494; Sichuan Jinyang, 56584; Sichuan Panzhihua, 56666<br>3. Yunnan Yanjin, 56497; Yunnan Xianggelila, 56543; Yunnan Weixi, 56548; Yunnan Ninglang, 56567; Yunnan Ludian, 56585; Yunnan Weixin, 56596; Yunnan Liuku, 56643; Yunnan Lanping, 56645; Yunnan Jianchuan, 56646; Yunnan Eryuan, 56649; Yunnan Lijiang, 56651; Yunnan Yongsheng, 56652; Yunnan Heqing, 56654; Yunnan Huaping, 56664; Yunnan Yongren, 56669; Yunnan Dongchuan, 56688; Yunnan Xuanwei, 56697; Yunnan Tengchong, 56739; Yunnan Yunlong, 56742; Yunnan Yangbi, 56745; Yunnan Yongping, 56746; Yunnan Baoshan, 56748; Yunnan Dali, 56751; Yunnan Binchuan, 56752; Yunnan Midu, 56755; Yunnan Weishan, 56757; Yunnan Yaoan, 56764; Yunnan Mouding, 56766; Yunnan Nanhua, 56767; Yunnan Fumin, 56772; Yunnan Wuding, 56774; Yunnan Lufeng, 56777; Yunnan Kunming, 56778; Yunnan Malong, 56782; Yunnan Qujing, 56783; Yunnan Songming, 56785; Yunnan Fuyuan, 56790; Yunnan Lianghe, 56840; Yunnan Longling, 56841; Yunnan Shidian, 56842; Yunnan Changning, 56843; Yunnan Fengqing, 56846; Yunnan Yongde, 56849; Yunnan Yunxian, 56854; Yunnan Jingdong, 56856; Yunnan Shuangbai, 56862; Yunnan Anning, 56863; Yunnan Zhenyuan, 56867; Yunnan Xinping, 56869; Yunnan Yimen, 56870; Yunnan Jinning, 56871; Yunnan Chengjiang, 56873; Yunnan Yuxi, 56875; Yunnan Huaning, 56879; Yunnan Yiliang, 56880; Yunnan Shilin, 56881; Yunnan Shizong, 56883; Yunnan Mile, 56885; Yunnan Luxi, 56886; Yunnan Qiubei, 56889; Yunnan Luoping, 56891; Yunnan Eshan, 56898; Yunnan Cangyuan, 56944; Yunnan Gengma, 56946; Yunnan Ximeng, 56948; Yunnan Menglian, 56949; Yunnan Shuangjiang, 56950; Yunnan Lincang, 56951; Yunnan

	Jinggu, 56952; Yunnan Lancang, 56954; Yunnan Menghai, 56958; Yunnan Mojiang, 56962; Yunnan Simao, 56964; Yunnan Yuanjiang, 56966; Yunnan Mengla, 56969; Yunnan Shiping, 56970; Yunnan Yuanyang, 56976; Yunnan Luchun, 56978; Yunnan Kaiyuan, 56982; Yunnan Gejiu, 56984; Yunnan Yanshan, 56991; Yunnan Xichou, 56992; Yunnan Wenshan, 56994; Yunnan Guangnan, 59007
Cluster G	1. Guangxi Napo, 59209; Guangxi Pingxiang, 59419<br>2. Heilongjiang Beijicun, 50137; Heilongjiang Aihui, 50468; Heilongjiang Jiayin, 50673; Heilongjiang Fuyuan, 50779; Heilongjiang Raohe, 50892; Heilongjiang Qitaihe, 50971; Heilongjiang Hulin, 50983; Heilongjiang Mishan, 50985; Heilongjiang Jidong, 50987; Heilongjiang Suifenhe, 54096<br>3. Jilin Longjing, 54290; Jilin Huichun, 54291; Jilin Linjiang, 54374; Jilin Changbai, 54386<br>4. Tibet Nielamu, 55655<br>5. Xinjiang Habahe, 51053; Xinjiang Tacheng, 51133; Xinjiang Yumin, 51137; Xinjiang Alashankou, 51232<br>6. Yunnan Gongshan, 56533; Yunnan Fugong, 56641; Yunnan Longchuan, 56835; Yunnan Yingjiang, 56836; Yunnan Zhenkang, 56839; Yunnan Jiangcheng, 56977; Yunnan Jinping, 56987; Yunnan Funing, 59205
Cluster H	1. Hainan Haikou, 59758; Hainan Lingao, 59842; Hainan Chengmai, 59843; Hainan Danzhou, 59845; Hainan Changjiang, 59847; Hainan Baisha, 59848; Hainan Dingan, 59851; Hainan Tunchang, 59854; Hainan Qonghai, 59855; Hainan Wenchang, 59856; Hainan Baoting, 59945

Table A7. Merging process of SO_2 emissions from power generation, industrial, residential and transportation sectors in 2010 produced by R package.

merge	[,1]	[,2]				
[1,]	-1	-17	Anhui Dangshan	Anhui Funan	58015	58202
[2,]	-501	1	Henan Mianchi	Anhui Dangshan	57063	58015
[3,]	-540	2	Henan Runan	Anhui Xiaoxian	57197	58016
[4,]	-551	3	Henan Guangshan	Anhui Bozhou	57299	58102
[5,]	-561	4	Henan Gushi	Anhui Jieshou	58208	58108
[6,]	-2	-3	Anhui Xiaoxian	Anhui Bozhou	58016	58102
[7,]	-4	6	Anhui Jieshou	Anhui Tianzhushan	58108	58112
[8,]	-414	7	Hebei Fengfeng	Anhui Suixi	53894	58113
[9,]	-415	8	Hebei Weixian	Anhui Woyang	53896	58114
[10,]	-480	9	Hebei Daming	Anhui Leysin	54804	58117
[11,]	-484	10	Henan Qixian	Anhui Mengcheng	53974	58118
[12,]	-485	11	Henan Jiyuan	Anhui Suzhou	53978	58122
[13,]	-490	12	Henan Xinxiang	Anhui Lingbi	53986	58125
[14,]	-492	13	Henan Junxian	Anhui Sixian	53992	58126
[15,]	-497	14	Henan Qingfeng	Anhui Huaiyuan	54902	58127
[16,]	-498	15	Henan Fanxian	Anhui Guzhen	54903	58128
[17,]	-503	16	Henan Xinan	Anhui Wuhe	57070	58129
[18,]	-504	17	Henan Mengjin	Anhui Funan	57071	58202
[19,]	-506	18	Henan Yichuan	Anhui Fuyang	57074	58203
[20,]	-507	19	Henan Ruzhou	Anhui Yingshang	57075	58210
[21,]	-509	20	Henan Wenxian	Anhui Fengtai	57079	58212
[22,]	-511	21	Henan Xingyang	Anhui Huoqiu	57081	58214
[23,]	-514	22	Henan Xuchang	Anhui Changfeng	57089	58220
[24,]	-515	23	Henan Zhongmou	Anhui Fengyang	57090	58222
[25,]	-516	24	Henan Kaifeng	Anhui Mingguang	57091	58223
[26,]	-517	25	Henan Lankao	Anhui Dingyuan	57093	58225
[27,]	-518	26	Henan Yanling	Anhui Laian	57095	58234
[28,]	-519	27	Henan Qixian	Anhui Chuzhou	57096	58236
[29,]	-520	28	Henan Taikang	Anhui Tianchang	57099	58240
[30,]	-521	29	Henan Xixia	Anhui Jinzhai	57156	58306
[31,]	-522	30	Henan Neixiang	Anhui Luan	57169	58311
[32,]	-524	31	Henan Zhenping	Anhui Huoshan	57175	58314
[33,]	-526	32	Henan Wugang	Anhui Shucheng	57177	58316
[34,]	-527	33	Henan Nanyang	Anhui Yuexi	57178	58317
[35,]	-533	34	Henan Wuyang	Anhui Tongcheng	57185	58319
[36,]	-536	35	Henan Xiping	Anhui Feixi	57188	58320
[37,]	-537	36	Henan Suiping	Anhui Feidong	57189	58323
[38,]	-548	37	Henan Zhengyang	Anhui Chaohu	57295	58326
[39,]	-556	38	Henan Yucheng	Anhui Lujiang	58006	58327

[40,]	-558	39	Henan Xiayi	Anhui Wuwei	58017	58329
[41,]	-559	40	Henan Dancheng	Anhui Hanshan	58100	58330
[42,]	-5	-6	Anhui Taihe	Anhui Tianzhushan	58109	58112
[43,]	-8	42	Anhui Woyang	Anhui Maanshan	58114	58336
[44,]	-9	43	Anhui Leysin	Anhui Wuhuxian	58117	58338
[45,]	-10	44	Anhui Mengcheng	Anhui Susong	58118	58417
[46,]	-11	45	Anhui Suzhou	Anhui Tongling	58122	58429
[47,]	-12	46	Anhui Lingbi	Anhui Nanling	58125	58431
[48,]	-13	47	Anhui Sixian	Anhui Jingxian	58126	58432
[49,]	-14	48	Anhui Huaiyuan	Anhui Xuancheng	58127	58433
[50,]	-15	49	Anhui Guzhen	Anhui Jingde	58128	58435
[51,]	-16	50	Anhui Wuhe	Anhui Ningguo	58129	58436
[52,]	-19	51	Anhui Yingshang	Anhui Huangshan	58210	58437
[53,]	-20	52	Anhui Fengtai	Anhui Guangde	58212	58441
[54,]	-21	53	Anhui Huoqiu	Anhui Langxi	58214	58442
[55,]	-22	54	Anhui Changfeng	Anhui Qimen	58220	58520
[56,]	-23	55	Anhui Fengyang	Anhui Yixian	58222	58523
[57,]	-24	56	Anhui Mingguang	Anhui Shexian	58223	58530
[58,]	-25	57	Anhui Dingyuan	Anhui Tunxi	58225	58531
[59,]	-26	58	Anhui Laian	Beijing Shunyi	58234	54398
[60,]	-27	59	Anhui Chuzhou	Beijing Haidian	58236	54399
[61,]	-30	60	Anhui Luan	Beijing Yanqing	58311	54406
[62,]	-32	61	Anhui Shucheng	Beijing Miyun	58316	54416
[63,]	-33	62	Anhui Yuexi	Beijing Miyunshangdianzi	58317	54421
[64,]	-34	63	Anhui Tongcheng	Beijing Pinggu	58319	54424
[65,]	-35	64	Anhui Feixi	Beijing Tongzhou	58320	54431
[66,]	-36	65	Anhui Feidong	Beijing Changping	58323	54499
[67,]	-37	66	Anhui Chaohu	Beijing Zhaitang	58326	54501
[68,]	-38	67	Anhui Lujiang	Beijing	58327	54511
[69,]	-39	68	Anhui Wuwei	Beijing Xiayunling	58329	54597
[70,]	-40	69	Anhui Hanshan	Fujian Guangze	58330	58724
[71,]	-41	70	Anhui Wuhu	Fujian Shaowu	58334	58725
[72,]	-42	71	Anhui Maanshan	Fujian Wuyishan	58336	58730
[73,]	-43	72	Anhui Wuhuxian	Fujian Pucheng	58338	58731
[74,]	-45	73	Anhui Tongling	Fujian Jianyang	58429	58734
[75,]	-46	74	Anhui Nanling	Fujian Songxi	58431	58735
[76,]	-47	75	Anhui Jingxian	Fujian Zhenghe	58432	58736
[77,]	-48	76	Anhui Xuancheng	Fujian Shouning	58433	58744
[78,]	-49	77	Anhui Jingde	Fujian Zhouning	58435	58747
[79,]	-50	78	Anhui Ningguo	Fujian Fuan	58436	58748
[80,]	-51	79	Anhui Huangshan	Fujian Zherong	58437	58749
[81,]	-862	80	Jiangsu Gaochun	Fujian Fuding	58339	58754
[82,]	-7	-29	Anhui Suixi	Anhui Jinzhai	58113	58306

[83,]	-31	82	Anhui Huoshan	Fujian Taining	58314	58820
[84,]	-44	83	Anhui Susong	Fujian Jianning	58417	58822
[85,]	-52	84	Anhui Guangde	Fujian Shunchang	58441	58823
[86,]	-53	85	Anhui Langxi	Fujian Mingxi	58442	58824
[87,]	-56	86	Anhui Shexian	Fujian Sanming	58530	58828
[88,]	-819	87	Jiangsu Fengxian	Fujian Gutian	58012	58836
[89,]	-820	88	Jiangsu Peixian	Fujian Youxi	58013	58837
[90,]	-830	89	Jiangsu Suining	Fujian Shuqing	58130	58839
[91,]	-834	90	Jiangsu Xuyi	Fujian Xiapu	58138	58843
[92,]	-845	91	Jiangsu Pukou	Fujian Minhou	58237	58844
[93,]	-846	92	Jiangsu Nanjing	Fujian Ningde	58238	58846
[94,]	-867	93	Jiangsu Yixing	Fujian Lianjiang	58346	58848
[95,]	-18	-55	Anhui Fuyang	Anhui Yixian	58203	58523
[96,]	-28	-822	Anhui Tianchang	Jiangsu Xuzhou	58240	58027
[97,]	-823	96	Jiangsu Donghai	Fujian Datian	58036	58923
[98,]	-825	97	Jiangsu Ganyu	Fujian Zhangping	58040	58926
[99,]	-842	98	Jiangsu Yancheng	Fujian Huaan	58154	58928
[100,]	-843	99	Jiangsu Dafeng	Fujian Anxi	58158	58929
[101,]	-847	100	Jiangsu Gaoyou	Fujian Jiuxianshan	58241	58931
[102,]	-850	101	Jiangsu Taizhou	Fujian Yongtai	58246	58932
[103,]	-852	102	Jiangsu Taixing	Fujian Xiuyu	58249	58938
[104,]	-854	103	Jiangsu Dongtai	Fujian Changle	58251	58941
[105,]	-855	104	Jiangsu Haian	Fujian Fuqing	58254	58942
[106,]	-856	105	Jiangsu Rugao	Fujian Pingtan	58255	58944
[107,]	-857	106	Jiangsu Jingjiang	Fujian Putian	58257	58946
[108,]	-859	107	Jiangsu Rudong	Fujian Yongding	58264	59113
[109,]	-865	108	Jiangsu Jintan	Fujian Changtai	58342	59122
[110,]	-869	109	Jiangsu Changshu	Fujian Nanjing	58352	59124
[111,]	-870	110	Jiangsu Zhangjiagang	Fujian Pinghe	58353	59125
[112,]	-872	111	Jiangsu Kunshan	Fujian Zhangpu	58356	59129
[113,]	-873	112	Jiangsu Haimen	Fujian Tongan	58360	59130
[114,]	-1204	113	Shandong Yicheng	Fujian Nanan	58022	59131
[115,]	-54	-57	Anhui Qimen	Anhui Tunxi	58520	58531
[116,]	-69	115	Fujian Guangze	Fujian Zhaoan	58724	59320
[117,]	-701	116	Hubei Wuxue	Fujian Yunxiao	58501	59322
[118,]	-875	117	Jiangxi Xiushui	Gansu Mazongshan	57598	52323
[119,]	-881	118	Jiangxi Fenyi	Gansu Subei	57792	52515
[120,]	-882	119	Jiangxi Yichun	Gansu Jiuquan	57793	52533
[121,]	-883	120	Jiangxi Xinyu	Gansu Gaitai	57796	52546
[122,]	-884	121	Jiangxi Anfu	Gansu Linze	57798	52557
[123,]	-885	122	Jiangxi Jianxian	Gansu Sunan	57799	52643
[124,]	-886	123	Jiangxi Xiaping	Gansu Minle	57883	52656
[125,]	-887	124	Jiangxi Yongxin	Gansu Yongchang	57891	52674

[126,]	-888	125	Jiangxi Wanan	Gansu Wuwei	57895	52679
[127,]	-889	126	Jiangxi Suichuan	Gansu Minqin	57896	52681
[128,]	-890	127	Jiangxi Taihe	Gansu Gulang	57899	52784
[129,]	-891	128	Jiangxi Chongyi	Gansu Wushaoling	57990	52787
[130,]	-896	129	Jiangxi Ruichang	Gansu Jingtai	58503	52797
[131,]	-897	130	Jiangxi Lushan	Gansu Tianzhu	58506	52881
[132,]	-898	131	Jiangxi Wuning	Gansu Gaolan	58507	52884
[133,]	-899	132	Jiangxi Dean	Gansu Yongdeng	58508	52885
[134,]	-900	133	Jiangxi Hukou	Gansu Jingyuan	58510	52895
[135,]	-901	134	Jiangxi Pengze	Gansu Baiyin	58512	52896
[136,]	-903	135	Jiangxi Poyang	Gansu Xiahe	58519	52978
[137,]	-904	136	Jiangxi Jingdezhen	Gansu Yongjing	58527	52980
[138,]	-905	137	Jiangxi Wuyuan	Gansu Dongxiang	58529	52981
[139,]	-906	138	Jiangxi Jingan	Gansu Guanghe	58600	52982
[140,]	-907	139	Jiangxi Fengxin	Gansu Yuzhong	58601	52983
[141,]	-909	140	Jiangxi Gaoan	Gansu Hezheng	58605	52985
[142,]	-911	141	Jiangxi Yugan	Gansu Lintao	58612	52986
[143,]	-912	142	Jiangxi Jinxian	Gansu Kangle	58614	52988
[144,]	-916	143	Jiangxi Dexing	Gansu Huining	58622	52993
[145,]	-917	144	Jiangxi Shangraoxian	Gansu Anding	58623	52995
[146,]	-919	145	Jiangxi Qianshan	Gansu Huajialing	58629	52996
[147,]	-922	146	Jiangxi Xiajiang	Gansu Weiyuan	58704	52998
[148,]	-924	147	Jiangxi Lean	Gansu Huanxian	58706	53821
[149,]	-925	148	Jiangxi Chongren	Gansu Qingcheng	58710	53829
[150,]	-926	149	Jiangxi Jinxi	Gansu Jingning	58712	53906
[151,]	-927	150	Jiangxi Nancheng	Gansu Kongtong	58715	53915
[152,]	-928	151	Jiangxi Nanfeng	Gansu Zhuanglang	58718	53917
[153,]	-929	152	Jiangxi Lichuan	Gansu Xifeng	58719	53923
[154,]	-930	153	Jiangxi Xingguo	Gansu Lingtai	58804	53924
[155,]	-931	154	Jiangxi Ningdu	Gansu Zhenyuan	58806	53925
[156,]	-932	155	Jiangxi Guangchang	Gansu Jingchuan	58813	53926
[157,]	-933	156	Jiangxi Shicheng	Gansu Huating	58814	53927
[158,]	-934	157	Jiangxi Yudu	Gansu Huachi	58905	53930
[159,]	-935	158	Jiangxi Huichang	Gansu Huishui	58906	53934
[160,]	-936	159	Jiangxi Anyuan	Gansu Zhengning	58907	53935
[161,]	-938	160	Jiangxi Longnan	Gansu Luqu	59092	56071
[162,]	-58	-59	Beijing Shunyi	Beijing Haidian	54398	54399
[163,]	-63	162	Beijing Pinggu	Gansu Hezuo	54424	56080
[164,]	-65	163	Beijing Changping	Gansu Lintan	54499	56081
[165,]	-60	-66	Beijing Yanqing	Beijing Zhaitang	54406	54501
[166,]	-68	165	Beijing Xiayunling	Gansu Dibu	54597	56084
[167,]	-384	166	Hebei Kangbao	Gansu Zhangxian	53392	56091
[168,]	-386	167	Hebei Zhangbei	Gansu Longxi	53399	56092

[169,]	-389	168	Hebei Xuanhua	Gansu Minxian	53498	56093
[170,]	-390	169	Hebei Wanquan	Gansu Zhouqu	53499	56094
[171,]	-392	170	Hebei Shunping	Gansu Dangchang	53596	56095
[172,]	-398	171	Hebei Fuping	Gansu Wudu	53690	56096
[173,]	-400	172	Hebei Dingzhou	Gansu Wenxian	53696	56192
[174,]	-405	173	Hebei Luancheng	Gansu Gangu	53789	57001
[175,]	-407	174	Hebei Zanhuang	Gansu Qinan	53795	57002
[176,]	-416	175	Hebei Guyuan	Gansu Wushan	54301	57004
[177,]	-418	176	Hebei Fengning	Gansu Tianshui	54308	57006
[178,]	-419	177	Hebei Weichang	Gansu Lixian	54311	57007
[179,]	-420	178	Hebei Longhua	Gansu Qingshui	54318	57011
[180,]	-421	179	Hebei Pingquan	Gansu Zhangjiachuan	54319	57012
[181,]	-422	180	Hebei Zhangjiakou	Gansu Maiji	54401	57014
[182,]	-423	181	Hebei Chicheng	Gansu Chengxian	54404	57102
[183,]	-424	182	Hebei Huailai	Gansu Kangxian	54405	57105
[184,]	-425	183	Hebei Zhulu	Gansu Huixian	54408	57110
[185,]	-429	184	Hebei Zunhua	Gansu Liangdang	54429	57111
[186,]	-430	185	Hebei Chengdexian	Guangdong Lechang	54430	57988
[187,]	-432	186	Hebei Qianxi	Guangdong Renhua	54434	57989
[188,]	-434	187	Hebei Luannan	Guangdong Nanxiong	54437	57996
[189,]	-436	188	Hebei Qianan	Guangdong Lianshan	54439	59074
[190,]	-437	189	Hebei Qinhuangdao	Guangdong Yangshan	54449	59075
[191,]	-440	190	Hebei Gaobeidian	Guangdong Ruyuan	54506	59081
[192,]	-444	191	Hebei Sanhe	Guangdong Shaoguan	54520	59082
[193,]	-450	192	Hebei Changli	Guangdong Fogang	54540	59087
[194,]	-454	193	Hebei Anxin	Guangdong Yingde	54605	59088
[195,]	-459	194	Hebei Dacheng	Guangdong Shixing	54613	59090
[196,]	-460	195	Hebei Hejian	Guangdong Wengyuan	54614	59094
[197,]	-461	196	Hebei Qingxian	Guangdong Lianping	54615	59096
[198,]	-462	197	Hebei Cangzhou	Guangdong Heping	54616	59099
[199,]	-463	198	Hebei Botou	Guangdong Pingyuan	54618	59106
[200,]	-464	199	Hebei Huanghua	Guangdong Longchuan	54624	59107
[201,]	-467	200	Hebei Xinhe	Guangdong Xingning	54633	59109
[202,]	-469	201	Hebei Wuqiang	Guangdong Jiaoling	54700	59114
[203,]	-470	202	Hebei Xinji	Guangdong Daipu	54701	59116
[204,]	-471	203	Hebei Hengshui	Guangdong Meixian	54702	59117
[205,]	-475	204	Hebei Fucheng	Guangdong Yunan	54710	59268
[206,]	-965	205	Liaoning Jianchang	Guangdong Huaiji	54452	59270
[207,]	-1133	206	Shandong Ningjin	Guangdong Guangning	54716	59271
[208,]	-1177	207	Shandong Rushan	Guangdong Sihui	54861	59276
[209,]	-1187	208	Shandong Yanzhou	Guangdong Sanshui	54916	59279
[210,]	-1474	209	Tianjin Jinghai	Guangdong Qingyuan	54619	59280
[211,]	-1475	210	Tianjin Jinnan	Guangdong Huadu	54622	59284

[212,]	-61	-64	Beijing Miyun	Beijing Tongzhou	54416	54431
[213,]	-426	212	Hebei Luanping	Guangdong Guangzhou	54420	59287
[214,]	-67	-441	Beijing	Hebei Dachang	54511	54510
[215,]	-70	-71	Fujian Shaowu	Fujian Wuyishan	58725	58730
[216,]	-72	215	Fujian Pucheng	Guangdong Heyuan	58731	59293
[217,]	-74	216	Fujian Songxi	Guangdong Boluo	58735	59297
[218,]	-75	217	Fujian Zhenghe	Guangdong Wuhua	58736	59303
[219,]	-78	218	Fujian Fuan	Guangdong Zijin	58748	59304
[220,]	-79	219	Fujian Zherong	Guangdong Jiexi	58749	59306
[221,]	-80	220	Fujian Fuding	Guangdong Fengshun	58754	59310
[222,]	-82	221	Fujian Taining	Guangdong Chaozhou	58820	59312
[223,]	-86	222	Fujian Sanming	Guangdong Raoping	58828	59313
[224,]	-87	223	Fujian Gutian	Guangdong Puning	58836	59314
[225,]	-88	224	Fujian Youxi	Guangdong Shantou	58837	59316
[226,]	-89	225	Fujian Shuqing	Guangdong Xinyi	58839	59456
[227,]	-91	226	Fujian Minhou	Guangdong Luoding	58844	59462
[228,]	-92	227	Fujian Ningde	Guangdong Yangchun	58846	59469
[229,]	-93	228	Fujian Lianjiang	Guangdong Xinxing	58848	59470
[230,]	-95	229	Fujian Wuping	Guangdong Yunfu	58917	59471
[231,]	-96	230	Fujian Datian	Guangdong Heshan	58923	59473
[232,]	-97	231	Fujian Zhangping	Guangdong Kaiping	58926	59475
[233,]	-98	232	Fujian Huaan	Guangdong Xinhui	58928	59476
[234,]	-99	233	Fujian Anxi	Guangdong Enping	58929	59477
[235,]	-100	234	Fujian Jiuxianshan	Guangdong Panyu	58931	59481
[236,]	-102	235	Fujian Xiuyu	Guangdong Doumen	58938	59487
[237,]	-103	236	Fujian Changle	Guangdong Zhuhai	58941	59488
[238,]	-104	237	Fujian Fuqing	Guangdong Huidong	58942	59492
[239,]	-105	238	Fujian Pingtan	Guangdong Shenzhen	58944	59493
[240,]	-106	239	Fujian Putian	Guangdong Haifeng	58946	59500
[241,]	-108	240	Fujian Changtai	Guangdong Lufeng	59122	59502
[242,]	-109	241	Fujian Nanjing	Guangdong Suixi	59124	59650
[243,]	-112	242	Fujian Tongan	Guangdong Gaozhou	59130	59653
[244,]	-113	243	Fujian Nanan	Guangdong Lianjiang	59131	59654
[245,]	-114	244	Fujian Chongwu	Guangdong Huazhou	59133	59655
[246,]	-1715	245	Zhejiang Wencheng	Guangdong Wuchuan	58750	59656
[247,]	-73	-77	Fujian Jianyang	Fujian Zhouning	58734	58747
[248,]	-83	247	Fujian Jianning	Guangdong Leizhou	58822	59750
[249,]	-84	248	Fujian Shunchang	Guangdong Xuwen	58823	59754
[250,]	-85	249	Fujian Mingxi	Guangxi Ziyuan	58824	57859
[251,]	-90	250	Fujian Xiapu	Guangxi Tiane	58843	57927
[252,]	-94	251	Fujian Changting	Guangxi Sanjiang	58911	57941
[253,]	-101	252	Fujian Yongtai	Guangxi Longsheng	58932	57942
[254,]	-111	253	Fujian Zhangpu	Guangxi Rongshui	59129	57948

[255,]	-1714	254	Zhejiang Taishun	Guangxi Yongfu	58746	57949
[256,]	-107	-115	Fujian Yongding	Fujian Zhaoan	59113	59320
[257,]	-186	256	Guangdong Renhua	Guangxi Xingan	57989	57955
[258,]	-187	257	Guangdong Nanxiong	Guangxi Quanzhou	57996	57960
[259,]	-190	258	Guangdong Ruyuan	Guangxi Guanyang	59081	57964
[260,]	-191	259	Guangdong Shaoguan	Guangxi Xilin	59082	59004
[261,]	-192	260	Guangdong Fogang	Guangxi Leye	59087	59012
[262,]	-195	261	Guangdong Wengyuan	Guangxi Lingyun	59094	59015
[263,]	-196	262	Guangdong Lianping	Guangxi Tianlin	59096	59017
[264,]	-197	263	Guangdong Heping	Guangxi Fengshan	59099	59021
[265,]	-199	264	Guangdong Longchuan	Guangxi Hechi	59107	59023
[266,]	-200	265	Guangdong Xingning	Guangxi Bama	59109	59027
[267,]	-203	266	Guangdong Meixian	Guangxi Yizhou	59117	59034
[268,]	-205	267	Guangdong Huaiji	Guangxi Duan	59270	59037
[269,]	-206	268	Guangdong Guangning	Guangxi Xincheng	59271	59038
[270,]	-207	269	Guangdong Sihui	Guangxi Liucheng	59276	59041
[271,]	-208	270	Guangdong Sanshui	Guangxi Luzhai	59279	59045
[272,]	-209	271	Guangdong Qingyuan	Guangxi Pingle	59280	59053
[273,]	-210	272	Guangdong Huadu	Guangxi Lipu	59284	59055
[274,]	-211	273	Guangdong Conghua	Guangxi Jinxiu	59285	59057
[275,]	-213	274	Guangdong Dongguan	Guangxi Mengshan	59289	59058
[276,]	-215	275	Guangdong Heyuan	Guangxi Hezhou	59293	59065
[277,]	-216	276	Guangdong Boluo	Guangxi Napo	59297	59209
[278,]	-219	277	Guangdong Jiexi	Guangxi Baise	59306	59211
[279,]	-220	278	Guangdong Fengshun	Guangxi Debao	59310	59215
[280,]	-221	279	Guangdong Chaozhou	Guangxi Jingxi	59312	59218
[281,]	-222	280	Guangdong Raoping	Guangxi Tiandong	59313	59224
[282,]	-223	281	Guangdong Puning	Guangxi Tiandeng	59314	59227
[283,]	-224	282	Guangdong Shantou	Guangxi Longan	59316	59229
[284,]	-229	283	Guangdong Yunfu	Guangxi Mashan	59471	59230
[285,]	-230	284	Guangdong Heshan	Guangxi Shanglin	59473	59235
[286,]	-231	285	Guangdong Kaiping	Guangxi Wuming	59475	59237
[287,]	-232	286	Guangdong Xinhui	Guangxi Binyang	59476	59238
[288,]	-234	287	Guangdong Panyu	Guangxi Xiangzhou	59481	59241
[289,]	-235	288	Guangdong Doumen	Guangxi Laibin	59487	59242
[290,]	-236	289	Guangdong Zhuhai	Guangxi Wuxuan	59488	59246
[291,]	-237	290	Guangdong Huidong	Guangxi Pingnan	59492	59255
[292,]	-238	291	Guangdong Shenzhen	Guangxi Tengxian	59493	59256
[293,]	-240	292	Guangdong Lufeng	Guangxi Wuzhou	59502	59265
[294,]	-241	293	Guangdong Suixi	Guangxi Cangwu	59650	59266
[295,]	-242	294	Guangdong Gaozhou	Guangxi Pingxiang	59653	59419
[296,]	-243	295	Guangdong Lianjiang	Guangxi Daxin	59654	59421
[297,]	-244	296	Guangdong Huazhou	Guangxi Shangsi	59655	59429

[298,]	-245	297	Guangdong Wuchuan	Guangxi Nanning	59656	59431
[299,]	-246	298	Guangdong Maoming	Guangxi Yongning	59659	59435
[300,]	-247	299	Guangdong Leizhou	Guangxi Hengxian	59750	59441
[301,]	-937	300	Jiangxi Quannan	Guangxi Lingshan	59091	59446
[302,]	-939	301	Jiangxi Dingnan	Guangxi Pubei	59093	59448
[303,]	-117	-118	Gansu Mazongshan	Gansu Subei	52323	52515
[304,]	-119	303	Gansu Jiuquan	Guangxi Beiliu	52533	59451
[305,]	-164	304	Gansu Zhuoni	Guangxi Rongxian	56082	59452
[306,]	-985	305	Inner Mongolia Tulihe	Guangxi Cenxi	50434	59454
[307,]	-991	306	Inner Mongolia Xinbaerhuyouqi	Guangxi Luchuan	50603	59457
[308,]	-993	307	Inner Mongolia Zhalantun	Guangxi Qinzhou	50639	59632
[309,]	-996	308	Inner Mongolia Aershan	Guangxi Hepu	50727	59640
[310,]	-998	309	Inner Mongolia Wulagai	Guizhou Hezhang	50913	56598
[311,]	-1000	310	Inner Mongolia Houlinguole	Guizhou Weining	50924	56691
[312,]	-1007	311	Inner Mongolia Hailisu	Guizhou Puan	53231	56792
[313,]	-1023	312	Inner Mongolia Wulateqianqi	Guizhou Panxian	53433	56793
[314,]	-1097	313	Qinghai Lenghu	Guizhou Tongzi	52602	57606
[315,]	-1110	314	Qinghai Xinghai	Guizhou Daozhen	52943	57623
[316,]	-1118	315	Qinghai Zhiduo	Guizhou Zhengan	56016	57625
[317,]	-1119	316	Qinghai Zaduo	Guizhou Wuchuan	56018	57634
[318,]	-1120	317	Qinghai Qumacai	Guizhou Yanhe	56021	57636
[319,]	-1128	318	Qinghai Jiuzhi	Guizhou Dejiang	56067	57637
[320,]	-1505	319	Xinjiang Fuyun	Guizhou Songtao	51087	57647
[321,]	-1509	320	Xinjiang Hebukesaier	Guizhou Bijie	51156	57707
[322,]	-1512	321	Xinjiang Touli	Guizhou Dafang	51241	57708
[323,]	-1522	322	Xinjiang Tianshandaxigou	Guizhou Jinsha	51468	57714
[324,]	-1526	323	Xinjiang Mulei	Guizhou Zunyi	51482	57717
[325,]	-1531	324	Xinjiang Tulufandongkan	Guizhou Xifeng	51572	57718
[326,]	-1545	325	Xinjiang Alaer	Guizhou Kaiyang	51730	57719
[327,]	-1547	326	Xinjiang Tieqianlike	Guizhou Meitan	51765	57722
[328,]	-1558	327	Xinjiang Minfeng	Guizhou Fenggang	51839	57723
[329,]	-1560	328	Xinjiang Yutian	Guizhou Wengan	51931	57728
[330,]	-1563	329	Xinjiang Yiwu	Guizhou Yuqing	52118	57729
[331,]	-1565	330	Xinjiang Hongliuhe	Guizhou Sinan	52313	57731
[332,]	-120	-121	Gansu Gaitai	Gansu Linze	52546	52557
[333,]	-124	332	Gansu Yongchang	Guizhou Cengong	52674	57735
[334,]	-129	333	Gansu Jingtai	Guizhou Jiangkou	52797	57736
[335,]	-133	334	Gansu Jingyuan	Guizhou Shibing	52895	57737
[336,]	-134	335	Gansu Baiyin	Guizhou Yuping	52896	57739

[337,]	-152	336	Gansu Xifeng	Guizhou Wanshan	53923	57742
[338,]	-161	337	Gansu Maqu	Guizhou Nayong	56074	57800
[339,]	-176	338	Gansu Tianshui	Guizhou Xianxi	57006	57803
[340,]	-179	339	Gansu Zhangjiachuan	Guizhou Zhijin	57012	57805
[341,]	-1080	340	Ningxia Huinong	Guizhou Anshun	53519	57806
[342,]	-1213	341	Shanxi Pinglu	Guizhou Liuzhi	53574	57807
[343,]	-1216	342	Shanxi Ningwu	Guizhou Xiuwen	53577	57811
[344,]	-1218	343	Shanxi Daixian	Guizhou Pingba	53579	57814
[345,]	-1220	344	Shanxi Yingxian	Guizhou Fuquan	53584	57821
[346,]	-1221	345	Shanxi Fanshi	Guizhou Huangping	53585	57822
[347,]	-1224	346	Shanxi Linxian	Guizhou Guiding	53659	57824
[348,]	-1225	347	Shanxi Kelan	Guizhou Kaili	53662	57825
[349,]	-1226	348	Shanxi Wuzhai	Guizhou Duyun	53663	57827
[350,]	-1227	349	Shanxi Xingxian	Guizhou Sansui	53664	57832
[351,]	-1228	350	Shanxi Lanxian	Guizhou Taijiang	53665	57834
[352,]	-1229	351	Shanxi Jingle	Guizhou Jianhe	53666	57835
[353,]	-1230	352	Shanxi Yuanping	Guizhou Leishan	53673	57837
[354,]	-1231	353	Shanxi Xinfu	Guizhou Liping	53674	57839
[355,]	-1233	354	Shanxi Jiancaoping	Guizhou Tianzhu	53677	57840
[356,]	-1235	355	Shanxi Wutaixian	Guizhou Jinping	53681	57844
[357,]	-1236	356	Shanxi Yuxian	Guizhou Qinglong	53685	57900
[358,]	-1237	357	Shanxi Pingding	Guizhou Guanling	53687	57903
[359,]	-1243	358	Shanxi Zhongyang	Guizhou Zhenfeng	53767	57905
[360,]	-1244	359	Shanxi Xiaoyi	Guizhou Wangmo	53768	57906
[361,]	-1246	360	Shanxi Taigu	Guizhou Xingyi	53775	57907
[362,]	-1247	361	Shanxi Pingyao	Guizhou Ceheng	53778	57909
[363,]	-1248	362	Shanxi Shouyang	Guizhou Ziyun	53780	57910
[364,]	-1249	363	Shanxi Yangquan	Guizhou Huishui	53782	57912
[365,]	-1250	364	Shanxi Zuoquan	Guizhou Longli	53786	57913
[366,]	-1251	365	Shanxi Yushe	Guizhou Luodian	53787	57916
[367,]	-1254	366	Shanxi Xixian	Guizhou Pingtang	53853	57921
[368,]	-1256	367	Shanxi Jiaokou	Guizhou Dushan	53860	57922
[369,]	-1258	368	Shanxi Lingshi	Guizhou Sandu	53862	57923
[370,]	-1260	369	Shanxi Puxian	Guizhou Libo	53864	57926
[371,]	-1263	370	Shanxi Wuxiang	Guizhou Rongjiang	53871	57932
[372,]	-1264	371	Shanxi Qinxian	Guizhou Congjiang	53872	57936
[373,]	-1265	372	Shanxi Changzi	Hainan Haikou	53873	59758
[374,]	-1267	373	Shanxi Qinyuan	Hainan Lingao	53875	59842
[375,]	-1268	374	Shanxi Anze	Hainan Chengmai	53877	59843
[376,]	-1276	375	Shanxi Xinjiang	Hainan Danzhou	53964	59845
[377,]	-1280	376	Shanxi Qingshui	Hainan Changjiang	53970	59847
[378,]	-1537	377	Xinjiang Kuerle	Hainan Baisha	51656	59848
[379,]	-122	-123	Gansu Sunan	Gansu Minle	52643	52656

[380,]	-125	379	Gansu Wuwei	Hainan Tunchang	52679	59854
[381,]	-126	380	Gansu Minqin	Hainan Qonghai	52681	59855
[382,]	-127	381	Gansu Gulang	Hainan Wenchang	52784	59856
[383,]	-128	382	Gansu Wushaoling	Hainan Baoting	52787	59945
[384,]	-130	383	Gansu Tianzhu	Hainan Wanning	52881	59951
[385,]	-132	384	Gansu Yongdeng	Hebei Kangbao	52885	53392
[386,]	-139	385	Gansu Yuzhong	Hebei Shangyi	52983	53397
[387,]	-140	386	Gansu Hezheng	Hebei Zhangbei	52985	53399
[388,]	-142	387	Gansu Kangle	Hebei Huaian	52988	53491
[389,]	-143	388	Gansu Huining	Hebei Yangyuan	52993	53492
[390,]	-144	389	Gansu Anding	Hebei Xuanhua	52995	53498
[391,]	-145	390	Gansu Huajialing	Hebei Wanquan	52996	53499
[392,]	-147	391	Gansu Huanxian	Hebei Weixian	53821	53593
[393,]	-153	392	Gansu Lingtai	Hebei Shunping	53924	53596
[394,]	-155	393	Gansu Jingchuan	Hebei Laiyuan	53926	53599
[395,]	-156	394	Gansu Huating	Hebei Lingshou	53927	53680
[396,]	-160	395	Gansu Luqu	Hebei Quyang	56071	53682
[397,]	-163	396	Gansu Lintan	Hebei Xingtang	56081	53688
[398,]	-166	397	Gansu Zhangxian	Hebei Jinzhou	56091	53689
[399,]	-170	398	Gansu Dangchang	Hebei Fuping	56095	53690
[400,]	-171	399	Gansu Wudu	Hebei Tangxian	56096	53692
[401,]	-173	400	Gansu Gangu	Hebei Dingzhou	57001	53696
[402,]	-175	401	Gansu Wushan	Hebei Wuji	57004	53699
[403,]	-178	402	Gansu Qingshui	Hebei Linzhang	57011	53773
[404,]	-180	403	Gansu Maiji	Hebei Shahe	57014	53781
[405,]	-181	404	Gansu Chengxian	Hebei Baixiang	57102	53785
[406,]	-182	405	Gansu Kangxian	Hebei Luancheng	57105	53789
[407,]	-981	406	Liaoning Changhai	Hebei Longyao	54579	53794
[408,]	-988	407	Inner Mongolia Evenkeqi	Hebei Zanhuang	50525	53795
[409,]	-1004	408	Inner Mongolia Narenbaolige	Hebei Ningjin	53083	53796
[410,]	-1075	409	Inner Mongolia Taibushiqi	Hebei Julu	54305	53799
[411,]	-1077	410	Inner Mongolia Balihan	Hebei Shexian	54316	53886
[412,]	-1078	411	Inner Mongolia Ningchengxian	Hebei Wuan	54320	53890
[413,]	-1087	412	Ningxia Zhongning	Hebei Handan	53705	53892
[414,]	-1088	413	Ningxia Xingren	Hebei Quzhou	53707	53893
[415,]	-1089	414	Ningxia Yanchi	Hebei Fengfeng	53723	53894
[416,]	-1090	415	Ningxia Maihuangshan	Hebei Weixian	53727	53896
[417,]	-1092	416	Ningxia Tongxin	Hebei Guyuan	53810	54301
[418,]	-1093	417	Ningxia Guyuan	Hebei Chongli	53817	54304
[419,]	-1095	418	Ningxia Xiji	Hebei Fengning	53903	54308

[420,]	-1096	419	Ningxia Liupanshan	Hebei Weichang	53910	54311
[421,]	-1098	420	Qinghai Nuomuhong	Hebei Longhua	52825	54318
[422,]	-1099	421	Qinghai Doulan	Hebei Pingquan	52836	54319
[423,]	-1100	422	Qinghai Qinghaihu 151	Hebei Zhangjiakou	52854	54401
[424,]	-1101	423	Qinghai Gonghe	Hebei Chicheng	52856	54404
[425,]	-1103	424	Qinghai Xining	Hebei Huailai	52866	54405
[426,]	-1105	425	Qinghai Pingan	Hebei Zhulu	52875	54408
[427,]	-1108	426	Qinghai Wudaoliang	Hebei Luanping	52908	54420
[428,]	-1109	427	Qinghai Shazhuyu	Hebei Chengde	52941	54423
[429,]	-1111	428	Qinghai Guinan	Hebei Xinglong	52955	54425
[430,]	-1112	429	Qinghai Tongde	Hebei Zunhua	52957	54429
[431,]	-1115	430	Qinghai Xunhua	Hebei Chengdexian	52972	54430
[432,]	-1116	431	Qinghai Tongren	Hebei Kuancheng	52974	54432
[433,]	-1117	432	Qinghai Tuotuohe	Hebei Qianxi	56004	54434
[434,]	-1121	433	Qinghai Yushu	Hebei Qinglong	56029	54436
[435,]	-1123	434	Qinghai Qingshuihe	Hebei Luannan	56034	54437
[436,]	-1124	435	Qinghai Maqin	Hebei Lulong	56043	54438
[437,]	-1125	436	Qinghai Gander	Hebei Qianan	56045	54439
[438,]	-1126	437	Qinghai Dari	Hebei Qinhuangdao	56046	54449
[439,]	-1297	438	Shaanxi Zhidan	Hebei Zhuozhou	53832	54502
[440,]	-1502	439	Xinjiang Buerjin	Hebei Rongcheng	51060	54503
[441,]	-1508	440	Xinjiang Emin	Hebei Gaobeidian	51145	54506
[442,]	-1511	441	Xinjiang Bole	Hebei Dachang	51238	54510
[443,]	-1513	442	Xinjiang Kelamayi	Hebei Guan	51243	54512
[444,]	-1514	443	Xinjiang Jinghe	Hebei Yongqing	51334	54519
[445,]	-1515	444	Xinjiang Shawan	Hebei Sanhe	51357	54520
[446,]	-1523	445	Xinjiang Urumqi Pastoral Test Station	Hebei Xianghe	51469	54521
[447,]	-1525	446	Xinjiang Dabancheng	Hebei Yutian	51477	54522
[448,]	-1527	447	Xinjiang Kumishi	Hebei Tangshan	51526	54534
[449,]	-1530	448	Xinjiang Tuokexun	Hebei Caofeidian	51571	54535
[450,]	-1532	449	Xinjiang Tulufan	Hebei Leting	51573	54539
[451,]	-1535	450	Xinjiang Xinhe	Hebei Changli	51636	54540
[452,]	-1539	451	Xinjiang Wuqia	Hebei Funing	51705	54541
[453,]	-1541	452	Xinjiang Kashi	Hebei Xushui	51709	54601
[454,]	-1542	453	Xinjiang Yuepuhu	Hebei Goyang	51717	54603
[455,]	-1546	454	Xinjiang Tazhong	Hebei Anxin	51747	54605
[456,]	-1551	455	Xinjiang Yecheng	Hebei Raoyang	51814	54606
[457,]	-1553	456	Xinjiang Pishan	Hebei Shenzhou	51818	54608
[458,]	-1555	457	Xinjiang Moyu	Hebei Renqiu	51827	54610
[459,]	-1559	458	Xinjiang Qiemuo	Hebei Wenan	51855	54612
[460,]	-1561	459	Xinjiang Balikun	Hebei Dacheng	52101	54613
[461,]	-1562	460	Xinjiang Naomaohu	Hebei Hejian	52112	54614

[462,]	-1564	461	Xinjiang Hami	Hebei Qingxian	52203	54615
[463,]	-131	-148	Gansu Gaolan	Gansu Qingcheng	52884	53829
[464,]	-174	463	Gansu Qinan	Hebei Botou	57002	54618
[465,]	-135	-136	Gansu Xiahe	Gansu Yongjing	52978	52980
[466,]	-138	465	Gansu Guanghe	Hebei Haixing	52982	54628
[467,]	-141	466	Gansu Lintao	Hebei Guangzong	52986	54631
[468,]	-146	467	Gansu Weiyuan	Hebei Xinhe	52998	54633
[469,]	-149	468	Gansu Jingning	Hebei Jize	53906	54640
[470,]	-162	469	Gansu Hezuo	Hebei Wuqiang	56080	54700
[471,]	-165	470	Gansu Dibu	Hebei Xinji	56084	54701
[472,]	-167	471	Gansu Longxi	Hebei Hengshui	56092	54702
[473,]	-168	472	Gansu Minxian	Hebei Wuyi	56093	54703
[474,]	-177	473	Gansu Lixian	Hebei Qinghe	57007	54706
[475,]	-387	474	Hebei Huaian	Hebei Gucheng	53491	54707
[476,]	-391	475	Hebei Weixian	Hebei Fucheng	53593	54710
[477,]	-482	476	Henan Linzhou	Hebei Jingxian	53889	54711
[478,]	-1064	477	Inner Mongolia Zhengxiangbaiqi	Hebei Dongguang	54204	54713
[479,]	-1074	478	Inner Mongolia Kulun	Hebei-Weixian	54234	54800
[480,]	-1094	479	Ningxia Weizhou	Hebei Linxi	53881	54801
[481,]	-1208	480	Shanxi Youyu	Hebei Daming	53478	54804
[482,]	-1214	481	Shanxi Shenchi	Hebei Guantao	53575	54809
[483,]	-1217	482	Shanxi Shuozhou	Henan Linzhou	53578	53889
[484,]	-1219	483	Shanxi Hunyuan	Henan Qinyang	53582	53972
[485,]	-1222	484	Shanxi Wutaishan	Henan Qixian	53588	53974
[486,]	-1223	485	Shanxi Guangling	Henan Jiyuan	53590	53978
[487,]	-1234	486	Shanxi Xiaodian	Henan Jiaozuo	53679	53982
[488,]	-1262	487	Shanxi Hongtong	Henan Fengqiu	53866	53983
[489,]	-1266	488	Shanxi Guxian	Henan Xiuwu	53874	53984
[490,]	-1270	489	Shanxi Lucheng	Henan Huixian	53880	53985
[491,]	-1272	490	Shanxi Jishan	Henan Xinxiang	53954	53986
[492,]	-1279	491	Shanxi Yuanqu	Henan Tangyin	53968	53991
[493,]	-1501	492	Xinjiang Akedala	Henan Junxian	51058	53992
[494,]	-1516	493	Xinjiang Manasi	Henan Neihuang	51359	53993
[495,]	-1517	494	Xinjiang Hutubi	Henan Changheng	51367	53998
[496,]	-1521	495	Xinjiang Jimusaer	Henan Taiqian	51378	54817
[497,]	-1528	496	Xinjiang Bayinbuluke	Henan Puyang	51542	54900
[498,]	-1533	497	Xinjiang Wushi	Henan Qingfeng	51627	54902
[499,]	-1543	498	Xinjiang Kepin	Henan Fanxian	51720	54903
[500,]	-1548	499	Xinjiang Yengjisha	Henan Sanmenxia	51802	57051
[501,]	-1549	500	Xinjiang Maigaiti	Henan Lingbao	51810	57056
[502,]	-1550	501	Xinjiang Shashe	Henan Mianchi	51811	57063
[503,]	-1552	502	Xinjiang Zepu	Henan Luoning	51815	57066

[504,]	-1554	503	Xinjiang Cele	Henan Xinan	51826	57070
[505,]	-1556	504	Xinjiang Hetan	Henan Mengjin	51828	57071
[506,]	-1557	505	Xinjiang Luopu	Henan Mengzhou	51829	57072
[507,]	-137	-150	Gansu Dongxiang	Gansu Kongtong	52981	53915
[508,]	-1086	507	Ningxia Zhongwei	Henan Ruzhou	53704	57075
[509,]	-157	-1091	Gansu Huachi	Ningxia Haiyuan	53930	53806
[510,]	-158	-1107	Gansu Huishui	Qinghai Hualong	53934	52877
[511,]	-159	-1238	Gansu Zhengning	Shanxi Liulin	53935	53753
[512,]	-169	-172	Gansu Zhouqu	Gansu Wenxian	56094	56192
[513,]	-1130	512	Qinghai Banma	Henan Dengfeng	56151	57082
[514,]	-1356	513	Sichuan Shiqu	Henan Changge	56038	57087
[515,]	-1357	514	Sichuan Ruoergai	Henan Xuchang	56079	57089
[516,]	-1358	515	Sichuan Jiuzhaigou	Henan Zhongmou	56097	57090
[517,]	-1360	516	Sichuan Ganzi	Henan Kaifeng	56146	57091
[518,]	-1362	517	Sichuan Seda	Henan Lankao	56152	57093
[519,]	-1363	518	Sichuan Luhuo	Henan Yanling	56158	57095
[520,]	-1364	519	Sichuan Rangtang	Henan Qixian	56164	57096
[521,]	-1365	520	Sichuan Daofu	Henan Taikang	56167	57099
[522,]	-1366	521	Sichuan Jinchuan	Henan Xixia	56168	57156
[523,]	-1367	522	Sichuan Aba	Henan Neixiang	56171	57169
[524,]	-1368	523	Sichuan Maerkang	Henan Lushan	56172	57173
[525,]	-1369	524	Sichuan Hongyuan	Henan Zhenping	56173	57175
[526,]	-1370	525	Sichuan Xiaojin	Henan Nanzhao	56178	57176
[527,]	-1373	526	Sichuan Songpan	Henan Wugang	56182	57177
[528,]	-1375	527	Sichuan Lixian	Henan Nanyang	56184	57178
[529,]	-1376	528	Sichuan Heishui	Henan Fangcheng	56185	57179
[530,]	-1382	529	Sichuan Batang	Henan Jiaxian	56247	57180
[531,]	-1383	530	Sichuan Xinlong	Henan Xiangcheng	56251	57182
[532,]	-1384	531	Sichuan Litang	Henan Linying	56257	57183
[533,]	-1385	532	Sichuan Danba	Henan Yexian	56263	57184
[534,]	-1386	533	Sichuan Yajiang	Henan Wuyang	56267	57185
[535,]	-1387	534	Sichuan Baoxing	Henan Luohe	56273	57186
[536,]	-1388	535	Sichuan Lushan	Henan Sheqi	56279	57187
[537,]	-1396	536	Sichuan Daocheng	Henan Xiping	56357	57188
[538,]	-1397	537	Sichuan Luding	Henan Suiping	56371	57189
[539,]	-1402	538	Sichuan Hongya	Henan Huaiyang	56380	57192
[540,]	-1414	539	Sichuan Xiangcheng	Henan Xihua	56443	57193
[541,]	-1415	540	Sichuan Muli	Henan Runan	56459	57197
[542,]	-1417	541	Sichuan Ganluo	Henan Xinye	56473	57271
[543,]	-1420	542	Sichuan Xide	Henan Tanghe	56478	57273
[544,]	-1421	543	Sichuan Chaojue	Henan Biyang	56479	57281
[545,]	-1424	544	Sichuan Meigu	Henan Tongbai	56487	57285
[546,]	-1432	545	Sichuan Dechang	Henan Zhumadian	56569	57290

[547,]	-1443	546	Sichuan Qingchuan	Henan Pingyu	57204	57292
[548,]	-1567	547	Yunnan Suijiang	Henan Xincai	56483	57293
[549,]	-183	-446	Gansu Huixian	Hebei Yutian	57110	54522
[550,]	-1310	549	Shaanxi Chengcheng	Henan Xixian	53949	57296
[551,]	-1473	550	Tianjin Baodi	Henan Xinyang	54525	57297
[552,]	-184	-642	Gansu Liangdang	Hubei Zhuxi	57111	57249
[553,]	-1048	552	Inner Mongolia Henan	Henan Jigongshan	53732	57390
[554,]	-1239	553	Shanxi Shilou	Henan Xixian	53759	57396
[555,]	-1253	554	Shanxi Yonghe	Henan Minquan	53852	58004
[556,]	-1255	555	Shanxi Jixian	Henan Shangqiu	53859	58005
[557,]	-1284	556	Shanxi Ruicheng	Henan Yucheng	57053	58006
[558,]	-1286	557	Shaanxi Yulin	Henan Echeng	53646	58007
[559,]	-1287	558	Shaanxi Shenmu	Henan Xiayi	53651	58017
[560,]	-1289	559	Shaanxi Dingbian	Henan Dancheng	53725	58100
[561,]	-1290	560	Shaanxi Jingbian	Henan Huangchuan	53735	58207
[562,]	-1291	561	Shaanxi Wuqi	Henan Gushi	53738	58208
[563,]	-1292	562	Shaanxi Hengshan	Henan Shangcheng	53740	58301
[564,]	-1293	563	Shaanxi Zichang	Heilongjiang Mohe	53748	50136
[565,]	-1294	564	Shaanxi Suide	Heilongjiang Beijicun	53754	50137
[566,]	-1298	565	Shaanxi Ansai	Heilongjiang Tahe	53841	50246
[567,]	-1299	566	Shaanxi Ganquan	Heilongjiang Huzhong	53848	50247
[568,]	-1301	567	Shaanxi Yanchang	Heilongjiang Xinlin	53854	50349
[569,]	-1303	568	Shaanxi Fuxian	Heilongjiang Huma	53931	50353
[570,]	-1304	569	Shaanxi Xunyi	Heilongjiang Jiagedaqi	53938	50442
[571,]	-1305	570	Shaanxi Baishui	Heilongjiang Aihui	53941	50468
[572,]	-1306	571	Shaanxi Huangling	Heilongjiang Nenjiang	53944	50557
[573,]	-1307	572	Shaanxi Huanglong	Heilongjiang Sunwu	53946	50564
[574,]	-1312	573	Shaanxi Longxian	Heilongjiang Xunke	57003	50566
[575,]	-1313	574	Shaanxi Baoji	Heilongjiang Nehe	57016	50646
[576,]	-1316	575	Shaanxi Fufeng	Heilongjiang Wudalianchi	57026	50655
[577,]	-1322	576	Shaanxi Xingping	Heilongjiang Beian	57038	50656
[578,]	-1327	577	Shaanxi Huashan	Heilongjiang Keshan	57046	50658
[579,]	-1329	578	Shaanxi Lueyang	Heilongjiang Kedong	57106	50659
[580,]	-1330	579	Shaanxi Fengxian	Heilongjiang Jiayin	57113	50673
[581,]	-1332	580	Shaanxi Liuba	Heilongjiang Wuyiling	57124	50674
[582,]	-1334	581	Shaanxi Foping	Heilongjiang Longjiang	57134	50739
[583,]	-1335	582	Shaanxi Ningshan	Heilongjiang Gannan	57137	50741
[584,]	-1337	583	Shaanxi Shangxian	Heilongjiang Fuyu	57143	50742
[585,]	-1338	584	Shaanxi Danfeng	Heilongjiang Qiqihaer	57153	50745
[586,]	-1339	585	Shaanxi Shangnan	Heilongjiang Lindian	57154	50749
[587,]	-1340	586	Shaanxi Shanyang	Heilongjiang Yian	57155	50750
[588,]	-1342	587	Shaanxi Nanzheng	Heilongjiang Baiquan	57213	50755
[589,]	-1343	588	Shaanxi Ziyang	Heilongjiang Hailun	57231	50756

[590,]	-1344	589	Shaanxi Hanyin	Heilongjiang Minshui	57233	50758
[591,]	-1345	590	Shaanxi Zhenba	Heilongjiang Suileng	57238	50767
[592,]	-1347	591	Shaanxi Ankang	Heilongjiang Wuying	57245	50772
[593,]	-1349	592	Shaanxi Pingli	Heilongjiang Yichun	57248	50774
[594,]	-188	-189	Guangdong Lianshan	Guangdong Yangshan	59074	59075
[595,]	-193	594	Guangdong Yingde	Heilongjiang Luobei	59088	50776
[596,]	-194	595	Guangdong Shixing	Heilongjiang Tongjiang	59090	50778
[597,]	-198	596	Guangdong Pingyuan	Heilongjiang Fuyuan	59106	50779
[598,]	-202	597	Guangdong Daipu	Heilongjiang Suibin	59116	50787
[599,]	-214	598	Guangdong Longmen	Heilongjiang Fujin	59290	50788
[600,]	-217	599	Guangdong Wuhua	Heilongjiang Dumeng	59303	50842
[601,]	-218	600	Guangdong Zijin	Heilongjiang Tailai	59304	50844
[602,]	-225	601	Guangdong Xinyi	Heilongjiang Daqing	59456	50850
[603,]	-226	602	Guangdong Luoding	Heilongjiang Qinggang	59462	50851
[604,]	-227	603	Guangdong Yangchun	Heilongjiang Wangkui	59469	50852
[605,]	-228	604	Guangdong Xinxing	Heilongjiang Beilin	59470	50853
[606,]	-233	605	Guangdong Enping	Heilongjiang Anda	59477	50854
[607,]	-940	606	Jiangxi Xunwu	Heilongjiang Zhaodong	59102	50858
[608,]	-204	-305	Guangdong Yunan	Guangxi Cenxi	59268	59454
[609,]	-250	-251	Guangxi Tiane	Guangxi Sanjiang	57927	57941
[610,]	-253	609	Guangxi Rongshui	Heilongjiang Tieli	57948	50862
[611,]	-256	610	Guangxi Xingan	Heilongjiang Bayan	57955	50867
[612,]	-257	611	Guangxi Quanzhou	Heilongjiang Tangyuan	57960	50871
[613,]	-260	612	Guangxi Leye	Heilongjiang Jiamusi	59012	50873
[614,]	-261	613	Guangxi Lingyun	Heilongjiang Yilang	59015	50877
[615,]	-263	614	Guangxi Fengshan	Heilongjiang Huachuan	59021	50878
[616,]	-264	615	Guangxi Hechi	Heilongjiang Huanan	59023	50879
[617,]	-265	616	Guangxi Bama	Heilongjiang Shuangyashan	59027	50884
[618,]	-266	617	Guangxi Yizhou	Heilongjiang Baoqing	59034	50888
[619,]	-267	618	Guangxi Duan	Heilongjiang Raohe	59037	50892
[620,]	-268	619	Guangxi Xincheng	Heilongjiang Zhaozhou	59038	50950
[621,]	-272	620	Guangxi Lipu	Heilongjiang Haerbin	59055	50953
[622,]	-273	621	Guangxi Jinxiu	Heilongjiang Hulan	59057	50956
[623,]	-274	622	Guangxi Mengshan	Heilongjiang Acheng	59058	50958
[624,]	-275	623	Guangxi Hezhou	Heilongjiang Binxian	59065	50960
[625,]	-277	624	Guangxi Baise	Heilongjiang Mulan	59211	50962
[626,]	-280	625	Guangxi Tiandong	Heilongjiang Tonghe	59224	50963
[627,]	-281	626	Guangxi Tiandeng	Heilongjiang Zhengfang	59227	50964
[628,]	-282	627	Guangxi Longan	Heilongjiang Yanshou	59229	50965
[629,]	-291	628	Guangxi Tengxian	Heilongjiang Shangzhi	59256	50968
[630,]	-295	629	Guangxi Daxin	Heilongjiang Qitaihe	59421	50971
[631,]	-296	630	Guangxi Shangsi	Heilongjiang Boli	59429	50973

[632,]	-297	631	Guangxi Nanning	Heilongjiang Jixi	59431	50978
[633,]	-299	632	Guangxi Hengxian	Heilongjiang Linkou	59441	50979
[634,]	-300	633	Guangxi Lingshan	Heilongjiang Hulin	59446	50983
[635,]	-301	634	Guangxi Pubei	Heilongjiang Mishan	59448	50985
[636,]	-302	635	Guangxi Bobai	Heilongjiang Jidong	59449	50987
[637,]	-361	636	Guizhou Ceheng	Heilongjiang Wuchang	57909	54080
[638,]	-254	-279	Guangxi Yongfu	Guangxi Jingxi	57949	59218
[639,]	-255	-269	Guangxi Lingui	Guangxi Liucheng	57954	59041
[640,]	-278	639	Guangxi Debao	Heilongjiang Mudanjiang	59215	54094
[641,]	-289	640	Guangxi Wuxuan	Heilongjiang Suifenhe	59246	54096
[642,]	-292	641	Guangxi Wuzhou	Heilongjiang Ningan	59265	54098
[643,]	-308	642	Guangxi Hepu	Hubei Zhuxi	59640	57249
[644,]	-259	-262	Guangxi Xilin	Guangxi Tianlin	59004	59017
[645,]	-270	-271	Guangxi Luzhai	Guangxi Pingle	59045	59053
[646,]	-298	645	Guangxi Yongning	Hubei Shiyan	59435	57256
[647,]	-307	646	Guangxi Qinzhou	Hubei Zhushan	59632	57257
[648,]	-276	-294	Guangxi Napo	Guangxi Pingxiang	59209	59419
[649,]	-283	-284	Guangxi Mashan	Guangxi Shanglin	59230	59235
[650,]	-287	649	Guangxi Xiangzhou	Hubei Gucheng	59241	57268
[651,]	-288	650	Guangxi Laibin	Hubei Xiangyang	59242	57278
[652,]	-293	651	Guangxi Cangwu	Hubei Zaoyang	59266	57279
[653,]	-285	-290	Guangxi Wuming	Guangxi Pingnan	59237	59255
[654,]	-303	653	Guangxi Beiliu	Hubei Xingshan	59451	57359
[655,]	-306	654	Guangxi Luchuan	Hubei Baokang	59457	57361
[656,]	-286	-304	Guangxi Binyang	Guangxi Rongxian	59238	59452
[657,]	-309	-313	Guizhou Hezhang	Guizhou Tongzi	56598	57606
[658,]	-314	657	Guizhou Daozhen	Hubei Yicheng	57623	57370
[659,]	-315	658	Guizhou Zhengan	Hubei Jingmen	57625	57377
[660,]	-316	659	Guizhou Wuchuan	Hubei Zhongxiang	57634	57378
[661,]	-317	660	Guizhou Yanhe	Hubei Suizhou	57636	57381
[662,]	-318	661	Guizhou Dejiang	Hubei Xiaochang	57637	57386
[663,]	-319	662	Guizhou Songtao	Hubei Jingshan	57647	57387
[664,]	-320	663	Guizhou Bijie	Hubei Anlu	57707	57388
[665,]	-321	664	Guizhou Dafang	Hubei Hongan	57708	57398
[666,]	-322	665	Guizhou Jinsha	Hubei Macheng	57714	57399
[667,]	-324	666	Guizhou Xifeng	Hubei Lichuan	57718	57439
[668,]	-325	667	Guizhou Kaiyang	Hubei Jianshi	57719	57445
[669,]	-326	668	Guizhou Meitan	Hubei Enshi	57722	57447
[670,]	-327	669	Guizhou Fenggang	Hubei Yiling	57723	57453
[671,]	-328	670	Guizhou Wengan	Hubei Wufeng	57728	57458
[672,]	-329	671	Guizhou Yuqing	Hubei Songzi	57729	57469
[673,]	-330	672	Guizhou Sinan	Hubei Qianjiang	57731	57475
[674,]	-331	673	Guizhou Shiqian	Hubei Gongan	57734	57477

[675,]	-332	674	Guizhou Cengong	Hubei Yingcheng	57735	57481
[676,]	-333	675	Guizhou Jiangkou	Hubei Xiaogan	57736	57482
[677,]	-334	676	Guizhou Shibing	Hubei Tianmen	57737	57483
[678,]	-335	677	Guizhou Yuping	Hubei Shayang	57739	57484
[679,]	-337	678	Guizhou Nayong	Hubei Xiantao	57800	57485
[680,]	-340	679	Guizhou Anshun	Hubei Hanchuan	57806	57486
[681,]	-341	680	Guizhou Liuzhi	Hubei Caidian	57807	57489
[682,]	-343	681	Guizhou Pingba	Hubei Xinzhou	57814	57492
[683,]	-344	682	Guizhou Fuquan	Hubei Wuhan	57821	57494
[684,]	-346	683	Guizhou Guiding	Hubei Tuanfeng	57824	57495
[685,]	-347	684	Guizhou Kaili	Hubei Ezhou	57825	57496
[686,]	-348	685	Guizhou Duyun	Hubei Xianfeng	57827	57540
[687,]	-349	686	Guizhou Sansui	Hubei Xuanen	57832	57541
[688,]	-350	687	Guizhou Taijiang	Hubei Hefeng	57834	57543
[689,]	-351	688	Guizhou Jianhe	Hubei Shishou	57835	57571
[690,]	-352	689	Guizhou Leishan	Hubei Jianli	57837	57573
[691,]	-353	690	Guizhou Liping	Hubei Honghu	57839	57581
[692,]	-354	691	Guizhou Tianzhu	Hubei Chibi	57840	57582
[693,]	-355	692	Guizhou Jinping	Hubei Jiayu	57844	57583
[694,]	-356	693	Guizhou Qinglong	Hubei Chongyang	57900	57586
[695,]	-357	694	Guizhou Guanling	Hubei Tongcheng	57903	57589
[696,]	-358	695	Guizhou Zhenfeng	Hubei Xianning	57905	57590
[697,]	-359	696	Guizhou Wangmo	Hubei Luotian	57906	58401
[698,]	-362	697	Guizhou Ziyun	Hubei Yingshan	57910	58402
[699,]	-363	698	Guizhou Huishui	Hubei Qichun	57912	58408
[700,]	-365	699	Guizhou Luodian	Hubei Huangmei	57916	58409
[701,]	-367	700	Guizhou Dushan	Hubei Yangxin	57922	58500
[702,]	-368	701	Guizhou Sandu	Hubei Wuxue	57923	58501
[703,]	-370	702	Guizhou Rongjiang	Hunan Longshan	57932	57544
[704,]	-652	703	Hubei Padang	Hunan Sangzhi	57355	57554
[705,]	-727	704	Hunan Fenghuang	Hunan Zhangjiajie	57740	57558
[706,]	-728	705	Hunan Xinhuang	Hunan Lixian	57744	57565
[707,]	-743	706	Hunan Tongtao	Hunan Nanxian	57845	57574
[708,]	-757	707	Hunan Shuangpai	Hunan Huarong	57962	57575
[709,]	-1348	708	Shaanxi Langao	Hunan Yueyang	57247	57584
[710,]	-1351	709	Shaanxi Zhenping	Hunan Baojing	57343	57642
[711,]	-1575	710	Yunnan Yiliang	Hunan Yongshun	56594	57643
[712,]	-1721	711	Chongqing Wuxi	Hunan Guzhang	57345	57646
[713,]	-1722	712	Chongqing Wushan	Hunan Jishou	57349	57649
[714,]	-1726	713	Chongqing Zhongxian	Hunan Yuanling	57437	57655
[715,]	-1727	714	Chongqing Shizhu	Hunan Luxi	57438	57657
[716,]	-1732	715	Chongqing Tongliang	Hunan Taoyuan	57510	57661
[717,]	-1735	716	Chongqing Bishan	Hunan Changde	57514	57662

[718,]	-1736	717	Chongqing Jiangjin	Hunan Hanshou	57517	57663
[719,]	-1737	718	Chongqing Banan	Hunan Taojiang	57518	57666
[720,]	-1739	719	Chongqing Changshou	Hunan Anhua	57520	57669
[721,]	-1741	720	Chongqing Wulong	Hunan Yuanjiang	57525	57671
[722,]	-1742	721	Chongqing Qianjiang	Hunan Xiangyin	57536	57673
[723,]	-1743	722	Chongqing Pengshui	Hunan Ningxiang	57537	57678
[724,]	-1744	723	Chongqing Qijiang	Hunan Milo	57612	57680
[725,]	-311	-323	Guizhou Puan	Guizhou Zunyi	56792	57717
[726,]	-336	725	Guizhou Wanshan	Hunan Changsha	57742	57687
[727,]	-338	726	Guizhou Xianxi	Hunan Liuyang	57803	57688
[728,]	-339	727	Guizhou Zhijin	Hunan Fenghuang	57805	57740
[729,]	-342	728	Guizhou Xiuwen	Hunan Xinhuang	57811	57744
[730,]	-345	729	Guizhou Huangping	Hunan Zhijiang	57822	57745
[731,]	-364	730	Guizhou Longli	Hunan Xupu	57913	57752
[732,]	-312	-360	Guizhou Panxian	Guizhou Xingyi	56793	57907
[733,]	-1413	732	Sichuan Derong	Hunan Lengshuijiang	56441	57760
[734,]	-1428	733	Sichuan Pingshan	Hunan Xinhua	56494	57761
[735,]	-1439	734	Sichuan Panzhihua	Hunan Loudi	56666	57763
[736,]	-1568	735	Yunnan Yanjin	Hunan Shaoshan	56497	57771
[737,]	-1570	736	Yunnan Xianggelila	Hunan Xiangxiang	56543	57772
[738,]	-1571	737	Yunnan Weixi	Hunan Shuangfeng	56548	57774
[739,]	-1572	738	Yunnan Ninglang	Hunan Nanyue	56567	57776
[740,]	-1574	739	Yunnan Ludian	Hunan Hengshan	56585	57777
[741,]	-1577	740	Yunnan Weixin	Hunan Youxian	56596	57779
[742,]	-1579	741	Yunnan Liuku	Hunan Zhuzhou	56643	57780
[743,]	-1580	742	Yunnan Lanping	Hunan Liling	56645	57781
[744,]	-1581	743	Yunnan Jianchuan	Hunan Tongtao	56646	57845
[745,]	-1582	744	Yunnan Eryuan	Hunan Lengshuitan	56649	57865
[746,]	-1583	745	Yunnan Lijiang	Hunan Yongzhou	56651	57866
[747,]	-1584	746	Yunnan Yongsheng	Hunan Dongan	56652	57867
[748,]	-1585	747	Yunnan Heqing	Hunan Qiyang	56654	57868
[749,]	-1586	748	Yunnan Huaping	Hunan Qidong	56664	57870
[750,]	-1587	749	Yunnan Yongren	Hunan Hengyangxian	56669	57871
[751,]	-1589	750	Yunnan Dongchuan	Hunan Hengyang	56688	57872
[752,]	-1591	751	Yunnan Tengchong	Hunan Changning	56739	57874
[753,]	-1592	752	Yunnan Yunlong	Hunan Hengnan	56742	57875
[754,]	-1593	753	Yunnan Yangbi	Hunan Anren	56745	57881
[755,]	-1594	754	Yunnan Yongping	Hunan Chaling	56746	57882
[756,]	-1595	755	Yunnan Baoshan	Hunan Yongxing	56748	57887
[757,]	-1596	756	Yunnan Dali	Hunan Guangxi	56751	57889
[758,]	-1597	757	Yunnan Binchuan	Hunan Shuangpai	56752	57962
[759,]	-1598	758	Yunnan Midu	Hunan Daoxian	56755	57965
[760,]	-1599	759	Yunnan Weishan	Hunan Ningyuan	56757	57966

[761,]	-1600	760	Yunnan Yaoan	Hunan Jiangyong	56764	57969
[762,]	-1601	761	Yunnan Mouding	Hunan Xintian	56766	57971
[763,]	-1602	762	Yunnan Nanhua	Hunan Chenzhou	56767	57972
[764,]	-1603	763	Yunnan Fumin	Hunan Guiyang	56772	57973
[765,]	-1604	764	Yunnan Wuding	Hunan Jiahe	56774	57974
[766,]	-1605	765	Yunnan Lufeng	Hunan Lanshan	56777	57975
[767,]	-1606	766	Yunnan Kunming	Hunan Yizhang	56778	57976
[768,]	-1607	767	Yunnan Malong	Hunan Linwu	56782	57978
[769,]	-1608	768	Yunnan Qujing	Hunan Zixing	56783	57981
[770,]	-1609	769	Yunnan Songming	Hunan Rucheng	56785	57985
[771,]	-1614	770	Yunnan Lianghe	Hunan Jianghua	56840	59063
[772,]	-1615	771	Yunnan Longling	Jilin Baicheng	56841	50936
[773,]	-1616	772	Yunnan Shidian	Jilin Taonan	56842	50939
[774,]	-1617	773	Yunnan Changning	Jilin Zhenlai	56843	50940
[775,]	-1618	774	Yunnan Fengqing	Jilin Daan	56846	50945
[776,]	-1619	775	Yunnan Yongde	Jilin Songyuan	56849	50946
[777,]	-1620	776	Yunnan Yunxian	Jilin Qianan	56854	50948
[778,]	-1621	777	Yunnan Jingdong	Jilin Qianguo	56856	50949
[779,]	-1622	778	Yunnan Shuangbai	Jilin Tongyu	56862	54041
[780,]	-1623	779	Yunnan Anning	Jilin Changling	56863	54049
[781,]	-1624	780	Yunnan Zhenyuan	Jilin Fuyu	56867	54063
[782,]	-1625	781	Yunnan Xinping	Jilin Nongan	56869	54064
[783,]	-1626	782	Yunnan Yimen	Jilin Dehui	56870	54065
[784,]	-1627	783	Yunnan Jinning	Jilin Jiutai	56871	54069
[785,]	-1629	784	Yunnan Yuxi	Jilin Yushu	56875	54072
[786,]	-1630	785	Yunnan Huaning	Jilin Shulan	56879	54076
[787,]	-1631	786	Yunnan Yiliang	Jilin Shuangliao	56880	54142
[788,]	-1632	787	Yunnan Shilin	Jilin Lishu	56881	54154
[789,]	-1633	788	Yunnan Shizong	Jilin Gujiazi	56883	54155
[790,]	-1634	789	Yunnan Mile	Jilin Changchun	56885	54161
[791,]	-1635	790	Yunnan Luxi	Jilin Yitong	56886	54164
[792,]	-1636	791	Yunnan Qiubei	Jilin Shuangyang	56889	54165
[793,]	-1637	792	Yunnan Luoping	Jilin Yantongshan	56891	54169
[794,]	-1638	793	Yunnan Eshan	Jilin Yongji	56898	54171
[795,]	-1639	794	Yunnan Cangyuan	Jilin Jilin Suburb	56944	54172
[796,]	-1640	795	Yunnan Gengma	Jilin Jiaohe	56946	54181
[797,]	-1641	796	Yunnan Ximeng	Jilin Dunhua	56948	54186
[798,]	-1642	797	Yunnan Menglian	Jilin Antu	56949	54187
[799,]	-1643	798	Yunnan Shuangjiang	Jilin Luozigou	56950	54192
[800,]	-1644	799	Yunnan Lincang	Jilin Wangqing	56951	54195
[801,]	-1645	800	Yunnan Jinggu	Jilin Liaoyuan	56952	54260
[802,]	-1646	801	Yunnan Lancang	Jilin Tongfeng	56954	54261
[803,]	-1647	802	Yunnan Menghai	Jilin Panshi	56958	54263

[804,]	-1648	803	Yunnan Mojiang	Jilin Liuhe	56962	54267
[805,]	-1649	804	Yunnan Simao	Jilin Huadian	56964	54273
[806,]	-1650	805	Yunnan Yuanjiang	Jilin Huinan	56966	54274
[807,]	-1651	806	Yunnan Mengla	Jilin Jiangyuan	56969	54279
[808,]	-1652	807	Yunnan Shiping	Jilin Donggang	56970	54284
[809,]	-1653	808	Yunnan Yuanyang	Jilin Erdao	56976	54285
[810,]	-1655	809	Yunnan Luchun	Jilin Helong	56978	54286
[811,]	-1656	810	Yunnan Kaiyuan	Jilin Longjing	56982	54290
[812,]	-1657	811	Yunnan Gejiu	Jilin Huichun	56984	54291
[813,]	-1659	812	Yunnan Yanshan	Jilin Yanji	56991	54292
[814,]	-1660	813	Yunnan Xichou	Jilin Tonghuaxian	56992	54362
[815,]	-1661	814	Yunnan Wenshan	Jilin Tonghua	56994	54363
[816,]	-1662	815	Yunnan Guangnan	Jilin Baishan	59007	54371
[817,]	-366	-369	Guizhou Pingtang	Guizhou Libo	57921	57926
[818,]	-371	817	Guizhou Congjiang	Jilin Jian	57936	54377
[819,]	-372	-373	Hainan Haikou	Hainan Lingao	59758	59842
[820,]	-375	819	Hainan Danzhou	Jiangsu Fengxian	59845	58012
[821,]	-378	820	Hainan Dingan	Jiangsu Peixian	59851	58013
[822,]	-380	821	Hainan Qonghai	Jiangsu Pizhou	59855	58026
[823,]	-381	822	Hainan Wenchang	Jiangsu Xuzhou	59856	58027
[824,]	-382	823	Hainan Baoting	Jiangsu Donghai	59945	58036
[825,]	-374	-376	Hainan Chengmai	Hainan Changjiang	59843	59847
[826,]	-377	825	Hainan Baisha	Jiangsu Ganyu	59848	58040
[827,]	-379	826	Hainan Tunchang	Jiangsu Xiliandao	59854	58041
[828,]	-385	-571	Hebei Shangyi	Heilongjiang Nenjiang	53397	50557
[829,]	-574	828	Heilongjiang Nehe	Jiangsu Xiangshui	50646	58045
[830,]	-576	829	Heilongjiang Beian	Jiangsu Guanyun	50656	58047
[831,]	-577	830	Heilongjiang Keshan	Jiangsu Suining	50658	58130
[832,]	-586	831	Heilongjiang Yian	Jiangsu Suyu	50750	58131
[833,]	-588	832	Heilongjiang Hailun	Jiangsu Siyang	50756	58132
[834,]	-589	833	Heilongjiang Minshui	Jiangsu Sihong	50758	58135
[835,]	-591	834	Heilongjiang Wuying	Jiangsu Xuyi	50772	58138
[836,]	-595	835	Heilongjiang Tongjiang	Jiangsu Hongze	50778	58139
[837,]	-599	836	Heilongjiang Dumeng	Jiangsu Lianshui	50842	58140
[838,]	-608	837	Heilongjiang Qingan	Jiangsu Huaian	50861	58141
[839,]	-612	838	Heilongjiang Jiamusi	Jiangsu Funing	50873	58143
[840,]	-626	839	Heilongjiang Zhengfang	Jiangsu Jianhu	50964	58146
[841,]	-632	840	Heilongjiang Linkou	Jiangsu Jinhu	50979	58147
[842,]	-637	841	Heilongjiang Hailin	Jiangsu Baoying	54092	58148
[843,]	-639	842	Heilongjiang Mudanjiang	Jiangsu Yancheng	54094	58154
[844,]	-984	843	Inner Mongolia Eerguna	Jiangsu Dafeng	50425	58158
[845,]	-989	844	Inner Mongolia Yakeshi	Jiangsu Liuhe	50526	58235
[846,]	-990	845	Inner Mongolia	Jiangsu Pukou	50548	58237

			Xiaoergou			
[847,]	-994	846	Inner Mongolia Moulidawawoer	Jiangsu Nanjing	50645	58238
[848,]	-995	847	Inner Mongolia Arunqi	Jiangsu Gaoyou	50647	58241
[849,]	-997	848	Inner Mongolia Suolun	Jiangsu Yizheng	50834	58242
[850,]	-999	849	Inner Mongolia Wuzhumuqindong	Jiangsu Xinghua	50915	58243
[851,]	-1013	850	Inner Mongolia Guyangxian	Jiangsu Taizhou	53357	58246
[852,]	-1014	851	Inner Mongolia Siziwang	Jiangsu Yangzhong	53362	58247
[853,]	-1016	852	Inner Mongolia Wuchuanxian	Jiangsu Taixing	53368	58249
[854,]	-1018	853	Inner Mongolia Chayouhouqi	Jiangsu Jiangyan	53384	58250
[855,]	-1019	854	Inner Mongolia Shangdu	Jiangsu Dongtai	53385	58251
[856,]	-1047	855	Inner Mongolia Eduokeqianqi	Jiangsu Haian	53730	58254
[857,]	-1051	856	Inner Mongolia Zhalute	Jiangsu Rugao	54026	58255
[858,]	-1053	857	Inner Mongolia Gaoliban	Jiangsu Jingjiang	54031	58257
[859,]	-1054	858	Inner Mongolia Shebotu	Jiangsu Nantong	54039	58259
[860,]	-1055	859	Inner Mongolia Kezuozhongqi	Jiangsu Rudong	54047	58264
[861,]	-1060	860	Inner Mongolia Alukeerqinqi	Jiangsu Lvsi	54122	58265
[862,]	-1062	861	Inner Mongolia Kailu	Jiangsu Qidong	54134	58269
[863,]	-1066	862	Inner Mongolia Duolunxian	Jiangsu Gaochun	54208	58339
[864,]	-1067	863	Inner Mongolia Wengniuteqi	Jiangsu Lishui	54213	58340
[865,]	-1070	864	Inner Mongolia Neiman	Jiangsu Danyang	54223	58341
[866,]	-388	-413	Hebei Yangyuan	Hebei Quzhou	53492	53893
[867,]	-473	866	Hebei Qinghe	Jiangsu Jurong	54706	58344
[868,]	-394	-396	Hebei Lingshou	Hebei Xingtang	53680	53688
[869,]	-397	868	Hebei Jinzhou	Jiangsu Suzhou	53689	58349
[870,]	-399	869	Hebei Tangxian	Jiangsu Changshu	53692	58352
[871,]	-401	870	Hebei Wuji	Jiangsu Zhangjiagang	53699	58353
[872,]	-403	871	Hebei Shahe	Jiangsu Wuxi	53781	58354
[873,]	-404	872	Hebei Baixiang	Jiangsu Kunshan	53785	58356
[874,]	-406	873	Hebei Longyao	Jiangsu Haimen	53794	58360
[875,]	-408	874	Hebei Ningjin	Jiangsu Taicang	53796	58377
[876,]	-409	875	Hebei Julu	Jiangxi Xiushui	53799	57598
[877,]	-411	876	Hebei Wuan	Jiangxi Tonggu	53890	57694
[878,]	-427	877	Hebei Chengde	Jiangxi Wanzai	54423	57698

[879,]	-433	878	Hebei Qinglong	Jiangxi Shanggao	54436	57699
[880,]	-438	879	Hebei Zhuozhou	Jiangxi Pingxiang	54502	57786
[881,]	-439	880	Hebei Rongcheng	Jiangxi Lianhua	54503	57789
[882,]	-443	881	Hebei Yongqing	Jiangxi Fenyi	54519	57792
[883,]	-449	882	Hebei Leting	Jiangxi Yichun	54539	57793
[884,]	-451	883	Hebei Funing	Jiangxi Xinyu	54541	57796
[885,]	-455	884	Hebei Raoyang	Jiangxi Anfu	54606	57798
[886,]	-465	885	Hebei Haixing	Jiangxi Jianxian	54628	57799
[887,]	-468	886	Hebei Jize	Jiangxi Xiaping	54640	57883
[888,]	-478	887	Hebei-Weixian	Jiangxi Yongxin	54800	57891
[889,]	-395	-447	Hebei Quyang	Hebei Tangshan	53682	54534
[890,]	-458	889	Hebei Wenan	Jiangxi Suichuan	54612	57896
[891,]	-402	-483	Hebei Linzhang	Henan Qinyang	53773	53972
[892,]	-486	891	Henan Jiaozuo	Jiangxi Chongyi	53982	57990
[893,]	-487	892	Henan Fengqiu	Jiangxi Nankang	53983	57992
[894,]	-488	893	Henan Xiuwu	Jiangxi Ganxian	53984	57993
[895,]	-489	894	Henan Huixian	Jiangxi Xinfeng	53985	57995
[896,]	-491	895	Henan Tangyin	Jiangxi Jiujiang	53991	58502
[897,]	-493	896	Henan Neihuang	Jiangxi Ruichang	53993	58503
[898,]	-505	897	Henan Mengzhou	Jiangxi Lushan	57072	58506
[899,]	-510	898	Henan Gongyi	Jiangxi Wuning	57080	58507
[900,]	-512	899	Henan Dengfeng	Jiangxi Dean	57082	58508
[901,]	-513	900	Henan Changge	Jiangxi Hukou	57087	58510
[902,]	-523	901	Henan Lushan	Jiangxi Pengze	57173	58512
[903,]	-529	902	Henan Jiaxian	Jiangxi Duchang	57180	58517
[904,]	-530	903	Henan Xiangcheng	Jiangxi Poyang	57182	58519
[905,]	-531	904	Henan Linying	Jiangxi Jingdezhen	57183	58527
[906,]	-535	905	Henan Sheqi	Jiangxi Wuyuan	57187	58529
[907,]	-550	906	Henan Xinyang	Jiangxi Jingan	57297	58600
[908,]	-1201	907	Shandong Caoxian	Jiangxi Fengxin	58002	58601
[909,]	-410	-1212	Hebei Shexian	Shanxi Pianguan	53886	53565
[910,]	-1215	909	Shanxi Shanyin	Jiangxi Gaoan	53576	58605
[911,]	-1241	910	Shanxi Gujiao	Jiangxi Nanchang	53763	58606
[912,]	-1242	911	Shanxi Lishi	Jiangxi Yugan	53764	58612
[913,]	-1257	912	Shanxi Xiangfen	Jiangxi Jinxian	53861	58614
[914,]	-1259	913	Shanxi Jiexiu	Jiangxi Wannian	53863	58615
[915,]	-1261	914	Shanxi Fenxi	Jiangxi Dongxiang	53865	58618
[916,]	-1269	915	Shanxi Licheng	Jiangxi Linchuan	53878	58619
[917,]	-1277	916	Shanxi Jiangxian	Jiangxi Dexing	53965	58622
[918,]	-1281	917	Shanxi Gaoping	Jiangxi Shangraoxian	53973	58623
[919,]	-417	-448	Hebei Chongli	Hebei Caofeidian	54304	54535
[920,]	-431	-435	Hebei Kuancheng	Hebei Lulong	54432	54438
[921,]	-445	920	Hebei Xianghe	Jiangxi Yushan	54521	58634

[922,]	-453	921	Hebei Goyang	Jiangxi Xinjian	54603	58693
[923,]	-456	922	Hebei Shenzhou	Jiangxi Xiajiang	54608	58704
[924,]	-457	923	Hebei Renqiu	Jiangxi Yongfeng	54610	58705
[925,]	-442	-466	Hebei Guan	Hebei Guangzong	54512	54631
[926,]	-472	-474	Hebei Wuyi	Hebei Gucheng	54703	54707
[927,]	-481	926	Hebei Guantao	Jiangxi Jinxi	54809	58712
[928,]	-495	927	Henan Taiqian	Jiangxi Nancheng	54817	58715
[929,]	-1136	928	Shandong Leling	Jiangxi Nanfeng	54726	58718
[930,]	-1173	929	Shandong Zhucheng	Jiangxi Lichuan	54848	58719
[931,]	-476	-496	Hebei Jingxian	Henan Puyang	54711	54900
[932,]	-1132	931	Shandong Linyi	Jiangxi Ningdu	54712	58806
[933,]	-1139	932	Shandong Binzhou	Jiangxi Guangchang	54734	58813
[934,]	-1141	933	Shandong Laizhou	Jiangxi Shicheng	54749	58814
[935,]	-1142	934	Shandong Longdao	Jiangxi Yudu	54751	58905
[936,]	-1143	935	Shandong Penglai	Jiangxi Huichang	54752	58906
[937,]	-1147	936	Shandong Fushan	Jiangxi Anyuan	54764	58907
[938,]	-1148	937	Shandong Yantai	Jiangxi Quannan	54765	59091
[939,]	-1149	938	Shandong Chengshantou	Jiangxi Longnan	54776	59092
[940,]	-1150	939	Shandong Wendeng	Jiangxi Dingnan	54777	59093
[941,]	-1151	940	Shandong Linqing	Jiangxi Xunwu	54802	59102
[942,]	-1154	941	Shandong Qihe	Jiangxi Shangli	54812	57783
[943,]	-1158	942	Shandong Jiyang	Liaoning Zhangwu	54821	54236
[944,]	-1159	943	Shandong Zouping	Liaoning Changtu	54822	54243
[945,]	-1161	944	Shandong Taian	Liaoning Kangping	54827	54244
[946,]	-1163	945	Shandong Zibo	Liaoning Shenbei	54830	54248
[947,]	-1165	946	Shandong Shouguang	Liaoning Tieling	54832	54249
[948,]	-1166	947	Shandong Huantai	Liaoning Xifeng	54833	54252
[949,]	-1167	948	Shandong Yiyuan	Liaoning Qingyuan	54836	54259
[950,]	-1168	949	Shandong Changyi	Liaoning Jianpingzhen	54841	54321
[951,]	-1169	950	Shandong Pingdu	Liaoning Chaoyang	54842	54324
[952,]	-1170	951	Shandong Weifang	Liaoning Yangshan	54843	54325
[953,]	-1172	952	Shandong Gaomi	Liaoning Jianpingxian	54846	54326
[954,]	-1174	953	Shandong Jiaozhou	Liaoning Lingyuan	54849	54327
[955,]	-1176	954	Shandong Jimo	Liaoning Liaozhong	54855	54332
[956,]	-1179	955	Shandong Yuncheng	Liaoning Xinmin	54905	54333
[957,]	-1185	956	Shandong Wenshang	Liaoning Taian	54912	54336
[958,]	-1191	957	Shandong Mengyin	Liaoning Panshan	54923	54338
[959,]	-1196	958	Shandong Linyi	Liaoning Anshan	54938	54339
[960,]	-1197	959	Shandong Junan	Liaoning Sujiatun	54939	54340
[961,]	-1207	960	Shandong Linshu	Liaoning Shenyang	58032	54342
[962,]	-477	-1155	Hebei Dongguang	Shandong Chiping	54713	54814
[963,]	-479	-1131	Hebei Linxi	Shandong Wucheng	54801	54709
[964,]	-1162	963	Shandong Laiwu	Liaoning Fushun	54828	54351

[965,]	-1190	964	Shandong Xintai	Liaoning Xinbin	54922	54353
[966,]	-1195	965	Shandong Yishui	Liaoning Jianchang	54932	54452
[967,]	-494	-500	Henan Changheng	Henan Lingbao	53998	57056
[968,]	-502	967	Henan Luoning	Liaoning Suizhong	57066	54454
[969,]	-508	968	Henan Ruyang	Liaoning Xingcheng	57078	54455
[970,]	-528	969	Henan Fangcheng	Liaoning Yingkou	57179	54471
[971,]	-534	970	Henan Luohe	Liaoning Gaizhou	57186	54474
[972,]	-547	971	Henan Xincai	Liaoning Dashiqiao	57293	54475
[973,]	-549	972	Henan Xixian	Liaoning Caohekou	57296	54483
[974,]	-554	973	Henan Minquan	Liaoning Xiuyan	58004	54486
[975,]	-560	974	Henan Huangchuan	Liaoning Kuandian	58207	54493
[976,]	-525	-539	Henan Nanzhao	Henan Xihua	57176	57193
[977,]	-546	976	Henan Pingyu	Liaoning Dandong	57292	54497
[978,]	-831	977	Jiangsu Suyu	Liaoning Wafangdian	58131	54563
[979,]	-532	-555	Henan Yexian	Henan Shangqiu	57184	58005
[980,]	-1153	979	Shandong Xinxian	Liaoning Pulandian	54808	54569
[981,]	-538	-1282	Henan Huaiyang	Shanxi Lingchuan	57192	53981
[982,]	-541	-658	Henan Xinye	Hubei Jingmen	57271	57377
[983,]	-696	982	Hubei Luotian	Liaoning Zhuanghe	58401	54584
[984,]	-543	-544	Henan Biyang	Henan Tongbai	57281	57285
[985,]	-552	984	Henan Jigongshan	Inner Mongolia Eerguna	57390	50425
[986,]	-553	985	Henan Xixian	Inner Mongolia Tulihe	57396	50434
[987,]	-562	986	Henan Shangcheng	Inner Mongolia Elunchunqi	58301	50445
[988,]	-643	987	Hubei Yunxi	Inner Mongolia Manzhouli	57251	50514
[989,]	-644	988	Hubei Yunxian	Inner Mongolia Evenkeqi	57253	50525
[990,]	-645	989	Hubei Shiyan	Inner Mongolia Yakeshi	57256	50526
[991,]	-646	990	Hubei Zhushan	Inner Mongolia Xiaoergou	57257	50548
[992,]	-647	991	Hubei Fangxian	Inner Mongolia Xinbaerhuyouqi	57259	50603
[993,]	-648	992	Hubei Danjiangkou	Inner Mongolia Xinbaerhuzuoqi	57260	50618
[994,]	-649	993	Hubei Gucheng	Inner Mongolia Zhalantun	57268	50639
[995,]	-653	994	Hubei Xingshan	Inner Mongolia Moulidawawoer	57359	50645
[996,]	-654	995	Hubei Baokang	Inner Mongolia Arunqi	57361	50647
[997,]	-655	996	Hubei Shennongjia	Inner Mongolia Aershan	57362	50727
[998,]	-656	997	Hubei Nanzhang	Inner Mongolia Suolun	57363	50834
[999,]	-659	998	Hubei Zhongxiang	Inner Mongolia Wulagai	57378	50913
[1000,]	-660	999	Hubei Suizhou	Inner Mongolia	57381	50915

				Wuzhumuqindong		
[1001,]	-662	1000	Hubei Jingshan	Inner Mongolia Houlinguole	57387	50924
[1002,]	-665	1001	Hubei Macheng	Inner Mongolia Bayaertuhushuo	57399	50928
[1003,]	-666	1002	Hubei Lichuan	Inner Mongolia Tuquan	57439	50934
[1004,]	-668	1003	Hubei Enshi	Inner Mongolia Erlianhaote	57447	53068
[1005,]	-669	1004	Hubei Yiling	Inner Mongolia Narenbaolige	57453	53083
[1006,]	-670	1005	Hubei Wufeng	Inner Mongolia Mandula	57458	53149
[1007,]	-673	1006	Hubei Gongan	Inner Mongolia Abagaqi	57477	53192
[1008,]	-678	1007	Hubei Xiantao	Inner Mongolia Hailisu	57485	53231
[1009,]	-685	1008	Hubei Xianfeng	Inner Mongolia Xianghuangqi	57540	53289
[1010,]	-686	1009	Hubei Xuanen	Inner Mongolia Wulatezhongqi	57541	53336
[1011,]	-693	1010	Hubei Chongyang	Inner Mongolia Wuyuan	57586	53337
[1012,]	-695	1011	Hubei Xianning	Inner Mongolia Dashetai	57590	53348
[1013,]	-697	1012	Hubei Yingshan	Inner Mongolia Damaoqi	58402	53352
[1014,]	-698	1013	Hubei Qichun	Inner Mongolia Guyangxian	58408	53357
[1015,]	-699	1014	Hubei Huangmei	Inner Mongolia Siziwang	58409	53362
[1016,]	-1350	1015	Shaanxi Baihe	Inner Mongolia Xilamuren	57254	53367
[1017,]	-545	-557	Henan Zhumadian	Henan Echeng	57290	58007
[1018,]	-563	-565	Heilongjiang Mohe	Heilongjiang Tahe	50136	50246
[1019,]	-566	1018	Heilongjiang Huzhong	Inner Mongolia Chayouhouqi	50247	53384
[1020,]	-568	1019	Heilongjiang Huma	Inner Mongolia Shangdu	50353	53385
[1021,]	-572	1020	Heilongjiang Sunwu	Inner Mongolia Huade	50564	53391
[1022,]	-573	1021	Heilongjiang Xunke	Inner Mongolia Dengkou	50566	53419
[1023,]	-580	1022	Heilongjiang Wuyiling	Inner Mongolia Hangjinhouqi	50674	53420
[1024,]	-582	1023	Heilongjiang Gannan	Inner Mongolia Wulateqianqi	50741	53433
[1025,]	-587	1024	Heilongjiang Baiquan	Inner Mongolia Baotou	50755	53446
[1026,]	-592	1025	Heilongjiang Yichun	Inner Mongolia Tuyouqi	50774	53455
[1027,]	-597	1026	Heilongjiang Suibin	Inner Mongolia Dalateqi	50787	53457
[1028,]	-602	1027	Heilongjiang Qinggang	Inner Mongolia Huhehaote	50851	53463
[1029,]	-616	1028	Heilongjiang Shuangyashan	Inner Mongolia Tumutezuoqi	50884	53464

[1030,]	-617	1029	Heilongjiang Baoqing	Inner Mongolia Huhehaote suburb	50888	53466
[1031,]	-624	1030	Heilongjiang Mulan	Inner Mongolia Tuoketuoxian	50962	53467
[1032,]	-625	1031	Heilongjiang Tonghe	Inner Mongolia Helingeerxian	50963	53469
[1033,]	-628	1032	Heilongjiang Shangzhi	Inner Mongolia Zhuozi	50968	53472
[1034,]	-564	-579	Heilongjiang Beijicun	Heilongjiang Jiayin	50137	50673
[1035,]	-618	1034	Heilongjiang Raohe	Inner Mongolia Jining	50892	53480
[1036,]	-633	1035	Heilongjiang Hulin	Inner Mongolia Chayouqianqi	50983	53481
[1037,]	-810	1036	Jilin Longjing	Inner Mongolia Xinghe	54290	53483
[1038,]	-811	1037	Jilin Huichun	Inner Mongolia Wuhai	54291	53512
[1039,]	-816	1038	Jilin Linjiang	Inner Mongolia Linhe	54374	53513
[1040,]	-1507	1039	Xinjiang Yumin	Inner Mongolia Yikewusu	51137	53522
[1041,]	-1510	1040	Xinjiang Alashankou	Inner Mongolia Etukeqi	51232	53529
[1042,]	-567	-569	Heilongjiang Xinlin	Heilongjiang Jiagedaqi	50349	50442
[1043,]	-986	1042	Inner Mongolia Elunchunqi	Inner Mongolia Dongsheng	50445	53543
[1044,]	-987	1043	Inner Mongolia Manzhouli	Inner Mongolia Ejinhuoluoqi	50514	53545
[1045,]	-992	1044	Inner Mongolia Xinbaerhuzuoqi	Inner Mongolia Zhungeerqi	50618	53553
[1046,]	-1001	1045	Inner Mongolia Bayaertuhushuo	Inner Mongolia Qingshuihexian	50928	53562
[1047,]	-1003	1046	Inner Mongolia Erlianhaote	Inner Mongolia Wushenqi	53068	53644
[1048,]	-1005	1047	Inner Mongolia Mandula	Inner Mongolia Eduokeqianqi	53149	53730
[1049,]	-1006	1048	Inner Mongolia Abagaqi	Inner Mongolia Henan	53192	53732
[1050,]	-1008	1049	Inner Mongolia Xianghuangqi	Inner Mongolia Xiwuzhumuqin	53289	54012
[1051,]	-1009	1050	Inner Mongolia Wulatezhongqi	Inner Mongolia Fuhe	53336	54024
[1052,]	-1011	1051	Inner Mongolia Dashetai	Inner Mongolia Zhalute	53348	54026
[1053,]	-1015	1052	Inner Mongolia Xilamuren	Inner Mongolia Balinzuoqi	53367	54027
[1054,]	-1017	1053	Inner Mongolia Chayouzhongqi	Inner Mongolia Gaoliban	53378	54031
[1055,]	-1020	1054	Inner Mongolia Huade	Inner Mongolia Shebotu	53391	54039
[1056,]	-1021	1055	Inner Mongolia Dengkou	Inner Mongolia Kezuozhongqi	53419	54047
[1057,]	-1022	1056	Inner Mongolia	Inner Mongolia	53420	54102

			Hangjinhouqi	Xilinhaote		
[1058,]	-1024	1057	Inner Mongolia Baotou	Inner Mongolia Balinyouqi	53446	54113
[1059,]	-1025	1058	Inner Mongolia Tuyouqi	Inner Mongolia Linxixian	53455	54115
[1060,]	-1026	1059	Inner Mongolia Dalateqi	Inner Mongolia Keshiketengqi	53457	54117
[1061,]	-1029	1060	Inner Mongolia Huhehaote suburb	Inner Mongolia Alukeerqinqi	53466	54122
[1062,]	-1030	1061	Inner Mongolia Tuoketuoxian	Inner Mongolia Qinglongshan	53467	54132
[1063,]	-1032	1062	Inner Mongolia Zhuozi	Inner Mongolia Kailu	53472	54134
[1064,]	-1033	1063	Inner Mongolia Liangcheng	Inner Mongolia Tongliao	53475	54135
[1065,]	-1036	1064	Inner Mongolia Xinghe	Inner Mongolia Zhengxiangbaiqi	53483	54204
[1066,]	-1037	1065	Inner Mongolia Wuhai	Inner Mongolia Zhenglanqi	53512	54205
[1067,]	-1039	1066	Inner Mongolia Yikewusu	Inner Mongolia Duolunxian	53522	54208
[1068,]	-1040	1067	Inner Mongolia Etukeqi	Inner Mongolia Wengniuteqi	53529	54213
[1069,]	-1041	1068	Inner Mongolia Hangjinqi	Inner Mongolia Gangzi	53533	54214
[1070,]	-1042	1069	Inner Mongolia Dongsheng	Inner Mongolia Chifeng	53543	54218
[1071,]	-1043	1070	Inner Mongolia Ejinhuoluoqi	Inner Mongolia Neiman	53545	54223
[1072,]	-1044	1071	Inner Mongolia Zhungeerqi	Inner Mongolia Aohanqi	53553	54225
[1073,]	-1045	1072	Inner Mongolia Qingshuihexian	Inner Mongolia Baoguotu	53562	54226
[1074,]	-1046	1073	Inner Mongolia Wushenqi	Inner Mongolia Kezuohouqi	53644	54231
[1075,]	-1049	1074	Inner Mongolia Xiwuzhumuqin	Inner Mongolia Kulun	54012	54234
[1076,]	-1050	1075	Inner Mongolia Fuhe	Inner Mongolia Taibushiqi	54024	54305
[1077,]	-1052	1076	Inner Mongolia Balinzuoqi	Inner Mongolia Kalaqinqi	54027	54313
[1078,]	-1056	1077	Inner Mongolia Xilinhaote	Inner Mongolia Balihan	54102	54316
[1079,]	-1057	1078	Inner Mongolia Balinyouqi	Inner Mongolia Ningchengxian	54113	54320
[1080,]	-1058	1079	Inner Mongolia Linxixian	Ningxia Shitanjing	54115	53517

[1081,]	-1059	1080	Inner Mongolia Keshiketengqi	Ningxia Huinong	54117	53519
[1082,]	-1063	1081	Inner Mongolia Tongliao	Ningxia Helan	54135	53610
[1083,]	-1065	1082	Inner Mongolia Zhenglanqi	Ningxia Pingluo	54205	53611
[1084,]	-1068	1083	Inner Mongolia Gangzi	Ningxia Wuzhong	54214	53612
[1085,]	-1069	1084	Inner Mongolia Chifeng	Ningxia Taole	54218	53615
[1086,]	-1072	1085	Inner Mongolia Baoguotu	Ningxia Yongning	54226	53618
[1087,]	-1076	1086	Inner Mongolia Kalaqinqi	Ningxia Zhongwei	54313	53704
[1088,]	-578	-606	Heilongjiang Kedong	Heilongjiang Zhaodong	50659	50858
[1089,]	-621	1088	Heilongjiang Hulan	Ningxia Xingren	50956	53707
[1090,]	-630	1089	Heilongjiang Boli	Ningxia Yanchi	50973	53723
[1091,]	-631	1090	Heilongjiang Jixi	Ningxia Maihuangshan	50978	53727
[1092,]	-638	1091	Heilongjiang Muling	Ningxia Haiyuan	54093	53806
[1093,]	-581	-641	Heilongjiang Longjiang	Heilongjiang Ningan	50739	54098
[1094,]	-584	-594	Heilongjiang Qiqihaer	Heilongjiang Luobei	50745	50776
[1095,]	-607	1094	Heilongjiang Lansi	Ningxia Weizhou	50859	53881
[1096,]	-613	1095	Heilongjiang Yilang	Ningxia Xiji	50877	53903
[1097,]	-614	1096	Heilongjiang Huachuan	Ningxia Liupanshan	50878	53910
[1098,]	-622	1097	Heilongjiang Acheng	Qinghai Lenghu	50958	52602
[1099,]	-623	1098	Heilongjiang Binxian	Qinghai Nuomuhong	50960	52825
[1100,]	-585	-600	Heilongjiang Lindian	Heilongjiang Tailai	50749	50844
[1101,]	-771	1100	Jilin Baicheng	Qinghai Qinghaihu 151	50936	52854
[1102,]	-772	1101	Jilin Taonan	Qinghai Gonghe	50939	52856
[1103,]	-774	1102	Jilin Daan	Qinghai Huzhu	50945	52863
[1104,]	-778	1103	Jilin Tongyu	Qinghai Xining	54041	52866
[1105,]	-780	1104	Jilin Fuyu	Qinghai Guide	54063	52868
[1106,]	-795	1105	Jilin Jiaohe	Qinghai Pingan	54181	52875
[1107,]	-796	1106	Jilin Dunhua	Qinghai Minhe	54186	52876
[1108,]	-797	1107	Jilin Antu	Qinghai Hualong	54187	52877
[1109,]	-798	1108	Jilin Luozigou	Qinghai Wudaoliang	54192	52908
[1110,]	-799	1109	Jilin Wangqing	Qinghai Shazhuyu	54195	52941
[1111,]	-806	1110	Jilin Jiangyuan	Qinghai Xinghai	54279	52943
[1112,]	-808	1111	Jilin Erdao	Qinghai Guinan	54285	52955
[1113,]	-809	1112	Jilin Helong	Qinghai Tongde	54286	52957
[1114,]	-812	1113	Jilin Yanji	Qinghai Jianzha	54292	52963
[1115,]	-815	1114	Jilin Baishan	Qinghai Zeku	54371	52968
[1116,]	-598	-615	Heilongjiang Fujin	Heilongjiang Huanan	50788	50879
[1117,]	-601	-1480	Heilongjiang Daqing	Tibet Naqu	50850	55299
[1118,]	-1482	1117	Tibet Dangxiong	Qinghai Tuotuohe	55493	56004
[1119,]	-1483	1118	Tibet Lazi	Qinghai Zhiduo	55569	56016
[1120,]	-1484	1119	Tibet Nanmulin	Qinghai Zaduo	55572	56018

[1121,]	-1485	1120	Tibet Muozhugongka	Qinghai Qumacai	55593	56021
[1122,]	-1489	1121	Tibet Jiangzi	Qinghai Yushu	55680	56029
[1123,]	-1491	1122	Tibet Leiwuqi	Qinghai Maduo	56128	56033
[1124,]	-1497	1123	Tibet Miling	Qinghai Qingshuihe	56317	56034
[1125,]	-603	-611	Heilongjiang Wangkui	Heilongjiang Tangyuan	50852	50871
[1126,]	-604	-609	Heilongjiang Beilin	Heilongjiang Tieli	50853	50862
[1127,]	-629	-635	Heilongjiang Qitaihe	Heilongjiang Jidong	50971	50987
[1128,]	-640	1127	Heilongjiang Suifenhe	Qinghai Henan	54096	56065
[1129,]	-634	-1500	Heilongjiang Mishan	Xinjiang Habahe	50985	51053
[1130,]	-1506	1129	Xinjiang Tacheng	Qinghai Nangqian	51133	56125
[1131,]	-650	-651	Hubei Xiangyang	Hubei Zaoyang	57278	57279
[1132,]	-664	1131	Hubei Hongan	Shandong Wucheng	57398	54709
[1133,]	-671	1132	Hubei Songzi	Shandong Linyi	57469	54712
[1134,]	-672	1133	Hubei Qianjiang	Shandong Ningjin	57475	54716
[1135,]	-677	1134	Hubei Shayang	Shandong Yangxin	57484	54723
[1136,]	-679	1135	Hubei Hanchuan	Shandong Shanghe	57486	54724
[1137,]	-680	1136	Hubei Caidian	Shandong Leling	57489	54726
[1138,]	-681	1137	Hubei Xinzhou	Shandong Zhangqiu	57492	54727
[1139,]	-682	1138	Hubei Wuhan	Shandong Gaoqing	57494	54729
[1140,]	-684	1139	Hubei Ezhou	Shandong Binzhou	57496	54734
[1141,]	-661	-674	Hubei Xiaochang	Hubei Yingcheng	57386	57481
[1142,]	-675	1141	Hubei Xiaogan	Shandong Laizhou	57482	54749
[1143,]	-676	1142	Hubei Tianmen	Shandong Longdao	57483	54751
[1144,]	-700	1143	Hubei Yangxin	Shandong Penglai	58500	54752
[1145,]	-687	-702	Hubei Hefeng	Hunan Longshan	57543	57544
[1146,]	-703	1145	Hunan Sangzhi	Shandong Zhaoyuan	57554	54755
[1147,]	-715	1146	Hunan Taoyuan	Shandong Qixia	57661	54759
[1148,]	-736	1147	Hunan Xiangxiang	Shandong Fushan	57772	54764
[1149,]	-751	1148	Hunan Changning	Shandong Yantai	57874	54765
[1150,]	-880	1149	Jiangxi Lianhua	Shandong Chengshantou	57789	54776
[1151,]	-688	-689	Hubei Shishou	Hubei Jianli	57571	57573
[1152,]	-690	1151	Hubei Honghu	Shandong Linqing	57581	54802
[1153,]	-691	1152	Hubei Chibi	Shandong Liaocheng	57582	54806
[1154,]	-694	1153	Hubei Tongcheng	Shandong Xinxian	57589	54808
[1155,]	-704	1154	Hunan Zhangjiajie	Shandong Qihe	57558	54812
[1156,]	-705	1155	Hunan Lixian	Shandong Chiping	57565	54814
[1157,]	-706	1156	Hunan Nanxian	Shandong Donge	57574	54815
[1158,]	-707	1157	Hunan Huarong	Shandong Feicheng	57575	54819
[1159,]	-708	1158	Hunan Yueyang	Shandong Jiyang	57584	54821
[1160,]	-711	1159	Hunan Guzhang	Shandong Zouping	57646	54822
[1161,]	-712	1160	Hunan Jishou	Shandong Jinan	57649	54823
[1162,]	-714	1161	Hunan Luxi	Shandong Taian	57657	54827
[1163,]	-716	1162	Hunan Changde	Shandong Laiwu	57662	54828

[1164,]	-717	1163	Hunan Hanshou	Shandong Zibo	57663	54830
[1165,]	-718	1164	Hunan Taojiang	Shandong Qingzhou	57666	54831
[1166,]	-720	1165	Hunan Yuanjiang	Shandong Shouguang	57671	54832
[1167,]	-722	1166	Hunan Ningxiang	Shandong Huantai	57678	54833
[1168,]	-723	1167	Hunan Milo	Shandong Yiyuan	57680	54836
[1169,]	-724	1168	Hunan Pingjiang	Shandong Changyi	57682	54841
[1170,]	-725	1169	Hunan Changsha	Shandong Pingdu	57687	54842
[1171,]	-726	1170	Hunan Liuyang	Shandong Weifang	57688	54843
[1172,]	-730	1171	Hunan Xupu	Shandong Anqiu	57752	54844
[1173,]	-731	1172	Hunan Hongjiang	Shandong Gaomi	57754	54846
[1174,]	-732	1173	Hunan Lengshuijiang	Shandong Zhucheng	57760	54848
[1175,]	-733	1174	Hunan Xinhua	Shandong Jiaozhou	57761	54849
[1176,]	-734	1175	Hunan Loudi	Shandong Laiyang	57763	54852
[1177,]	-735	1176	Hunan Shaoshan	Shandong Jimo	57771	54855
[1178,]	-737	1177	Hunan Shuangfeng	Shandong Rushan	57774	54861
[1179,]	-738	1178	Hunan Nanyue	Shandong Juancheng	57776	54904
[1180,]	-739	1179	Hunan Hengshan	Shandong Yuncheng	57777	54905
[1181,]	-740	1180	Hunan Youxian	Shandong Heze	57779	54906
[1182,]	-741	1181	Hunan Zhuzhou	Shandong Yutai	57780	54907
[1183,]	-742	1182	Hunan Liling	Shandong Dingtao	57781	54909
[1184,]	-744	1183	Hunan Lengshuitan	Shandong Liangshan	57865	54910
[1185,]	-745	1184	Hunan Yongzhou	Shandong Dongping	57866	54911
[1186,]	-747	1185	Hunan Qiyang	Shandong Wenshang	57868	54912
[1187,]	-748	1186	Hunan Qidong	Shandong Juye	57870	54914
[1188,]	-749	1187	Hunan Hengyangxian	Shandong Yanzhou	57871	54916
[1189,]	-750	1188	Hunan Hengyang	Shandong Zoucheng	57872	54919
[1190,]	-752	1189	Hunan Hengnan	Shandong Sishui	57875	54920
[1191,]	-753	1190	Hunan Anren	Shandong Xintai	57881	54922
[1192,]	-754	1191	Hunan Chaling	Shandong Mengyin	57882	54923
[1193,]	-755	1192	Hunan Yongxing	Shandong Pingyi	57887	54925
[1194,]	-756	1193	Hunan Guangxi	Shandong Tengzhou	57889	54927
[1195,]	-759	1194	Hunan Ningyuan	Shandong Feixian	57966	54929
[1196,]	-761	1195	Hunan Xintian	Shandong Yishui	57971	54932
[1197,]	-763	1196	Hunan Guiyang	Shandong Linyi	57973	54938
[1198,]	-764	1197	Hunan Jiahe	Shandong Junan	57974	54939
[1199,]	-768	1198	Hunan Zixing	Shandong Wulian	57981	54940
[1200,]	-876	1199	Jiangxi Tonggu	Shandong Huangdao	57694	54943
[1201,]	-879	1200	Jiangxi Pingxiang	Shandong Rizhao	57786	54945
[1202,]	-941	1201	Jiangxi Shangli	Shandong Caoxian	57783	58002
[1203,]	-709	-1464	Hunan Baojing	Sichuan Linshui	57642	57416
[1204,]	-1720	1203	Chongqing Yunyang	Shandong Xuechen	57339	58021
[1205,]	-1725	1204	Chongqing Wanzhou	Shandong Yicheng	57432	58022
[1206,]	-1731	1205	Chongqing Wansheng	Shandong Zaozhuang	57509	58024

[1207,]	-1733	1206	Chongqing Beibei	Shandong Cangshan	57511	58030
[1208,]	-1734	1207	Chongqing Yubei	Shandong Linshu	57513	58032
[1209,]	-1738	1208	Chongqing Nanchuan	Shanxi Youyu	57519	53478
[1210,]	-1740	1209	Chongqing Fengdu	Shanxi Yanggao	57523	53486
[1211,]	-710	-770	Hunan Yongshun	Hunan Jianghua	57643	59063
[1212,]	-719	-758	Hunan Anhua	Hunan Daoxian	57669	57965
[1213,]	-760	1212	Hunan Jiangyong	Shanxi Pianguan	57969	53565
[1214,]	-762	-766	Hunan Chenzhou	Hunan Yizhang	57972	57976
[1215,]	-767	1214	Hunan Linwu	Shanxi Shenchi	57978	53575
[1216,]	-773	-804	Jilin Zhenlai	Jilin Huadian	50940	54273
[1217,]	-775	-776	Jilin Songyuan	Jilin Qianan	50946	50948
[1218,]	-781	1217	Jilin Nongan	Shanxi Shuozhou	54064	53578
[1219,]	-782	1218	Jilin Dehui	Shanxi Daixian	54065	53579
[1220,]	-791	1219	Jilin Shuangyang	Shanxi Hunyuan	54165	53582
[1221,]	-817	1220	Jilin Jian	Shanxi Yingxian	54377	53584
[1222,]	-777	-783	Jilin Qianguo	Jilin Jiutai	50949	54069
[1223,]	-785	1222	Jilin Shulan	Shanxi Wutaishan	54076	53588
[1224,]	-792	1223	Jilin Yantongshan	Shanxi Guangling	54169	53590
[1225,]	-800	1224	Jilin Liaoyuan	Shanxi Linxian	54260	53659
[1226,]	-801	1225	Jilin Tongfeng	Shanxi Kelan	54261	53662
[1227,]	-802	1226	Jilin Panshi	Shanxi Wuzhai	54263	53663
[1228,]	-805	1227	Jilin Huinan	Shanxi Xingxian	54274	53664
[1229,]	-779	-784	Jilin Changling	Jilin Yushu	54049	54072
[1230,]	-790	1229	Jilin Yitong	Shanxi Jingle	54164	53666
[1231,]	-793	1230	Jilin Yongji	Shanxi Yuanping	54171	53673
[1232,]	-794	1231	Jilin Jilin Suburb	Shanxi Xinfu	54172	53674
[1233,]	-814	1232	Jilin Tonghua	Shanxi Dingxiang	54363	53676
[1234,]	-787	-803	Jilin Lishu	Jilin Liuhe	54154	54267
[1235,]	-943	1234	Liaoning Changtu	Shanxi Xiaodian	54243	53679
[1236,]	-944	1235	Liaoning Kangping	Shanxi Wutaixian	54244	53681
[1237,]	-946	1236	Liaoning Tieling	Shanxi Yuxian	54249	53685
[1238,]	-952	1237	Liaoning Jianpingxian	Shanxi Pingding	54326	53687
[1239,]	-954	1238	Liaoning Liaozhong	Shanxi Liulin	54332	53753
[1240,]	-955	1239	Liaoning Xinmin	Shanxi Shilou	54333	53759
[1241,]	-957	1240	Liaoning Panshan	Shanxi Fangshan	54338	53760
[1242,]	-962	1241	Liaoning Benxi	Shanxi Gujiao	54346	53763
[1243,]	-964	1242	Liaoning Xinbin	Shanxi Lishi	54353	53764
[1244,]	-971	1243	Liaoning Dashiqiao	Shanxi Zhongyang	54475	53767
[1245,]	-972	1244	Liaoning Caohekou	Shanxi Xiaoyi	54483	53768
[1246,]	-973	1245	Liaoning Xiuyan	Shanxi Qingxu	54486	53774
[1247,]	-977	1246	Liaoning Wafangdian	Shanxi Taigu	54563	53775
[1248,]	-978	1247	Liaoning Jinzhou	Shanxi Pingyao	54568	53778
[1249,]	-980	1248	Liaoning Pikou	Shanxi Shouyang	54575	53780

[1250,]	-813	-956	Jilin Tonghuaxian	Liaoning Taian	54362	54336
[1251,]	-958	1250	Liaoning Anshan	Shanxi Zuoquan	54339	53786
[1252,]	-960	1251	Liaoning Shenyang	Shanxi Yushe	54342	53787
[1253,]	-969	1252	Liaoning Yingkou	Shanxi Heshun	54471	53788
[1254,]	-970	1253	Liaoning Gaizhou	Shanxi Yonghe	54474	53852
[1255,]	-975	1254	Liaoning Fengcheng	Shanxi Xixian	54494	53853
[1256,]	-979	1255	Liaoning Pulandian	Shanxi Jixian	54569	53859
[1257,]	-821	-824	Jiangsu Pizhou	Jiangsu Shuyang	58026	58038
[1258,]	-826	1257	Jiangsu Xiliandao	Shanxi Xiangfen	58041	53861
[1259,]	-827	1258	Jiangsu Lianyungang	Shanxi Lingshi	58044	53862
[1260,]	-828	1259	Jiangsu Xiangshui	Shanxi Jiexiu	58045	53863
[1261,]	-829	1260	Jiangsu Guanyun	Shanxi Puxian	58047	53864
[1262,]	-832	1261	Jiangsu Siyang	Shanxi Fenxi	58132	53865
[1263,]	-833	1262	Jiangsu Sihong	Shanxi Hongtong	58135	53866
[1264,]	-835	1263	Jiangsu Hongze	Shanxi Wuxiang	58139	53871
[1265,]	-836	1264	Jiangsu Lianshui	Shanxi Qinxian	58140	53872
[1266,]	-837	1265	Jiangsu Huaian	Shanxi Changzi	58141	53873
[1267,]	-838	1266	Jiangsu Funing	Shanxi Guxian	58143	53874
[1268,]	-839	1267	Jiangsu Jianhu	Shanxi Qinyuan	58146	53875
[1269,]	-841	1268	Jiangsu Baoying	Shanxi Anze	58148	53877
[1270,]	-844	1269	Jiangsu Liuhe	Shanxi Licheng	58235	53878
[1271,]	-848	1270	Jiangsu Yizheng	Shanxi Lucheng	58242	53880
[1272,]	-851	1271	Jiangsu Yangzhong	Shanxi Xiangning	58247	53953
[1273,]	-860	1272	Jiangsu Lvsi	Shanxi Jishan	58265	53954
[1274,]	-864	1273	Jiangsu Danyang	Shanxi Wanrong	58341	53956
[1275,]	-871	1274	Jiangsu Wuxi	Shanxi Hejin	58354	53957
[1276,]	-1181	1275	Shandong Yutai	Shanxi Yanhu	54907	53959
[1277,]	-849	-858	Jiangsu Xinghua	Jiangsu Nantong	58243	58259
[1278,]	-853	-863	Jiangsu Jiangyan	Jiangsu Lishui	58250	58340
[1279,]	-866	1278	Jiangsu Jurong	Shanxi Fushan	58344	53966
[1280,]	-868	1279	Jiangsu Suzhou	Shanxi Yuanqu	58349	53968
[1281,]	-861	-1355	Jiangsu Qidong	Shanghai Fengxian	58269	58463
[1282,]	-874	-1664	Jiangsu Taicang	Zhejiang Changxing	58377	58443
[1283,]	-877	-893	Jiangxi Wanzai	Jiangxi Ganxian	57698	57993
[1284,]	-918	1283	Jiangxi Guixi	Shanxi Yongji	58626	57052
[1285,]	-920	1284	Jiangxi Yushan	Shanxi Ruicheng	58634	57053
[1286,]	-921	1285	Jiangxi Xinjian	Shaanxi Fugu	58693	53567
[1287,]	-878	-914	Jiangxi Shanggao	Jiangxi Dongxiang	57699	58618
[1288,]	-892	-894	Jiangxi Nankang	Jiangxi Xinfeng	57992	57995
[1289,]	-908	-915	Jiangxi Anyi	Jiangxi Linchuan	58602	58619
[1290,]	-910	-913	Jiangxi Nanchang	Jiangxi Wannian	58606	58615
[1291,]	-923	1290	Jiangxi Yongfeng	Shaanxi Jingbian	58705	53735
[1292,]	-942	-949	Liaoning Zhangwu	Liaoning Jianpingzhen	54236	54321

[1293,]	-968	1292	Liaoning Xingcheng	Shaanxi Hengshan	54455	53740
[1294,]	-982	1293	Liaoning Zhuanghe	Shaanxi Zichang	54584	53748
[1295,]	-945	-947	Liaoning Shenbei	Liaoning Xifeng	54248	54252
[1296,]	-951	1295	Liaoning Yangshan	Shaanxi Wubao	54325	53756
[1297,]	-963	1296	Liaoning Fushun	Shaanxi Qingjian	54351	53757
[1298,]	-974	1297	Liaoning Kuandian	Shaanxi Zhidan	54493	53832
[1299,]	-976	1298	Liaoning Dandong	Shaanxi Ansai	54497	53841
[1300,]	-983	1299	Liaoning Changxingdao	Shaanxi Ganquan	54565	53848
[1301,]	-948	-1061	Liaoning Qingyuan	Inner Mongolia Qinglongshan	54259	54132
[1302,]	-1073	1301	Inner Mongolia Kezuohouqi	Shaanxi Yanchang	54231	53854
[1303,]	-966	-1529	Liaoning Lianshan	Xinjiang Yanqi	54453	51567
[1304,]	-1010	-1034	Inner Mongolia Wuyuan	Inner Mongolia Jining	53337	53480
[1305,]	-1038	1304	Inner Mongolia Linhe	Shaanxi Xunyi	53513	53938
[1306,]	-1012	-1028	Inner Mongolia Damaoqi	Inner Mongolia Tumutezuoqi	53352	53464
[1307,]	-1031	-1079	Inner Mongolia Helingeerxian	Ningxia Shitanjing	53469	53517
[1308,]	-1082	-1084	Ningxia Pingluo	Ningxia Taole	53611	53615
[1309,]	-1085	1308	Ningxia Yongning	Shaanxi Tongchuan	53618	53947
[1310,]	-1102	-1106	Qinghai Huzhu	Qinghai Minhe	52863	52876
[1311,]	-1104	-1127	Qinghai Guide	Qinghai Henan	52868	56065
[1312,]	-1113	-1114	Qinghai Jianzha	Qinghai Zeku	52963	52968
[1313,]	-1122	1312	Qinghai Maduo	Shaanxi Longxian	56033	57003
[1314,]	-1129	1313	Qinghai Nangqian	Shaanxi Baoji	56125	57016
[1315,]	-1134	-1164	Shandong Yangxin	Shandong Qingzhou	54723	54831
[1316,]	-1194	1315	Shandong Feixian	Shaanxi Linyou	54929	57022
[1317,]	-1135	-1137	Shandong Shanghe	Shandong Zhangqiu	54724	54727
[1318,]	-1138	1317	Shandong Gaoqing	Shaanxi Meixian	54729	57027
[1319,]	-1140	1318	Shandong Kenli	Shaanxi Liquan	54744	57029
[1320,]	-1144	1319	Shandong Longkou	Shaanxi Yongshou	54753	57030
[1321,]	-1145	1320	Shandong Zhaoyuan	Shaanxi Wugong	54755	57034
[1322,]	-1146	1321	Shandong Qixia	Shaanxi Yaoxian	54759	57037
[1323,]	-1152	1322	Shandong Liaocheng	Shaanxi Xingping	54806	57038
[1324,]	-1156	1323	Shandong Donge	Shaanxi Sanyuan	54815	57041
[1325,]	-1157	1324	Shandong Feicheng	Shaanxi Fuping	54819	57042
[1326,]	-1160	1325	Shandong Jinan	Shaanxi Dali	54823	57043
[1327,]	-1171	1326	Shandong Anqiu	Shaanxi Weinan	54844	57045
[1328,]	-1175	1327	Shandong Laiyang	Shaanxi Huashan	54852	57046
[1329,]	-1178	1328	Shandong Juancheng	Shaanxi Luonan	54904	57057
[1330,]	-1180	1329	Shandong Heze	Shaanxi Lueyang	54906	57106
[1331,]	-1183	1330	Shandong Liangshan	Shaanxi Fengxian	54910	57113

[1332,]	-1184	1331	Shandong Dongping	Shaanxi Mianxian	54911	57119
[1333,]	-1186	1332	Shandong Juye	Shaanxi Liuba	54914	57124
[1334,]	-1189	1333	Shandong Sishui	Shaanxi Chenggu	54920	57128
[1335,]	-1192	1334	Shandong Pingyi	Shaanxi Foping	54925	57134
[1336,]	-1198	1335	Shandong Wulian	Shaanxi Ningshan	54940	57137
[1337,]	-1199	1336	Shandong Huangdao	Shaanxi Zhashui	54943	57140
[1338,]	-1205	1337	Shandong Zaozhuang	Shaanxi Shangxian	58024	57143
[1339,]	-1206	1338	Shandong Cangshan	Shaanxi Danfeng	58030	57153
[1340,]	-1182	-1193	Shandong Dingtao	Shandong Tengzhou	54909	54927
[1341,]	-1188	-1200	Shandong Zoucheng	Shandong Rizhao	54919	54945
[1342,]	-1209	-1210	Shanxi Yanggao	Shanxi Datong	53486	53487
[1343,]	-1232	1342	Shanxi Dingxiang	Shaanxi Nanzheng	53676	57213
[1344,]	-1240	1343	Shanxi Fangshan	Shaanxi Ziyang	53760	57231
[1345,]	-1245	1344	Shanxi Qingxu	Shaanxi Hanyin	53774	57233
[1346,]	-1252	1345	Shanxi Heshun	Shaanxi Zhenba	53788	57238
[1347,]	-1275	1346	Shanxi Yanhu	Shaanxi Xunyang	53959	57242
[1348,]	-1278	1347	Shanxi Fushan	Shaanxi Ankang	53966	57245
[1349,]	-1274	-1309	Shanxi Hejin	Shaanxi Pucheng	53957	53948
[1350,]	-1314	1349	Shaanxi Qianyang	Shaanxi Pingli	57021	57248
[1351,]	-1315	1350	Shaanxi Linyou	Shaanxi Baihe	57022	57254
[1352,]	-1324	1351	Shaanxi Fuping	Shaanxi Zhenping	57042	57343
[1353,]	-1326	1352	Shaanxi Weinan	Shanghai Minhang	57045	58361
[1354,]	-1288	-1295	Shaanxi Jiaxian	Shaanxi Wubao	53658	53756
[1355,]	-1296	1354	Shaanxi Qingjian	Shanghai Jinshan	53757	58460
[1356,]	-1308	1355	Shaanxi Tongchuan	Shanghai Fengxian	53947	58463
[1357,]	-1311	1356	Shaanxi Heyang	Sichuan Shiqu	53950	56038
[1358,]	-1320	1357	Shaanxi Wugong	Sichuan Ruoergai	57034	56079
[1359,]	-1325	1358	Shaanxi Dali	Sichuan Jiuzhaigou	57043	56097
[1360,]	-1328	1359	Shaanxi Luonan	Sichuan Dege	57057	56144
[1361,]	-1333	1360	Shaanxi Chenggu	Sichuan Ganzi	57128	56146
[1362,]	-1336	1361	Shaanxi Zhashui	Sichuan Baiyu	57140	56147
[1363,]	-1346	1362	Shaanxi Xunyang	Sichuan Seda	57242	56152
[1364,]	-1477	1363	Tianjin Dagang	Sichuan Luhuo	54645	56158
[1365,]	-1300	-1317	Shaanxi Yanchuan	Shaanxi Meixian	53850	57027
[1366,]	-1318	1365	Shaanxi Liquan	Sichuan Daofu	57029	56167
[1367,]	-1319	1366	Shaanxi Yongshou	Sichuan Jinchuan	57030	56168
[1368,]	-1321	1367	Shaanxi Yaoxian	Sichuan Aba	57037	56171
[1369,]	-1323	1368	Shaanxi Sanyuan	Sichuan Maerkang	57041	56172
[1370,]	-1331	1369	Shaanxi Mianxian	Sichuan Hongyuan	57119	56173
[1371,]	-1341	-1422	Shaanxi Ningqiang	Sichuan Mabian	57211	56480
[1372,]	-1429	1371	Sichuan Xingwen	Sichuan Maoxian	56496	56180
[1373,]	-1448	1372	Sichuan Wanyuan	Sichuan Chongzhou	57237	56181
[1374,]	-1352	-1353	Shanghai Minhang	Shanghai Baoshan	58361	58362

[1375,]	-1354	-1667	Shanghai Jinshan	Zhejiang Fuyang	58460	58449
[1376,]	-1674	1375	Zhejiang Cixi	Sichuan Lixian	58467	56184
[1377,]	-1677	1376	Zhejiang Kaihua	Sichuan Heishui	58537	56185
[1378,]	-1686	1377	Zhejiang Dongyang	Sichuan Mianzhu	58558	56186
[1379,]	-1690	1378	Zhejiang Yinzhou	Sichuan Wenjiang	58562	56187
[1380,]	-1359	-1479	Sichuan Dege	Tibet Gaize	56144	55248
[1381,]	-1481	1380	Tibet Pulan	Sichuan Pengzhou	55437	56189
[1382,]	-1486	1381	Tibet Zedang	Sichuan Deyang	55598	56198
[1383,]	-1488	1382	Tibet Dingri	Sichuan Batang	55664	56247
[1384,]	-1490	1383	Tibet Dingqing	Sichuan Xinlong	56116	56251
[1385,]	-1493	1384	Tibet Luolong	Sichuan Litang	56223	56257
[1386,]	-1494	1385	Tibet Bomi	Sichuan Danba	56227	56263
[1387,]	-1498	1386	Tibet Zuogong	Sichuan Yajiang	56331	56267
[1388,]	-1499	1387	Tibet Chayu	Sichuan Baoxing	56434	56273
[1389,]	-1361	-1478	Sichuan Baiyu	Tibet Shiquanhe	56147	55228
[1390,]	-1495	1389	Tibet Basu	Sichuan Mingshan	56228	56280
[1391,]	-1496	1390	Tibet Linzhi	Sichuan Pujiang	56312	56281
[1392,]	-1371	-1372	Sichuan Maoxian	Sichuan Chongzhou	56180	56181
[1393,]	-1374	1392	Sichuan Wenchuan	Sichuan Pengshan	56183	56289
[1394,]	-1377	1393	Sichuan Mianzhu	Sichuan Jintang	56186	56296
[1395,]	-1378	1394	Sichuan Wenjiang	Sichuan Renshou	56187	56297
[1396,]	-1380	1395	Sichuan Pengzhou	Sichuan Ziyang	56189	56298
[1397,]	-1381	1396	Sichuan Deyang	Sichuan Daocheng	56198	56357
[1398,]	-1389	1397	Sichuan Mingshan	Sichuan Luding	56280	56371
[1399,]	-1390	1398	Sichuan Pujiang	Sichuan Yingjing	56281	56373
[1400,]	-1391	1399	Sichuan Longquanyi	Sichuan Kangding	56286	56374
[1401,]	-1392	1400	Sichuan Pengshan	Sichuan Hanyuan	56289	56376
[1402,]	-1393	1401	Sichuan Jintang	Sichuan Shimian	56296	56378
[1403,]	-1394	1402	Sichuan Renshou	Sichuan Hongya	56297	56380
[1404,]	-1395	1403	Sichuan Ziyang	Sichuan Jiajiang	56298	56382
[1405,]	-1398	1404	Sichuan Yingjing	Sichuan Qingshen	56373	56383
[1406,]	-1399	1405	Sichuan Kangding	Sichuan Emeishan	56374	56385
[1407,]	-1400	1406	Sichuan Hanyuan	Sichuan Ebian	56376	56387
[1408,]	-1403	1407	Sichuan Jiajiang	Sichuan Qianwei	56382	56389
[1409,]	-1404	1408	Sichuan Qingshen	Sichuan Jingyan	56383	56390
[1410,]	-1405	1409	Sichuan Emeishan	Sichuan Zizhong	56385	56393
[1411,]	-1406	1410	Sichuan Ebian	Sichuan Weiyuan	56387	56395
[1412,]	-1407	1411	Sichuan Qianwei	Sichuan Zigong	56389	56396
[1413,]	-1409	1412	Sichuan Zizhong	Sichuan Fushun	56393	56399
[1414,]	-1410	1413	Sichuan Weiyuan	Sichuan Derong	56395	56441
[1415,]	-1411	1414	Sichuan Zigong	Sichuan Xiangcheng	56396	56443
[1416,]	-1412	1415	Sichuan Fushun	Sichuan Muli	56399	56459
[1417,]	-1418	1416	Sichuan Mianning	Sichuan Jiulong	56474	56462

[1418,]	-1419	1417	Sichuan Yuexi	Sichuan Ganluo	56475	56473
[1419,]	-1426	1418	Sichuan Yibinxian	Sichuan Mianning	56491	56474
[1420,]	-1427	1419	Sichuan Nanxi	Sichuan Yuexi	56493	56475
[1421,]	-1431	1420	Sichuan Yanyuan	Sichuan Xide	56565	56478
[1422,]	-1433	1421	Sichuan Xichang	Sichuan Chaojue	56571	56479
[1423,]	-1434	1422	Sichuan Puge	Sichuan Mabian	56575	56480
[1424,]	-1435	1423	Sichuan Butuo	Sichuan Leibo	56580	56485
[1425,]	-1437	1424	Sichuan Changning	Sichuan Meigu	56593	56487
[1426,]	-1438	1425	Sichuan Yanbian	Sichuan Muchuan	56665	56490
[1427,]	-1440	1426	Sichuan Miyi	Sichuan Yibinxian	56670	56491
[1428,]	-1441	1427	Sichuan Huili	Sichuan Nanxi	56671	56493
[1429,]	-1442	1428	Sichuan Huidong	Sichuan Pingshan	56675	56494
[1430,]	-1444	1429	Sichuan Guangyuan	Sichuan Xingwen	57206	56496
[1431,]	-1446	1430	Sichuan Nanjiang	Sichuan Gongxian	57216	56499
[1432,]	-1447	1431	Sichuan Wangcang	Sichuan Yanyuan	57217	56565
[1433,]	-1449	1432	Sichuan Langzhong	Sichuan Dechang	57306	56569
[1434,]	-1450	1433	Sichuan Xichong	Sichuan Xichang	57309	56571
[1435,]	-1451	1434	Sichuan Bazhong	Sichuan Puge	57313	56575
[1436,]	-1452	1435	Sichuan Nanbu	Sichuan Butuo	57314	56580
[1437,]	-1453	1436	Sichuan Yilong	Sichuan Jinyang	57315	56584
[1438,]	-1454	1437	Sichuan Yingshan	Sichuan Changning	57318	56593
[1439,]	-1455	1438	Sichuan Tongjiang	Sichuan Yanbian	57320	56665
[1440,]	-1456	1439	Sichuan Pingchang	Sichuan Panzhihua	57324	56666
[1441,]	-1458	1440	Sichuan Kaijiang	Sichuan Miyi	57329	56670
[1442,]	-1459	1441	Sichuan Shehong	Sichuan Huili	57401	56671
[1443,]	-1460	1442	Sichuan Suining	Sichuan Huidong	57405	56675
[1444,]	-1461	1443	Sichuan Gaoping	Sichuan Qingchuan	57411	57204
[1445,]	-1462	1444	Sichuan Quxian	Sichuan Guangyuan	57413	57206
[1446,]	-1463	1445	Sichuan Guangan	Sichuan Jiange	57415	57208
[1447,]	-1466	1446	Sichuan Dazhu	Sichuan Nanjiang	57420	57216
[1448,]	-1467	1447	Sichuan Dongxing	Sichuan Wangcang	57503	57217
[1449,]	-1468	1448	Sichuan Longchang	Sichuan Wanyuan	57507	57237
[1450,]	-1469	1449	Sichuan Jiangan	Sichuan Langzhong	57600	57306
[1451,]	-1470	1450	Sichuan Hejiang	Sichuan Xichong	57603	57309
[1452,]	-1471	1451	Sichuan Xuyong	Sichuan Bazhong	57608	57313
[1453,]	-1588	1452	Yunnan Qiaojia	Sichuan Nanbu	56673	57314
[1454,]	-1723	1453	Chongqing Tongnan	Sichuan Yilong	57409	57315
[1455,]	-1728	1454	Chongqing Dazu	Sichuan Yingshan	57502	57318
[1456,]	-1729	1455	Chongqing Rongchang	Sichuan Tongjiang	57505	57320
[1457,]	-1730	1456	Chongqing Yongchuan	Sichuan Pingchang	57506	57324
[1458,]	-1379	-1408	Sichuan Dujiangyan	Sichuan Jingyan	56188	56390
[1459,]	-1457	1458	Sichuan Dachuan	Sichuan Kaijiang	57328	57329
[1460,]	-1401	-1425	Sichuan Shimian	Sichuan Muchuan	56378	56490

[1461,]	-1416	-1423	Sichuan Jiulong	Sichuan Leibo	56462	56485
[1462,]	-1465	-1719	Sichuan Wusheng	Chongqing Kaixian	57417	57338
[1463,]	-1472	-1476	Tianjin Wuqing	Tianjin Tanggu	54523	54623
[1464,]	-1520	-1540	Xinjiang Fukang	Xinjiang Jiashi	51377	51707
[1465,]	-1534	-1538	Xinjiang Akesu	Xinjiang Atushi	51628	51704
[1466,]	-1536	-1544	Xinjiang Shaya	Xinjiang Awati	51639	51722
[1467,]	-1566	-1573	Yunnan Deqin	Yunnan Daguan	56444	56582
[1468,]	-1578	-1612	Yunnan Fugong	Yunnan Yingjiang	56641	56836
[1469,]	-1590	-1610	Yunnan Xuanwei	Yunnan Fuyuan	56697	56790
[1470,]	-1611	-1613	Yunnan Longchuan	Yunnan Zhenkang	56835	56839
[1471,]	-1665	-1666	Zhejiang Anji	Zhejiang Linan	58446	58448
[1472,]	-1668	1471	Zhejiang Huzhou	Sichuan Xuyong	58450	57608
[1473,]	-1669	1472	Zhejiang Jiashan	Tianjin Wuqing	58451	54523
[1474,]	-1670	1473	Zhejiang Jiaxing	Tianjin Baodi	58452	54525
[1475,]	-1671	1474	Zhejiang Shaoxing	Tianjin Jinghai	58453	54619
[1476,]	-1672	1475	Zhejiang Deqing	Tianjin Jinnan	58454	54622
[1477,]	-1673	1476	Zhejiang Hangzhou	Tianjin Tanggu	58457	54623
[1478,]	-1678	1477	Zhejiang Tonglu	Tianjin Dagang	58542	54645
[1479,]	-1679	1478	Zhejiang Jiande	Tibet Shiquanhe	58544	55228
[1480,]	-1680	1479	Zhejiang Pujiang	Tibet Gaize	58546	55248
[1481,]	-1681	1480	Zhejiang Longyou	Tibet Naqu	58547	55299
[1482,]	-1682	1481	Zhejiang Jinhua	Tibet Pulan	58549	55437
[1483,]	-1683	1482	Zhejiang Zhuji	Tibet Dangxiong	58550	55493
[1484,]	-1684	1483	Zhejiang Xinchang	Tibet Lazi	58555	55569
[1485,]	-1685	1484	Zhejiang Shengzhou	Tibet Nanmulin	58556	55572
[1486,]	-1687	1485	Zhejiang Tiantai	Tibet Muozhugongka	58559	55593
[1487,]	-1688	1486	Zhejiang Panan	Tibet Zedang	58560	55598
[1488,]	-1691	1487	Zhejiang Fenghua	Tibet Nielamu	58565	55655
[1489,]	-1692	1488	Zhejiang Xiangshan	Tibet Dingri	58566	55664
[1490,]	-1693	1489	Zhejiang Ninghai	Tibet Jiangzi	58567	55680
[1491,]	-1695	1490	Zhejiang Shipu	Tibet Dingqing	58569	56116
[1492,]	-1696	1491	Zhejiang Putuo	Tibet Leiwuqi	58570	56128
[1493,]	-1699	1492	Zhejiang Wuyi	Tibet Changdu	58642	56137
[1494,]	-1700	1493	Zhejiang Yongkang	Tibet Luolong	58643	56223
[1495,]	-1702	1494	Zhejiang Lishui	Tibet Bomi	58646	56227
[1496,]	-1704	1495	Zhejiang Xianju	Tibet Basu	58652	56228
[1497,]	-1705	1496	Zhejiang Jinyun	Tibet Linzhi	58654	56312
[1498,]	-1706	1497	Zhejiang Leqing	Tibet Miling	58656	56317
[1499,]	-1707	1498	Zhejiang Qingtian	Tibet Zuogong	58657	56331
[1500,]	-1708	1499	Zhejiang Yongjia	Tibet Chayu	58658	56434
[1501,]	-1709	1500	Zhejiang Linhai	Xinjiang Habahe	58660	51053
[1502,]	-1711	1501	Zhejiang Hongjia	Xinjiang Akedala	58665	51058
[1503,]	-1712	1502	Zhejiang Dachen	Xinjiang Buerjin	58666	51060

[1504,]	-1716	1503	Zhejiang Pingyang	Xinjiang Fuhai	58751	51068
[1505,]	-1694	-1703	Zhejiang Sanmen	Zhejiang Longquan	58568	58647
[1506,]	-1697	-1698	Zhejiang Changshan	Zhejiang Jiangshan	58631	58632
[1507,]	-1713	-1717	Zhejiang Yunhe	Zhejiang Jingning	58742	58648
[1508,]	81	94	Fujian Ninghuai	Fujian Changting	58818	58911
[1509,]	-428	551	Hebei Xinglong	Henan Guangshan	54425	57299
[1510,]	506	1465	Henan Yichuan	Sichuan Wusheng	57074	57417
[1511,]	114	1276	Fujian Chongwu	Shanxi Xinjiang	59133	53964
[1512,]	161	255	Gansu Maqu	Guangxi Lingui	56074	57954
[1513,]	211	593	Guangdong Conghua	Heilongjiang Hegang	59285	50775
[1514,]	-81	246	Fujian Ninghuai	Guangdong Maoming	58818	59659
[1515,]	-76	1289	Fujian Shouning	Shaanxi Dingbian	58744	53725
[1516,]	302	607	Guangxi Bobai	Heilongjiang Lansi	59449	50859
[1517,]	-116	-185	Fujian Yunxiao	Guangdong Lechang	59322	57988
[1518,]	462	508	Hebei Cangzhou	Henan Ruyang	54616	57078
[1519,]	464	1309	Hebei Huanghua	Shaanxi Pucheng	54624	53948
[1520,]	-151	-1035	Gansu Zhuanglang	Inner Mongolia Chayouqianqi	53917	53481
[1521,]	510	1310	Henan Gongyi	Shaanxi Chengcheng	57080	53949
[1522,]	511	978	Henan Xingyang	Liaoning Jinzhou	57081	54568
[1523,]	-249	-310	Guangxi Ziyuan	Guizhou Weining	57859	56691
[1524,]	637	818	Heilongjiang Hailin	Jilin Changbai	54092	54386
[1525,]	647	655	Hubei Fangxian	Hubei Shennongjia	57259	57362
[1526,]	724	1150	Hunan Pingjiang	Shandong Wendeng	57682	54777
[1527,]	-1436	816	Sichuan Jinyang	Jilin Linjiang	56584	54374
[1528,]	-383	-1576	Hainan Wanning	Yunnan Zhenxiong	59951	56595
[1529,]	-393	1370	Hebei Laiyuan	Sichuan Xiaojin	53599	56178
[1530,]	888	962	Jiangxi Wanan	Liaoning Benxi	57895	54346
[1531,]	890	924	Jiangxi Taihe	Jiangxi Lean	57899	58706
[1532,]	925	1316	Jiangxi Chongren	Shaanxi Fufeng	58710	57026
[1533,]	-590	1092	Heilongjiang Suileng	Ningxia Tongxin	50767	53810
[1534,]	-583	1126	Heilongjiang Fuyu	Qinghai Dari	50742	56046
[1535,]	-605	1116	Heilongjiang Anda	Qinghai Tongren	50854	52974
[1536,]	-627	1125	Heilongjiang Yanshou	Qinghai Gander	50965	56045
[1537,]	-667	1140	Hubei Jianshi	Shandong Kenli	57445	54744
[1538,]	-663	-683	Hubei Anlu	Hubei Tuanfeng	57388	57495
[1539,]	1202	1210	Shandong Chengwu	Shanxi Datong	58003	53487
[1540,]	-713	-729	Hunan Yuanling	Hunan Zhijiang	57655	57745
[1541,]	-721	1213	Hunan Xiangyin	Shanxi Pinglu	57673	53574
[1542,]	-769	1215	Hunan Rucheng	Shanxi Shanyin	57985	53576
[1543,]	-840	1282	Jiangsu Jinhu	Shanxi Lingchuan	58147	53981
[1544,]	-895	1287	Jiangxi Jiujiang	Shaanxi Shenmu	58502	53651
[1545,]	1294	1303	Shaanxi Suide	Shaanxi Fuxian	53754	53931

[1546,]	1300	1302	Shaanxi Yanchuan	Shaanxi Yichuan	53850	53857
[1547,]	-950	-1071	Liaoning Chaoyang	Inner Mongolia Aohanqi	54324	54225
[1548,]	-1211	-1271	Shanxi Hequ	Shanxi Xiangning	53564	53953
[1549,]	1379	1504	Sichuan Dujiangyan	Xinjiang Aletai	56188	51076
[1550,]	-1445	1460	Sichuan Jiange	Sichuan Suining	57208	57405
[1551,]	1467	1527	Sichuan Dongxing	Xinjiang Kumishi	57503	51526
[1552,]	-1718	1526	Chongqing Chengkou	Xinjiang Mulei	57333	51482
[1553,]	-1273	1510	Shanxi Wanrong	Xinjiang Alashankou	53956	51232
[1554,]	1507	1549	Xinjiang Yumin	Xinjiang Maigaiti	51137	51810
[1555,]	1506	1512	Xinjiang Tacheng	Xinjiang Touli	51133	51241
[1556,]	95	1508	Fujian Wuping	Xinjiang Emin	58917	51145
[1557,]	-620	1124	Heilongjiang Haerbin	Qinghai Maqin	50953	56043
[1558,]	1516	1542	Xinjiang Manasi	Xinjiang Yuepuhu	51359	51717
[1559,]	213	1391	Guangdong Dongguan	Sichuan Longquanyi	59289	56286
[1560,]	1130	1388	Qinghai Banma	Sichuan Lushan	56151	56279
[1561,]	1339	1513	Shaanxi Shangnan	Xinjiang Kelamayi	57154	51243
[1562,]	1016	1511	Inner Mongolia Wuchuanxian	Xinjiang Bole	53368	51238
[1563,]	-1081	1519	Ningxia Helan	Xinjiang Miquan	53610	51369
[1564,]	1353	1548	Shanghai Baoshan	Xinjiang Yengjisha	58362	51802
[1565,]	1554	1556	Xinjiang Cele	Xinjiang Hetan	51826	51828
[1566,]	1518	1529	Xinjiang Changji	Xinjiang Yanqi	51368	51567
[1567,]	-1724	1539	Chongqing Dianjiang	Xinjiang Wuqia	57425	51705
[1568,]	-62	1314	Beijing Miyunshangdianzi	Shaanxi Qianyang	54421	57021
[1569,]	-1285	214	Shaanxi Fugu	Guangdong Longmen	53567	59290
[1570,]	-1283	918	Shanxi Yongji	Jiangxi Guixi	57052	58626
[1571,]	930	1280	Jiangxi Xingguo	Shanxi Qingshui	58804	53970
[1572,]	-1202	961	Shandong Chengwu	Liaoning Liaoyangxian	58003	54345
[1573,]	-1203	1572	Shandong Xuechen	Yunnan Ninglang	58021	56567
[1574,]	1115	1364	Qinghai Xunhua	Sichuan Rangtang	52972	56164
[1575,]	-961	-1302	Liaoning Liaoyangxian	Shaanxi Yichuan	54345	53857
[1576,]	378	1522	Hainan Dingan	Xinjiang Tianshandaxigou	59851	51468
[1577,]	644	1551	Hubei Yunxian	Xinjiang Yecheng	57253	51814
[1578,]	-499	1567	Henan Sanmenxia	Yunnan Suijiang	57051	56483
[1579,]	41	1578	Anhui Wuhu	Yunnan Fugong	58334	56641
[1580,]	1288	1517	Shaanxi Jiaxian	Xinjiang Hutubi	53658	51367
[1581,]	656	1525	Hubei Nanzhang	Xinjiang Dabancheng	57363	51477
[1582,]	1561	1562	Xinjiang Balikun	Xinjiang Naomaohu	52101	52112
[1583,]	509	1340	Henan Wenxian	Shaanxi Shanyang	57079	57155
[1584,]	731	975	Hunan Hongjiang	Liaoning Fengcheng	57754	54494
[1585,]	-1083	919	Ningxia Wuzhong	Jiangxi Qianshan	53612	58629
[1586,]	-452	1277	Hebei Xushui	Shanxi Jiangxian	54601	53965
[1587,]	1228	1463	Shanxi Lanxian	Sichuan Guangan	53665	57415

[1588,]	548	1523	Henan Zhengyang	Xinjiang Urumqi Pastoral Test Station	57295	51469
[1589,]	-1675	1528	Zhejiang Shengsi	Xinjiang Bayinbuluke	58472	51542
[1590,]	5	1017	Anhui Taihe	Inner Mongolia Chayouzhongqi	58109	53378
[1591,]	1521	1547	Xinjiang Jimusaer	Xinjiang Tieqianlike	51378	51765
[1592,]	-1663	648	Yunnan Funing	Hubei Danjiangkou	59205	57260
[1593,]	-412	1144	Hebei Handan	Shandong Longkou	53892	54753
[1594,]	980	981	Liaoning Pikou	Liaoning Changhai	54575	54579
[1595,]	1087	1306	Ningxia Zhongning	Shaanxi Huangling	53705	53944
[1596,]	-596	1128	Heilongjiang Fuyuan	Qinghai Jiuzhi	50779	56067
[1597,]	-788	1221	Jilin Gujiazi	Shanxi Fanshi	54155	53585
[1598,]	-818	-1654	Jilin Changbai	Yunnan Jiangcheng	54386	56977
[1599,]	-1487	1470	Tibet Nielamu	Sichuan Hejiang	55655	57603
[1600,]	-1524	331	Xinjiang Tianchi	Guizhou Shiqian	51470	57734
[1601,]	1530	1546	Xinjiang Tuokexun	Xinjiang Tazhong	51571	51747
[1602,]	-1518	1532	Xinjiang Changji	Xinjiang Tulufan	51368	51573
[1603,]	608	1524	Heilongjiang Qingan	Xinjiang Tianchi	50861	51470
[1604,]	-252	1603	Guangxi Longsheng	Yunnan Fumin	57942	56772
[1605,]	1574	1575	Yunnan Ludian	Yunnan Yiliang	56585	56594
[1606,]	-212	1558	Guangdong Guangzhou	Xinjiang Minfeng	59287	51839
[1607,]	1553	1582	Xinjiang Pishan	Yunnan Eryuan	51818	56649
[1608,]	1531	1583	Xinjiang Tulufandongkan	Yunnan Lijiang	51572	56651
[1609,]	1566	1576	Yunnan Deqin	Yunnan Zhenxiong	56444	56595
[1610,]	1571	1601	Yunnan Weixi	Yunnan Mouding	56548	56766
[1611,]	824	1589	Jiangsu Shuyang	Yunnan Dongchuan	58038	56688
[1612,]	-248	-1701	Guangdong Xuwen	Zhejiang Suichang	59754	58644
[1613,]	1249	1597	Shanxi Yangquan	Yunnan Binchuan	53782	56752
[1614,]	1348	1459	Shaanxi Langao	Sichuan Shehong	57247	57401
[1615,]	-1503	1461	Xinjiang Fuhai	Sichuan Gaoping	51068	57411
[1616,]	966	1538	Liaoning Lianshan	Xinjiang Atushi	54453	51704
[1617,]	1552	1565	Xinjiang Zepu	Xinjiang Hongliuhe	51815	52313
[1618,]	-657	1550	Hubei Yicheng	Xinjiang Shashe	57370	51811
[1619,]	867	1568	Jiangsu Yixing	Yunnan Yanjin	58346	56497
[1620,]	1535	1557	Xinjiang Xinhe	Xinjiang Luopu	51636	51829
[1621,]	1537	1569	Xinjiang Kuerle	Yunnan Gongshan	51656	56533
[1622,]	-959	1587	Liaoning Sujiatun	Yunnan Yongren	54340	56669
[1623,]	1509	1559	Xinjiang Hebukesaier	Xinjiang Qiemuo	51156	51855
[1624,]	1041	1560	Inner Mongolia Hangjinqi	Xinjiang Yutian	53533	51931
[1625,]	-1628	643	Yunnan Chengjiang	Hubei Yunxi	56873	57251
[1626,]	-692	1457	Hubei Jiayu	Sichuan Dachuan	57583	57328
[1627,]	1577	1604	Yunnan Weixin	Yunnan Wuding	56596	56774
[1628,]	1545	1614	Xinjiang Alaer	Yunnan Lianghe	51730	56840

[1629,]	-1676	1541	Zhejiang Daishan	Xinjiang Kashi	58484	51709
[1630,]	-154	1616	Gansu Zhenyuan	Yunnan Shidian	53925	56842
[1631,]	-636	983	Heilongjiang Wuchang	Liaoning Changxingdao	54080	54565
[1632,]	1341	1591	Shaanxi Ningqiang	Yunnan Tengchong	57211	56739
[1633,]	1468	1599	Sichuan Longchang	Yunnan Weishan	57507	56757
[1634,]	1588	1607	Yunnan Qiaojia	Yunnan Malong	56673	56782
[1635,]	1373	1621	Sichuan Songpan	Yunnan Jingdong	56182	56856
[1636,]	-542	1629	Henan Tanghe	Yunnan Yuxi	57273	56875
[1637,]	1520	1608	Xinjiang Fukang	Yunnan Qujing	51377	56783
[1638,]	1564	1590	Xinjiang Hami	Yunnan Xuanwei	52203	56697
[1639,]	1555	1580	Xinjiang Moyu	Yunnan Lanping	51827	56645
[1640,]	865	1466	Jiangsu Jintan	Sichuan Dazhu	58342	57420
[1641,]	-1002	1093	Inner Mongolia Tuquan	Ningxia Guyuan	50934	53817
[1642,]	1305	1307	Shaanxi Baishui	Shaanxi Huanglong	53941	53946
[1643,]	-1430	1505	Sichuan Gongxian	Xinjiang Fuyun	56499	51087
[1644,]	1311	1600	Shaanxi Heyang	Yunnan Yaoan	53950	56764
[1645,]	1610	1628	Yunnan Fuyuan	Yunnan Chengjiang	56790	56873
[1646,]	1595	1640	Yunnan Baoshan	Yunnan Gengma	56748	56946
[1647,]	-619	1622	Heilongjiang Zhaozhou	Yunnan Shuangbai	50950	56862
[1648,]	1033	1605	Inner Mongolia Liangcheng	Yunnan Lufeng	53475	56777
[1649,]	-110	1286	Fujian Pinghe	Shaanxi Yulin	59125	53646
[1650,]	-201	-1710	Guangdong Jiaoling	Zhejiang Wenling	59114	58664
[1651,]	-1519	1462	Xinjiang Miquan	Sichuan Quxian	51369	57413
[1652,]	827	1611	Jiangsu Lianyungang	Yunnan Longchuan	58044	56835
[1653,]	-765	1540	Hunan Lanshan	Xinjiang Jiashi	57975	51707
[1654,]	-593	1631	Heilongjiang Hegang	Yunnan Yiliang	50775	56880
[1655,]	1515	1612	Xinjiang Shawan	Yunnan Yingjiang	51357	56836
[1656,]	1615	1630	Yunnan Longling	Yunnan Huaning	56841	56879
[1657,]	-789	1534	Jilin Changchun	Xinjiang Akesu	54161	51628
[1658,]	1543	1634	Xinjiang Kepin	Yunnan Mile	51720	56885
[1659,]	1211	1563	Shanxi Hequ	Xinjiang Yiwu	53564	52118
[1660,]	-967	1632	Liaoning Suizhong	Yunnan Shilin	54454	56881
[1661,]	1464	1618	Sichuan Linshui	Yunnan Fengqing	57416	56846
[1662,]	1593	1642	Yunnan Yangbi	Yunnan Menglian	56745	56949
[1663,]	1626	1643	Yunnan Yimen	Yunnan Shuangjiang	56870	56950
[1664,]	1606	1617	Yunnan Kunming	Yunnan Changning	56778	56843
[1665,]	1609	1658	Yunnan Songming	Yunnan Jinping	56785	56987
[1666,]	1573	1579	Yunnan Daguan	Yunnan Liuku	56582	56643
[1667,]	1256	1623	Shanxi Jiaokou	Yunnan Anning	53860	56863
[1668,]	1644	1652	Yunnan Lincang	Yunnan Shiping	56951	56970
[1669,]	-1689	1655	Zhejiang Zhenhai	Yunnan Luchun	58561	56978
[1670,]	1533	1656	Xinjiang Wushi	Yunnan Kaiyuan	51627	56982

[1671,]	1639	1664	Yunnan Cangyuan	Zhejiang Changxing	56944	58443
[1672,]	-786	1659	Jilin Shuangliao	Yunnan Yanshan	54142	56991
[1673,]	-258	1627	Guangxi Guanyang	Yunnan Jinning	57964	56871
[1674,]	1374	1619	Sichuan Wenchuan	Yunnan Yongde	56183	56849
[1675,]	1536	1654	Xinjiang Shaya	Yunnan Jiangcheng	51639	56977
[1676,]	-239	1291	Guangdong Haifeng	Shaanxi Wuqi	59500	53738
[1677,]	1099	1635	Qinghai Doulan	Yunnan Luxi	52836	56886
[1678,]	1646	1665	Yunnan Lancang	Zhejiang Anji	56954	58446
[1679,]	1514	1650	Xinjiang Jinghe	Yunnan Yuanjiang	51334	56966
[1680,]	1637	1672	Yunnan Luoping	Zhejiang Deqing	56891	58454
[1681,]	1586	1653	Yunnan Huaping	Yunnan Yuanyang	56664	56976
[1682,]	1570	1645	Yunnan Xianggelila	Yunnan Jinggu	56543	56952
[1683,]	1584	1638	Yunnan Yongsheng	Yunnan Eshan	56652	56898
[1684,]	-1027	1585	Inner Mongolia Huhehaote	Yunnan Heqing	53463	56654
[1685,]	-575	1674	Heilongjiang Wudalianchi	Zhejiang Cixi	50655	58467
[1686,]	1602	1641	Yunnan Nanhua	Yunnan Ximeng	56767	56948
[1687,]	1594	1680	Yunnan Yongping	Zhejiang Pujiang	56746	58546
[1688,]	-1492	1624	Tibet Changdu	Yunnan Zhenyuan	56137	56867
[1689,]	1636	1676	Yunnan Qiubei	Zhejiang Daishan	56889	58484
[1690,]	1620	1667	Yunnan Yunxian	Zhejiang Fuyang	56854	58449
[1691,]	1647	1663	Yunnan Menghai	Yunnan Funing	56958	59205
[1692,]	908	1682	Jiangxi Anyi	Zhejiang Jinhua	58602	58549
[1693,]	-807	-953	Jilin Donggang	Liaoning Lingyuan	54284	54327
[1694,]	1598	1688	Yunnan Midu	Zhejiang Panan	56755	58560
[1695,]	652	1581	Hubei Padang	Yunnan Jianchuan	57355	56646
[1696,]	1651	1661	Yunnan Mengla	Yunnan Wenshan	56969	56994
[1697,]	1648	1691	Yunnan Mojiang	Zhejiang Fenghua	56962	58565
[1698,]	-746	638	Hunan Dongan	Heilongjiang Muling	57867	54093
[1699,]	164	1690	Gansu Zhuoni	Zhejiang Yinzhou	56082	58562
[1700,]	-1504	1668	Xinjiang Aletai	Zhejiang Huzhou	51076	58450
[1701,]	1675	1684	Zhejiang Shengsi	Zhejiang Xinchang	58472	58555
[1702,]	1681	1686	Zhejiang Longyou	Zhejiang Dongyang	58547	58558
[1703,]	-902	1671	Jiangxi Duchang	Zhejiang Shaoxing	58517	58453
[1704,]	1666	1703	Zhejiang Linan	Zhejiang Longquan	58448	58647
[1705,]	1669	1679	Zhejiang Jiashan	Zhejiang Jiande	58451	58544
[1706,]	-1658	1592	Yunnan Jinping	Yunnan Yunlong	56987	56742
[1707,]	1678	1704	Zhejiang Tonglu	Zhejiang Xianju	58542	58652
[1708,]	1677	1683	Zhejiang Kaihua	Zhejiang Zhuji	58537	58550
[1709,]	-1569	1633	Yunnan Gongshan	Yunnan Shizong	56533	56883
[1710,]	1673	1709	Zhejiang Hangzhou	Zhejiang Linhai	58457	58660
[1711,]	1662	1695	Yunnan Guangnan	Zhejiang Shipu	59007	58569
[1712,]	1613	1699	Yunnan Zhenkang	Zhejiang Wuyi	56839	58642

[1713,]	1216	1711	Shanxi Ningwu	Zhejiang Hongjia	53577	58665
[1714,]	1657	1701	Yunnan Gejiu	Zhejiang Suichang	56984	58644
[1715,]	1233	1670	Shanxi Jiancaoping	Zhejiang Jiaxing	53677	58452
[1716,]	1697	1710	Zhejiang Changshan	Zhejiang Wenling	58631	58664
[1717,]	1596	1694	Yunnan Dali	Zhejiang Sanmen	56751	58568
[1718,]	1625	1698	Yunnan Xinping	Zhejiang Jiangshan	56869	58632
[1719,]	1660	1696	Yunnan Xichou	Zhejiang Putuo	56992	58570
[1720,]	1700	1707	Zhejiang Yongkang	Zhejiang Qingtian	58643	58657
[1721,]	1717	1718	Zhejiang Jingning	Chongqing Chengkou	58648	57333
[1722,]	1687	1715	Zhejiang Tiantai	Zhejiang Wencheng	58559	58750
[1723,]	1469	1706	Sichuan Jiangan	Zhejiang Leqing	57600	58656
[1724,]	1689	1702	Zhejiang Zhenhai	Zhejiang Lishui	58561	58646
[1725,]	1716	1720	Zhejiang Pingyang	Chongqing Yunyang	58751	57339
[1726,]	1692	1705	Zhejiang Xiangshan	Zhejiang Jinyun	58566	58654
[1727,]	1712	1721	Zhejiang Dachen	Chongqing Wuxi	58666	57345
[1728,]	1649	1722	Yunnan Simao	Chongqing Wushan	56964	57349
[1729,]	1713	1714	Zhejiang Yunhe	Zhejiang Taishun	58742	58746
[1730,]	-570	1719	Heilongjiang Aihui	Chongqing Kaixian	50468	57338
[1731,]	1544	1724	Xinjiang Awati	Chongqing Dianjiang	51722	57425
[1732,]	1281	1693	Shanxi Gaoping	Zhejiang Ninghai	53973	58567
[1733,]	-610	1723	Heilongjiang Bayan	Chongqing Tongnan	50867	57409
[1734,]	1708	1728	Zhejiang Yongjia	Chongqing Dazu	58658	57502
[1735,]	1725	1726	Chongqing Wanzhou	Chongqing Zhongxian	57432	57437
[1736,]	1727	1733	Chongqing Shizhu	Chongqing Beibei	57438	57511
[1737,]	1730	1732	Chongqing Yongchuan	Chongqing Tongliang	57506	57510
[1738,]	1734	1736	Chongqing Yubei	Chongqing Jiangjin	57513	57517
[1739,]	1685	1735	Zhejiang Shengzhou	Chongqing Bishan	58556	57514
[1740,]	1729	1731	Chongqing Rongchang	Chongqing Wansheng	57505	57509
[1741,]	1738	1739	Chongqing Nanchuan	Chongqing Changshou	57519	57520
[1742,]	1737	1740	Chongqing Banan	Chongqing Fengdu	57518	57523
[1743,]	1741	1742	Chongqing Wulong	Chongqing Qianjiang	57525	57536

Additional Legends to Figures

Fig. 1. The 782 isolated places in upper panel include 90 in Inner Mongolia, 85 in Yunnan, 60 in Xinjiang, 56 in Heilongjiang, 56 in Shaanxi, 53 in Hebei, 49 in Jiangxi, 43 in Hubei, 33 in Qinghai, 32 in Sichuan, 30 in Gansu, 26 in Jiangsu, 23 in Jilin, 18 in Guangxi, 14 in Chongqing, 14 in Henan, 13 in Shanxi, 12 in Hunan, 12 in Ningxia, 11 in Hainan, 11 in Shandong, 10 in Anhui, 9 in Guizhou, 6 in Fujian, 4 in Liaoning, 4 in Tianjin, 3 in Guangdong, 2 in Beijing, 2 in Shanghai, and 1 in Tibet.

The 816 isolated places in lower panel include 91 in Yunnan, 85 in Inner, 60 in Gansu, 59 in Shaanxi, 55 in Guizhou, 54 in Xinjiang, 46 in Jiangxi, 45 in Hebei, 38 in Heilongjiang, 38 in Sichuan, 34 in Guangxi, 32 in Hubei, 31 in Qinghai, 29 in Jiangsu, 15 in Jilin, 14 in Chongqing, 12 in Hainan, 11 in Anhui, 11 in Ningxia, 10 in Fujian, 10 in Shanxi, 9 in Hunan, 8 in Henan, 4 in Beijing, 4 in Shandong, 3 in Liaoning, 3 in Tianjin, 2 in Shanghai, 1 in Guangdong, 1 in Tibet, and 1 in Zhejiang.

Fig. 3. For upper panel, cluster A contains 330 places including 51 from Anhui, 5 from Fujian, 2 from Guansu, 60 from Guangdong, 22 from Guizhou, 4 from Hainan, 8 from Hubei, 67 from Hunan, 76 from Jiangxi, 5 from Ningxia, 2 from Shaanxi, 1 from Sichuan, 1 from Yunnan, 3 from Zhejiang, 23 from Chongqing; cluster B contains 240 places including 1 from Anhui, 29 from Fujian, 3 from Gansu. 33 from Guizhou, 6 from Henan, 52 from Hubei, 1 from Hunan, 46 from Jiangsu, 1 from Inner Mongolia, 1 from Ningxia, 3 from Shandong, 5 from Shanxi, 55 from Shaanxi, 1 from Yunnan, 2 from Zhejiang; cluster C contains 173 places including 3 from Guangdong, 57 from Guangxi, 7 from Guizhou, 2 from Hebei, 1 from Hunan, 9 from Shaanxi, 3 from Sichuan, 3 from Tianjin, 86 from Yunnan; cluster D contains 172 places including 34 from Gansu, 1 from Heilongjiang, 1 from Jilin, 18 from Liaoning, 63 from Inner Mongolia, 10 from Ningxia, 5 from Qinghai, 40 from Xinjiang; cluster E contains 145 places including 1 from Gansu, 3 from Hebei, 1 from Henan, 27 from Heilongjiang, 22 from Jilin, 24 from Qinghai, 67 from Shanxi; cluster F contains 119 places including 2 from Gansu, 1 from Qinghai, 1 from Shaanxi, 108 from Sichuan, 2 from Yunnan, 4 from Chongqing; cluster G contains 102 places including 12 from Gansu, 41 from Heilongjiang, 3 from Jilin, 22 from Liaoning, 8 from Inner Mongolia, 16 from Xinjiang; cluster H contains 98 places including 5 from Anhui, 14 from Gansu, 4 from Hebei, 72 from Henan, 2 from Shandong, 1 from Shanxi; cluster I contains 93 places including 4 from Beijing, 85 from Hebei, 1 from Liaoning, 1 from Inner Mongolia, 2 from Tianjin; cluster J contains 79 places including 1 from Hebei, 2 from Henan, 1 from Jilin, 71 from Shandong, 3 from Shanxi; cluster K contains 64 places including 14 from Fujian, 1 from Shanghai, 49 from Zhejiang; cluster L contains 33 places including 1 from Hebei, 3 from Heilongjiang, 1 from Jilin, 2 from Ningxia, 2 from Sichuan, 1 from Tianjin, 20 from Tibet, 2 from Xinjiang; cluster M contains 30 places including 1 from Hebei, 1 from Liaoning, 22 from Inner Mongolia, 6 from Xinjiang; and cluster N contains 17 places including 1 from Heilongjiang, 16 from Jilin (Table A1 in Supplementary Materials).

For lower panel, cluster A contains 401 places including 9 from Anhui, 48 from Fujian, 14 from Gansu, 47 from Guangdong, 5 from Hebei, 72 from Henan, 7 from Hubei, 61 from Hunan, 1 from Jiangsu, 66 from Jiangxi, 3 from Inner Mongolia, 4 from Ningxia, 2 from

Shandong, 50 from Shanxi, 1 from Shanghai, 2 from Xinjiang, 9 from Zhejiang; cluster B contains 233 places including 47 from Anhui, 2 from Gansu, 16 from Guangdong, 56 from Guizhou, 8 from Hainan, 1 from Hubei, 7 from Hunan, 8 from Jiangsu, 1 from Jiangxi, 2 from Ningxia, 2 from Shanxi, 14 from Shaanxi, 2 from Sichuan, 2 from Yunnan, 42 from Zhejiang, 23 from Chongqing; cluster C contains 190 places including 36 from Gansu, 1 from Hebei, 2 from Heilongjiang, 1 from Jilin, 10 from Liaoning, 62 from Inner Mongolia, 11 from Ningxia, 29 from Qinghai, 1 from Shaanxi, 37 from Xinjiang; cluster D contains 164 places including 4 from Beijing, 83 from Hebei, 2 from Henan, 1 from Liaoning, 72 from Shandong, 2 from Tianjin; cluster E contains 157 places including 1 from Anhui, 1 from Gansu, 4 from Hainan, 6 from Henan, 52 from Hubei, 46 from Jiangsu, 1 from Inner Mongolia, 3 from Shandong, 4 from Shanxi, 38 from Shaanxi, 1 from Zhejiang; cluster F contains 138 places including 12 from Gansu, 3 from Hebei, 1 from Henan, 42 from Heilongjiang, 6 from Liaoning, 29 from Inner Mongolia, 1 from Ningxia, 21 from Shanxi, 23 from Xinjiang; cluster G contains 118 places including 2 from Gansu, 1 from Qinghai, 1 from Shaanxi, 108 from Sichuan, 2 from Yunnan, 4 from Chongqing; cluster H contains 95 places including 3 from Guizhou, 4 from Sichuan, 86 from Yunnan, 2 from Zhejiang; cluster I contains 83 places including 1 from Gansu, 1 from Guangdong, 57 from Guangxi, 4 from Guizhou, 2 from Hebei, 1 from Hunan, 12 from Shaanxi, 4 from Tianjin; cluster J contains 67 places including 27 from Heilongjiang, 40 from Jilin; and cluster K contains 31 places including 2 from Beijing, 1 from Hebei, 2 from Heilongjiang, 1 from Shaanxi, 2 from Sichuan, 21 from Tibet, 2 from Xinjiang (Table A2 in Supplementary Materials).

Fig. 4. For upper panel, cluster A contains 536 places including 64 from Gansu, 4 from Hebei, 1 from Henan, 78 from Heilongjiang, 47 from Jilin, 41 from Liaoning, 93 from Inner Mongolia, 18 from Ningxia, 32 from Qinghai, 67 from Shanxi, 2 from Shaanxi, 2 from Sichuan, 21 from Tibet, 66 from Xinjiang; cluster B contains 392 places including 6 from Anhui, 11 from Beijing, 2 from Gansu, 94 from Hebei, 73 from Henan, 1 from Hubei, 46 from Jiangsu, 1 from Liaoning, 1 from Inner Mongolia, 77 from Shandong, 10 from Shanxi, 61 from Shaanxi, 6 from Tianjin, 1 from Tibet, 2 from Yunnan; cluster C contains 348 places including 2 from Gansu, 1 from Guangdong, 2 from Guangxi, 60 from Guizhou, 7 from Henan, 58 from Hubei, 69 from Hunan, 4 from Jiangxi, 1 from Qinghai, 4 from Shaanxi, 109 from Sichuan, 4 from Yunnan, 27 from Chongqing; cluster D contains 139 places including 2 from Fujian, 62 from Guangdong, 55 from Guangxi, 1 from Guizhou, 12 from Hainan, 1 from Jilin, 3 from Jiangxi, 2 from Yunnan; cluster E contains 113 places including 49 from Anhui, 10 from Jiangsu, 4 from Shanghai, 50 from Zhejiang; cluster F contains 98 places including 2 from Guangxi, 2 from Guizhou, 5 from Sichuan, 89 from Yunnan; and cluster G contains 67 places including 2 from Anhui, 2 from Fujian, 1 from Hubei, 60 from Jiangxi, 2 from Zhejiang; cluster H contains 47 places including 44 from Fujian, 1 from Guangxi, 2 from Zhejiang. Two isolated places are Heilongjiang Beijicun, 50137 and Yunnan Jinping, 56987 (Table A3 in Supplementary Materials).

For lower panel, cluster A contains 535 places including 63 from Gansu, 4 from Hebei, 1 from Henan, 79 from Heilongjiang, 47 from Jilin, 40 from Liaoning, 94 from Inner Mongolia, 18 from Ningxia, 32 from Qinghai, 67 from Shanxi, 1 from Shaanxi, 2 from Sichuan, 21 from Tibet, 66 from Xinjiang; cluster B contains 392 places including 6 from Anhui, 11 from Beijing,

3 from Gansu, 94 from Hebei, 73 from Henan, 45 from Jiangsu, 2 from Liaoning, 1 from Inner Mongolia, 77 from Shandong, 10 from Shanxi, 62 from Shaanxi, 6 from Tianjin, 1 from Tibet; cluster C contains 345 places including 2 from Gansu, 1 from Guangxi, 60 from Guizhou, 7 from Henan, 58 from Hubei, 68 from Hunan, 4 from Jiangxi, 1 from Qinghai, 4 from Shaanxi, 109 from Sichuan, 4 from Yunnan, 27 from Chongqing; cluster D contains 140 places including 2 from Fujian, 63 from Guangdong, 56 from Guangxi, 1 from Guizhou, 12 from Hainan, 1 from Jilin, 3 from Jiangxi, 2 from Yunnan; cluster E contains 114 places including 49 from Anhui, 11 from Jiangsu, 4 from Shanghai, 50 from Zhejiang; cluster F contains 99 places including 2 from Guangxi, 2 from Guizhou, 5 from Sichuan, 90 from Yunnan; cluster G contains 67 places including 2 from Anhui, 2 from Fujian, 1 from Hubei, 60 from Jiangxi, 2 from Zhejiang; and cluster H contains 46 places including 44 from Fujian, 2 from Zhejiang (Table A4 in Supplementary Materials).

Fig. 5 For upper panel, cluster A contains 649 places including 2 from Beijing, 62 from Gansu, 89 from Hebei, 3 from Henan, 2 from Heilongjiang, 1 from Hubei, 43 from Jilin, 42 from Liaoning, 11 from Inner Mongolia, 17 from Ningxia, 1 from Qinghai, 71 from Shandong, 76 from Shanxi, 64 from Shaanxi, 107 from Sichuan, 6 from Tianjin, 1 from Tibet, 44 from Xinjiang, 2 from Yunnan, 5 from Chongqing; cluster B contains 416 places including 55 from Anhui, 1 from Guangxi, 57 from Guizhou, 4 from Hebei, 78 from Henan, 58 from Hubei, 63 from Hunan, 56 from Jiangsu, 4 from Jiangxi, 6 from Shandong, 1 from Shanxi, 3 from Shaanxi, 3 from Shanghai, 2 from Sichuan, 2 from Yunnan, 1 from Zhejiang, 22 from Chongqing; cluster C contains 158 places including 2 from Anhui, 45 from Fujian, 1 from Hubei, 1 from Hunan, 56 from Jiangxi, 1 from Shanghai, 52 from Zhejiang; cluster D contains 150 places including 1 from Gansu, 1 from Hebei, 67 from Heilongjiang, 1 from Jilin, 76 from Inner Mongolia, 1 from Ningxia, 1 from Sichuan, 2 from Xinjiang; cluster E contains 138 places including 3 from Fujian, 63 from Guangdong, 57 from Guangxi, 4 from Guizhou, 5 from Hunan, 6 from Jiangxi; cluster F contains 92 places including 2 from Guizhou, 5 from Sichuan, 85 from Yunnan; cluster G contains 29 places including from Guangxi, 10 from Heilongjiang, 4 from Jilin, 1 from Tibet, 4 from Xinjiang, 8 from Yunnan; cluster H contains 27 places including 1 from Gansu, 26 from Qinghai; cluster I contains 13 places including 9 from Beijing, 4 from Hebei; cluster J contains 12 places including from Hainan; and cluster K contains 10 places including from Tibet (Table A5 in Supplementary Materials).

For lower panel, cluster A contains 697 places including 11 from Beijing, 64 from Gansu, 93 from Hebei, 3 from Henan, 3 from Heilongjiang, 1 from Hubei, 43 from Jilin, 42 from Liaoning, 12 from Inner Mongolia, 17 from Ningxia, 26 from Qinghai, 71 from Shandong, 76 from Shanxi, 64 from Shaanxi, 109 from Sichuan, 6 from Tianjin, 3 from Tibet, 46 from Xinjiang, 2 from Yunnan, 5 from Chongqing; cluster B contains 415 places including 55 from Anhui, 1 from Guangxi, 57 from Guizhou, 4 from Hebei, 78 from Henan, 58 from Hubei, 63 from Hunan, 56 from Jiangsu, 4 from Jiangxi, 6 from Shandong, 1 from Shanxi, 3 from Shaanxi, 3 from Shanghai, 2 from Sichuan, 1 from Yunnan, 1 from Zhejiang, 22 from Chongqing; cluster C contains 159 places including 2 from Anhui, 45 from Fujian, 1 from Hubei, 1 from Hunan, 57 from Jiangxi, 1 from Shanghai, 52 from Zhejiang; cluster D contains 152 places including 1 from Hebei, 66 from Heilongjiang, 1 from Jilin, 75 from Inner Mongolia, 1 from Ningxia, 8 from Tibet; cluster E contains 137 places including 3 from Fujian, 64 from

Guangdong, 56 from Guangxi, 4 from Guizhou, 5 from Hunan, 5 from Jiangxi; cluster F contains 90 places including 2 from Guizhou, 4 from Sichuan, 84 from Yunnan; cluster G contains 29 places including 2 from Guangxi, 10 from Heilongjiang, 4 from Jilin, 1 from Tibet, 4 from Xinjiang, 8 from Yunnan; and cluster H contains 11 places from Hainan (Table A6 in Supplementary Materials).
